# Fluorescent turn-on probes for wash-free mRNA imaging *via* covalent site-specific enzymatic labeling†
†Electronic supplementary information (ESI) available. See DOI: 10.1039/c7sc03150e



**DOI:** 10.1039/c7sc03150e

**Published:** 2017-08-29

**Authors:** Cun Yu Zhou, Seth C. Alexander, Neal K. Devaraj

**Affiliations:** a Department of Chemistry and Biochemistry , University of California , 9500 Gilman Dr La Jolla , San Diego , CA 92093 , USA . Email: ndevaraj@ucsd.edu

## Abstract

Investigating the many roles RNA plays in cellular regulation and function has increased demand for tools to explore RNA tracking and localization within cells. Our recently reported RNA-TAG (transglycosylation at guanine) approach uses an RNA-modifying enzyme, tRNA-guanine transglycosylase (TGT), to accomplish covalent labeling of an RNA of interest with fluorescent tracking agents in a highly selective and efficient manner. Unfortunately, labeling by this method currently suffers from a high nonspecific fluorescent background and is currently unsuitable for imaging RNA within complex cellular environments. Herein we report the design and synthesis of novel fluorogenic thiazole orange probes that significantly lower nonspecific binding and background fluorescence and, as a result, provide up to a 100-fold fluorescence intensity increase after labeling. Using these fluorogenic labeling agents, we were able to image mRNA expressed in Chinese Hamster Ovary cells in a wash-free manner.

## Abstract 2

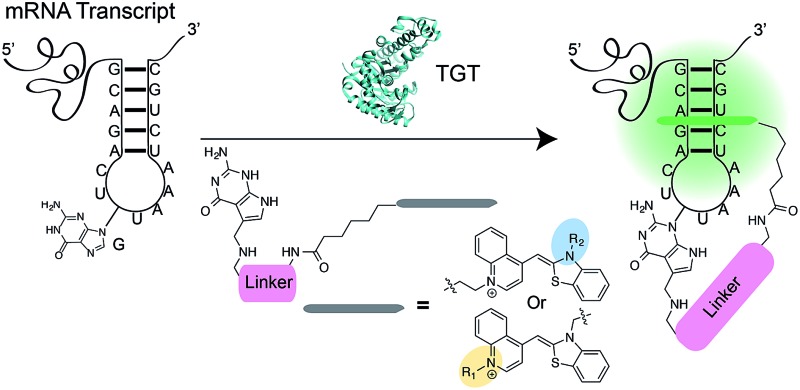
Investigating the many roles RNA plays in cellular regulation and function has increased demand for tools to explore RNA tracking and localization within cells.

## Introduction

RNA has been shown to play numerous roles in the regulation of a range of cellular biochemical processes.^1^ Recent advances in chemical biology have enabled the discovery of novel RNA structures and functions in cells.^2^ These discoveries have potential applications in understanding and treating disease^3–5^ as well as accelerating the development of RNA as a therapeutic target.^6–8^ Labeling RNA with imaging agents enables tracking of individual RNAs within cells, potentially linking localization and concentration of the RNA with specific functions.^9,10^ Conventional methodologies used for RNA detection include antisense probes,^11–13^ aptamers,^14,15^ molecular beacons^16^ and fusion proteins that recognize specific RNA secondary structures.^17,18^ These approaches rely on reversible non-covalent interactions between the imaging agent and RNA, limiting robustness for applications where irreversible linkage of the imaging agent and RNA would be preferred.^19,20^ The exploration of RNA-modifying enzymes capable of covalently modifying RNA with tracking molecules has been a major thrust to address this shortcoming.^19,21^ For example, Rentmeister and co-workers have successfully harnessed an mRNA capping enzyme, trimethylguanosine synthase (GlaTgs2), to attach small functional handles site-specifically at the 5′ cap of cellular mRNAs.^22,23^ Additionally, the tRNA modifying enzyme Tias has also been shown to accept small primary amines bearing azide or alkyne handles for subsequent labeling with fluorescent agents; however the enzyme requires the entire tRNA structure to be incorporated into the RNA of interest, as well as millimolar concentration of propargylamine for successful incorporation.^24^ Unfortunately, both approaches suffer from the necessity of secondary “click” reactions.

**Fig. 1:**
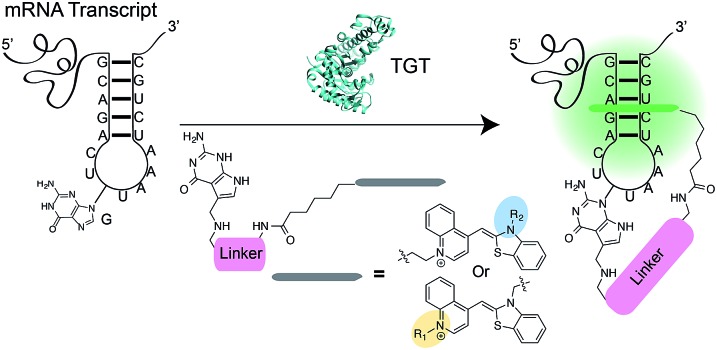
Schematic representation of RNA-TAG labeling using the bacterial TGT enzyme with preQ_1_-TO probes. Upon the exchange of the guanine with the preQ_1_-TO probe within the recognition element of the mRNA, the TO fluorophore likely intercalates to the RNA of interest leading to a dramatic increase in fluorescence intensity.

Recently, our group introduced a covalent labeling strategy, RNA-TAG (transglycosylation at guanosine), capable of site-selectively and covalently modifying an RNA of interest with fluorophores and affinity handles. The technique relies on hijacking a bacterial tRNA-guanine transglycosylase (TGT) enzyme.^25^ TGT recognizes and exchanges a specific guanine residue for a preQ_1_ derivative within a short (17-nt) hairpin structural element,^26,27^ which can be genetically encoded into an RNA of interest (Fig. 1).^25^

**Fig. 2:**
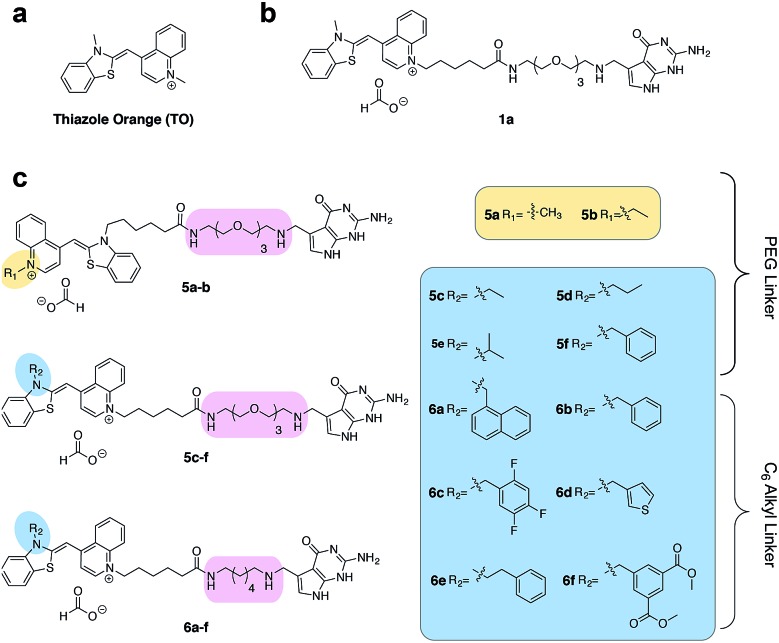
(a) The structure of thiazole orange (b) the structure of a previously synthesized preQ_1_-PEG3-TO-Me **1a** (c) structures of modified preQ_1_-TO probes that show enhanced fluorescent turn-on.

Asymmetric cyanine dyes such as thiazole orange (TO) (Fig. 2a) are well poised to detect RNA as they emit a strong fluorescence upon binding nucleic acids.^28,29^ TO's fluorogenic interaction with nucleic acids can give up to 1000-fold fluorescent enhancement, and TO derivatives have been widely adopted in a variety of PNA and DNA forced-intercalating (FIT) probes,^13,30–32^ ECHO probes,^33–35^ an RNA GTP sensor,^28^ and fluorogenic RNA aptamers such as RNA mango.^15^ In our previous work, we chemically modified TGT's natural substrate, preQ_1_, with a TO moiety to yield **1a** (Fig. 2b) and observed a strong fluorescence increase upon covalent incorporation into a short (17-nt) RNA hairpin. However when a full-length mRNA transcript was modified, the increase was reduced to only 3-fold due to non-specific binding with RNA.^25^ Unfortunately, the observed nonspecific RNA background fluorescence prevented successful imaging of the target RNA amongst the complexity of the cell (Fig. S1†). To address these challenges, we investigated an array of preQ_1_-TO derivatives designed to reduce nonspecific RNA binding, while still eliciting a fluorogenic response upon covalent incorporation by RNA-TAG (Fig. 2c). The nucleic acid promoted fluorogenicity of TO is derived from favorable binding of the planar and positively charged molecular structure to the minor groove of negatively charged nucleic acid polymers.^29^ We envisioned that installation of a bulky substituent on the TO moiety would disfavor nonspecific binding to nucleic acids and thus lower the fluorescent background. Meanwhile, covalent linkage with the target RNA will drastically increase the effective molarity of the TO probe, thus promoting a fluorescent bound state.^28,36^

## Results and discussion

We first synthesized an array of preQ_1_-TO derivatives by incorporating bulky side chains on the TO scaffold at the benzothiazole's N3 or quinoline's N1 positions (Fig. 2c, Schemes S1 and S2†). 2-Methylbenzonthiazole and 4-methylthioquinoline were modified with various alkyl halides to yield the corresponding quaternary ammonium salts **2a–j** and **3a–c**. Following previously established protocols, the thiazole orange core structure **4a–k** can then be conveniently prepared in a high yield by coupling alkylated benzothiazolium **2a–i** and quinolinium with a carboxylic handle **3c**, or alkylated quinolinium **3a–b** and benzothiazolium with a carboxylic handle **2j** in the presence of triethylamine (Schemes S3 and S4†). The TO-carboxylic acid **4a–k** was further coupled to a preQ_1_-PEG3-NH_2_ or preQ_1_-C6-NH_2_ to yield the corresponding preQ_1_-TO derivatives **5a–f** and **6a–f** (Fig. 2c and Schemes S5–S7†).

**Fig. 3:**
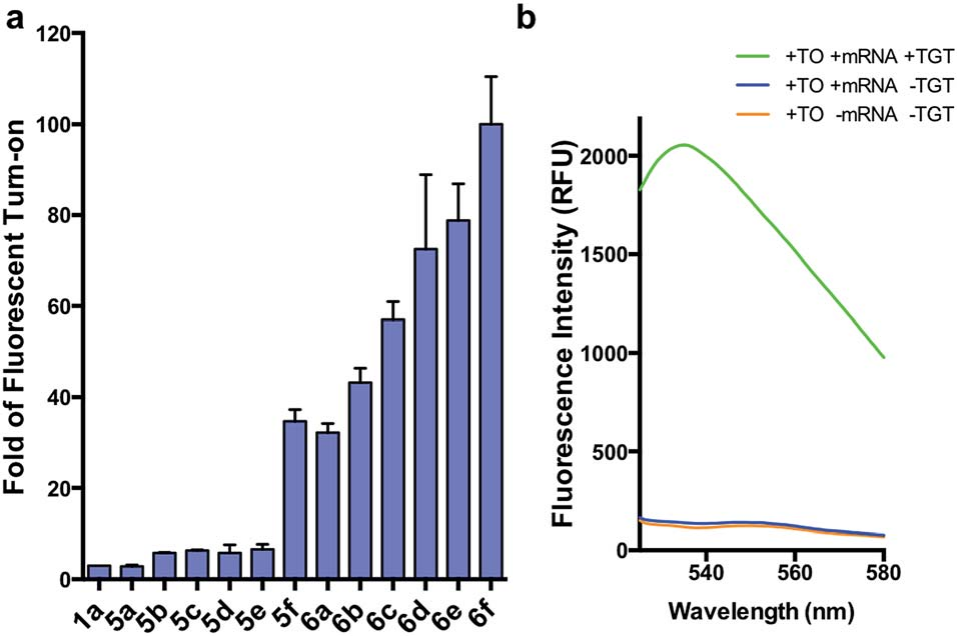
(a) Fold fluorescent turn-on of **5** and **6** when covalently linked to mCherry mRNA *via* transglycosylation reactions. The probes are ranked in the ascending order of fluorescent turn-on. All fluorescent measurements were performed in triplicates. Error bars denote standard deviation (*n* = 3) (b) emission spectra of **6f** demonstrating an approximate 100-fold fluorescent enhancement when the mRNA is labeled. All spectra represent the average of three runs.

We initially prepared a small collection of preQ_1_-PEG3-TO probes, **5a–f** that are structurally similar to a previously reported probe, **1a**.^25^ We chose to examine alternative points of attachment of our probes to PreQ_1_ (**5a–b**), as well as the alkyl groups at the TO moiety to an ethyl, propyl, isopropyl, and benzyl group, thus gradually increasing the steric bulk (**5c–f**). With these derivatives in hand, we first investigated probe background fluorescence in the absence of enzyme at various concentrations of *in vitro* transcribed mCherry mRNA, which contained a 17 nt TGT recognition element within the 3′ UTR. We observed that as steric bulk on the TO moiety increased, the derivative emitted less fluorescence (Me > Et > Pr > iPr ≈ Bn) (Fig. S2†) likely due to reduced nonspecific interaction between the probe and RNA. We next tested the RNA-TAG labeling reaction with our collection of probes **5a–f** *in vitro* to examine relative fluorescent turn-on after enzymatic incorporation onto the mCherry transcript. Relative fluorescence was measured following treatment of the target mRNA with 1 μM PreQ_1_-TO probe and 1 μM TGT for 2 h at 37 °C (Fig. 3a). Our results indicated that preQ_1_-TO derivatives containing aliphatic substitutions (**5a–e**) demonstrated little improvement over our first generation probe **1a**. However, **5f**, substituted with an aromatic benzyl group, demonstrated an improved 35-fold turn-on. We also evaluated the importance of the point of attachment on the TO moiety for methyl and ethyl substitutions. We found similar increases in fluorescence between methyl (**1a**, **5a**) and ethyl (**5b**, **5c**) substitutions of the two regioisomer pairs indicating that the substituent identity, rather than their location, is the dominating factor in minimizing non-specific background fluorescence. From this data we can conclude probe **5f** significantly lowers the fluorescent background from nonspecific interactions with nucleic acids while maintaining high fluorescent intensity once covalently linked through RNA-TAG enzymatic transglycosylation.

After investigating substitution of the TO derivatives **5a–f**, we next explored an alternative linker to improve kinetics of transglycosylation. We examined what effect replacement of the hydrophilic PEG3 linker by a more hydrophobic 6-carbon alkyl linker would have on labeling efficiency. Using HPLC to quantify reaction completion, we observed that when 10 μM 17-nt ECY-A1 hairpin was treated with 1 μM (0.1 eq.) TGT and 10 μM **1a**, approximately 65% of the substrate was labeled after 2 h. However, use of **1b**, which employed an alkyl linker (Scheme S8†), resulted in nearly quantitative labeling under identical conditions (Fig. S3†). In light of these results, we employed the C6 alkyl linker exclusively to further improve fluorogenic preQ_1_-TO probes bearing aromatic substitutions.

We next synthesized probes **6a–f**, derivatized with a variety of aromatic substitutions (Fig. 2c). We measured the relative observed fluorescence before and after covalent incorporation of our alkyl linker derivatives, **6a–f**, onto the mCherry transcript (Fig. 3a). From this screening we were delighted that substrate **6f** elicited a remarkable 100-fold turn on when covalently conjugated to mRNA. The quantum yield of mRNA labeled **6f** was determined to be 0.167 ± 0.009, with free **6f** in solution less than 0.001. The quantum yield value and its increase upon covalent labeling is comparable to that of the DNA FIT probe with TO as the fluorophore.^13^ We next estimated the enzymatic incorporation kinetics of **6f** into ECY-A1 hairpin following a previously established protocol.^25^ We determined a much improved estimated rate, *k*_cat_ = 26.7 × 10^–3^ s^–1^, and binding affinity, *K*_M_ = 1.6 μM (Fig. S4a–e†), when compared to the previously reported *k*_cat_ = 1.6 × 10^–3^ s^–1^, and *K*_M_ = 9.8 μM of **1a**.^25^ The lowered *K*_M_ implies less probe can be employed for cell imaging which should result in less background staining and further improve the signal-to-background ratio.

**Fig. 4:**
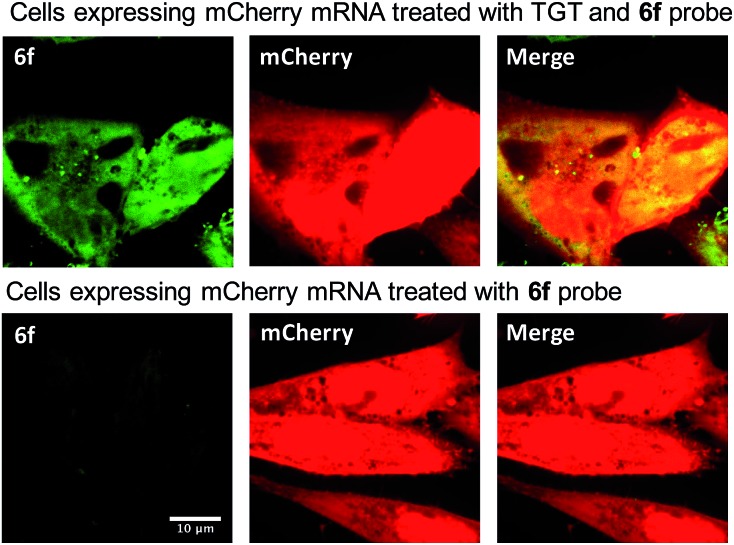
Imaging of the mCherry mRNA expressed in CHO cells using optimized preQ_1_-C6-TO probe **6f**. Cells were transfected with mCherry plasmid overnight. The cells were fixed and permeabilized before the treatment of 0.5 μM probe and 0.5 μM TGT in TGT reaction buffer. Bright green fluorescence is observed only in the presence of bacterial TGT. The red fluorescence indicates the successful expression of mCherry protein. Right column shows the merge of the TO and mCherry channels.

We next sought to apply these novel fluorogenic probes to visualize a single RNA sequence within the context of a complex cellular environment. mRNA localization is known to be critical for spatial and temporal expression of proteins and essential for cell development and physiology.^37^ Fixed cells retain the structural organization of the cellular contents, making visualization of the cellular distribution of mRNA possible.^38^ Chinese Hamster Ovary (CHO) cells were transiently transfected with the mCherry construct plasmid. After overnight culture, the cells were fixed, permeabilized, and subsequently treated with 0.5 μM **6f** and 0.5 μM TGT. Cells were then incubated for 3 h and subsequently imaged without a wash step. Significantly greater staining was observed for cells treated with TGT and **6f** compared with control cells that were; (1) not treated with TGT, (2) not transfected with the mCherry construct, and (3) only treated with **6f** in the absence of TGT and mRNA expression (Fig. 4 and S5†). The fluorogenic properties of the preQ_1_-TO derivatives make them a useful choice for cellular imaging in situations where an intense fluorescent signal with a clear contrast is critical to differentiate RNA specific signal from that of background probe staining. A wash free labeling system is particularly suitable to image smaller RNA targets in fixed cells that might be washed away, and could be valuable for future live cell RNA imaging applications, where a stringent washout of excess probe is not possible.^39^

In our effort to image mRNA in live cells using RNA-TAG, we transiently co-transfected HeLa cells with a plasmid capable of expressing TGT in mammalian cells and the mCherry construct plasmid used in our previous experiments (Fig. S6†). The TGT and mCherry co-expressing cells were subsequently treated with 0.5 μM of **6f** and incubated for 4 hours. Unfortunately, we did not observe significantly different fluorescent brightness between the transfected and non-transfected cells. Excessive washing of the live cells to remove the excess probes also did not reveal a significant fluorescent difference. Because it is possible that HeLa cells do not effectively express functional bacterial TGT, we microinjected a mixture of **6f** and purified TGT into HeLa cells. However, no significant difference in fluorescence between the TGT and no-TGT treated cells was observed (Fig. S6†). We therefore hypothesize that imaging of RNA in live cells are intrinsically difficult due to their significantly low cellular concentration (∼100 pM).^40^ A precise control of the imaging probe concentration is critical in the successful enzymatic labeling of RNA. An ideal probe concentration must leverage being low enough to avoid background staining but also high enough to be near to the *K*_M_ of TGT (1.6 μM) to be recognized by the enzyme efficiently. We support this hypothesis with evidence from a fixed cell imaging experiment with elevated probe concentration demonstrating that when using 2 μM of **6f** (4× our previously used concentration), RNA could not be efficiently imaged due to a high fluorescent background (Fig. S7†). Live cell imaging through microinjecting the probe and enzyme was unsuccessful, presumably due to the difficulty in the control of final imaging probe concentration in individual cells within such a narrow optimal concentration window. Future work will focus on adapting our system to live cell applications by engineering a more efficient TGT variant that requires a lower *K*_M_ for the RNA and preQ_1_ probe through directed evolution. We believe this could greatly benefit the labelling of the RNA *in vivo*. In order to increase the signal-to-noise ratio for the *in vivo* fluorescently labeled RNA, we also plan to construct multiple TAG sequence repeats genetically engineered in the target mRNA to increase the chances of labeling and abundance of signal per RNA.

## Conclusions

We have developed a new class of preQ_1_-TO fluorogenic probes for imaging mRNA in mammalian cells using RNA-TAG. The advantages of using RNA-TAG to image mRNA in cells include fast labeling kinetics, an easy preparation of the fluorogenic probes, and a submicromolar working concentration of both enzyme and probe. These novel preQ_1_-TO probes greatly reduced the fluorescent background and maintained a high fluorescent intensity after labeling. By exploring the structure activity relationship of preQ_1_-TO probes, we were able to optimize fluorogenic RNA-TAG labeling for cellular imaging. Our optimal probe, a benzyl substituted TO probe linked to preQ_1_ *via* a C6 linker, demonstrated greatly improved labeling kinetics and a 100-fold increase in fluorescence intensity upon covalent incorporation to a full length mRNA transcript. We believe that this robust and versatile technology will provide a powerful tool to detect and image RNAs of both fundamental and practical interest. We are currently exploring expanding this technology to enable live-cell detection of expressed mRNAs.

## Conflicts of interest

There are no conflicts to declare.
