# Supplementary material for: Fluorescent turn-on probes for wash-free mRNA imaging via covalent site-specific enzymatic labeling
Source: Chem Sci. 2017 Aug 29;8(10):7169–73. doi: 10.1039/c7sc03150e (PMC5635419; doi:10.1039/c7sc03150e)
Supplement: Supplementary file 1 [file SC-008-C7SC03150E-s001.pdf]

Electronic Supplementary Material (ESI) for Chemical Science.
This journal is © The Royal Society of Chemistry 2017

Fluorescent Turn-on Probes for Wash-Free mRNA Imaging via Covalent Site-Specific Enzymatic Labeling

Cun Yu Zhou, Seth C. Alexander, Neal K. Devaraj*

Department of Chemistry and Biochemistry, University of California, San Diego, La Jolla, California 92093

Supplementary Information

1. General Methods S3
2. Buffers S3
3. General procedure for synthesis of 3-alkyl-2-methylbenzothiazolium halide **2a-2j** S3
    Scheme S1. Synthesis of 3-alkyl-2-methylbenzothiazolium halide **2a-j** S3
4. General procedure for synthesis of 1-alkyl-4-methylthioquinolinium halide **3a-3c** S5
    Scheme S2. Synthesis of 1-alkyl-4-methylthioquinolinium halide **3a-c** S5
5. General procedure for synthesis of alkylated thiazole orange carboxylic acids **4a-4k** S6
    Scheme S3. Synthesis of alkylated thiazole orange carboxylic acids **4a-I** S6
    Scheme S4. Synthesis of alkylated thiazole orange carboxylic acids **4j-k** S8
6. General procedure for synthesis of preQ$_1$-PEG3-TO **5a-5f** S9
    Scheme S5. Synthesis of preQ$_1$-PEG3-TO **5a-b** S9
    Scheme S6. Synthesis of preQ$_1$-PEG3-TO **5c-f** S10
7. General procedure for synthesis of preQ$_1$-C6-TO **6a-6f** S11
    Scheme S7. Synthesis of preQ$_1$-C6-TO **6a-f** S11
8. Synthesis of preQ$_1$-C6-TO-Me **1b** S13
    Scheme S8. Synthesis of preQ$_1$-C6-TO-Me **1b** S13
9. Synthesis of preQ$_1$-C6-NHBoc **7** S14
    Scheme S9. Synthesis of preQ$_1$-C6-NHBoc **7** S14
10. Synthesis of preQ$_1$-C6-NH$_2$ **8** S14
    Scheme S10. Synthesis of preQ$_1$-C6-NH$_2$ **8** S14
11. Fixed cell labeling of mCherry mRNA with **1a** S15
    Fig. S1. TGT labeling of mCherry mRNA with **1a** S15
12. Preparation of plasmid construct S15
    12.1 mCherry plasmid S15
    12.2 CMV-TGT expression vector S16
13. *In vitro* transcription of mCherry mRNA S16

14. Fluorescent assay for TO **5a-f**/mCherry RNA binding S17

Fig. S2. Plot of raw fluorescence data for TO **5a-f**/mCherry RNA binding assay S17

15. Fluorescent turn-on measurements for mRNA labeling with **5a-f** and **6a-f** S17

16. Quantum yield measurements for mRNA labeled **6f** S17

17. ECY-A1 labeling efficiency with **1a** and **1b** S17

Fig. S3a-b. RNA labeling efficiency with PEG linker probe **1a** or alkyl linker probe **1b**. S18

18. Estimation of **6f** substrate kinetics with ECY-A1 RNA hairpin S19

Fig. S4a-e. Estimation of **6f** substrate kinetics with ECY-A1 RNA hairpin S20

19. Fixed cell labeling of mCherry mRNA S21

Fig. S5. Fluorescence microscopy of TGT labeling of CHO cell expressed mCherry mRNA. S21

20. Verification of TGT expression in HeLa cells by immunofluorescent imaging S22

Fig. S6. Varying TGT expression in HeLa cells. S22

21. Live cell labeling of mCherry mRNA with **6f** via microinjection S23

Fig. S7. Fluorescence microscopy images of microinjected HeLa cells S23

22. Fixed cell labeling of mCherry mRNA with increased **6f** concentration S24

Fig. S8. TGT labeling of expressed mRNA with increased **6f** concentration S24

23. Supporting information references S24

24. NMR spectra for synthesized material S25

1. General Methods

All reagents used for the synthesis of PreQ1-TO derivatives were purchased from Sigma-Aldrich (St. Louis, MO) and used without further purification. All DNA and RNA Oligonucleotides were purchased from Integrated DNA Technologies (Coralville, IA). All restriction enzymes, bio-reagents, nucleotide stains, and competent bacterial strains were purchased from New England Biolabs (Ipswitch, MA), Promega (Madison, WI), or Life Technologies (Carlsbad,CA). ^{1}H and ^{13}C NMR spectra were recorded on a Varian VX 500 MHz NMR Spectrometer. High resolution mass spectroscopy was collected on an Agilent Infinity 1260 LC and tandem Agilent 6230 high resolution time of flight (TOF) mass spectrometer managed by the UCSD Department of Chemistry and Biochemistry Molecular Mass Spectroscopy Facility. Reverse-phase HPLC purification and analysis were performed using an Agilent 1260 Infinity HPLC with Agilent 6120 Quadrapole mass spectrometer (Santa Clara, CA). PreQ1-TO derivatives were prepared and analyzed with an Agilent Zorbax SB-C18 semi-prep column (ID 9.4 x 250 mm, 5 µm, 80 Å) and an Agilent Zorbax eclipse plus C8 column, using a water/methanol gradient containing 0.1% TFA. Oligonucleotide HPLC analysis was carried out on a Phenomenex Clarity Oligo-MS analytical column (ID 2.1 x 50 mm, 2.6 µm, 100 Å) using a gradient of water containing 20% hexafluoroisopropanol (HFIP) and 0.1% TEA and methanol containing no additives. Fluorescence measurements were collected either on a JASCO FP-8500 fluorimeter (for spectral analysis and fluorescent turn-on assay) (Hachioji, Japan) or a Tecan Saphire-II plate reader (for kinetic analysis) (Tecan, Männedorf, Switzerland). Fluorescence microscopy images were acquired on an Axio Observer D1 inverted microscope (Carl Zeiss Microscopy GmbH, Germany) with a 20 x and 60 x and 1.42 NA air immersion objective and ORCA-ER camera (Hamamatsu, Japan) using the FLUOVIEW software package (Olympus, Japan). Fluorescent probes and proteins were excited with an argon laser at appropriate wavelengths. Images were subsequently analyzed and processed using ImageJ.

2. Buffers

TGT Storage Buffer: 25 mM HEPES, pH 7.3, 2 mM DTT, 1 mM EDTA, and 100 µM PMSF.
TGT Reaction Buffer: 100 mM HEPES, pH 7.3, 5 mM DTT, and 20 mM $MgCl_2$.
T7 Reaction Buffer: 40 mM Tris pH 7.5, 5 mM DTT, 25 mM $MgCl_2$, 2 mM spermidine.

3. General procedure for synthesis of 3-alkyl-2-methylbenzothiazolium halide **2a-j**

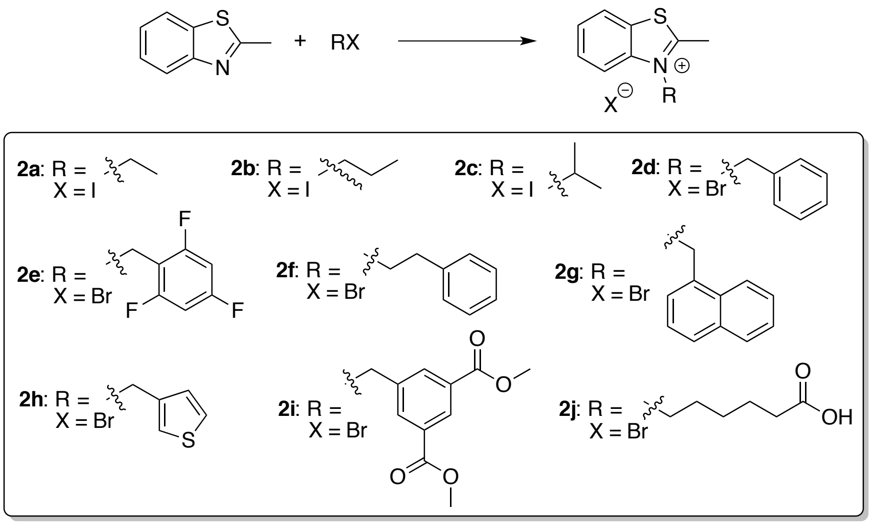

Scheme S1. Synthesis of 3-alkyl-2-methylbenzothiazolium halide **2a-j**

The synthesis of **2a-2j** was adopted from previous published protocol.[1,2] In a scintillation vial, 2-methylbenzothiazole and appropriate alkyl halide were mixed and stirred overnight under heat. The resulting solid was filtered and washed with diethylether. The product was used directly without further purification.

3-ethyl-2-methylbenzothiazol-3-ium iodide **2a**: A mixture of 298 mg (2 mmol) of 2-methylbenzothiazole and 780 mg (5 mmol) of ethyl iodide in 1 mL ACN was heated at 80 °C to yield 561 mg of **2a** as a light green solid in a 92% yield. ^{1}H NMR (500 MHz, CD_2Cl_2) δ 8.31 (d, *J* = 8.2 Hz, 1H), 8.07 (d, *J* = 8.4 Hz, 1H), 7.90 (t, *J* = 8.3 Hz, 1H), 7.80 (t, *J* = 8.1 Hz, 1H), 4.90 (q, *J* = 7.4 Hz, 2H), 3.40 (s, 3H), 1.65 (t, *J* = 7.4 Hz, 3H). ^{13}C NMR (126 MHz, CD_2Cl_2) δ 175.38, 141.25, 130.75, 129.74, 129.48, 125.05, 116.98, 47.20, 19.46, 14.29. HRMS $[M]^+$ m/z calcd. for $[C_{10}H_{12}NS]^+$ 178.0685, found 178.0683.

2-methyl-3-propylbenzothiazol-3-ium iodide **2b**: A mixture of 298 mg (2 mmol) of 2-methylbenzothiazole and 850 mg (5 mmol) of propyl iodide was heated at 80 °C to yield 517 mg of **2b** as a light grey solid in a 81% yield. ^{1}H NMR (500 MHz, $CDCl_3$) δ 8.36 (d, *J* = 8.2 Hz, 1H), 8.07 (d, *J* = 8.5 Hz, 1H), 7.81 (t, *J* = 7.9 Hz, 1H), 7.70 (t, *J* = 7.7 Hz, 1H), 4.84 (t, *J* = 7.9 Hz, 2H), 3.45 (s, 3H), 2.02 (h, *J* = 7.4 Hz, 2H), 1.11 (t, *J* = 7.4 Hz, 3H). ^{13}C NMR (126 MHz, $CDCl_3$) δ 175.22, 141.11, 130.12, 129.14, 128.81, 124.84, 116.79, 52.89, 22.38, 20.07, 11.55. HRMS $[M]^+$ m/z calcd. for $[C_{11}H_{14}NS]^+$ 192.0841, found 192.0841.

3-isopropyl-2-methylbenzothiazol-3-ium iodide **2c**: A mixture of 298 mg (2 mmol) of 2-methylbenzothiazole and 850 mg (5 mmol) of isopropyl iodide was heated at 150 °C to yield 274 mg of **2c** as a off-white solid in a 43% yield. ^{1}H NMR (500 MHz, DMSO-D6) δ 8.51 (d, *J* = 8.4 Hz, 1H), 8.47 (d, *J* = 8.1 Hz, 1H), 7.83 (t, *J* = 7.9 Hz, 1H), 7.78 (t, *J* = 7.5 Hz, 1H), 5.41 (s, 1H), 3.24 (s, 3H), 1.76 (d, *J* = 6.9 Hz, 6H). ^{13}C NMR (126 MHz, DMSO-D6) δ 177.42, 129.89, 129.51, 128.08, 125.36, 125.29, 118.67, 56.07, 19.95, 19.95, 18.44. HRMS $[M]^+$ m/z calcd. for $[C_{11}H_{14}NS]^+$ 192.0841, found 192.0841.

3-benzyl-2-methylbenzothiazol-3-ium bromide **2d**: A mixture of 140 mg (0.93 mmol) of 2-methylbenzothiazole and 210 mg (1.23 mmol) of benzyl bromide in 1 mL ACN was heated at 80 °C to yield 280 mg of **2d** as a light grey solid in a 93% yield. ^{1}H NMR (500 MHz, CD_2Cl_2) δ 8.35 (d, 1H), 8.02 (d, *J* = 8.5 Hz, 1H), 7.83 – 7.75 (m, 2H), 7.44 – 7.37 (m, 3H), 7.25 – 7.17 (m, 2H), 6.23 (s, 2H), 3.45 (s, 3H). ^{13}C NMR (126 MHz, CD_2Cl_2) δ 177.50, 142.12, 132.04, 130.75, 130.15, 130.15, 129.79, 129.75, 129.42, 127.29, 127.29, 125.06, 117.43, 54.37, 19.60. HRMS $[M]^+$ m/z calcd. for $[C_{15}H_{14}NS]^+$ 240.0841, found 240.0841.

2-methyl-3-(2,4,6-trifluorobenzyl)benzothiazol-3-ium bromide **2e**: A mixture of 597 mg (4 mmol) of 2-methylbenzothiazole and 210 mg (0.89 mmol) of 2-(bromomethyl)-1,3,5-trifluorobenzene was heated at 150 °C to yield 210 mg of **2e** as a white solid in a 63% yield. ^{1}H NMR (500 MHz, $CDCl_3$) δ 8.41 (d, *J* = 8.2 Hz, 1H), 7.87 (d, *J* = 8.6 Hz, 1H), 7.69 (t, 1H), 7.60 (t, *J* = 7.7 Hz, 1H), 6.76 (t, *J* = 8.2 Hz, 2H), 6.14 (s, 2H), 3.56 (s, 3H). ^{13}C NMR (126 MHz, $CDCl_3$) δ 178.32, 164.62, 162.72, 160.47, 140.96, 130.00, 128.91, 128.49, 125.20, 115.86, 104.80, 101.75, 101.75, 43.82, 19.62. HRMS $[M]^+$ m/z calcd. for $[C_{15}H_{11}NS]^+$ 294.0559, found 294.0557.

2-methyl-3-phenethylbenzothiazol-3-ium bromide **2f**: A mixture of 597 mg (4 mmol) of 2-methylbenzothiazole and 185 mg (1 mmol) of (2-bromoethyl)benzene was heated at 150 °C to yield 228 mg of **2f** as a white solid in a 68% yield. ^{1}H NMR (500 MHz, $CDCl_3$) δ 8.44 (d, *J* = 8.2 Hz, 1H), 8.16 (d, *J* = 8.5 Hz, 1H), 7.76 (t, *J* = 7.4 Hz, 1H), 7.65 (t, *J* = 7.7 Hz, 1H), 7.24 – 7.11 (m, 3H), 6.82 (d, *J* = 6.7 Hz, 2H), 5.20 (t, *J* = 7.2 Hz, 2H), 3.28 (t, *J* = 6.2 Hz, 2H), 2.64 (s, 3H). ^{13}C NMR (126 MHz, $CDCl_3$) δ 176.29, 140.62, 135.94, 130.05, 129.34, 129.34, 128.97, 128.88, 128.88, 128.64, 128.00, 125.07, 116.88, 53.01, 34.64, 18.13. HRMS $[M]^+$ m/z calcd. for $[C_{16}H_{16}NS]^+$ 254.0998, found 254.0995.

2-methyl-3-(naphthalen-1-ylmethyl)benzothiazol-3-ium bromide **2g**: A mixture of 597 mg (4 mmol) of 2-methylbenzothiazole and 221 mg (1 mmol) of 1-(bromomethyl)naphthalene was heated at 150 °C to yield 163 mg of **2g** as a white solid in a 44% yield. ^{1}H NMR (500 MHz, DMSO-D6) δ 8.58 (d, *J* = 7.8 Hz, 1H), 8.28 (d, *J* = 8.4 Hz, 1H), 8.05 (t, *J* = 7.6 Hz, 2H), 7.94 (d, *J* = 8.1 Hz, 1H), 7.91 – 7.74 (m, 3H), 7.69 (t, *J* = 7.9 Hz, 1H), 7.32 (t, *J* = 7.9 Hz, 1H), 6.62 (s, 2H), 6.52 (d, *J* = 7.2 Hz, 1H), 3.21 (s, 3H). ^{13}C NMR (126 MHz, DMSO-D6) δ 179.19, 146.72, 141.19, 133.37, 129.73, 129.63, 129.42, 128.59, 128.41, 128.27, 126.96, 126.71, 125.49, 124.94, 123.33, 121.65, 117.02, 50.16, 17.04. HRMS $[M]^+$ m/z calcd. for $[C_{19}H_{16}NS]^+$ 290.0998, found 290.0999.

2-methyl-3-(thiophen-3-ylmethyl)benzothiazol-3-ium bromide **2h**: A mixture of 597 mg (4 mmol) of 2-methylbenzothiazole and 177 mg (1 mmol) of 3-(bromomethyl)thiophene was heated at 150 °C to yield 277 mg of **2h** as a white solid in a 85% yield. ^{1}H NMR (500 MHz, CD_3OD) δ 8.34 (d, *J* = 8.2 Hz, 1H), 8.28 (d, *J* = 8.6 Hz, 1H), 7.89 (t, *J* = 7.5 Hz, 1H), 7.83 (t, *J* = 7.7 Hz, 1H), 7.57 – 7.52 (m, 1H), 7.50 (s, 1H), 7.13 (d, *J* = 5.0 Hz, 1H), 6.07 (s,

2H), 3.29 (s, 3H). ^{13}C NMR (126 MHz, CD_3OD) δ 178.01, 140.81, 133.14, 129.50, 129.27, 128.22, 128.00, 127.04, 125.17, 124.88, 117.08, 48.00, 17.33. HRMS $[M]^+$ m/z calcd. for $[C_{13}H_{12}NS]^+$ 246.0406, found 246.0404.

3-(3,5-bis(methoxycarbonyl)benzyl)-2-methylbenzothiazol-3-ium bromide **2i**: A mixture of 597 mg (4 mmol) of 2-methylbenzothiazole and 287 mg (1 mmol) of dimethyl 5-(bromomethyl)isophthalate[3] was heated at 150 °C to yield 392 mg of **2i** as a white solid in a 90% yield. ^{1}H NMR (500 MHz, DMSO-D6) δ 8.54 (d, J = 8.1 Hz, 1H), 8.42 (s, 1H), 8.27 (d, J = 8.4 Hz, 1H), 8.11 (s, 2H), 7.89 – 7.81 (m, 2H), 6.29 (s, 2H), 3.86 (s, 6H), 3.26 (s, 3H). ^{13}C NMR (126 MHz, DMSO-D6) δ 179.40, 164.99, 164.99, 141.09, 134.76, 132.24, 132.24, 131.01, 129.80, 129.54, 129.50, 129.34, 128.37, 125.10, 116.90, 52.84, 52.77, 51.05, 17.55. HRMS $[M]^+$ m/z calcd. for $[C_{19}H_{18}NO_4S]^+$ 356.0951, found 356.0948.

3-(5-carboxypentyl)-2-methylbenzothiazol-3-ium bromide **2j**: A mixture of 597 mg (4 mmol) of 2-methylbenzothiazole and 390 mg (2 mmol) of 6-bromohexanoic acid was heated in 150 °C to yield 220 mg of **2j** as a white solid in a 32% yield. ^{1}H NMR (500 MHz, CD_3OD) δ 8.30 (d, J = 8.1 Hz, 1H), 8.26 (d, J = 8.5 Hz, 1H), 7.93 (t, J = 7.4 Hz, 1H), 7.82 (t, J = 7.7 Hz, 1H), 4.82 – 4.73 (m, 2H), 3.23 (s, 3H), 2.35 (t, J = 7.2 Hz, 2H), 2.00 (p, J = 7.9 Hz, 2H), 1.71 (p, J = 7.2 Hz, 2H), 1.57 (p, J = 7.6 Hz, 2H).^{13}C NMR (126 MHz, CD_3OD) δ 177.70, 177.57, 142.43, 131.05, 130.55, 129.79, 125.23, 117.80, 50.74, 34.47, 28.98, 26.92, 25.35, 17.20. HRMS $[M]^+$ m/z calcd. for $[C_{14}H_{18}NO_2S]^+$ 264.1053, found 264.1054.

4. General procedure for synthesis of 1-alkyl-4-methylthioquinolinium halide **3a-c**

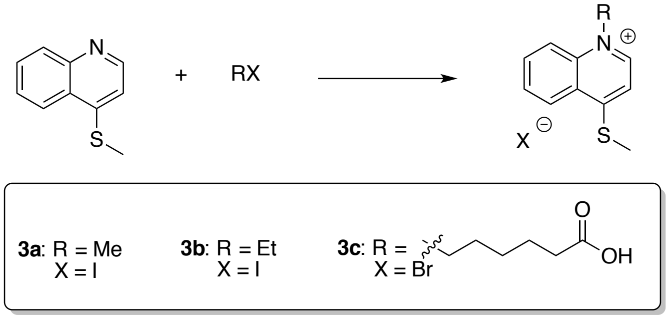

Scheme S2. Synthesis of 1-alkyl-4-methylthioquinolinium halide **3a-c.**

The synthesis of **3a-c** was adopted from previously published protocol.[4] 4-(methylthio)quinoline[5] and appropriate alkyl halide was dissolved in ACN or 1,4-dioxane. The mixture was stirred and heated overnight. The resulting solid was filtered and washed with diethylether. The solid was used directly without further purification.

1-methyl-4-(methylthio)quinolin-1-ium iodide **3a**: A mixture of 87.5 mg (0.5 mmol) 4-(methylthio)quinoline and 355 mg (2.5 mmol) of methyl iodide was heated at 50 °C in 5 mL ACN to yield 150 mg **3a** as a bright yellow powder in a 95% yield. ^{1}H NMR (500 MHz, CD_2Cl_2) δ 9.88 (d, J = 6.6 Hz, 1H), 8.44 (d, J = 8.4 Hz, 1H), 8.19 – 8.14 (m, 2H), 7.92 (ddd, J = 8.3, 4.5, 3.6 Hz, 1H), 7.85 (d, J = 6.6 Hz, 1H), 4.65 (s, 3H), 2.88 (s, 3H). ^{13}C NMR (126 MHz, CD_2Cl_2) δ 165.89, 147.65, 136.17, 136.17, 130.10, 126.95, 126.04, 119.08, 115.93, 45.27, 15.79. HRMS $[M]^+$ m/z calcd. for $[C_{11}H_{12}NS]^+$ 190.0685, found 190.0682.

1-ethyl-4-(methylthio)quinolin-1-ium iodide **3b**: A mixture of 87.5 mg (0.5 mmol) 4-(methylthio)quinoline and 390 mg (2.5 mmol) of ethyl iodide was heated at 100 °C in 5 mL 1,4-dioxane to yield 247 mg **3b** as a bright yellow powder in a 87 % yield. ^{1}H NMR (500 MHz, CD_2Cl_2) δ 9.89 (d, J = 6.6 Hz, 1H), 8.43 (d, J = 8.4 Hz, 1H), 8.22 (d, J = 8.9 Hz, 1H), 8.16 (ddd, J = 8.8, 6.9, 1.4 Hz, 1H), 7.96 – 7.83 (m, 2H), 5.12 (q, J = 7.3 Hz, 2H), 2.88 (s, 3H), 1.74 (t, J = 7.3 Hz, 3H). ^{13}C NMR (126 MHz, CD_2Cl_2) δ 165.56, 146.61, 136.07, 135.93, 129.91, 127.28, 126.24, 119.10, 116.34, 52.91, 15.88, 15.85. HRMS $[M]^+$ m/z calcd. for $[C_{12}H_{14}NS]^+$ 204.0841, found 204.0841.

1-(5-carboxypentyl)-4-(methylthio)quinolin-1-ium bromide **3c**: In a 250 mL round bottom flask, a mixture of 1.75 g (100 mmol) 4-(methylthio)quinoline and 1.95 g (100 mmol) of 6-bromohexanoic acid was heated at 100 °C in 100 mL 1,4-dioxane overnight to yield 3.51 g **3c** as a off-white powder in a 95% yield. ^{1}H NMR (500 MHz, CD_3OD) δ

9.05 (d, *J* = 6.6 Hz, 1H), 8.53 (dd, *J* = 8.5, 1.3 Hz, 1H), 8.45 (d, *J* = 9.0 Hz, 1H), 8.23 (ddd, *J* = 8.8, 7.0, 1.4 Hz, 1H), 7.98 (ddd, *J* = 8.2, 7.0, 1.0 Hz, 1H), 7.83 (d, *J* = 6.6 Hz, 1H), 4.93 (t, 2H), 2.91 (s, 3H), 2.33 (t, *J* = 7.2 Hz, 2H), 2.08 (p, *J* = 7.7 Hz, 2H), 1.69 (dt, *J* = 15.0, 7.3 Hz, 2H), 1.52 (p, *J* = 7.7 Hz, 2H). ^{13}C NMR (126 MHz, CD_3OD) δ 177.17, 166.95, 147.02, 137.10, 136.76, 130.63, 128.08, 126.53, 120.41, 116.19, 57.90, 34.46, 30.51, 26.91, 25.34, 14.95. HRMS $[M]^+$ m/z calcd. for $[C_{16}H_{20}NO_2S]^+$ 290.1209, found 290.1209.

5. General procedure for synthesis of alkylated thiazole orange carboxylic acids **4a-k**

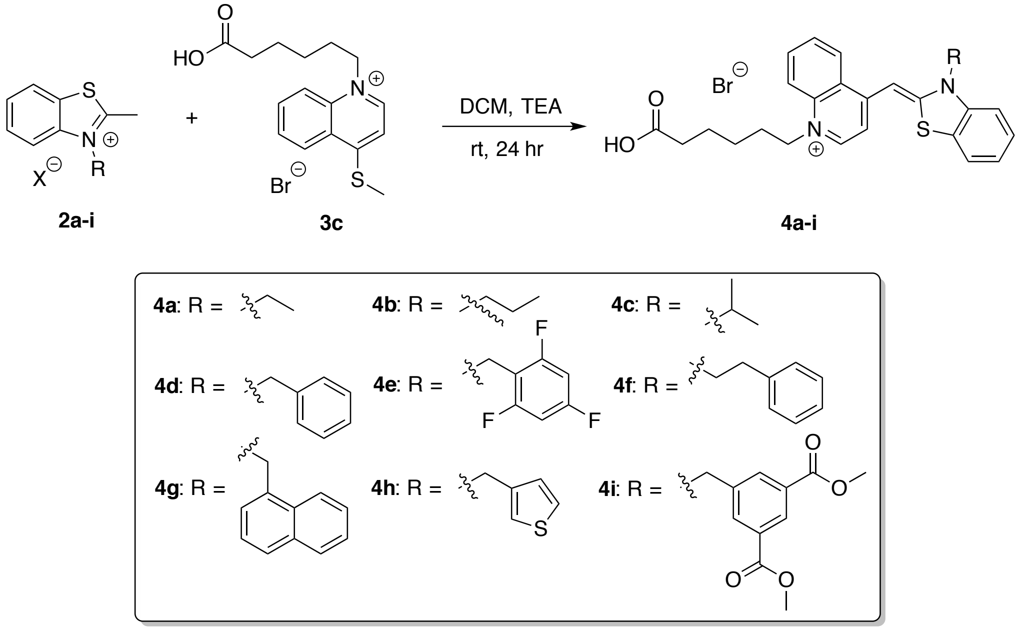

Scheme S3. Synthesis of alkylated thiazole orange carboxylic acids **4a-i**.

The synthesis of **4a-i** was adopted from previously published protocol.[6,7] In a 4 mL glass vial, **3c** and appropriate alkylated benzothiazolium derivative **2a-i** were suspended in DCM. Triethylamine was added and the mixture turned red. The mixture was allowed to stir at room temperature for 48 h. The solid was filtered and washed with acetone to yield analytical pure TO carboxylic acid derivative **4a-i**.

(*Z*)-1-(5-carboxypentyl)-4-((3-ethylbenzo[*d*]thiazol-2(3*H*)-ylidene)methyl)quinolin-1-ium bromide **4a**: A mixture of 200 mg (0.54 mmol) of **3c**, 200 mg (0.66 mmol) of **2a** and 362 µL (2.6 mmol) triethylamine were stirred in 500 µL DCM to yield 105 mg of **4a** as a red solid in a 39% yield. ^{1}H NMR (500 MHz, CD_3OD) δ 8.65 (d, *J* = 7.8 Hz, 1H), 8.45 (d, *J* = 7.2 Hz, 1H), 8.09 (d, *J* = 8.7 Hz, 1H), 7.98 (ddd, *J* = 8.6, 6.9, 1.3 Hz, 1H), 7.92 (d, *J* = 7.2 Hz, 1H), 7.76 (ddd, *J* = 8.3, 6.9, 1.1 Hz, 1H), 7.69 (d, *J* = 8.1 Hz, 1H), 7.62 (ddd, *J* = 8.4, 7.2, 1.2 Hz, 1H), 7.51 (d, *J* = 7.2 Hz, 1H), 7.43 (ddd, *J* = 8.2, 7.3, 1.0 Hz, 1H), 6.95 (s, 1H), 4.62 (dt, *J* = 16.8, 7.3 Hz, 4H), 2.26 (t, *J* = 7.2 Hz, 2H), 1.99 (p, *J* = 7.6 Hz, 2H), 1.69 (p, *J* = 7.3 Hz, 2H), 1.52 (t, *J* = 7.2 Hz, 3H), 1.47 (q, *J* = 7.4 Hz, 2H).^{13}C NMR (126 MHz, CD_3OD) δ 179.79, 161.19, 151.09, 145.03, 141.11, 138.81, 134.47, 129.55, 128.18, 126.58, 126.19, 125.94, 125.94, 123.82, 119.04, 113.60, 109.71, 88.56, 55.89, 42.57, 36.62, 30.06, 27.25, 26.19, 12.53. HRMS $[M]^+$ m/z calcd. for $[C_{25}H_{27}N_2O_2S]^+$ 419.1788, found 419.1789.

(*Z*)-1-(5-carboxypentyl)-4-((3-propylbenzo[*d*]thiazol-2(3*H*)-ylidene)methyl)quinolin-1-ium bromide **4b**: A mixture of 40 mg (0.11 mmol) of **3c**, 35 mg (0.11 mmol) of **2b** and 77 µL (0.55 mmol) triethylamine were stirred in 200 µL DCM to yield 24.2 mg of **4b** as a red solid in a 43% yield. ^{1}H NMR (500 MHz, CD_3OD) δ 8.56 (d, *J* = 8.4 Hz, 1H), 8.41 (d, *J* = 6.9 Hz, 1H), 8.05 (d, *J* = 8.7 Hz, 1H), 7.98 (d, *J* = 7.9 Hz, 1H), 7.88 (d, *J* = 7.9 Hz, 1H), 7.77 (d, *J* = 7.7 Hz, 1H), 7.65 (d, *J* = 8.2 Hz, 1H), 7.60 (t, *J* = 7.7 Hz, 1H), 7.46 (s, 1H), 7.42 (t, *J* = 7.5 Hz, 1H), 6.89 (s, 1H), 4.57 (s, 2H), 4.51 (s, 2H), 2.29 (t, *J* = 7.0 Hz, 2H), 1.98 (q, *J* = 7.3 Hz, 4H), 1.70 (p, *J* = 7.6 Hz, 2H), 1.49 (p, *J* = 7.6 Hz, 2H), 1.13 (t, *J* = 7.2 Hz, 3H). ^{13}C NMR (126 MHz, CD_3OD) δ 178.09, 160.12, 149.44, 143.64, 140.16, 137.36, 133.06,

127.99, 126.87, 124.92, 124.71, 124.30, 122.34, 117.59, 112.42, 108.39, 104.99, 87.59, 54.47, 47.35, 34.88, 28.63, 25.80, 24.55, 20.41, 10.15. HRMS $[M]^+$ m/z calcd. for $[C_{26}H_{29}N_2O_2S]^+$ 433.1944, found 433.1946.

(*Z*)-1-(5-carboxypentyl)-4-((3-isopropylbenzo[*d*]thiazol-2(3*H*)-ylidene)methyl)quinolin-1-ium bromide **4c**: A mixture of 40 mg (0.11 mmol) of **3c**, 35 mg (0.11 mmol) of **2c** and 77 µL (0.55 mmol) triethylamine were stirred in 200 µL DCM to yield 15.2 mg of **4c** as a red solid in a 27% yield. ^{1}H NMR (500 MHz, CD_3OD) δ 8.53 (d, 1H), 8.40 (d, *J* = 7.2 Hz, 1H), 8.05 (d, *J* = 8.7 Hz, 1H), 7.96 (t, *J* = 7.8 Hz, 1H), 7.88 – 7.80 (m, 2H), 7.74 (t, *J* = 7.7 Hz, 1H), 7.56 (t, *J* = 7.4 Hz, 1H), 7.52 (d, *J* = 7.1 Hz, 1H), 7.38 (t, *J* = 7.6 Hz, 1H), 6.98 (s, 1H), 5.44 (p, *J* = 7.0 Hz, 1H), 4.57 (t, *J* = 7.4 Hz, 2H), 2.32 (t, *J* = 6.9 Hz, 2H), 1.98 (p, *J* = 7.8 Hz, 2H), 1.82 (s, 3H), 1.81 (s, 3H), 1.69 (p, *J* = 7.2 Hz, 2H), 1.49 (p, *J* = 8.0, 7.3 Hz, 2H). ^{13}C NMR (126 MHz, CD_3OD) δ 177.76, 161.92, 150.90, 145.09, 140.64, 138.77, 134.42, 128.89, 128.15, 126.45, 126.19, 126.15, 125.60, 123.88, 118.97, 115.49, 109.71, 90.07, 55.81, 53.01, 35.05, 30.01, 27.08, 25.61, 19.82, 19.73. HRMS $[M]^+$ m/z calcd. for $[C_{26}H_{29}N_2O_2S]^+$ 433.1944, found 433.1945.

(*Z*)-4-((3-benzylbenzo[*d*]thiazol-2(3*H*)-ylidene)methyl)-1-(5-carboxypentyl)quinolin-1-ium bromide **4d**: A mixture of 80 mg (0.22 mmol) of **3c**, 80 mg (0.25 mmol) of **2d** and 154 µL (1.1 mmol) triethylamine were stirred in 300 µL DCM to yield 13.6 mg of **4d** as a red solid in a 11% yield. ^{1}H NMR (500 MHz, CD_3OD) δ 8.45 (d, 1H), 8.23 (d, *J* = 8.6 Hz, 1H), 8.04 (d, *J* = 8.8 Hz, 1H), 7.93 (t, *J* = 7.8 Hz, 2H), 7.66 (t, 2H), 7.57 (t, *J* = 7.8 Hz, 1H), 7.47 (d, *J* = 7.1 Hz, 1H), 7.45 – 7.36 (m, 3H), 7.35 – 7.29 (m, 3H), 6.84 (s, 1H), 5.80 (s, 2H), 4.57 (t, *J* = 7.4 Hz, 2H), 2.31 (t, *J* = 7.2 Hz, 2H), 1.95 (p, *J* = 7.5 Hz, 2H), 1.67 (p, *J* = 7.3 Hz, 2H), 1.46 (p, *J* = 7.3 Hz, 2H). ^{13}C NMR (126 MHz, CD_3OD) δ 177.34, 161.40, 150.92, 145.24, 141.93, 138.70, 135.88, 134.55, 130.37, 130.37, 129.63, 129.36, 128.26, 127.95, 127.64, 126.25, 126.17, 126.03, 125.62, 123.90, 119.06, 113.78, 110.08, 89.81, 55.99, 50.54, 34.62, 30.07, 27.04, 25.47. HRMS $[M]^+$ m/z calcd. for $[C_{26}H_{29}N_2O_2S]^+$ 481.1944, found 481.1948.

(*Z*)-1-(5-carboxypentyl)-4-((3-(2,4,6-trifluorobenzyl)benzo[*d*]thiazol-2(3*H*)-ylidene)methyl)quinolin-1-ium bromide **4e**: A mixture of 40 mg (0.11 mmol) of **3c**, 40 mg (0.11 mmol) of **2e** and 77 µL (0.55 mmol) triethylamine were stirred in 200 µL DCM to yield 63 mg of **4e** as a red solid in a 95% yield. ^{1}H NMR (500 MHz, CD_3OD) δ 8.50 – 8.46 (m, 2H), 8.10 (d, *J* = 8.8 Hz, 1H), 8.00 (t, 1H), 7.87 (d, *J* = 7.9 Hz, 1H), 7.81 – 7.72 (m, 2H), 7.59 (t, *J* = 7.9 Hz, 1H), 7.50 (d, *J* = 7.0 Hz, 1H), 7.40 (t, *J* = 7.6 Hz, 1H), 7.01 (t, *J* = 8.8 Hz, 2H), 6.89 (s, 1H), 5.82 (s, 2H), 4.60 (t, *J* = 7.3 Hz, 2H), 2.28 (t, *J* = 7.1 Hz, 2H), 1.97 (p, *J* = 7.6 Hz, 2H), 1.67 (p, *J* = 7.3 Hz, 2H), 1.46 (p, *J* = 7.9 Hz, 2H). ^{13}C NMR (126 MHz, CD_3OD) δ 178.83, 165.54, 163.98, 162.00, 161.07, 151.14, 145.50, 141.59, 138.79, 134.70, 134.70, 129.25, 128.42, 126.33, 126.01, 126.01, 125.09, 123.72, 119.28, 113.95, 110.37, 108.40, 102.11, 89.38, 56.14, 39.85, 35.84, 30.09, 27.14, 25.88. HRMS $[M]^+$ m/z calcd. for $[C_{30}H_{26}F_3N_2O_2S]^+$ 535.1662, found 535.1665.

(*Z*)-1-(5-carboxypentyl)-4-((3-phenethylbenzo[*d*]thiazol-2(3*H*)-ylidene)methyl)quinolin-1-ium bromide **4f**: A mixture of 40 mg (0.11 mmol) of **3c**, 40 mg (0.09 mmol) of **2f** and 77 µL (0.55 mmol) triethylamine were stirred in 200 µL DCM to yield 12.1 mg of **4f** as a red solid in a 23% yield. ^{1}H NMR (500 MHz, CD_3OD) δ 8.35 (d, *J* = 7.2 Hz, 1H), 8.27 (d, *J* = 8.4 Hz, 1H), 8.00 (d, *J* = 8.4 Hz, 1H), 7.95 (t, *J* = 7.9 Hz, 1H), 7.86 (d, *J* = 7.8 Hz, 1H), 7.78 – 7.69 (m, 1H), 7.52 (s, 2H), 7.42 – 7.30 (m, 2H), 7.22 – 7.10 (m, 4H), 7.07 – 6.98 (m, 1H), 6.56 (s, 1H), 4.80 (t, *J* = 6.3 Hz, 2H), 4.53 (t, *J* = 7.3 Hz, 2H), 3.24 (t, *J* = 6.3 Hz, 2H), 2.32 (t, *J* = 7.2 Hz, 2H), 2.04 – 1.90 (m, 2H), 1.68 (p, *J* = 15.3, 7.2 Hz, 2H), 1.54 – 1.44 (m, 2H). ^{13}C NMR (126 MHz, CD_3OD) δ 177.47, 161.75, 150.47, 144.76, 141.28, 138.88, 138.61, 134.38, 130.19, 130.19, 129.84, 129.84, 129.34, 128.11, 127.95, 126.81, 125.88, 125.59, 123.64, 123.64, 118.82, 114.03, 109.40, 89.61, 55.75, 49.28, 34.74, 34.53, 30.03, 27.07, 25.52. HRMS $[M]^+$ m/z calcd. for $[C_{31}H_{31}N_2O_2S]^+$ 495.2101, found 495.2098.

(*Z*)-1-(5-carboxypentyl)-4-((3-(naphthalen-1-ylmethyl)benzo[*d*]thiazol-2(3*H*)-ylidene)methyl)quinolin-1-ium bromide **4g**: A mixture of 80 mg (0.22 mmol) of **3c**, 80 mg (0.22 mmol) of **2g** and 154 µL (1.1 mmol) triethylamine were stirred in 300 µL DCM to yield 17.5 mg of **4g** as a red solid in a 13% yield. ^{1}H NMR (500 MHz, CD_3OD) δ 8.53 – 8.43 (m, 2H), 8.03 (dd, *J* = 19.1, 10.3 Hz, 3H), 7.96 – 7.68 (m, 5H), 7.67 – 7.52 (m, 3H), 7.47 (s, 1H), 7.43 – 7.32 (m, 2H), 6.91 (d, *J* = 7.0 Hz, 1H), 6.71 (s, 1H), 6.30 (s, 2H), 4.66 – 4.51 (m, 2H), 2.26 (s, 2H), 1.95 (s, 2H), 1.76 – 1.61 (m, 2H), 1.55 – 1.42 (m, 2H). ^{13}C NMR (126 MHz, CD_3OD) δ 179.19, 161.01, 150.25, 144.93, 141.69, 138.29, 135.33, 134.44, 131.85, 130.48, 130.48, 129.80, 129.49, 128.41, 128.13, 127.78, 126.70, 126.35, 125.84, 125.66, 125.51, 123.89, 123.74, 123.60, 118.90, 113.40, 109.84, 89.36, 55.91, 49.87, 36.25, 29.99, 27.09, 25.97. HRMS $[M]^+$ m/z calcd. for $[C_{34}H_{31}N_2O_2S]^+$ 531.2101, found 531.2099.

(*Z*)-1-(5-carboxypentyl)-4-((3-(thiophen-3-ylmethyl)benzo[*d*]thiazol-2(3*H*)-ylidene)methyl)quinolin-1-ium bromide **4h**: A mixture of 80 mg (0.22 mmol) of **3c**, 80 mg (0.25 mmol) of **2h** and 154 µL (1.1 mmol) triethylamine were

stirred in 300 μL DCM to yield 102.5 mg of **4h** as a red solid in a 84% yield. ^{1}H NMR (500 MHz, DMSO-D6) δ 8.68 (t, *J* = 8.4 Hz, 2H), 8.17 (d, *J* = 8.7 Hz, 1H), 8.08 (d, *J* = 7.9 Hz, 1H), 8.00 (t, *J* = 7.9 Hz, 1H), 7.89 (d, *J* = 8.3 Hz, 1H), 7.76 (d, *J* = 7.9 Hz, 1H), 7.64 – 7.59 (m, 2H), 7.56 – 7.55 (m, 1H), 7.49 – 7.40 (m, 2H), 7.16 – 7.01 (m, 2H), 5.92 (s, 2H), 4.61 (t, *J* = 7.5 Hz, 2H), 2.21 (t, *J* = 7.1 Hz, 2H), 1.84 (p, *J* = 14.1, 7.2 Hz, 2H), 1.54 (p, *J* = 15.1, 7.2 Hz, 2H), 1.47 – 1.32 (m, 2H). ^{13}C NMR (126 MHz, DMSO-D6) δ 174.43, 159.28, 148.66, 144.60, 140.00, 136.97, 135.53, 133.37, 128.31, 127.77, 127.05, 126.78, 125.67, 124.62, 124.35, 123.89, 123.87, 123.09, 118.27, 113.06, 108.33, 88.21, 54.13, 44.73, 33.53, 28.68, 25.47, 24.05. HRMS $[M]^+$ m/z calcd. for $[C_{28}H_{27}N_2O_2S_2]^+$ 487.1508, found 487.1506.

(*Z*)-4-((3-(3,5-bis(methoxycarbonyl)benzyl)benzo[*d*]thiazol-2(3*H*)-ylidene)methyl)-1-(5-carboxypentyl)quinolin-1-ium bromide **4i**: A mixture of 200 mg (0.54 mmol) of **3c**, 235 mg (0.54 mmol) of **2i** and 208 μL (2.7 mmol) triethylamine were stirred in 500 μL DCM to yield 131.5 mg of **4i** as a red solid in a 36% yield. ^{1}H NMR (500 MHz, CD_3OD) δ 8.56 (s, 1H), 8.53 (d, *J* = 7.2 Hz, 1H), 8.36 (d, *J* = 8.5 Hz, 1H), 8.24 (s, 2H), 8.10 (d, *J* = 8.8 Hz, 1H), 7.97 (t, *J* = 7.5 Hz, 2H), 7.74 – 7.65 (m, 2H), 7.61 (t, *J* = 7.7 Hz, 1H), 7.56 (d, *J* = 6.9 Hz, 1H), 7.45 (t, *J* = 7.6 Hz, 1H), 6.90 (s, 1H), 5.96 (s, 2H), 4.63 (t, *J* = 7.4 Hz, 2H), 3.90 (s, 6H), 2.27 (t, *J* = 7.2 Hz, 2H), 1.98 (p, *J* = 7.6 Hz, 2H), 1.67 (dt, *J* = 15.1, 7.3 Hz, 2H), 1.46 (dt, *J* = 15.0, 7.8 Hz, 2H). ^{13}C NMR (126 MHz, CD_3OD) δ 178.44, 166.85, 166.85, 160.95, 150.88, 145.45, 141.55, 138.62, 137.75, 134.67, 133.06, 133.06, 132.92, 132.92, 131.07, 131.02, 129.72, 128.46, 126.43, 126.18, 125.46, 124.02, 119.16, 113.49, 110.39, 89.46, 56.18, 53.23, 53.15, 49.66, 35.51, 30.11, 27.12, 25.79. HRMS $[M]^+$ m/z calcd. for $[C_{34}H_{33}N_2O_6S]^+$ 597.2054, found 597.2051.

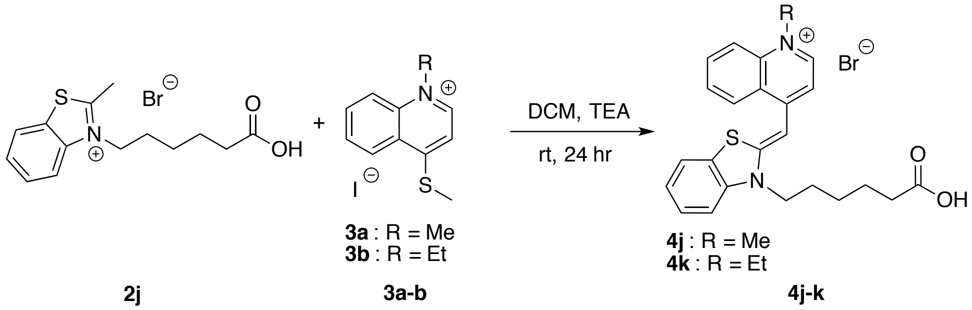

Scheme S4. Synthesis of alkylated thiazole orange carboxylic acids **4j-k**.

(*Z*)-4-((3-(5-carboxypentyl)benzo[*d*]thiazol-2(3*H*)-ylidene)methyl)-1-methylquinolin-1-ium bromide **4j**: A mixture of 37 mg (0.12 mmol) of **3a**, 40 mg (0.12 mmol) of **2j** and 77 μL (0.55 mmol) triethylamine were stirred in 200 μL DCM to yield 24.2 mg of **4j** as a red solid in a 43% yield. ^{1}H NMR (500 MHz, CD_3OD) δ 8.60 (d, *J* = 8.5, 1.2 Hz, 1H), 8.39 (d, *J* = 7.2 Hz, 1H), 8.06 – 7.97 (m, 2H), 7.89 (d, *J* = 7.9, 1.1 Hz, 1H), 7.81 (ddd, *J* = 8.3, 6.6, 1.5 Hz, 1H), 7.67 (d, 1H), 7.61 (ddd, *J* = 8.3, 7.2, 1.2 Hz, 1H), 7.49 (d, *J* = 7.1 Hz, 1H), 7.42 (ddd, *J* = 8.2, 7.2, 1.1 Hz, 1H), 6.93 (s, 1H), 4.56 (t, *J* = 7.7 Hz, 2H), 4.19 (s, 3H), 2.31 (t, *J* = 7.2 Hz, 2H), 1.96 (p, *J* = 7.7 Hz, 2H), 1.74 (p, *J* = 7.3 Hz, 2H), 1.65 – 1.55 (m, 2H). ^{13}C NMR (126 MHz, CD_3OD) δ 176.99, 159.94, 149.61, 144.19, 140.08, 138.41, 133.07, 128.11, 127.03, 124.80, 124.45, 124.31, 122.31, 117.52, 112.45, 108.21, 108.18, 87.38, 45.80, 41.67, 34.42, 26.66, 25.98, 24.76. HRMS $[M]^+$ m/z calcd. for $[C_{24}H_{25}N_2O_2S]^+$ 405.1631, found 405.1631.

(*Z*)-4-((3-(5-carboxypentyl)benzo[*d*]thiazol-2(3*H*)-ylidene)methyl)-1-ethylquinolin-1-ium bromide **4k**: A mixture of 39 mg (0.12 mmol) of **3b**, 40 mg (0.12 mmol) of **2j** and 77 μL (0.55 mmol) triethylamine were stirred in 200 μL DCM to yield 45.3 mg of **4k** as a red solid in a 78% yield. ^{1}H NMR (500 MHz, CD_3OD) δ 8.54 (d, *J* = 8.5 Hz, 1H), 8.40 (d, *J* = 7.2 Hz, 1H), 8.05 (d, *J* = 8.7 Hz, 1H), 7.96 (t, *J* = 7.8 Hz, 1H), 7.85 (d, *J* = 7.8 Hz, 1H), 7.76 (t, *J* = 7.7 Hz, 1H), 7.66 – 7.53 (m, 2H), 7.45 (d, *J* = 7.1 Hz, 1H), 7.38 (t, *J* = 7.4 Hz, 1H), 6.85 (s, 1H), 4.61 (q, *J* = 7.2 Hz, 2H), 4.50 (t, *J* = 7.6 Hz, 2H), 2.31 (s, 2H), 1.93 (p, *J* = 5.9, 4.2 Hz, 2H), 1.73 (p, *J* = 7.2 Hz, 2H), 1.58 (t, *J* = 7.0 Hz, 5H). ^{13}C NMR (126 MHz, CD_3OD) δ 177.64, 161.49, 151.06, 144.50, 141.53, 138.68, 134.50, 129.53, 128.32, 126.56, 126.21, 125.95, 125.76, 123.75, 118.86, 113.88, 110.03, 88.90, 51.21, 47.25, 34.95, 28.06, 27.32, 25.87, 15.06. HRMS $[M]^+$ m/z calcd. for $[C_{25}H_{27}N_2O_2S]^+$ 419.1788, found 419.1789.

6. General procedure for synthesis of preQ$_1$-PEG3-TO **5a-f**

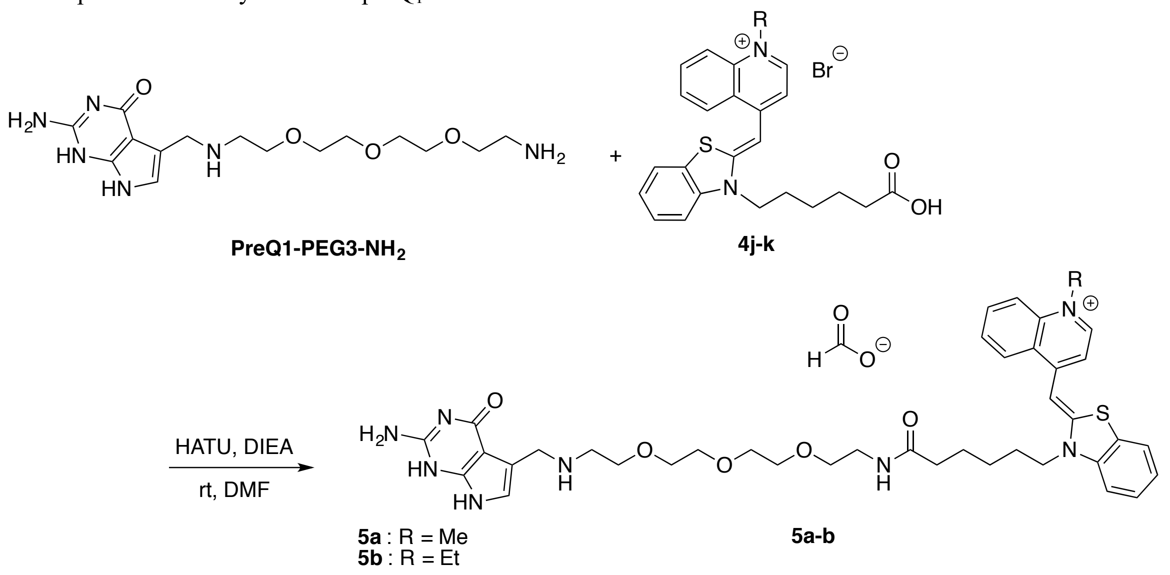

Scheme S5. Synthesis of preQ$_1$-PEG3-TO **5a-b**.

PreQ1-PEG3-TO **5a-f** was prepared following previous protocol.[8] In a 4 mL glass vial, appropriate thiazole orange carboxylic acids **4a-d, 4j-k** was dissolved in DMF anhydrous and the mixture was cooled to 0 °C. HATU and DIEA were added and the mixture was stirred at 0 °C for 10 min. A solution of preQ$_1$-PEG3-NH$_2$[8] was then added to the stirring mixture. The mixture was continued to stir for 1 h and reaction was monitored by LC-MS. Upon the completion of the reaction, the solvent was removed *in vacuo* and the residue was purified by HPLC. HPLC gradient: 0 min – 2.5 min 50% *Phase B* in *Phase A*, 2.5 min – 18 min 50% *Phase B* in *Phase A* to 90% *Phase B* in *Phase A*, 18 min – 28 min 95% *Phase B* in *Phase A*, 28 min – 32 min 50% *Phase B* in *Phase A* (*Phase A*: H_2O with 0.1% formic acid, *Phase B*: MeOH with 0.1% formic acid).

(*Z*)-4-((3-(1-(2-amino-4-oxo-4,7-dihydro-1*H*-pyrrolo[2,3-*d*]pyrimidin-5-yl)-15-oxo-5,8,11-trioxa-2,14-diazaicosan-20-yl)benzo[*d*]thiazol-2(3*H*)-ylidene)methyl)-1-methylquinolin-1-ium **5a**: A mixture of 5 mg (10 μmol) of **4j**, 3.5 (10 μmol) mg of preQ$_1$-PEG3-NH$_2$, 3.8 mg (10.0 μmol) of HATU and 8.8 μL (50.0 μmol) of DIEA were stirred in 200 μL of DMF to yield 2.2 mg of **5a** as a red solid in a 27% yield. ^{1}H NMR (500 MHz, CD$_3$OD) δ 8.58 (d, *J* = 8.6 Hz, 1H), 8.39 (d, *J* = 7.2 Hz, 1H), 8.07 – 7.96 (m, 2H), 7.90 (d, *J* = 7.9 Hz, 1H), 7.82 – 7.75 (m, 1H), 7.66 (d, *J* = 8.1 Hz, 1H), 7.61 (t, *J* = 7.8 Hz, 1H), 7.49 (d, *J* = 7.2 Hz, 1H), 7.46 – 7.38 (m, 1H), 6.91 (s, 1H), 6.81 (s, 1H), 4.59 – 4.54 (m, 2H), 4.24 (s, 2H), 4.20 (s, 3H), 3.78 – 3.69 (m, 2H), 3.63 (d, *J* = 8.3 Hz, 4H), 3.61 (dd, *J* = 4.4, 2.2 Hz, 2H), 3.56 (dt, *J* = 6.8, 3.1 Hz, 2H), 3.47 (t, *J* = 5.7 Hz, 2H), 3.29 (s, 2H), 3.25 – 3.16 (m, 2H), 2.23 (t, *J* = 7.3 Hz, 2H), 1.97 (p, *J* = 8.1 Hz, 2H), 1.74 (p, *J* = 7.2 Hz, 2H), 1.56 (dt, *J* = 15.6, 7.8 Hz, 2H). ^{13}C NMR (126 MHz, CD$_3$OD) δ 174.49, 161.11, 159.99, 153.12, 152.45, 149.66, 144.26, 140.13, 138.44, 133.08, 128.11, 126.96, 124.77, 124.52, 124.47, 124.33, 122.36, 117.81, 117.56, 112.45, 108.35, 108.25, 98.33, 87.41, 70.12, 70.05, 69.99, 69.74, 69.12, 65.66, 45.90, 45.83, 43.34, 41.69, 38.79, 35.18, 26.70, 25.92, 25.12. HRMS $[M]^+$ m/z calcd. for $[C_{39}H_{49}N_8O_5S]^+$ 741.3541, found 741.3543.

(*Z*)-4-((3-(1-(2-amino-4-oxo-4,7-dihydro-1*H*-pyrrolo[2,3-*d*]pyrimidin-5-yl)-15-oxo-5,8,11-trioxa-2,14-diazaicosan-20-yl)benzo[*d*]thiazol-2(3*H*)-ylidene)methyl)-1-ethylquinolin-1-ium **5b**: 5 mg (10 mmol) of **4k**, 3.5 mg (10 μmol) of preQ$_1$-PEG3-NH$_2$, 3.8 mg (10.0 μmol) of HATU and 8.8 μL (50.0 μmol) of DIEA were stirred in 200 μL of DMF to yield 4.2 mg of **5b** as a red solid in a 52% yield. ^{1}H NMR (500 MHz, CD$_3$OD) δ 8.54 (d, *J* = 8.5 Hz, 1H), 8.40 (d, *J* = 7.1 Hz, 1H), 8.04 (d, *J* = 8.6 Hz, 1H), 7.95 (t, *J* = 7.3 Hz, 1H), 7.85 (d, *J* = 7.8 Hz, 1H), 7.75 (t, *J* = 7.7 Hz, 1H), 7.58 (q, *J* = 8.4 Hz, 2H), 7.46 – 7.40 (m, 1H), 7.38 (t, *J* = 7.4 Hz, 1H), 6.84 (d, *J* = 4.6 Hz, 1H), 6.81 (s, 1H), 4.60 (q, *J* = 7.1 Hz, 2H), 4.50 (t, *J* = 7.0 Hz, 2H), 4.24 (s, 2H), 3.73 (t, *J* = 4.9 Hz, 2H), 3.67 – 3.59 (m, 6H), 3.55 (dd, *J* = 5.7, 3.1 Hz, 2H), 3.46 (t, *J* = 5.6 Hz, 2H), 3.29 (d, *J* = 5.6 Hz, 2H), 3.24 – 3.17 (m, 2H), 2.22 (t, *J* = 7.3 Hz, 2H), 1.92 (p, *J* = 7.6 Hz, 2H), 1.72 (p, *J* = 7.4 Hz, 2H), 1.56 (dt, *J* = 17.5, 7.4 Hz, 5H). ^{13}C NMR (126 MHz, CD$_3$OD) δ 174.50, 161.10, 159.84, 153.13, 152.39, 149.30, 143.04, 139.99, 137.12, 133.06, 128.09, 126.87, 125.07, 124.65, 124.50, 124.29, 122.33, 117.86, 117.43, 112.42, 108.53, 108.26, 98.30, 87.39, 70.10, 70.04, 69.96, 69.72, 69.09, 65.61, 49.81,

45.86, 45.79, 43.27, 38.79, 35.17, 26.70, 25.87, 25.14, 13.66. HRMS $[M]^+$ m/z calcd. for $[C_{40}H_{51}N_8O_5S]^+$ 755.3698, found 755.3693.

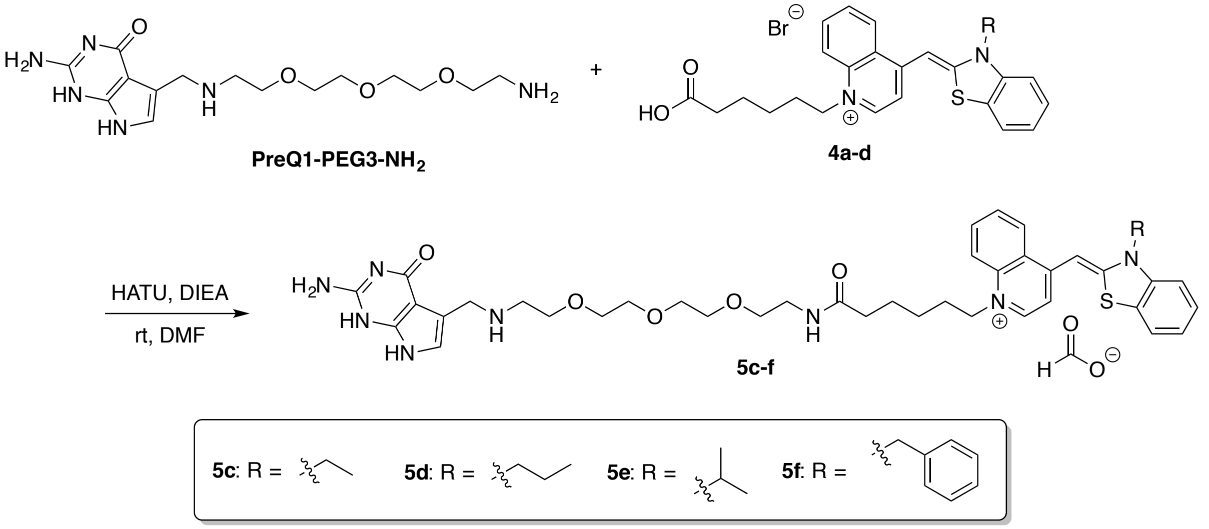

Scheme S6. Synthesis of preQ$_1$-PEG3-TO **5c-f**.

(*Z*)-1-(1-(2-amino-4-oxo-4,7-dihydro-1*H*-pyrrolo[2,3-*d*]pyrimidin-5-yl)-15-oxo-5,8,11-trioxa-2,14-diazaicosan-20-yl)-4-((3-ethylbenzo[*d*]thiazol-2(3*H*)-ylidene)methyl)quinolin-1-ium formate **5c**: 5 mg (10 µmol) of **4a**, 3.5 mg (10 µmol) of preQ$_1$-PEG3-NH$_2$, 3.8 mg (10.0 µmol) of HATU and 8.8 µL (50 µmol) of DIEA were stirred in 200 µL of DMF to yield 3.2 mg of **5c** in a red solid in a 41% yield. ^{1}H NMR (500 MHz, CD$_3$OD)δ 8.63 (d, *J* = 8.0 Hz, 1H), 8.42 (d, *J* = 7.1 Hz, 1H), 8.06 (d, *J* = 8.6 Hz, 1H), 7.97 (t, *J* = 7.3 Hz, 1H), 7.91 (d, *J* = 7.9 Hz, 1H), 7.76 (t, *J* = 7.6 Hz, 1H), 7.68 (d, *J* = 7.9 Hz, 1H), 7.62 (t, *J* = 7.8 Hz, 1H), 7.48 (d, *J* = 4.9 Hz, 1H), 7.43 (t, *J* = 7.1 Hz, 1H), 6.92 (s, 1H), 6.82 (s, 1H), 4.60 (dt, *J* = 14.7, 5.2 Hz, 4H), 4.26 (s, 2H), 3.74 (t, *J* = 4.9 Hz, 2H), 3.65 (s, 4H), 3.62 (dd, *J* = 6.1, 3.3 Hz, 2H), 3.57 (dd, *J* = 6.1, 3.3 Hz, 2H), 3.49 (t, *J* = 5.6 Hz, 2H), 3.35 – 3.32 (m, 2H), 3.22 (t, *J* = 4.9 Hz, 2H), 2.22 (t, *J* = 7.3 Hz, 2H), 1.97 (p, *J* = 7.6 Hz, 2H), 1.71 – 1.65 (m, 2H), 1.52 (tt, *J* = 7.2, 1.7 Hz, 3H), 1.47 – 1.41 (m, 2H). ^{13}C NMR (126 MHz, CD$_3$OD) δ 174.49, 161.17, 159.78, 153.17, 152.45, 149.57, 143.55, 139.66, 137.35, 133.06, 128.19, 126.78, 125.17, 124.73, 124.58, 124.50, 122.40, 117.85, 117.55, 112.24, 108.29, 108.25, 98.33, 87.18, 70.13, 70.06, 69.98, 69.74, 69.12, 65.64, 54.39, 45.89, 43.30, 41.19, 38.80, 35.14, 28.59, 25.63, 24.86, 11.14. HRMS $[M]^+$ m/z calcd. for $[C_{40}H_{51}N_8O_5S]^+$ 755.3698, found 755.3694.

(*Z*)-1-(1-(2-amino-4-oxo-4,7-dihydro-1*H*-pyrrolo[2,3-*d*]pyrimidin-5-yl)-15-oxo-5,8,11-trioxa-2,14-diazaicosan-20-yl)-4-((3-propylbenzo[*d*]thiazol-2(3*H*)-ylidene)methyl)quinolin-1-ium formate **5d**: 4.9 mg (9.6 µmol) of **4b**, 3.4 mg (9.6 µmol) of preQ$_1$-PEG3-NH$_2$, 3.8 mg (10.0 µmol) of HATU and 8.8 µL (50 µmol) of DIEA were stirred in 200 µL of DMF to yield 3.9 mg of **5d** as a red solid in a 50% yield. ^{1}H NMR (500 MHz, CD$_3$OD) δ 8.57 (d, *J* = 8.5 Hz, 1H), 8.41 (d, *J* = 7.3 Hz, 1H), 8.04 (d, *J* = 8.7 Hz, 1H), 7.97 (t, *J* = 7.8 Hz, 1H), 7.89 (d, *J* = 7.8 Hz, 1H), 7.75 (t, *J* = 7.7 Hz, 1H), 7.66 (d, *J* = 8.3 Hz, 1H), 7.60 (t, *J* = 7.4 Hz, 1H), 7.46 (d, *J* = 7.2 Hz, 1H), 7.41 (t, *J* = 7.6 Hz, 1H), 6.90 (s, 1H), 6.82 (s, 1H), 4.55 (dt, *J* = 22.3, 7.5 Hz, 4H), 4.26 (s, 2H), 3.77 – 3.71 (m, 2H), 3.66 – 3.60 (m, 6H), 3.59 – 3.55 (m, 2H), 3.49 (t, *J* = 5.6 Hz, 2H), 3.36 – 3.32 (m, 2H), 3.26 – 3.17 (m, 2H), 2.22 (t, *J* = 7.3 Hz, 2H), 1.97 (h, *J* = 7.4 Hz, 4H), 1.68 (p, *J* = 7.4 Hz, 2H), 1.47 – 1.39 (m, 2H), 1.12 (t, *J* = 7.4 Hz, 3H). ^{13}C NMR (126 MHz, CD$_3$OD) δ 174.50, 161.14, 160.12, 153.18, 152.43, 149.39, 143.56, 140.16, 137.32, 133.06, 128.11, 126.82, 125.05, 124.69, 124.55, 124.30, 122.34, 117.85, 117.57, 112.56, 108.31, 108.25, 98.33, 87.54, 70.11, 70.05, 69.97, 69.74, 69.12, 65.66, 54.40, 47.30, 45.89, 43.30, 38.81, 35.15, 28.59, 25.62, 24.87, 20.43, 10.11. HRMS $[M]^+$ m/z calcd. for $[C_{41}H_{53}N_8O_5S]^+$ 769.3854, found 769.3867.

(*Z*)-1-(1-(2-amino-4-oxo-4,7-dihydro-1*H*-pyrrolo[2,3-*d*]pyrimidin-5-yl)-15-oxo-5,8,11-trioxa-2,14-diazaicosan-20-yl)-4-((3-isopropylbenzo[*d*]thiazol-2(3*H*)-ylidene)methyl)quinolin-1-ium formate **5e**: 5 mg of **4c** (10 µmol), 3.5 mg (10.0 µmol) of preQ$_1$-PEG3-NH$_2$, 3.8 mg (10.0 µmol) of HATU and 8.8 µL (50 µmol) of DIEA were stirred in 200 µL of DMF to yield 1.2 mg of **5e** as a red solid in a 24% yield. ^{1}H NMR (500 MHz, CD$_3$OD) δ 8.56 (s, 1H), 8.40 (d, *J* = 7.1 Hz, 1H), 8.05 (d, *J* = 8.7 Hz, 1H), 7.97 (t, *J* = 7.8 Hz, 1H), 7.87 (dd, *J* = 17.1, 8.2 Hz, 2H), 7.75 (t, *J* = 7.6 Hz,

1H), 7.58 (t, *J* = 7.9 Hz, 1H), 7.54 (d, *J* = 7.0 Hz, 1H), 7.40 (t, *J* = 7.6 Hz, 1H), 7.00 (s, 1H), 6.83 (s, 1H), 5.45 (hept, *J* = 7.9, 7.3 Hz, 1H), 4.58 (t, *J* = 7.2 Hz, 2H), 4.27 (s, 2H), 3.78 – 3.72 (m, 2H), 3.66 – 3.60 (m, 6H), 3.59 – 3.56 (m, 2H), 3.49 (t, *J* = 5.5 Hz, 2H), 3.36 – 3.34 (m, 2H), 3.25 – 3.20 (m, 2H), 2.22 (t, *J* = 7.3 Hz, 2H), 1.98 (p, *J* = 8.0 Hz, 2H), 1.82 (d, *J* = 7.0 Hz, 6H), 1.67 (q, *J* = 7.5 Hz, 2H), 1.49 – 1.42 (m, 2H). ^{13}C NMR (126 MHz, CD_3OD) δ 174.49, 161.17, 160.56, 153.17, 152.48, 149.53, 143.68, 143.52, 139.29, 137.39, 133.03, 127.51, 126.86, 125.10, 124.81, 124.23, 122.49, 117.92, 117.58, 114.08, 108.35, 108.11, 98.37, 88.69, 70.13, 70.06, 70.00, 69.76, 69.14, 65.67, 54.39, 51.69, 45.94, 43.37, 38.83, 35.17, 28.59, 25.64, 24.85, 18.42, 18.32. HRMS $[M]^+$ m/z calcd. for $[C_{41}H_{53}N_8O_5S]^+$ 769.3854, found 769.3854.

(*Z*)-1-(1-(2-amino-4-oxo-4,7-dihydro-1*H*-pyrrolo[2,3-*d*]pyrimidin-5-yl)-15-oxo-5,8,11-trioxa-2,14-diazaicosan-20-yl)-4-((3-benzylbenzo[*d*]thiazol-2(3*H*)-ylidene)methyl)quinolin-1-ium formate **5f**: 4.9 mg (10 µmol) of **4d**, 3.5 mg (10 µmol) of preQ$_1$-PEG3-NH$_2$, 3.8 mg (10 µmol) of HATU and 8.8 µL (50 µmol) of DIEA were stirred in 200 µL of DMF to yield 3.3 mg of **5f** as a red solid in a 41% yield. ^{1}H NMR (500 MHz, CD_3OD) δ 8.46 (d, *J* = 6.1 Hz, 1H), 8.22 (d, *J* = 8.4 Hz, 1H), 8.05 (d, *J* = 8.7 Hz, 1H), 7.94 (t, *J* = 7.6 Hz, 2H), 7.67 (q, *J* = 7.9 Hz, 2H), 7.59 (t, *J* = 7.7 Hz, 1H), 7.49 (d, *J* = 6.3 Hz, 1H), 7.42 (dt, *J* = 18.2, 7.2 Hz, 3H), 7.33 (d, *J* = 7.2 Hz, 3H), 6.85 (s, 1H), 6.82 (s, 1H), 5.81 (s, 2H), 4.59 (t, *J* = 6.6 Hz, 2H), 4.26 (s, 2H), 3.73 (s, 2H), 3.62 (d, *J* = 20.8 Hz, 6H), 3.56 (s, 2H), 3.48 (s, 2H), 3.37 – 3.32 (m, 2H), 3.22 (s, 2H), 2.20 (t, *J* = 7.0 Hz, 2H), 2.00 – 1.90 (m, 2H), 1.72 – 1.61 (m, 2H), 1.41 (s, 2H). ^{13}C NMR (126 MHz, CD_3OD) δ 174.49, 161.10, 160.02, 153.35, 152.32, 149.55, 143.92, 140.56, 137.33, 134.48, 133.16, 128.95, 128.27, 127.91, 126.87, 126.82, 126.26, 126.15, 124.80, 124.72, 124.63, 124.21, 122.52, 117.86, 117.68, 112.38, 108.71, 108.62, 108.36, 88.39, 70.12, 70.06, 69.98, 69.74, 69.12, 65.65, 54.56, 49.14, 45.91, 43.34, 38.80, 35.13, 28.60, 25.59, 24.84. HRMS $[M]^+$ m/z calcd. for $[C_{45}H_{53}N_8O_5S]^+$ 817.3854, found 817.3854.

7. General procedure for synthesis of preQ$_1$-C6-TO **6a-f**

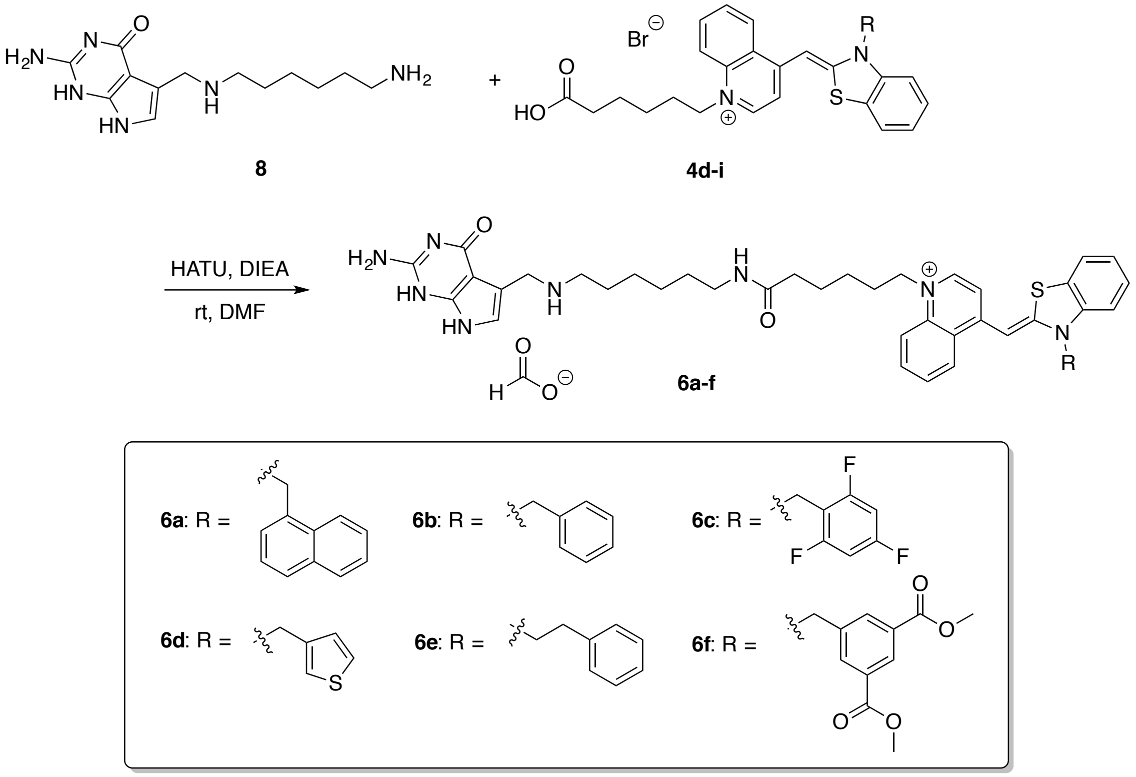

Scheme S7. Synthesis of preQ$_1$-C6-TO **6a-f**.

PreQ$_1$-C6-TO **6a-f** was prepared following previous protocol.[8] In a 4 mL glass vial, appropriate thiazole orange carboxylic acids **4d-i** was dissolved in DMF anhydrous and the mixture was cooled to 0 °C. HATU and DIEA were added and the mixture was stirred at 0 °C for 10 min. A solution of **8** was then added to the stirring mixture. The

mixture was stirred for 1 h and reaction was monitored by LC-MS. Upon the completion of the reaction, the solvent was removed *in vacuo* and the residue was purified by HPLC. HPLC gradient: 0 min – 2.5 min 50% *Phase B* in *Phase A*, 2.5 min – 18 min 50% *Phase B* in *Phase A* to 90% *Phase B* in *Phase A*, 18 min – 28 min 95% *Phase B* in *Phase A*, 28 min – 32 min 50% *Phase B* in *Phase A* (*Phase A*: H_2O with 0.1% formic acid, *Phase B*: MeOH with 0.1% formic acid).

(*Z*)-1-(6-((6-(((2-amino-4-oxo-4,7-dihydro-1*H*-pyrrolo[2,3-*d*]pyrimidin-5-yl)methyl)amino)hexyl)amino)-6-oxohexyl)-4-((3-(naphthalen-1-ylmethyl)benzo[*d*]thiazol-2(3*H*)-ylidene)methyl)quinolin-1-ium formate **6a**: 4.4 mg (7.2 µmol) of **4g**, 2 mg (7.2 µmol) of **8**, 2.7 mg (7.2 µmol) of HATU and 6.3 µL (36 µmol) of DIEA were stirred in 200 µL of DMF yield 1.3 mg of **6a** as a red solid in a 22% yield. ^{1}H NMR (500 MHz, CD_3OD) δ 8.47 (d, *J* = 6.6 Hz, 1H), 8.44 (d, *J* = 6.6 Hz, 1H), 8.06 – 7.99 (m, 2H), 7.91 – 7.68 (m, 5H), 7.57 (s, 2H), 7.53 (d, *J* = 6.9 Hz, 1H), 7.47 (d, *J* = 7.6 Hz, 1H), 7.42 – 7.31 (m, 2H), 6.89 (d, *J* = 6.9 Hz, 1H), 6.82 (s, 1H), 6.70 (s, 1H), 6.29 (s, 2H), 4.64 – 4.53 (m, 2H), 4.20 (s, 2H), 3.19 – 3.07 (m, 3H), 2.99 (s, 2H), 2.28 – 2.15 (m, 2H), 1.96 (s, 2H), 1.66 (s, 4H), 1.50 – 1.37 (m, 6H), 1.34 – 1.30 (m, 2H). ^{13}C NMR (126 MHz, CD_3OD) δ 175.71, 161.52, 154.60, 153.92, 150.94, 149.35, 145.44, 145.17, 142.02, 138.71, 135.51, 134.43, 131.99, 130.49, 130.24, 129.78, 128.24, 127.86, 127.65, 126.76, 126.38, 126.06, 125.97, 125.70, 124.07, 123.88, 123.50, 119.09, 118.91, 113.67, 110.25, 109.84, 99.67, 89.71, 55.96, 47.53, 44.76, 40.06, 36.62, 30.79, 30.72, 30.13, 29.97, 27.38, 27.23, 26.98, 26.31. HRMS $[M]^+$ m/z calcd. for $[C_{47}H_{51}N_8O_2S]^+$ 791.3850, found 791.3844.

(*Z*)-1-(6-((6-(((2-amino-4-oxo-4,7-dihydro-1*H*-pyrrolo[2,3-*d*]pyrimidin-5-yl)methyl)amino)hexyl)amino)-6-oxohexyl)-4-((3-benzylbenzo[*d*]thiazol-2(3*H*)-ylidene)methyl)quinolin-1-ium formate **6b**: 5.2 mg (9.3 µmol) of **4d**, 2.7 mg (9.7 µmol) of **8**, 3.5 mg (9.7 µmol) of HATU and 8.8 µL (50 µmol) of DIEA were stirred in 200 µL of DMF to yield 4.1 mg of **6b** as a red solid in a 54% yield. ^{1}H NMR (500 MHz, CD_3OD) δ 8.46 (d, *J* = 6.8 Hz, 1H), 8.21 (d, *J* = 8.4 Hz, 1H), 8.05 (d, *J* = 8.5 Hz, 1H), 7.97 – 7.90 (m, 2H), 7.66 (t, *J* = 7.9 Hz, 2H), 7.58 (t, *J* = 7.6 Hz, 1H), 7.49 (d, *J* = 6.5 Hz, 1H), 7.45 – 7.36 (m, 3H), 7.33 (d, *J* = 7.4 Hz, 3H), 6.85 (s, 1H), 6.82 (s, 1H), 5.80 (s, 2H), 4.59 (t, *J* = 6.8 Hz, 2H), 4.21 (s, 2H), 3.12 (t, *J* = 6.7 Hz, 2H), 3.00 (t, *J* = 6.7 Hz, 2H), 2.19 (t, *J* = 7.1 Hz, 2H), 2.01 – 1.90 (m, 2H), 1.74 – 1.62 (m, 4H), 1.51 – 1.36 (m, 6H), 1.34 – 1.30 (m, 2H). ^{13}C NMR (126 MHz, CD_3OD) δ 175.68, 161.45, 154.56, 153.95, 151.00, 145.31, 145.26, 141.96, 138.75, 135.86, 134.55, 130.35, 129.66, 129.36, 128.23, 127.65, 127.56, 126.22, 126.10, 126.02, 125.61, 123.91, 123.89, 119.13, 119.06, 113.80, 110.05, 109.84, 99.69, 89.83, 55.97, 50.59, 49.85, 47.54, 44.78, 40.07, 36.65, 30.10, 30.00, 27.27, 27.19, 27.01, 26.32. HRMS $[M]^+$ m/z calcd. for $[C_{43}H_{49}N_8O_2S]^+$ 741.3694, found 741.3688.

(*Z*)-1-(6-((6-(((2-amino-4-oxo-4,7-dihydro-1*H*-pyrrolo[2,3-*d*]pyrimidin-5-yl)methyl)amino)hexyl)amino)-6-oxohexyl)-4-((3-(2,4,6-trifluorobenzyl)benzo[*d*]thiazol-2(3*H*)-ylidene)methyl)quinolin-1-ium formate **6c**: 5.9 mg (9.6 µmol) of **4e**, 2.7 mg (9.7 µmol) of **8**, 3.5 mg (9.7 µmol) of HATU and 8.8 µL (50 µmol) of DIEA were stirred in 200 µL of DMF to yield 3.3 mg of **6c** as a red solid in a 55% yield. ^{1}H NMR (500 MHz, CD_3OD) δ 8.50 (d, *J* = 7.1 Hz, 1H), 8.46 (d, *J* = 8.4 Hz, 1H), 8.10 (d, *J* = 8.7 Hz, 1H), 8.00 (t, *J* = 7.7 Hz, 1H), 7.89 (d, *J* = 7.9 Hz, 1H), 7.82 – 7.74 (m, 2H), 7.60 (t, *J* = 7.8 Hz, 1H), 7.52 (d, *J* = 7.0 Hz, 1H), 7.42 (t, *J* = 7.6 Hz, 1H), 7.00 (t, *J* = 7.9 Hz, 2H), 6.91 (s, 1H), 6.82 (s, 1H), 5.83 (s, 2H), 4.62 (t, *J* = 7.2 Hz, 2H), 4.21 (s, 2H), 3.12 (t, *J* = 7.0 Hz, 2H), 3.01 (t, *J* = 7.3 Hz, 2H), 2.20 (t, *J* = 7.2 Hz, 2H), 1.98 (p, *J* = 7.3 Hz, 2H), 1.68 (h, *J* = 7.4, 6.0 Hz, 4H), 1.49 – 1.38 (m, 6H), 1.33 – 1.30 (m, 2H).^{13}C NMR (126 MHz, CD_3OD) δ 175.70, 165.54, 163.90, 162.00, 161.18, 154.57, 153.95, 151.20, 145.53, 145.48, 141.61, 138.82, 134.69, 129.25, 128.41, 126.35, 126.14, 125.98, 125.89, 125.11, 123.75, 123.72, 119.24, 119.12, 113.96, 110.34, 109.85, 102.05, 99.69, 89.42, 56.09, 47.55, 44.78, 40.08, 39.93, 36.65, 30.10, 30.04, 27.28, 27.20, 27.04, 26.99, 26.33. HRMS $[M]^+$ m/z calcd. for $[C_{43}H_{46}F_3N_8O_2S]^+$ 795.3411, found 795.3407.

(*Z*)-1-(6-((6-(((2-amino-4-oxo-4,7-dihydro-1*H*-pyrrolo[2,3-*d*]pyrimidin-5-yl)methyl)amino)hexyl)amino)-6-oxohexyl)-4-((3-(thiophen-3-ylmethyl)benzo[*d*]thiazol-2(3*H*)-ylidene)methyl)quinolin-1-ium formate **6d**: 4.1 mg (7.2 µmol) of **4h**, 2 mg (7.2 µmol) of **8**, 2.7 mg (7.2 µmol) of HATU and 6.3 µL (36 µmol) of DIEA were stirred in 200 µL of DMF to yield 3.2 mg of **6d** as a red solid in a 56% yield. ^{1}H NMR (500 MHz, CD_3OD) δ 8.47 (d, *J* = 7.0 Hz, 1H), 8.37 (d, *J* = 8.4 Hz, 1H), 8.07 (d, *J* = 8.7 Hz, 1H), 8.01 – 7.88 (m, 2H), 7.73 (q, *J* = 7.9 Hz, 2H), 7.66 – 7.58 (m, 1H), 7.56 – 7.39 (m, 4H), 7.16 – 7.04 (m, 1H), 6.97 (s, 1H), 6.83 (s, 1H), 5.80 (s, 2H), 4.61 (t, *J* = 7.1 Hz, 2H), 4.22 (s, 2H), 3.13 (t, *J* = 7.0 Hz, 2H), 3.01 (t, *J* = 7.1 Hz, 2H), 2.21 (t, *J* = 7.1 Hz, 2H), 2.05 – 1.93 (m, 2H), 1.75 – 1.63 (m, 4H), 1.50 – 1.38 (m, 6H), 1.33 (d, *J* = 7.2 Hz, 2H).^{13}C NMR (126 MHz, CD_3OD) δ 175.70, 161.36, 154.55, 153.97, 151.05, 145.37, 145.11, 141.63, 138.79, 136.36, 134.56, 129.53, 128.87, 128.57, 128.25, 127.37, 127.04, 126.45, 126.27, 125.67, 124.31, 123.88, 119.03, 113.74, 110.19, 109.86, 99.69, 89.82, 55.96, 47.57, 46.67, 44.81,

40.09, 36.66, 30.12, 30.01, 27.28, 27.21, 27.04, 26.99, 26.33. HRMS $[M]^+$ m/z calcd. for $[C_{41}H_{47}N_8O_2S_2]^+$ 747.3258, found 747.3252.

(*Z*)-1-(6-((6-(((2-amino-4-oxo-4,7-dihydro-1*H*-pyrrolo[2,3-*d*]pyrimidin-5-yl)methyl)amino)hexyl)amino)-6-oxohexyl)-4-((3-phenethylbenzo[*d*]thiazol-2(3*H*)-ylidene)methyl)quinolin-1-ium formate **6e**: 4.2 mg (7.3 µmol) of **4f** and 2 mg (7.2 µmol) of **8**, 2.7 mg (7.2 µmol) of HATU and 6.3 µL (36 µmol) of DIEA were stirred in 200 µL of DMF to yield 1.1 mg of **6e** as a red solid in a 19% yield. ^{1}H NMR (500 MHz, CD_3OD) δ 8.36 (d, *J* = 7.2 Hz, 1H), 8.27 (d, *J* = 8.3 Hz, 1H), 8.03 (d, *J* = 8.6 Hz, 1H), 7.96 (t, *J* = 7.6 Hz, 1H), 7.87 (d, *J* = 7.8 Hz, 1H), 7.73 (t, *J* = 7.7 Hz, 1H), 7.55 (d, *J* = 5.8 Hz, 2H), 7.40 (d, *J* = 7.2 Hz, 2H), 7.19 – 7.11 (m, 4H), 7.03 (t, *J* = 6.6 Hz, 1H), 6.82 (s, 1H), 6.60 (s, 1H), 4.56 (t, *J* = 7.3 Hz, 2H), 4.21 (s, 2H), 3.27 (t, *J* = 6.5 Hz, 2H), 3.14 (t, *J* = 7.1 Hz, 2H), 3.02 (t, *J* = 7.4 Hz, 2H), 2.20 (t, *J* = 7.3 Hz, 2H), 1.97 (p, *J* = 7.6 Hz, 2H), 1.69 (h, *J* = 7.8 Hz, 4H), 1.51 – 1.37 (m, 6H), 1.37 – 1.24 (m, 4H). ^{13}C NMR (126 MHz, CD_3OD) δ 175.71, 161.92, 154.55, 153.97, 150.69, 144.90, 144.68, 141.39, 138.91, 138.73, 134.41, 130.33, 130.03, 130.03, 129.71, 129.26, 128.28, 127.90, 126.88, 126.68, 125.98, 125.60, 123.62, 119.19, 118.83, 113.95, 109.85, 109.31, 99.69, 89.63, 55.75, 48.94, 47.57, 44.80, 40.11, 36.68, 34.57, 30.13, 29.99, 27.31, 27.21, 27.08, 27.01, 26.35. HRMS $[M]^+$ m/z calcd. for $[C_{44}H_{51}N_8O_2S]^+$ 755.3850, found 755.3849.

(*Z*)-1-(6-((6-(((2-amino-4-oxo-4,7-dihydro-1*H*-pyrrolo[2,3-*d*]pyrimidin-5-yl)methyl)amino)hexyl)amino)-6-oxohexyl)-4-((3-(3,5-bis(methoxycarbonyl)benzyl)benzo[*d*]thiazol-2(3*H*)-ylidene)methyl)quinolin-1-ium formate **6f**: 6.3 mg (9.3 µmol) of **4i**, 2.6 mg (9.3 µmol) of **8**, 3.5 mg (9.3 µmol) of HATU and 8.8 µL (50 µmol) of DIEA were stirred in 200 µL of DMF to yield 3.3 mg of **6f** as a red solid in a 39% yield. ^{1}H NMR (500 MHz, CD_3OD) δ 8.56 (s, 1H), 8.51 (d, *J* = 7.3 Hz, 1H), 8.36 (d, *J* = 8.6 Hz, 1H), 8.23 (s, 2H), 8.08 (d, *J* = 8.6 Hz, 1H), 8.04 – 7.93 (m, 2H), 7.77 – 7.66 (m, 2H), 7.65 – 7.59 (m, 1H), 7.54 (d, *J* = 7.1 Hz, 1H), 7.51 – 7.44 (m, 1H), 6.89 (s, 1H), 6.81 (s, 1H), 5.96 (s, 2H), 4.63 (q, *J* = 8.1, 7.6 Hz, 2H), 4.19 (s, 2H), 3.90 (s, 6H), 3.12 (d, *J* = 6.9 Hz, 2H), 3.08 – 2.95 (m, 2H), 2.27 – 2.17 (m, 2H), 1.98 (d, *J* = 7.6 Hz, 2H), 1.68 (d, *J* = 7.2 Hz, 4H), 1.54 – 1.42 (m, 4H), 1.36 – 1.27 (m, 4H). ^{13}C NMR (126 MHz, CD_3OD) δ 174.28, 165.48, 165.48, 161.26, 159.75, 153.13, 152.53, 149.78, 144.19, 144.08, 140.28, 137.34, 136.36, 133.28, 131.57, 131.57, 129.69, 129.62, 128.37, 127.05, 125.01, 124.91, 124.77, 124.10, 122.67, 117.76, 117.61, 112.15, 108.99, 108.63, 98.25, 88.11, 54.71, 52.76, 51.82, 51.70, 46.19, 43.40, 38.67, 35.21, 29.44, 29.31, 28.74, 28.62, 25.90, 25.61, 24.91. HRMS $[M]^+$ m/z calcd. for $[C_{47}H_{53}N_8O_6S]^+$ 857.3803, found 857.3795.

8. Synthesis of preQ$_1$-C6-TO-Me **1b**

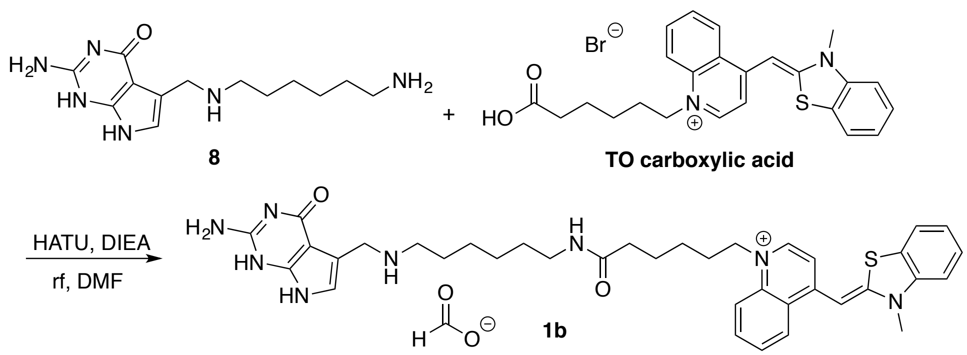

Scheme S8. Synthesis of preQ$_1$-C6-TO-Me **1b.**

In a 4 mL glass vial, 2.6 mg (5.4 µmol) of TO carboxylic acids (Otava Chemicals, Canada) was dissolved in 0.5 mL DMF anhydrous and the mixture was cooled to 0 °C. 2 mg (5.3 µmol) of HATU and 3.4 mg (26.5 µmol) of DIEA were added and the mixture was stirred at 0 °C for 10 min. 1.5 mg (5.4 µmol) of **8** in DMF was then added to the stirring mixture. The mixture was continued to stir for 1 h and reaction was monitored by LC-MS. Upon the completion of the reaction, the solvent was removed *in vacuo* and the residue was purified by HPLC. 1.4 mg of **1b** was collected as a red solid in a 37% yield. HPLC gradient: 0 min – 2.5 min 50% *Phase B* in *Phase A*, 2.5 min – 18 min 50% *Phase B* in *Phase A* to 90% *Phase B*, 18 min – 28 min 95% *Phase B*, 28 min – 32 min 95% *Phase B* in *Phase A* (*Phase A*: H_2O with 0.1% formic acid, *Phase B*: MeOH with 0.1% formic acid). ^{1}H NMR (500 MHz, CD_3OD) δ 8.68 (t, *J* = 8.4 Hz, 2H), 8.17 (d, *J* = 8.7 Hz, 1H), 8.08 (dd, *J* = 7.9, Hz, 1H), 8.00 (t, *J* = 7.5 Hz, 1H), 7.89 (d, *J* = 8.3 Hz, 1H), 7.76 (ddd, *J* = 52.1, 7.9, 0.0 Hz, 1H), 7.64 – 7.54 (m, 2H), 7.49 – 7.40 (m, 2H), 7.16 – 7.01 (m, 2H), 5.92 (s,

2H), 4.61 (t, 2H), 2.21 (t, J = 7.1 Hz, 2H), 1.92 – 1.78 (m, 2H), 1.62 – 1.48 (m, 2H), 1.47 – 1.32 (m, 2H). ^{13}C NMR (126 MHz, CD_3OD) δ 174.43, 159.28, 148.66, 144.60, 140.00, 136.97, 135.53, 133.37, 128.31, 127.77, 127.05, 126.78, 125.67, 124.62, 124.35, 123.89, 123.09, 118.27, 113.06, 108.33, 88.21, 54.13, 44.73, 33.53, 28.68, 25.47, 24.05. HRMS $[M]^+$ m/z calcd. for $[C_{37}H_{45}N_8O_2S]^+$ 665.3381, found 665.3375.

9. Synthesis of preQ$_1$-C6-NHBoc **7**

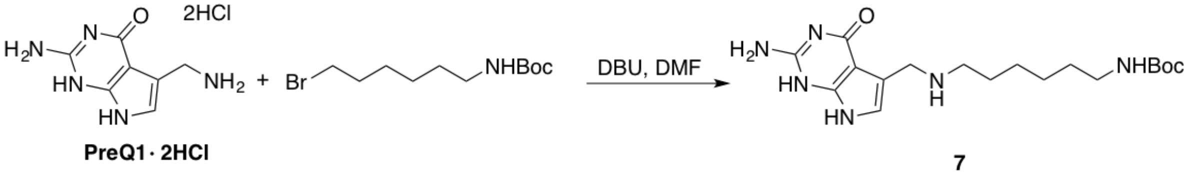

Scheme S9. Synthesis of preQ$_1$-PEG3-NHBoc **7**.

100 mg (0.40 mmol) of preQ$_1$ dihydrochloride was suspended in DMF. 182 mg (1.2 mmol) of DBU was added to the solution and the solid became fully dissolved. 78 mg (0.40 mmol) of 6-bromohexanoic acid was slowly added to the reaction mixture and the mixture was stirred at room temperature for 2 h. The solvent was removed *in vacuo* and the residue was purified by silica chromatography (DCM : MeOH : 30% NH_4OH = 7:1:0.1). 53 mg of 7 was collected as a pale yellow solid in a 35% yield. ^{1}H NMR (500 MHz, CD_3OD) δ 6.84 (s, 1H), 4.25 (s, 2H), 3.03 (dt, J = 11.2, 7.2 Hz, 4H), 1.73 (p, J = 7.5 Hz, 2H), 1.51 – 1.46 (m, 2H), 1.42 (s, 11H), 1.37 – 1.32 (m, 2H). ^{13}C NMR (126 MHz, CD_3OD) δ 161.34, 157.23, 153.21, 152.55, 117.89, 108.49, 98.34, 78.54, 46.28, 43.42, 39.72, 29.28, 27.45, 27.45, 27.45, 25.89, 25.82, 25.73. HRMS $[M]^+$ m/z calcd. for $[C_{18}H_{30}N_6O_3]^+$ 378.2379, found 378.2376.

10. Synthesis of preQ$_1$-C6-NH_2 **8**

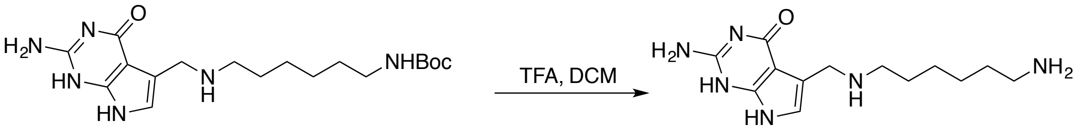

Scheme S10. Synthesis of preQ$_1$-PEG3-NH_2 **8**.

20 mg of **7** was suspended in 1 mL DCM and 100 μL of TFA was slowly added to the reaction mixture. The suspension became clear and the mixture was allowed to stir at room temperature for 1 h. The solvent was removed *in vacuo*. The residue was basified by the addition of triethylamine. The resulting **8** as a yellow oil was used in the next reaction without purification.

11. Fixed cell labeling of mCherry mRNA with **1a**.

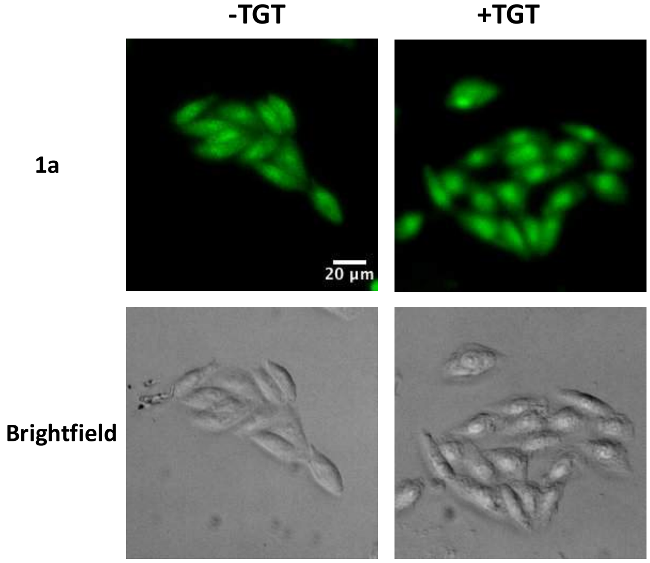

Fig. S1. TGT labeling of expressed mRNA with **1a**. The cell labeling experiment was performed as in Section 18, Fig. S5. mCherry construct transfected CHO cells were fixed, permeabilized, and treated with 1 μM **1a** in the presence or absence of 1 μM TGT. Cells were incubated at 37 °C for 3 h and imaged using a fluorescence microscope. Similar high fluorescent intensities were observed for both cells with and without the treatment of TGT. The high background staining suggests that **1a** is not a suitable probe for cellular mRNA imaging.

12. Preparation of plasmid constructs

12.1 mCherry plasmid

A synthetic gene block containing the sequence for mCherry was designed and ordered from IDT (Coralville, IA). Specifically, the gene was designed with the ECY-A1 hairpin downstream of the mCherry coding region within the 3' UTR. The geneblock was cloned into the mammalian expression vector pcDNA3 (Thermo Fisher, Waltham, MA) between cut sites BamHI and XhoI. DH5a competent cells (Life Technologies, Carlsbad, CA) were then transformed with the ligation product and screened against ampicillin on agar plates overnight. Colonies were selected and overgrown, and the overgrowth was subjected to DNA extraction with a QIAGEN Plasmid Maxi Kit (Qiagen, Venlo, Limburg Netherlands) and sequencing was performed to verify the inserted gene.

GGATCCACTAGTAACGGCCGCCAGTGTGCTGGAATTCTGCAGATATCGTTGACCTTGCAGAAGGAGAT
ATAATGGTGAGCAAGGGCGAGGAGGATAACATGGCCATCATCAAGGAGTTCATGCGCTTCAAGGTGC
ACATGGAGGGCTCCGTGAACGGCCACGAGTTCGAGATCGAGGGCGAGGGCGAGGGCCGCCCCTACGA
GGGCACCCAGACCGCCAAGCTGAAGGTGACCAAGGGTGGCCCCCTGCCCTTCGCCTGGGACATCCTGT
CCCCTCAGTTCATGTACGGCTCCAAGGCCTACGTGAAGCACCCCGCCGACATCCCCGACTACTTGAAG
CTGTCCTTCCCCGAGGGCTTCAAGTGGGAGCGCGTGATGAACTTCGAGGACGGCGGCGTGGTGACCGT
GACCCAGGACTCCTCCCTGCAAGACGGCGAGTTCATCTACAAGGTGAAGCTGCGCGGCACCAACTTCC
CCTCCGACGGCCCCGTAATGCAGAAGAAGACGATGGGCTGGGAGGCCTCCTCCGAGCGGATGTACCC
CGAGGACGGCGCCCTGAAGGGCGAGATCAAGCAGAGGCTGAAGCTGAAGGACGGCGGCCACTACGA
CGCTGAGGTCAAGACCACCTACAAGGCCAAGAAGCCCGTGCAGCTGCCCGGCGCCTACAACGTGAAC
ATCAAGTTGGACATCACCTCCCACAACGAGGACTACACCATCGTGGAACAGTACGAACGCGCCGAGG
GCCGCCACTCCACCGGCGGCATGGACGAGCTGTACAAGTAATATCTAACACAGACTCTCGGTACCATC
ATTTTCATATCCCCGCAGACTGTAAATCTGCACCACCATCATTTAATGAATTCCATCAGGAATCCCTCA
CTTAAAGCCCGCCGAAAGGCGGGCTTTTCTGTGTCTGCAGACTGGCCGTCGTTTACACTCGAG

12.2 CMV-TGT expression plasmid

A mammalian codon-optimized TGT expression vector was ordered from Genscript (Piscataway, NJ) with the following coding sequence inserted between EcoRI and XhoI cut sites of pcDNA3.1(+) mammalian expression vector:

GAATTCGCCACCATGAAGTTTGAGCTGGATACTACTGATGGGCGGGCTAGAAGAGGCAGACTGGTGTT
TGATAGAGGCGTCGTCGAGACCCCCTGTTTTATGCCAGTGGGCACCTACGGCACAGTGAAGGGAATGA
CCCCAGAGGAGGTGGAGGCTACAGGCGCTCAGATCATCCTGGGCAACACCTTCCACCTGTGGCTGCGG
CCTGGCCAGGAGATCATGAAGCTGCACGGCGACCTGCACGACTTCATGCAGTGGAAGGGCCCAATCCT
GACCGACTCCGGCGGCTTCCAGGTGTTTTCTCTGGGCGACATCCGCAAGATCACAGAGCAGGGCGTGC
ACTTCCGGAACCCCATCAATGGCGACCCTATCTTCCTGGACCCCGAGAAGAGCATGGAGATCCAGTAT
GACCTGGGCTCCGACATCGTGATGATCTTCGACGAGTGCACACCCTACCCTGCCGACTGGGATTATGC
TAAGCGCAGCATGGAGATGTCCCTGCGGTGGGCCAAGAGATCTCGCGAGCGGTTCGATAGCCTGGGC
AACAAGAATGCTCTGTTTGGCATCATCCAGGGCAGCGTGTACGAGGACCTGAGGGACATCAGCGTGA
AGGGCCTGGTGGACATCGGCTTTGATGGCTATGCTGTGGGAGGACTGGCTGTGGGAGAGCCAAAGGC
CGACATGCACAGAATCCTGGAGCACGTGTGCCCACAGATCCCCGCTGACAAGCCCAGATACCTGATGG
GCGTGGGCAAGCCTGAGGATCTGGTGGAGGGCGTGCGGAGGGGAATCGACATGTTCGATTGCGTGAT
GCCTACCAGGAACGCCAGAAATGGCCACCTGTTTGTGACAGACGGCGTGGTGAAGATCCGCAATGCC
AAGTACAAGTCCGATACCGGCCCTCTGGACCCAGAGTGCGATTGTTATACATGCCGCAACTACTCTCG
GGCCTATCTGCACCACCTGGACAGGTGTAATGAGATCCTGGGCGCTAGACTGAACACCATCCACAATC
TGAGGTACTATCAGAGACTGATGGCTGGACTGAGGAAGGCTATCGAGGAGGGCAAGCTGGAGAGCTT
CGTGACAGACTTCTACCAGCGGCAGGGCAGAGAGGTCCCACCACTGAATGTCGATCATCACCACCATC
ACCACTGACTCGAG

13. *In Vitro* transcription of mCherry mRNA

20 µL 600 ng/µL mCherry construct plasmid DNA was digested by 20 units XhoI in 500µL 1x CutSmart buffer in 37 °C warm water bath for 1h. Linearized DNA was then extracted with molecular biology grade phenol / chloroform / isoamyl alcohol (25:24:1) (Sigma-Aldrich, St. Louis, MO), and purified by ethanol precipitation. The DNA pellet was dissolved in RNAase free water. To set up *in vitro* transcription reaction, 15 µL of 333 ng/µL linearized DNA (5.0 µg), 44.1 µL RNAse free water, 10 µL of 10x T7-RNAP buffer, 20 µL NTPs (ATP, CTP, GTP, UTP, 25 mM each), additional 4 µL of 100 mM GTP, 0.2 µL of 2 U/µL inorganic PPase, 0.5 µL RNAse inhibitor murine, 1.2 µL of 12.5 µg/µL T7-RNAP were mixed together to give a total reaction volume of 100 µL. The transcription was run at 37 °C overnight. The reaction mixture was treated with 1.2 µL of 100 mM $CaCl_2$ followed by 1 µL 20 Units of Turbo DNAse (Life Technologies, Carlsbad, CA) at 37 °C for 30 min. The mixture was centrifuged at max speed (16,100 g) for 2 min. The supernatant was then added with 50 µL of 8 M LiCl and cooled to -20 °C for 40 min. The mixture was centrifuged again at 4 °C at max speed for 30 min. The supernatant was discarded, the pellet was allowed to dry at room temperature for 5 minutes by evaporation, and the pellet was subsequently dissolved in 100 µL RNAse free water to give a final mRNA concentration of 4800 ng/µL.

14. Fluorescent assay for TO **5a-f**/mCherry mRNA binding

TO probe/mRNA binding was analyzed by using a Tecan Saphire-II plate reader in a Greiner 384 well flat bottom plate (VWR, CAT# 89089-584). Each well was prepped with a variable amount (from 200 nM to 0.781 nM in serial 1/2 dilutions) of mCherry mRNA in 20 µL TGT reaction buffer. 5 µL of 25 µM TO **4a-f** was added to each dilution serial in triplicate. The plate was incubated at 37 °C for 20 min. For the fluorescent measurements, each well was monitored with an excitation of 501 nm and emission of 531. The excitation bandwidth was set to 20 nm and the emission bandwidth was set to 5 nm. The gain was held fixed and the integration time set to 1 second with 10 reads averaged to each data point collected. The data was plotted and analyzed in Graph Pad Prism v. 6.0a (La Jolla, CA).

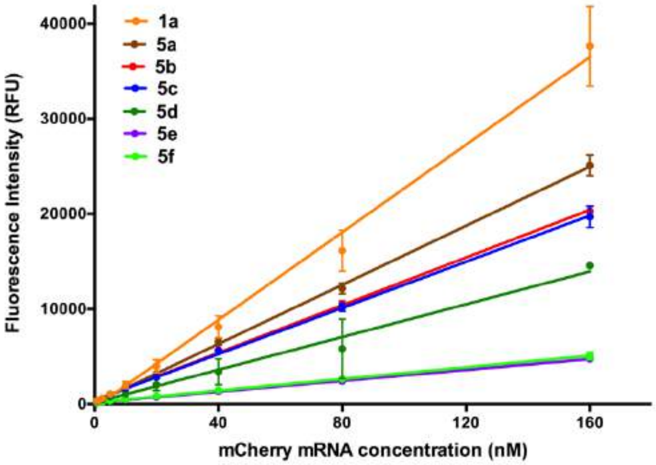

Fig. S2: Plot of raw fluorescence data as a function of mRNA concentration. Various concentration (160 nM to 0.625 nm) of mCherry mRNA was treated with 5 µM of TO **5a-f**. The fluorescence of TO-RNA binding decreases as the steric of the TO moiety increases (Me>Et>Pr>iPrBn). Error bar denotes the SD of three measurements.

15. Fluorescent turn-on measurements for mRNA labeling with **5a-f** and **6a-f**

20 µL mRNA labeling reactions were setup with 1 µM TGT enzyme, 1 µM PreQ1-TO **5a-f** or **6a-f**, and 100 nM mCherry mRNA in TGT reaction buffer. The reaction mixture was incubated at 37 °C for 2 hours. After the reaction, 180 µL of 1x TGT buffer was added to the reaction. Samples were measured on a JASCO FP-8500 fluorimeter by excitation at 505 nm and scanning emission from 525 nm to 580 nm, with excitation and emission slit at 5 nm and an integration time of 1 s. All reactions and measurements were performed in triplicates.

16. Quantum yield measurements for mRNA labeled **6f**.

200 µL mRNA labeling reactions were setup with 1 µM TGT enzyme, 1 µM **6f**, and 2 µM mCherry mRNA in TGT reaction buffer. The reaction mixture was incubated at 37 °C for 3 hours. After the reaction, the mRNA was purified by ethanol precipitation for three times. Absorbance was measured by a Thermo Scientific Nano-Drop 2000c UV-Vis spectrophotometer (Waltham, MA). Fluorescence were measured on a JASCO FP-8500 fluorimeter by excitation at 505 nm and scanning emission from 515 nm to 625 nm, with excitation and emission slit at 5 nm and an integration time of 1 s. Quantum yields were assessed by using fluorescein for **6f** as a standard, using excitation at 490 nm and emission from 500 nm to 610 nm. All reactions and measurements were performed in triplicates.

17. ECY-A1 labeling efficiency with **1a** and **1b**

In an Eppendorf tube, RNA labeling reactions were set up with 10 µM ECY-A1 RNA hairpin, 10 µM **1a** or **1b**, and various concentration (0.1 eq, 0.3 eq and 0.5 eq) of TGT enzyme in 30 µL of 1 x TGT buffer. The reaction mixtures were incubated at 37 °C for 2 h and analyzed by HPLC with the following gradient: (*Phase A* = water containing 20% hexafluoroisopropanol (HFIP) and 0.1% TEA, *Phase B* = methanol), 0 - 2 min 5% *Phase B* in *Phase A*, 2 - 10 min 5% *Phase B* in *Phase A* to 55% *Phase B* in *Phase A*. Samples were monitored at 260 nm and 505 nm.

Percentage labeling was calculated using the following equation: Percent labeling = (Area of TO-labeled ECY-A1) / (Area of All Peaks) x 100%.

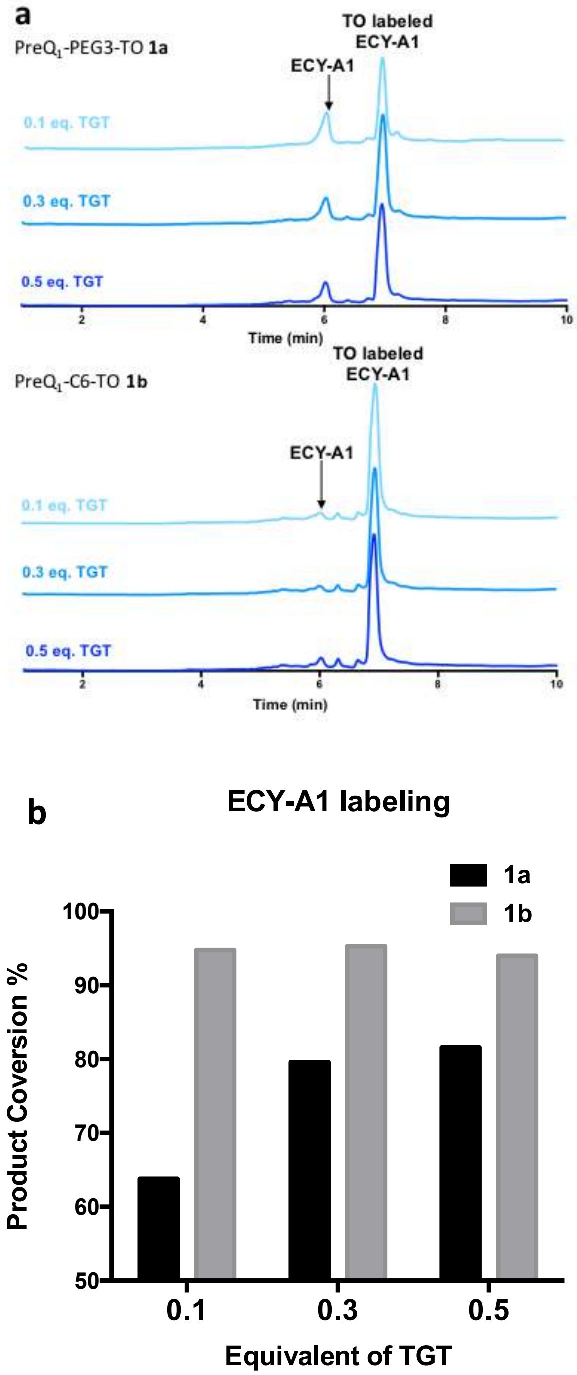

Fig. S3. RNA labeling efficiency with PEG linker probe **1a** or alkyl linker probe **1b**. a. HPLC trace of ECY-A1 labeling reactions with **1a** and **1b** at different TGT concentrations. b. Percent labeling of ECY-A1 RNA with **1a** or **1b** at different concentration of TGT.

18. Estimation of **6f** substrate kinetics with ECY-A1

An estimation of **6f** substrate kinetics with ECY-A1 was performed in a similar fashion to the previous published protocol.[8] A triplicate set of 20 µL reactions was analyzed using a Tecan Saphire-II plate reader in a Greiner 384 well flat bottom plate (VWR, CAT# 89089-584). Each well was prepped with 10 µM ECY-A1 and a variable amount (from 100 µM to 5 nM in serial 1/3 dilutions) of **6f** in TGT reaction buffer. The plate was covered and allowed to warm up to 37 °C for 10 minutes. TGT enzyme was added to a final concentration of 100 nM. The plate was immediately subjected to a fluorescence reading for the first kinetic time point at 37 °C. Each well was monitored with an excitation of 501 nm and emission of 531. The excitation bandwidth was set to 20 nm and the emission bandwidth was set to 5 nm. The gain was held fixed and the integration time was set to 1 second with 10 reads averaged to each data point collected. The sample was monitored for 20 minutes at 37 °C with a reading taken every 2 minutes. An ECYA1-PreQ1-TO calibration curve was used to convert fluorescence into concentration of product in each sample for each time point to achieve an estimation of the initial rate of each reaction. The initial rates were then plotted against the concentration of substrate to achieve a standard kinetic curve.

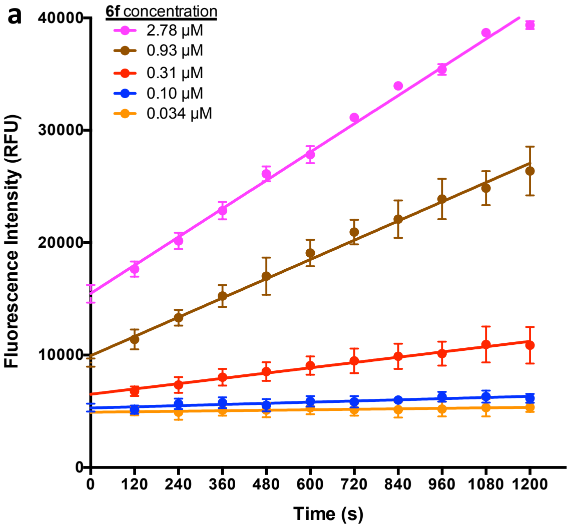

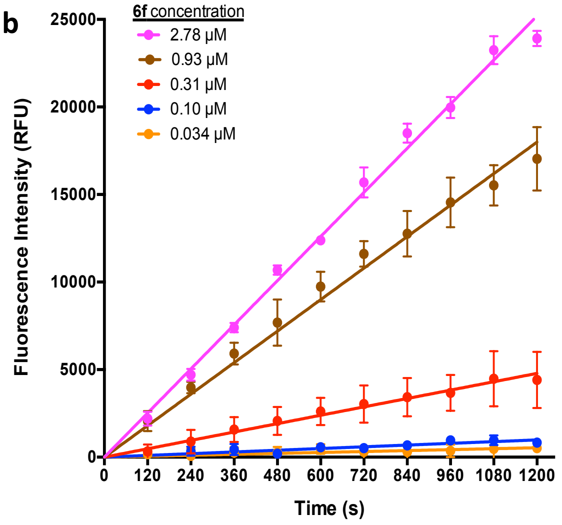

c

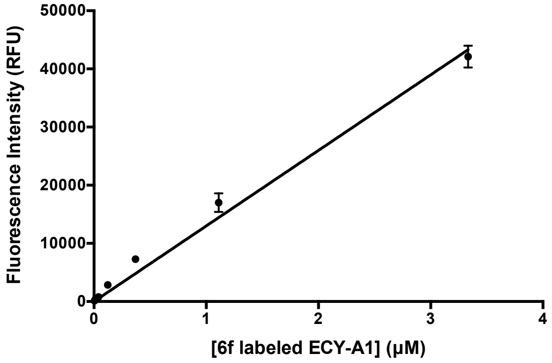

d

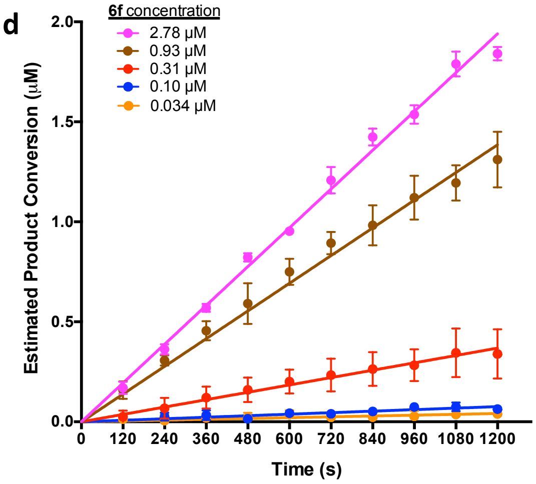

e

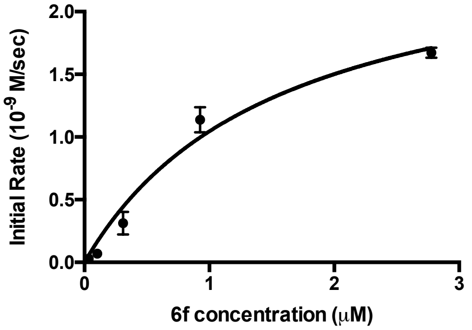

Fig. S4. a. Plot of raw fluorescence data as a function of TGT reaction time progress. b. Plot of background subtracted fluorescence data as a function of TGT reaction time progress. c. Calibration curve of ECY-A1-PreQ1-TO product relating fluorescence and concentration. d. Plot of estimated amount of PreQ1-TO incorporated into ECY-A1 as a function of TGT reaction time progress. e. Plotted initial rates observed from A to determine enzyme reaction kinetic parameters k_{cat} and k_M for PreQ1-TO with ECY-A1.

19. Fixed cell labeling of mCherry mRNA with **6f**

Chinese Hamster Ovary (CHO) cells (CRL-9606, ATCC, Manassas, VA) were cultured in Ham's F-12K media (Life Technologies, Carlsbad, CA). CHO cells were plated at an initial density of 10,000 cells per well in a Nunc Lab-Tek 8 well chamber slide (Thermo Scientific, Waltham, MA). Cells were allowed to adhere overnight and washed with Opti-MEM media (Life Technologies, Carlsbad, CA) and subsequently transfected with 1200 ng of mCherry construct plasmid per well with 1.6 μL of Lipofectamine 2000 (Life Technologies, Carlsbad, CA) in Opti-MEM as per manufacture's protocol. Control wells lacking transfection were treated and washed with Opti-MEM in the absence of DNA and Lipofectamine. After overnight transfection, the cells were washed twice with F-12K media (200 μL each) and allowed to recover at 37 °C for 6 h. The cells were fixed with 100 μL of a 3.7% paraformaldehyde in PBS solution for 30 minutes at room temperature and then permeabilized by treatment with 100 μL of 0.1% Triton X-100 in HBSS buffer (Life Technologies, Carlsbad, CA) for 30 minutes at room temperature and washed 2 x 200 μL with HBSS buffer. Cells were then treated for 3 h at 37 °C with 100 μL of TGT reaction buffer containing 500 nM **6f** either with or without 500 nM TGT enzyme added. After incubation, the cells were directly subjected to fluorescent microscopy imaging. TO probe **6f** was excited with a 488 HeNe laser and mCherry fluorescent protein was excited with a 543nm HeNe laser. Images were analyzed and processed using ImageJ (NIH, rsbweb.nih.gov).

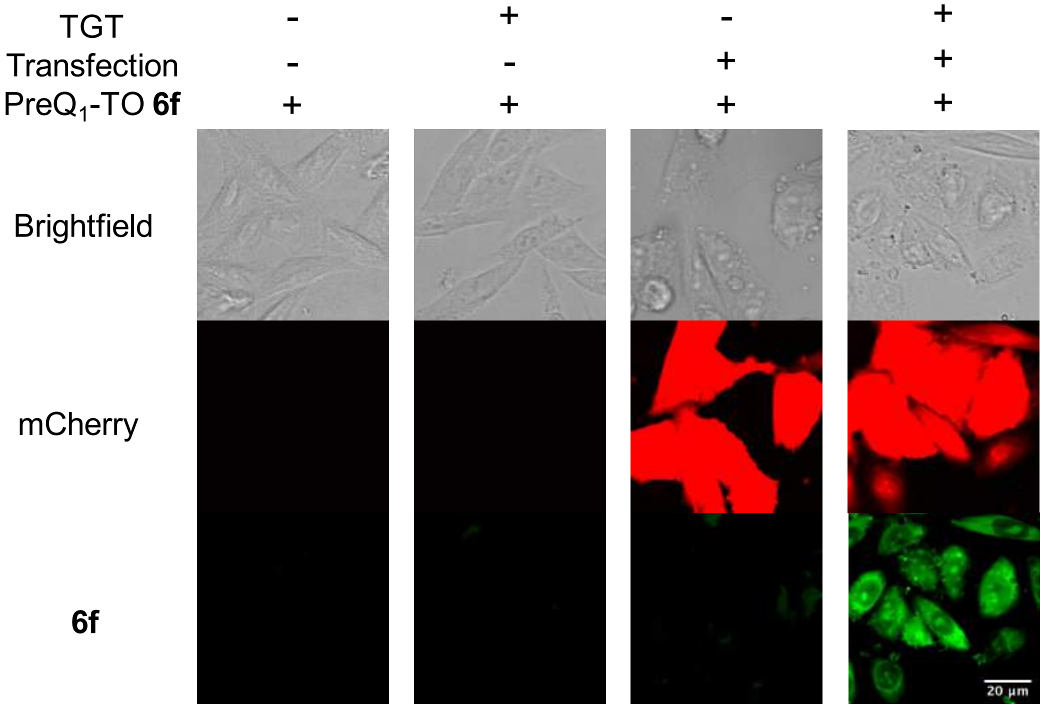

Fig. S5. Fluorescence microscopy of TGT labeling of CHO cell expressed mCherry mRNA. left. Untransfected cells only treated with **6f** do not show green fluorescence. middle left. Untransfected cells treated with both TGT and **6f** do not show green fluorescence. middle right. Transfected cells only treated with **6f** do not show green fluorescence. right. Transfected cells treated with both TGT and **6f** show green fluorescence, suggesting successful labeling of expressed mCherry. Scale bar (20 μm) is common to all image frames.

20. Verification of TGT expression in HeLa cells by immunofluorescent imaging

HeLa cells were first transfected in a similar manner to previous experiments. Cells were plated at 40,000 cells per well in an 8-well chambered lab-tek slide and allowed to adhere overnight. Cells were then washed and transfected with either mCherry plasmid or co-transfected with equal concentrations of mCherry and CMV-TGT plasmids using standard Lipofectamine protocols. After overnight transfection, cells were washed, fixed and permeabilized in a similar manner to prior imaging studies. Fixed cells were then blocked for 30 min. at room temperature with a PBS solution containing 1% BSA and 0.1% Tween 20 (Solution A) to block unspecific binding of antibodies. The cells were then incubated with diluted (1 µg/uL) 6x-His Tag monoclonal antibody, Clone HIS.H8 (# MA121315, Invitrogen) in Solution A overnight at 4 °C. Cells were subsequently washed 3x with PBS and incubated for 1 hour at r.t. in the dark with diluted (1 µg/uL) Donkey anti-mouse IgG (H+L) highly cross-adsorbed secondary antibody conjugated with Alexa Fluor 488 (# A-21202, Invitrogen) in Solution A according to manufacturer's protocols for immunofluorescent imaging. Cells were washed 3x with PBS and imaged by confocal microscopy in a similar fashion to our RNA imaging protocols.

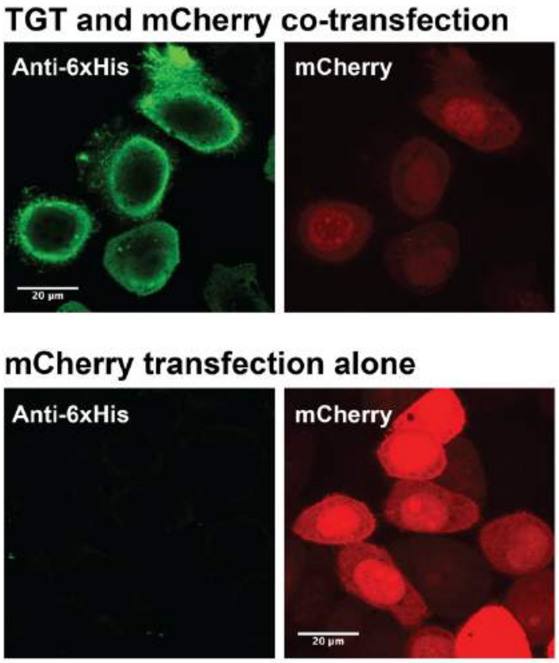

Fig. S6. Varying TGT expression in HeLa cells. HeLa cells were co-transfected with a plasmid capable of expressing TGT along with the mCherry plasmid (top), or mCherry plasmid alone (bottom). After 18 h, the cells were fixed and labeled with a 6x-His mouse antibody overnight at 4 °C, washed, and subsequently labeled with a donkey anti-mouse Alexa-488 conjugated antibody for immunoflurorescent imaging. The secondary antibody was washed and the cells were imaged by confocal microscopy. Anti-6x His antibody should selectively label the His Tag bearing TGT protein (green) which was observable by strong staining of co-transfected cells, while cells expressing mCherry alone were not stained by the anti-His antibody. mCherry expression (red) was evident in mCherry transfected cells, however the expression level was reduced in co-transfected cells presumably due to co-expression with the TGT construct.

21. Live cell labeling of mCherry mRNA with **6f** via microinjection

HeLa cells were cultured in DMEM media (Life Technologies, Carlsbad, CA) and were plated in a 50 mm glass bottom dish (Electron Microscopy Sciences, Hatfield, PA). Cells were allowed to adhere overnight and washed with Opti-MEM media (Life Technologies, Carlsbad, CA). A solution of 10 µL of 10 µM **6f** and 10 µM TGT in 1x TGT buffer was loaded in a femtotip II capillary (Eppendorf, Hamburg, Germany). The mixture was injected in 50 individual cells using an Eppendorf Micromanipulator 5171 and an Eppendorf Microinjector 5242. The injection pressure was set to 1400 hPa and the hold pressure was set to 500 hPa. Control cells were injected with the same concentration of **6f** with no TGT in 1x TGT buffer. The cells were incubated at 37 °C for 3 h and subjected to confocal fluorescence microscopy.

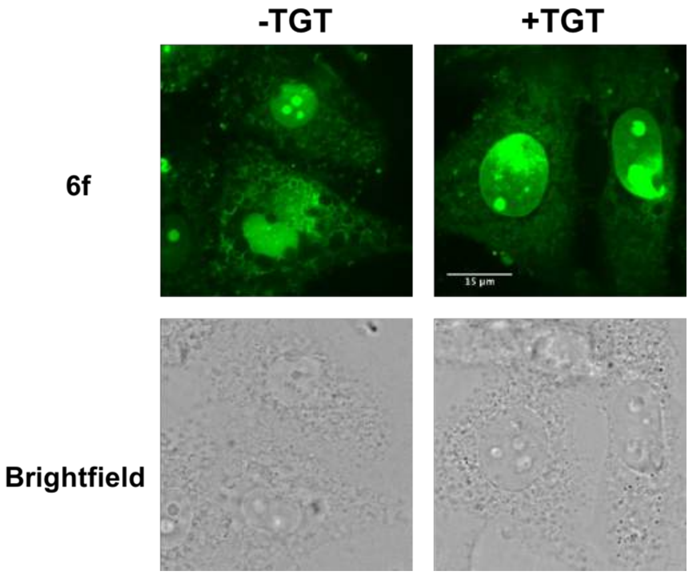

Fig. S7. Fluorescence microscopy images of microinjected HeLa cells. HeLa cells injected with a mixture of **6f** and TGT (right) shows similar fluorescent intensity comparing to the control cells that were injected with only **6f** (left).

22. Fixed cell labeling of mCherry mRNA with increased **6f** concentration

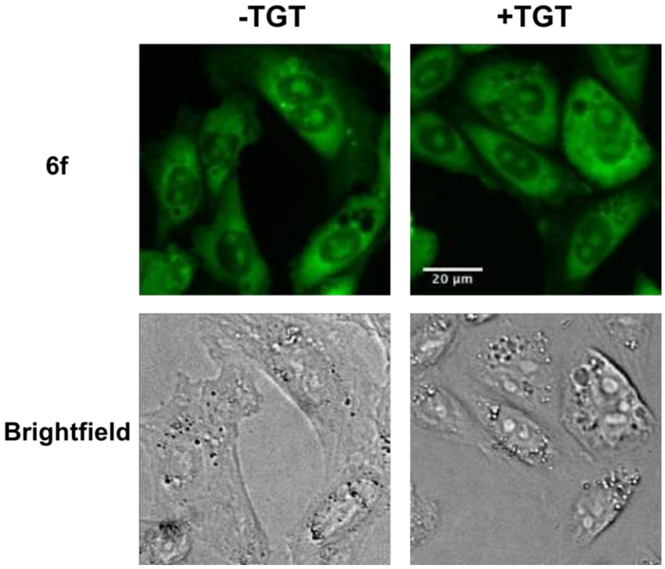

Fig. S8. TGT labeling of expressed mRNA with increased **6f** concentration. The cell labeling experiment was performed as in Section 19, Fig. S5. mCherry construct transfected CHO cells were fixed, permeabilized, and treated with 2 µM **6f** in the presence or absence of 0.5 µM TGT. Cells were incubated at 37 °C for 3 h and imaged using a fluorescence microscope. Similar fluorescent intensity was observed for both cells with and without the treatment of TGT. The high background staining suggests that the increased concentration of **6f** is not a suitable for cellular mRNA imaging.

23. Supporting information references

(1) Liu, J.; Sun, Y.-Q.; Huo, Y.; Zhang, H.; Wang, L.; Zhang, P.; Song, D.; Shi, Y.; Guo, W. *J. Am. Chem. Soc.* **2014**, *136* (2), 574–577.
(2) Ikeda, S.; Okamoto, A. *Chem. Asian J.* **2008**, *3* (6), 958–968.
(3) Li, H.-Y.; Wei, Y.-L.; Dong, X.-Y.; Zang, S.-Q.; Mak, T. C. W. *Chem. Mater.* **2015**, *27* (4), 1327–1331.
(4) Levillain, J; Paquer, D; Sene, A; Vazeux, M. *Synthesis.* **1998**, *1*, 99–104.
(5) Testaferri, L.; Tiecco, M.; Tingoli, M.; Chianelli, D.; Montanucci. M. *Synthesis.* **1983**, 9, 751–755.
(6) Carreon, J. R.; Stewart, K. M.; Mahon, K. P.; Shin, S.; Kelley, S. O. *Bioorg. Med. Chem. Lett.* **2007**, *17* (18), 5182–5185.
(7) Silva, G. L.; Ediz, V.; Yaron, D.; Armitage, B. A. *J. Am. Chem. Soc.* **2007**, *129*, 6710–5718.
(8) Alexander, S. C.; Busby, K. N.; Cole, C. M.; Zhou, C. Y.; Devaraj, N. K. *J. Am. Chem. Soc.* **2015**, *137* (40), 12756–12759.

24. NMR spectra for synthesized material

1b

^1H NMR

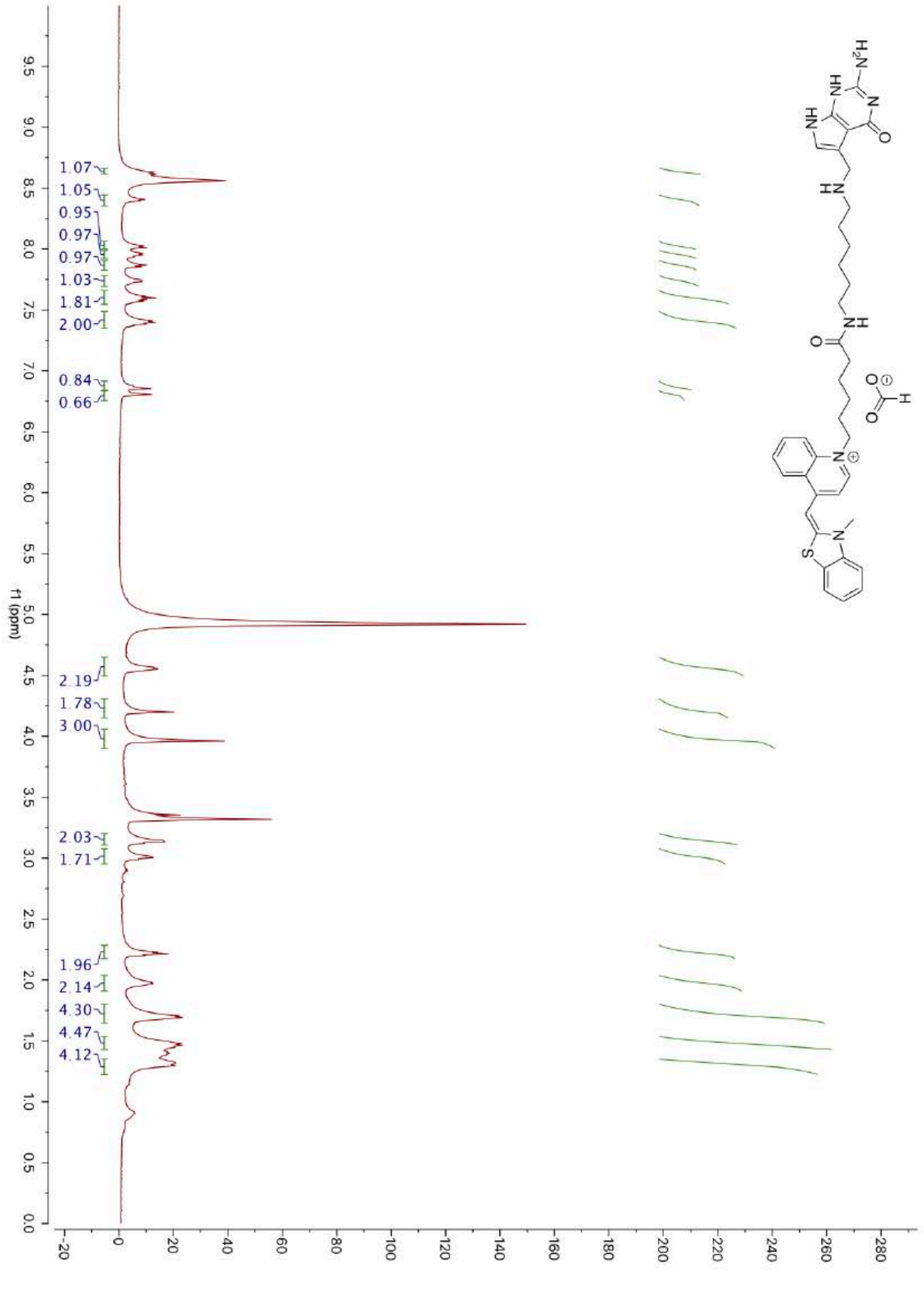

1b

^{13}C NMR

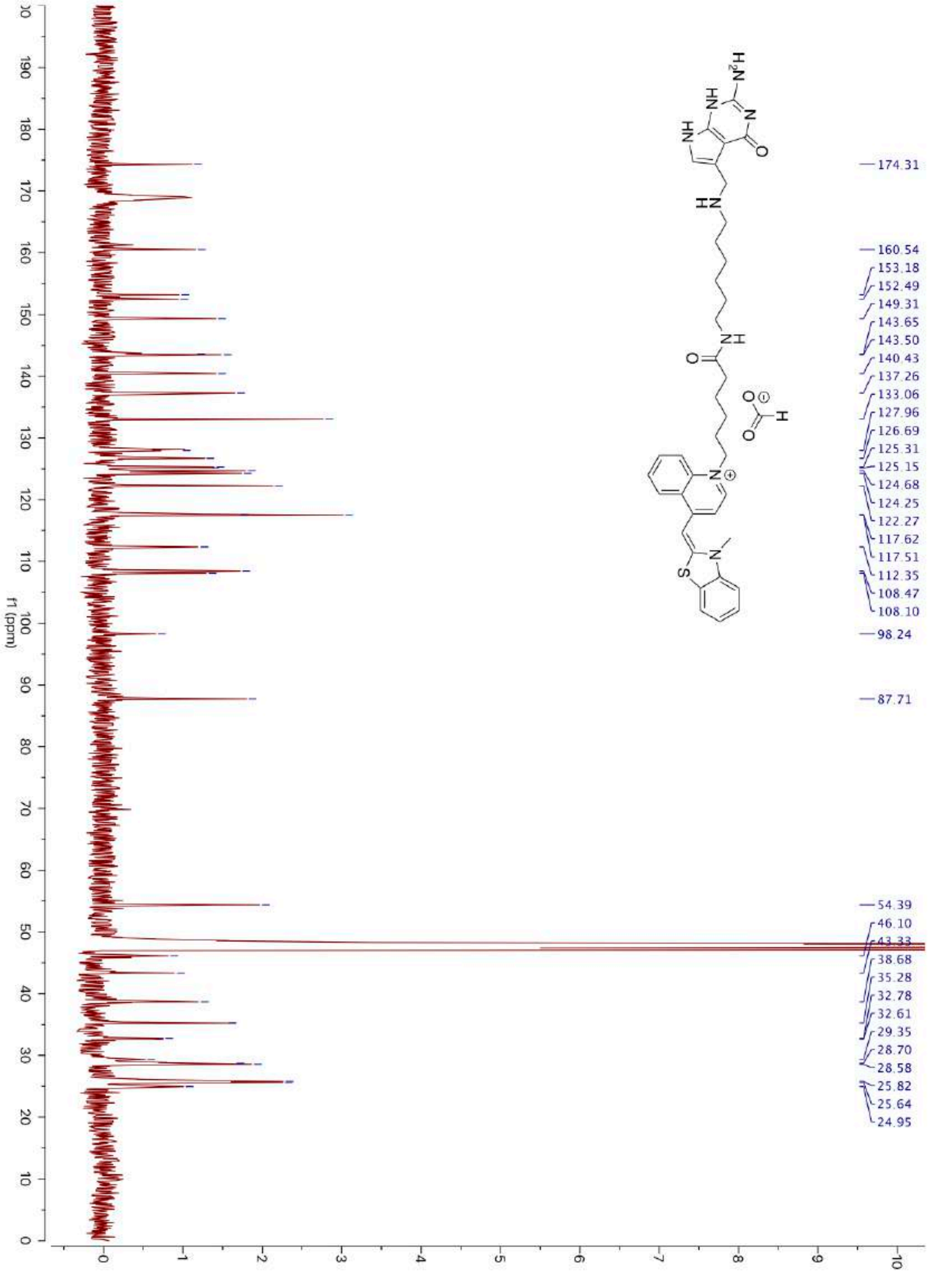

2a

^{1}H NMR

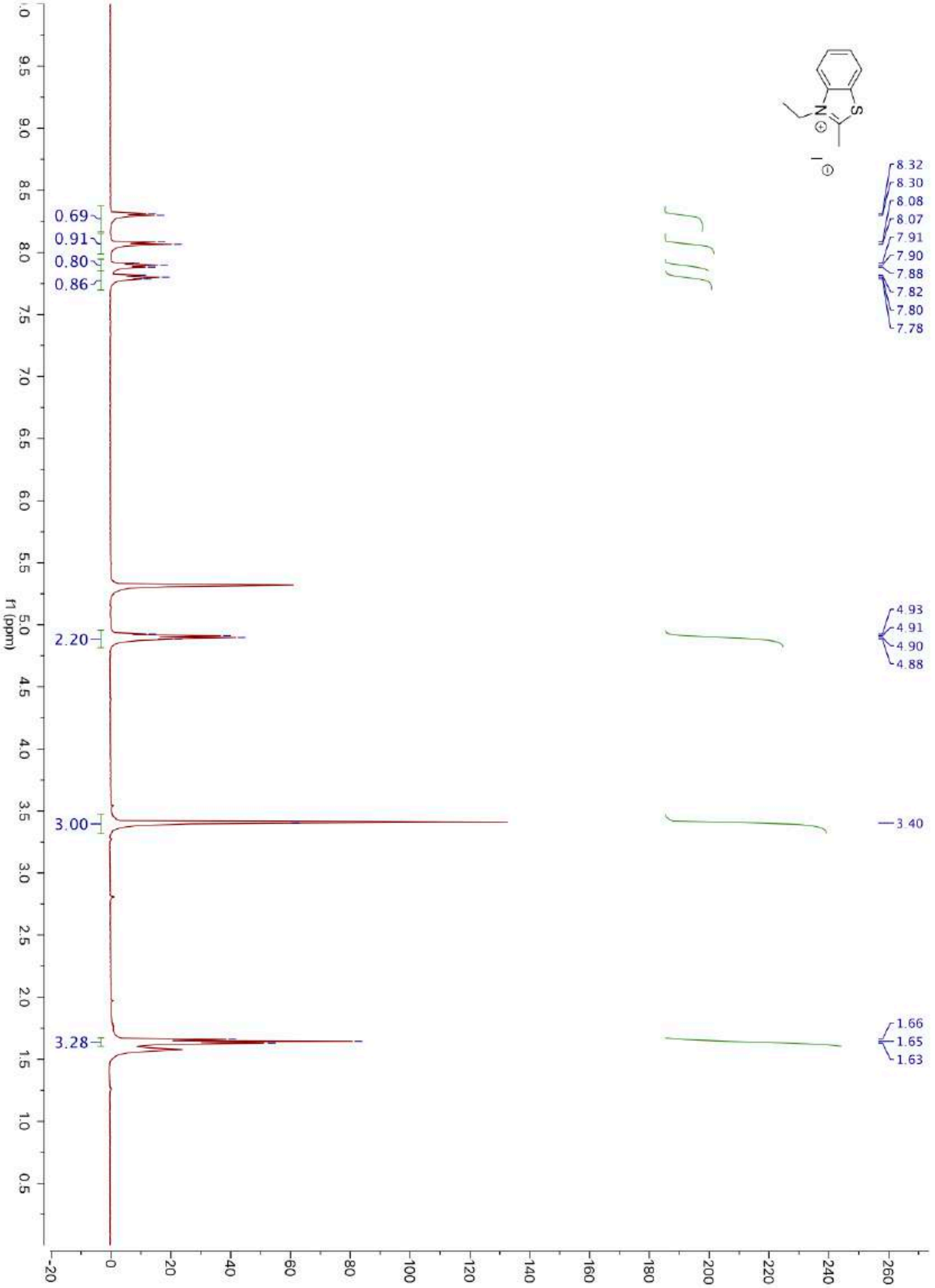

8.32
8.30
8.08
8.07
7.91
7.90
7.88
7.82
7.80
7.78
4.93
4.91
4.90
4.88
3.40
1.66
1.65
1.63
0.69
0.91
0.80
0.86
2.20
3.00
3.28
f1 (ppm)

2a

^{13}C NMR

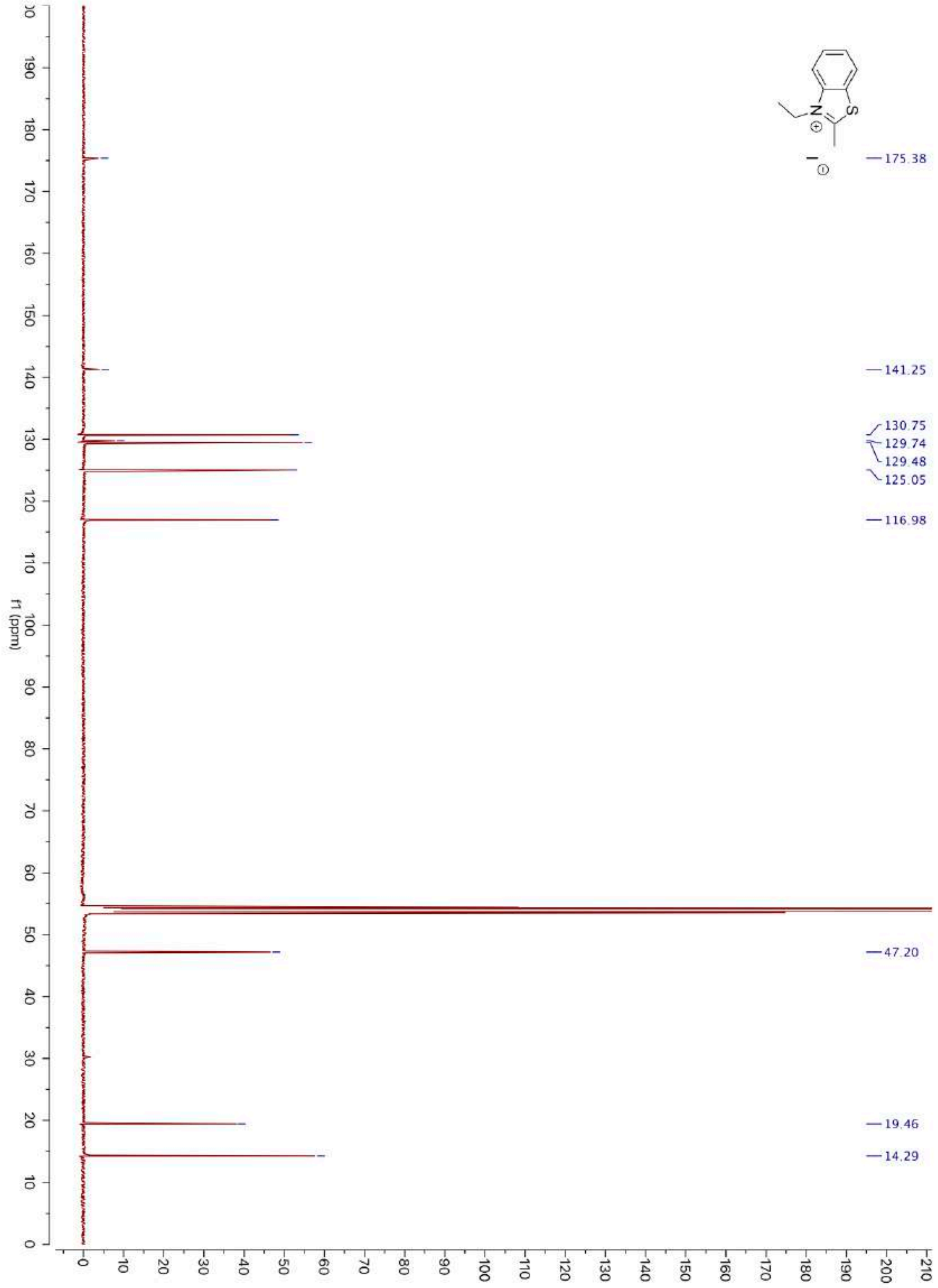
175.38
141.25
130.75
129.74
129.48
125.05
116.98
47.20
19.46
14.29
f1 (ppm)
S
N

2b

^{1}H NMR

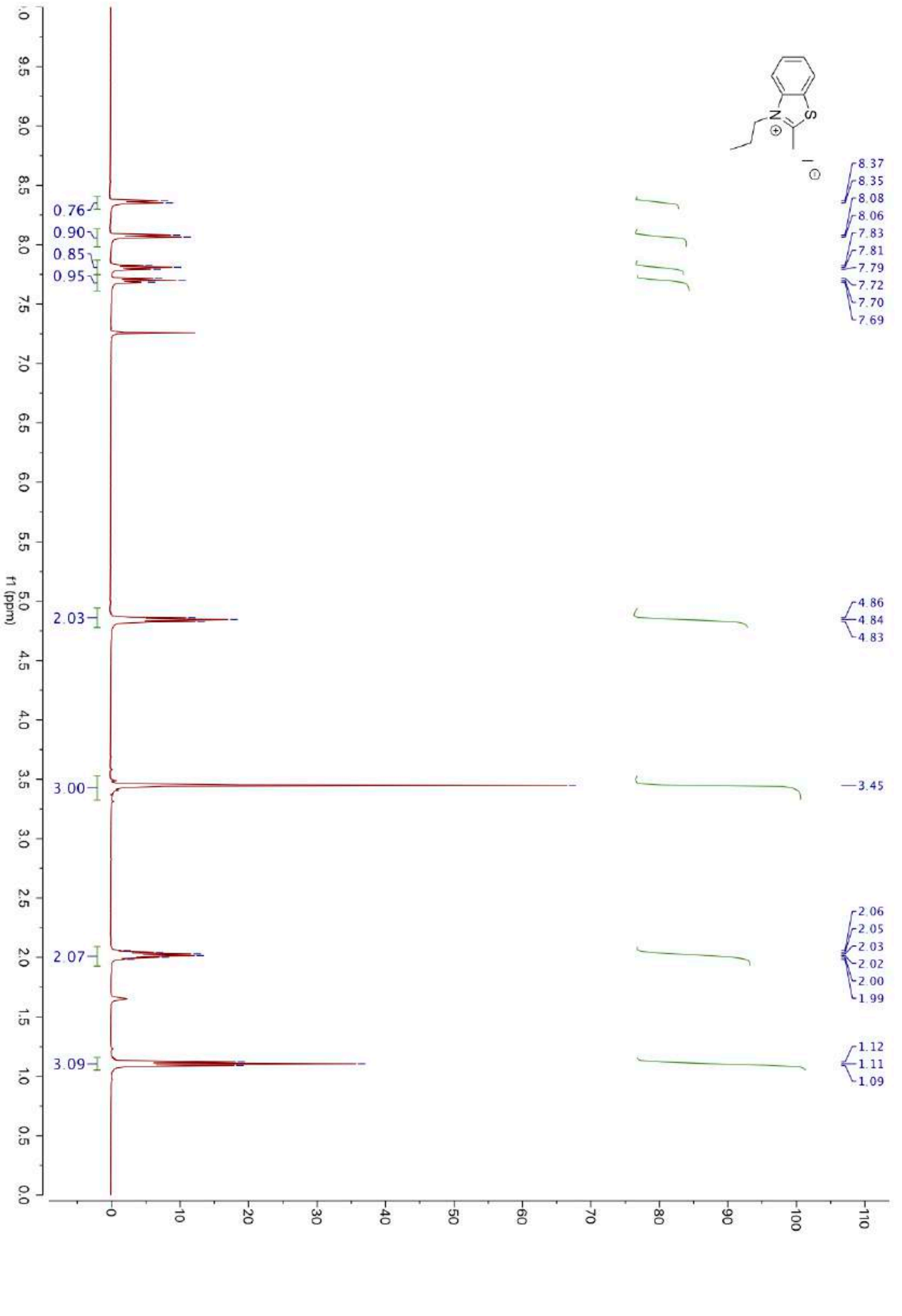

8.37
8.35
8.08
8.06
7.83
7.81
7.79
7.72
7.70
7.69
4.86
4.84
4.83
3.45
2.06
2.05
2.03
2.02
2.00
1.99
1.12
1.11
1.09
0.76
0.90
0.85
0.95
2.03
3.00
2.07
3.09
f1 (ppm)

2b
^{13}C NMR

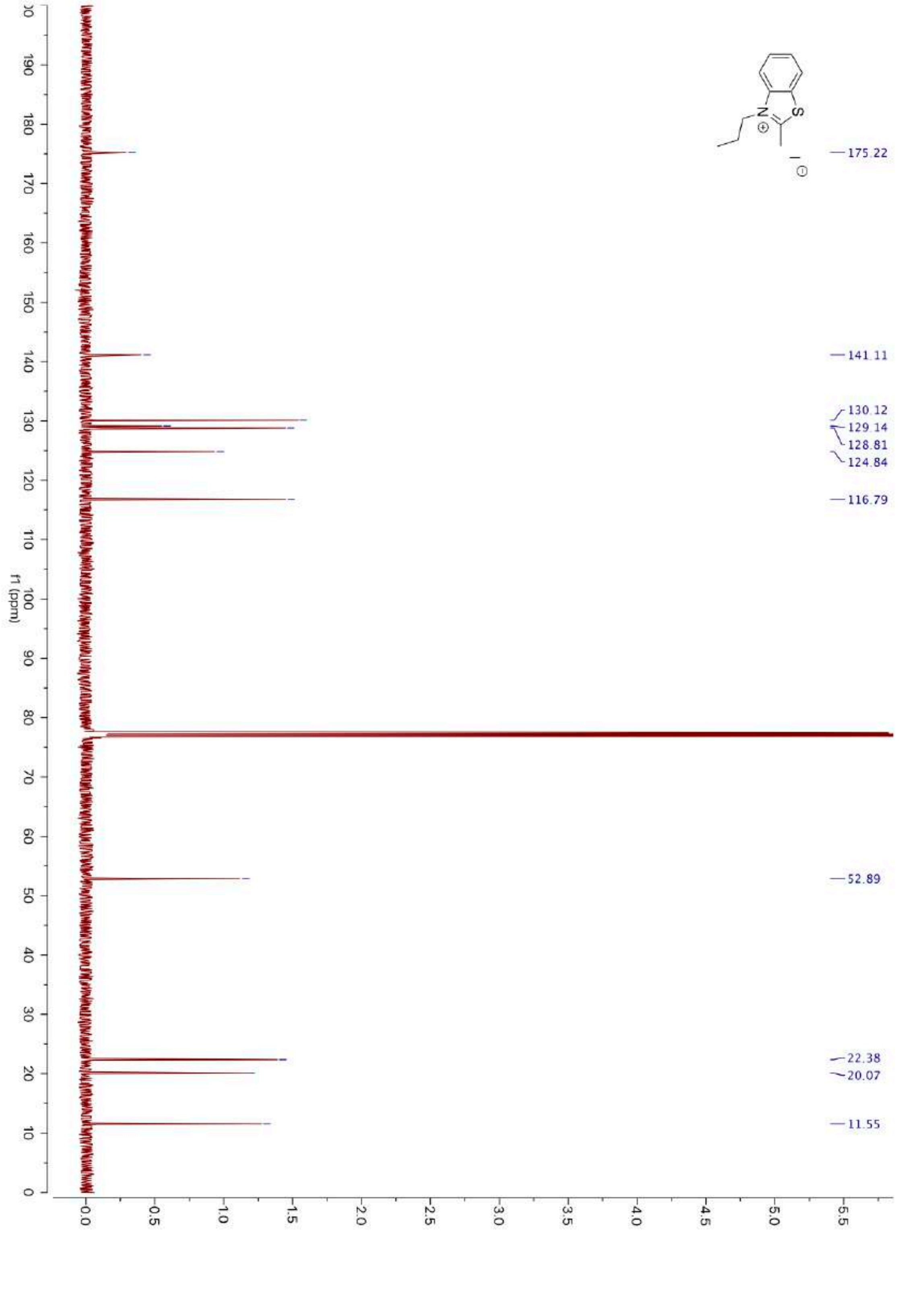

175.22
141.11
130.12
129.14
128.81
124.84
116.79
52.89
22.38
20.07
11.55
f1 (ppm)

2c
^{1}H NMR

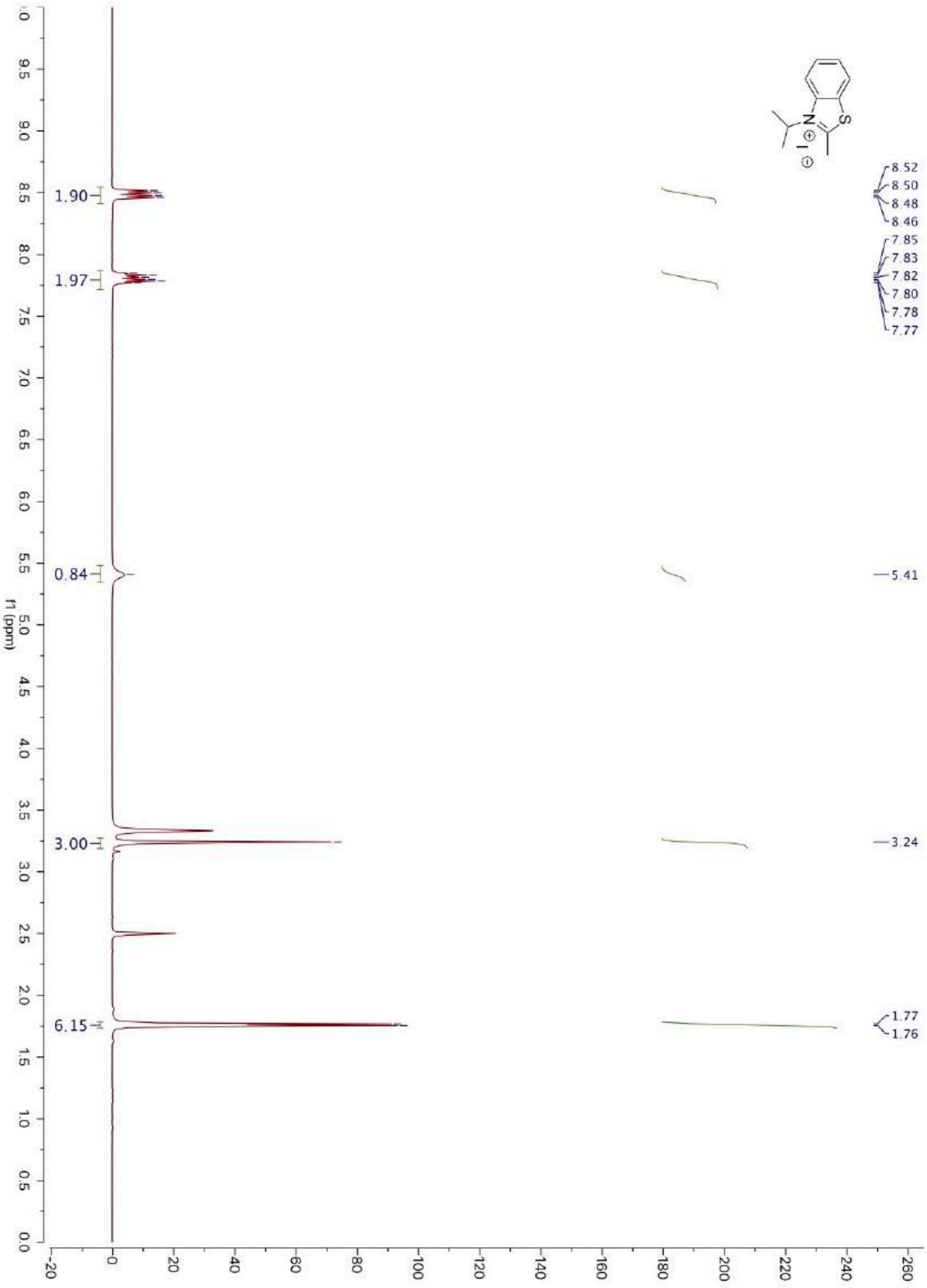

8.52
8.50
8.48
8.46
7.85
7.83
7.82
7.80
7.78
7.77
5.41
3.24
1.77
1.76
1.90
1.97
0.84
3.00
6.15
f1 (ppm)
S
N
I

2c
^{13}C NMR

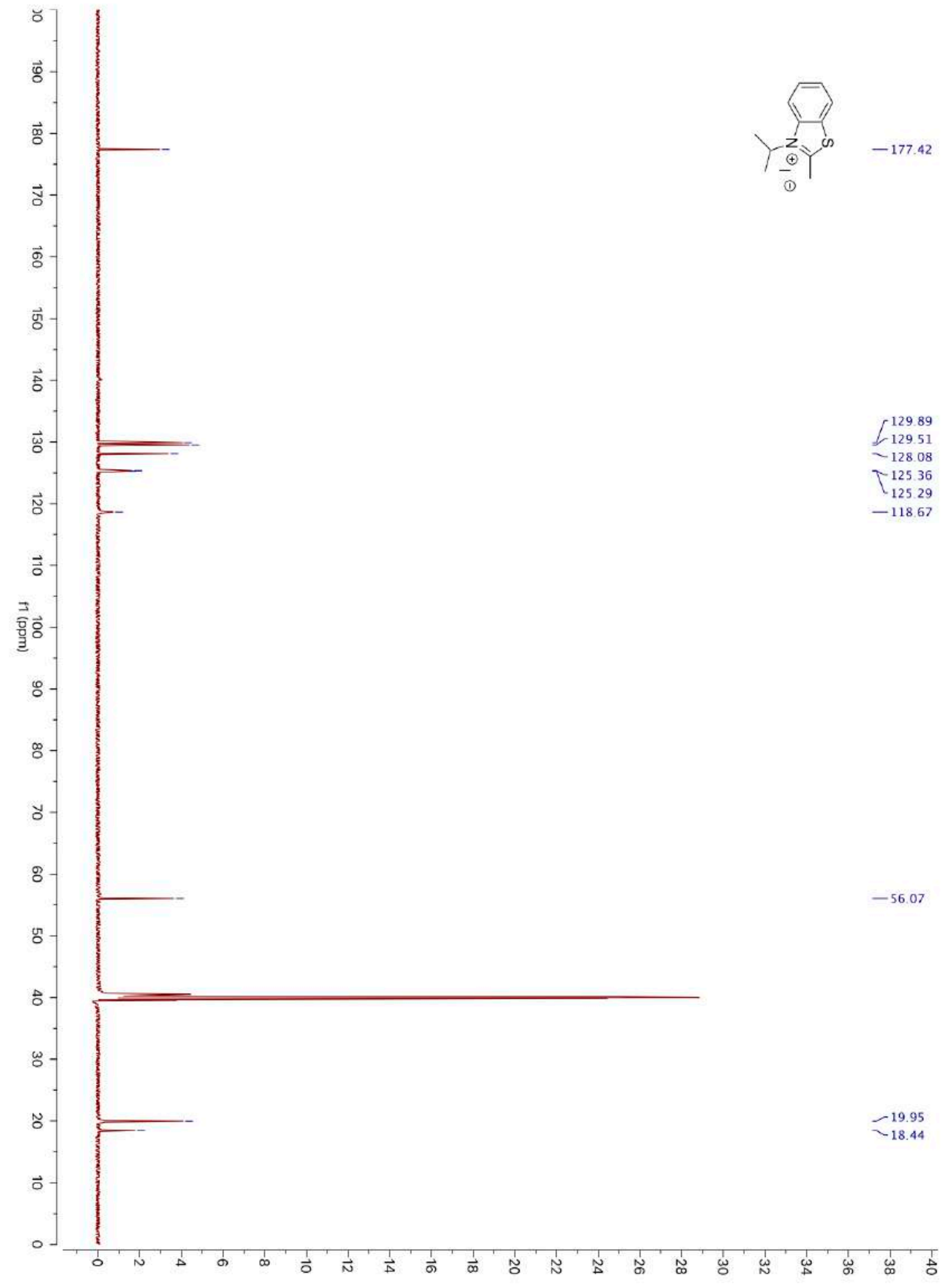
177.42
129.89
129.51
128.08
125.36
125.29
118.67
56.07
19.95
18.44
f1 (ppm)

2d
^{1}H NMR

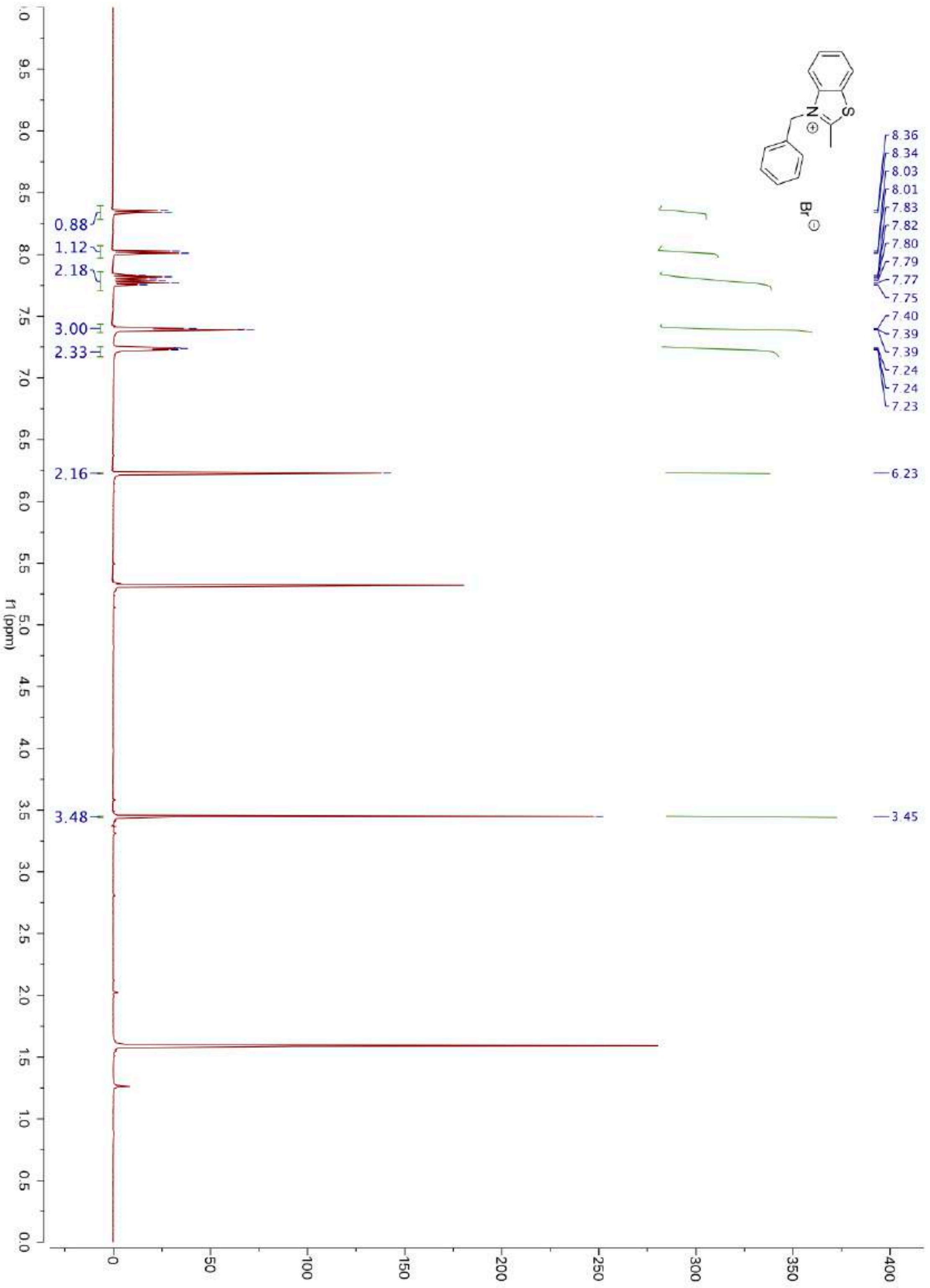

8.36
8.34
8.03
8.01
7.83
7.82
7.80
7.79
7.77
7.75
7.40
7.39
7.39
7.24
7.24
7.23
6.23
3.45
0.88
1.12
2.18
3.00
2.33
2.16
3.48
f1 (ppm)
Br
N
S

2d

^{13}C NMR

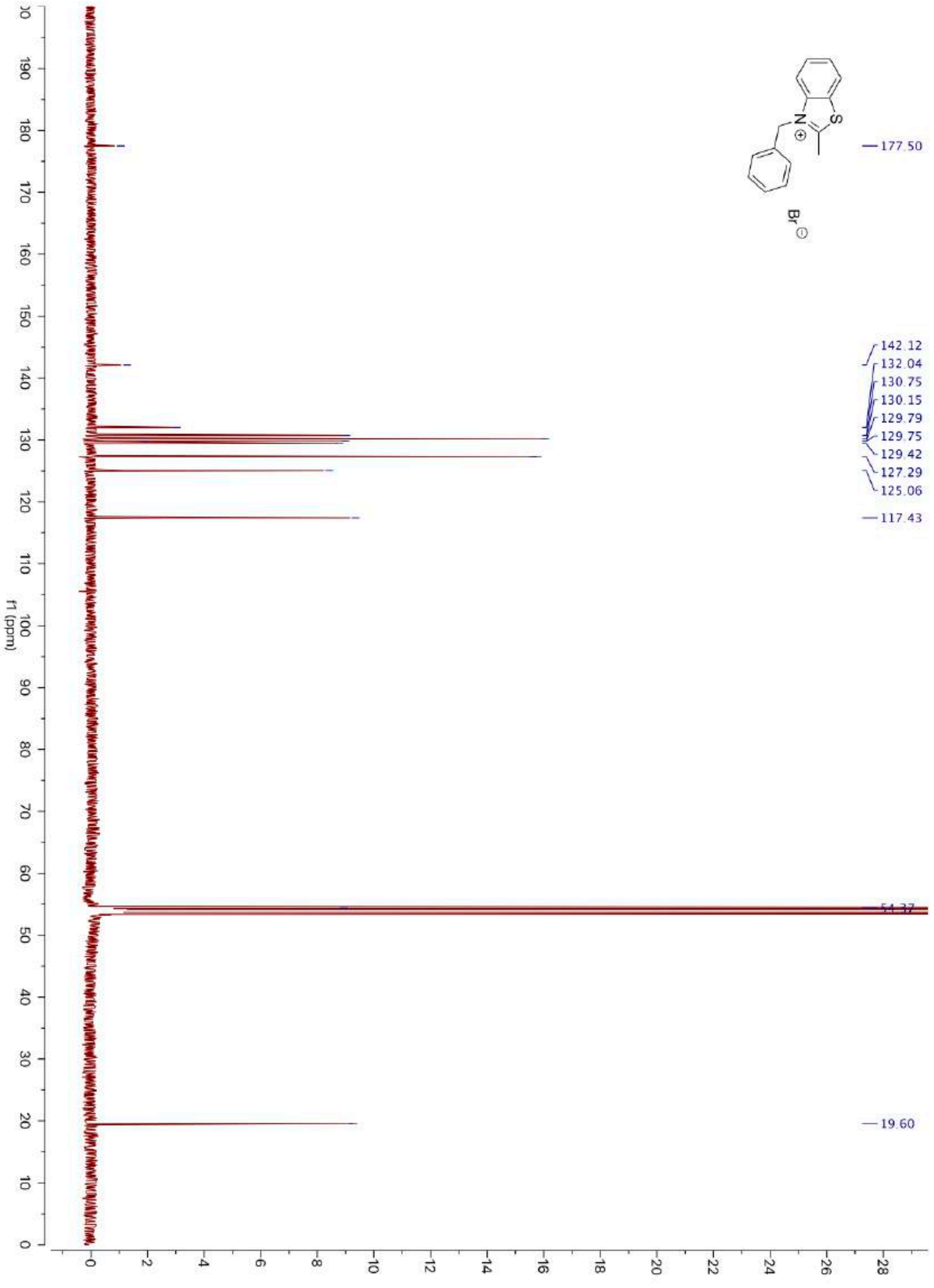

177.50
142.12
132.04
130.75
130.15
129.79
129.75
129.42
127.29
125.06
117.43
54.37
19.60
f1 (ppm)

2e

^{1}H NMR

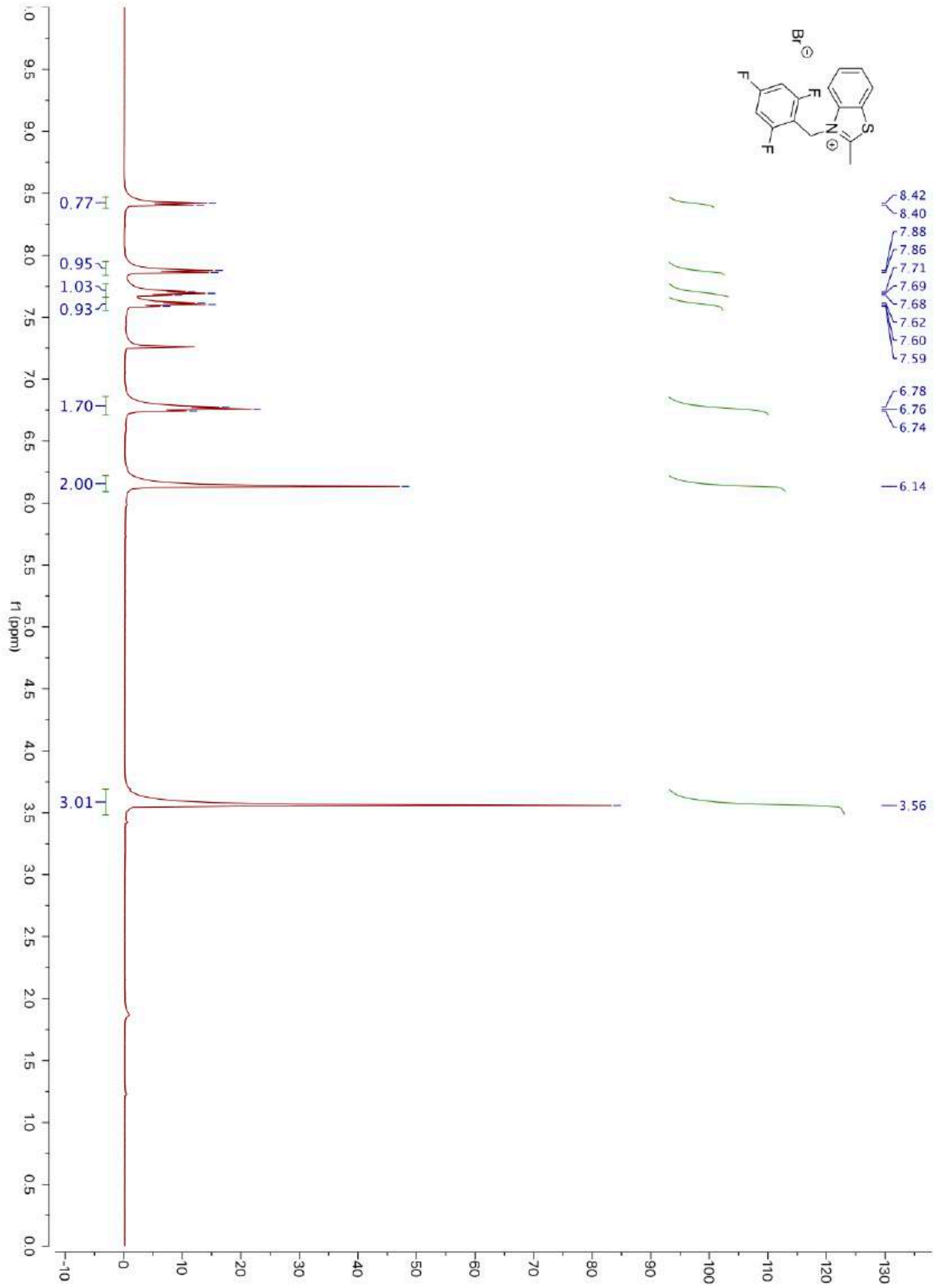

8.42
8.40
7.88
7.86
7.71
7.69
7.68
7.62
7.60
7.59
6.78
6.76
6.74
6.14
3.56
0.77
0.95
1.03
0.93
1.70
2.00
3.01
f1 (ppm)
Br
F
N
S

2e
^{13}C NMR

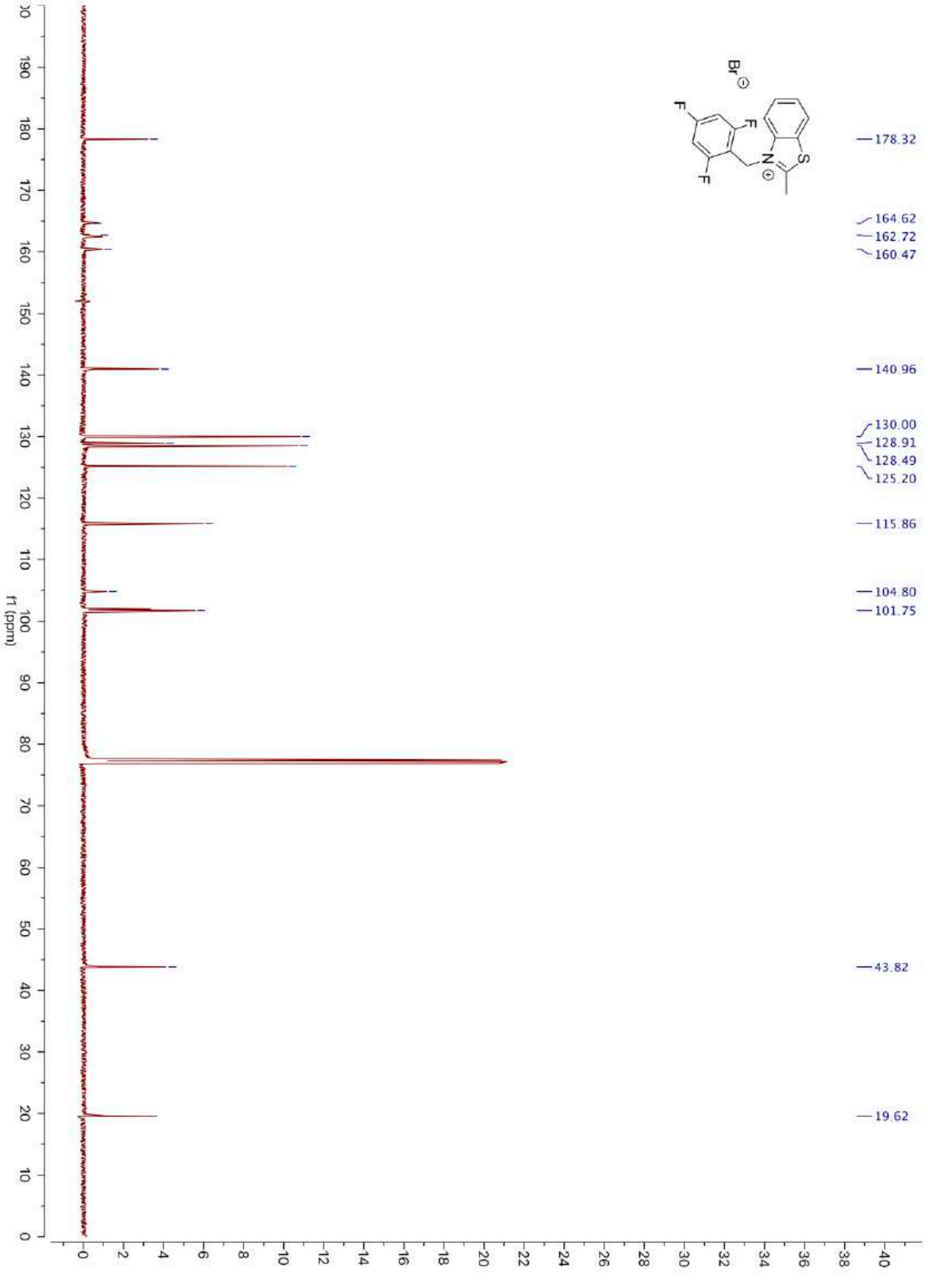

178.32
164.62
162.72
160.47
140.96
130.00
128.91
128.49
125.20
115.86
104.80
101.75
43.82
19.62
f1 (ppm)
Br
N
S
F
F
F

2f

^{1}H NMR

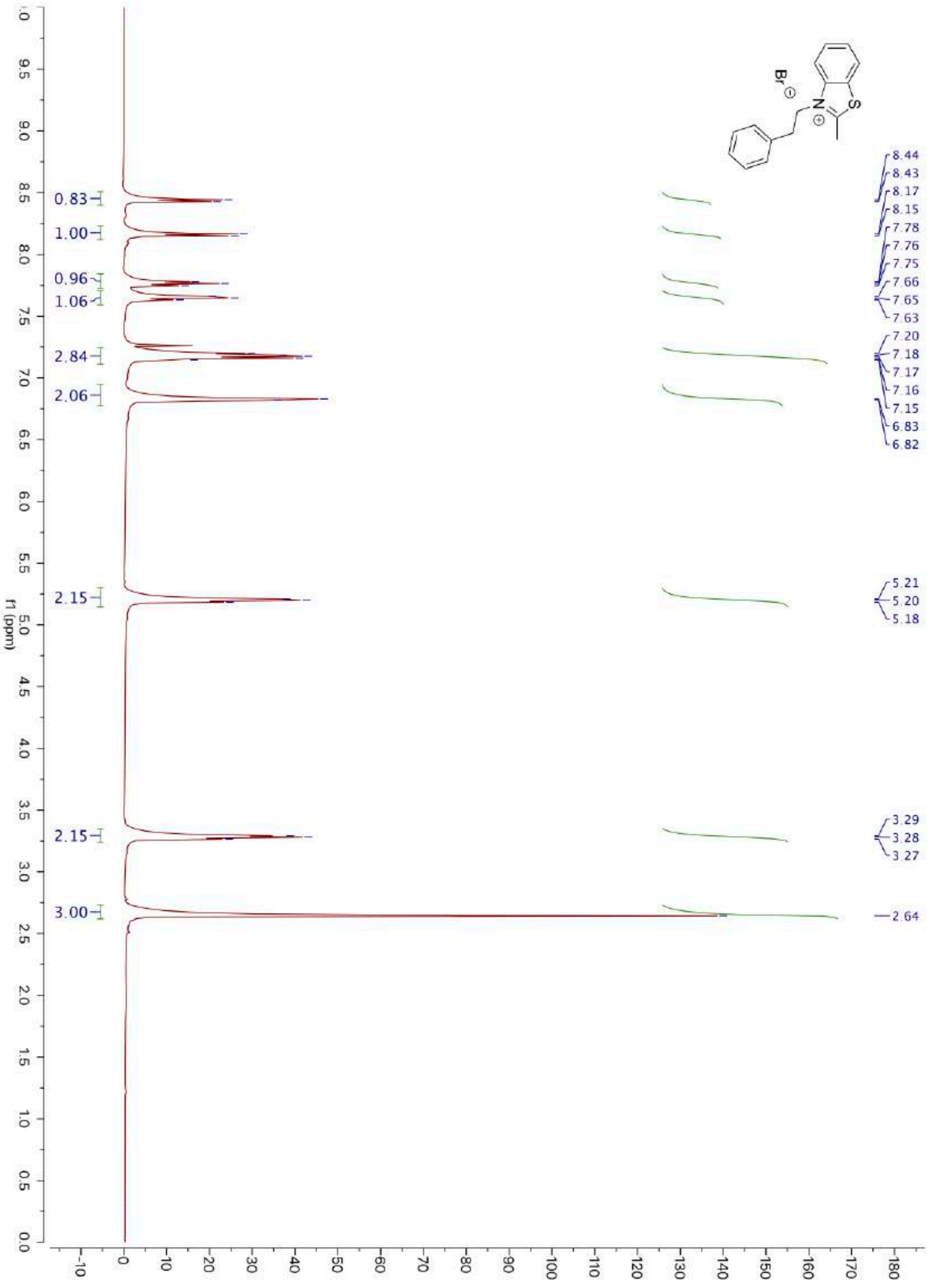
Br⁻
N⁺
S
8.44
8.43
8.17
8.15
7.78
7.76
7.75
7.66
7.65
7.63
7.20
7.18
7.17
7.16
7.15
6.83
6.82
5.21
5.20
5.18
3.29
3.28
3.27
2.64
0.83
1.00
0.96
1.06
2.84
2.06
2.15
2.15
3.00
f1 (ppm)

2f

^{13}C NMR

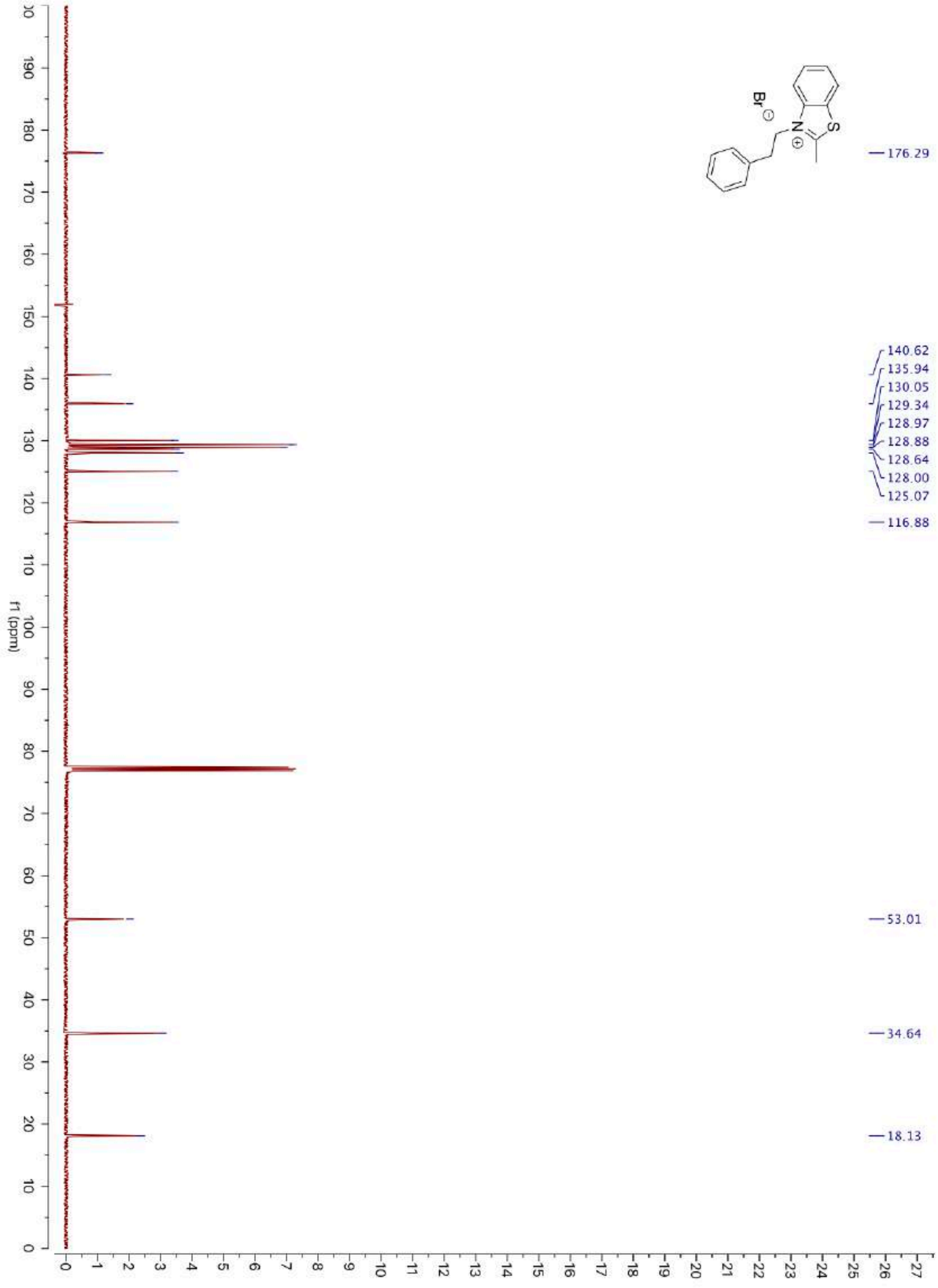

Br
N
S
176.29
140.62
135.94
130.05
129.34
128.97
128.88
128.64
128.00
125.07
116.88
53.01
34.64
18.13
f1 (ppm)

2g
^{1}H NMR

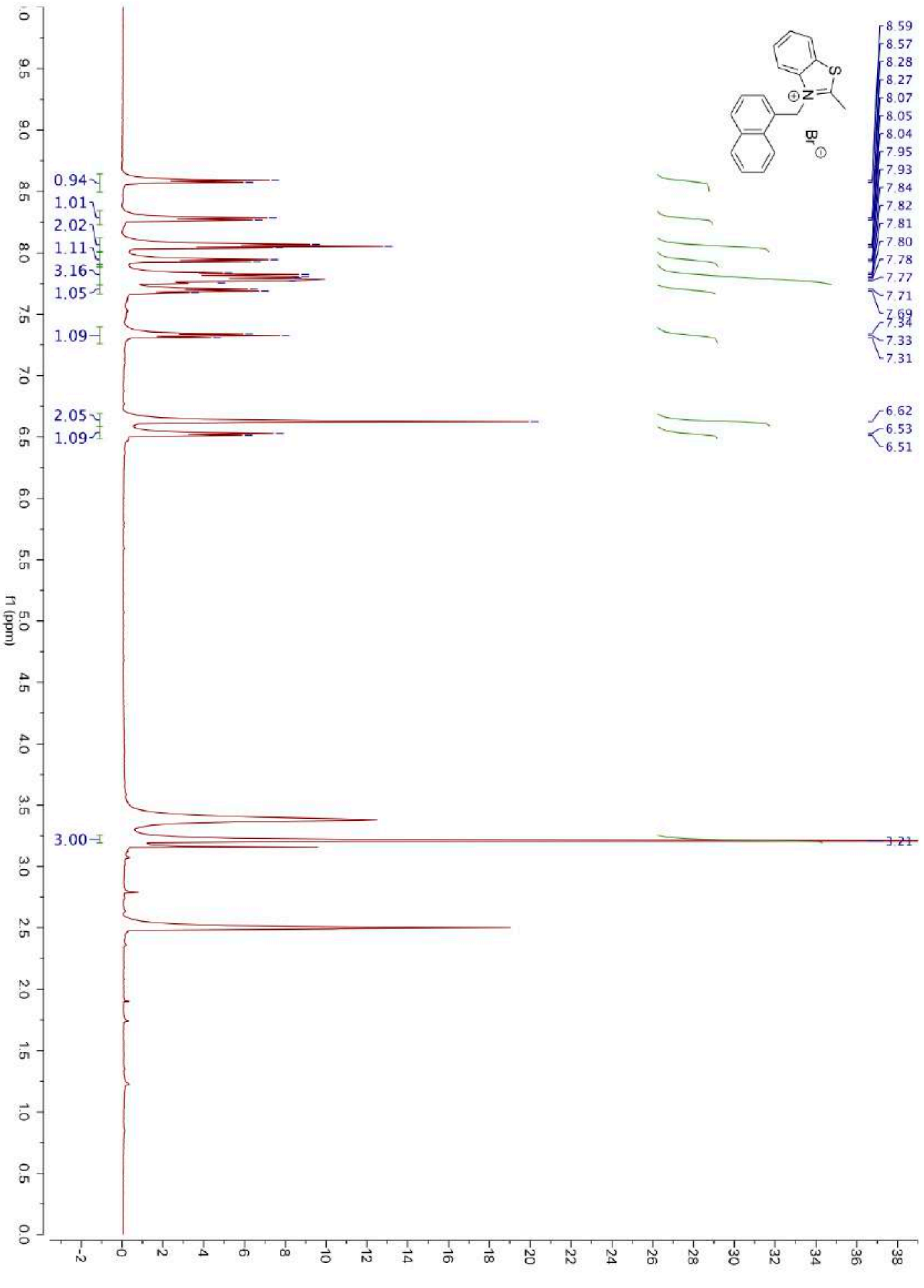

2g
^{13}C NMR

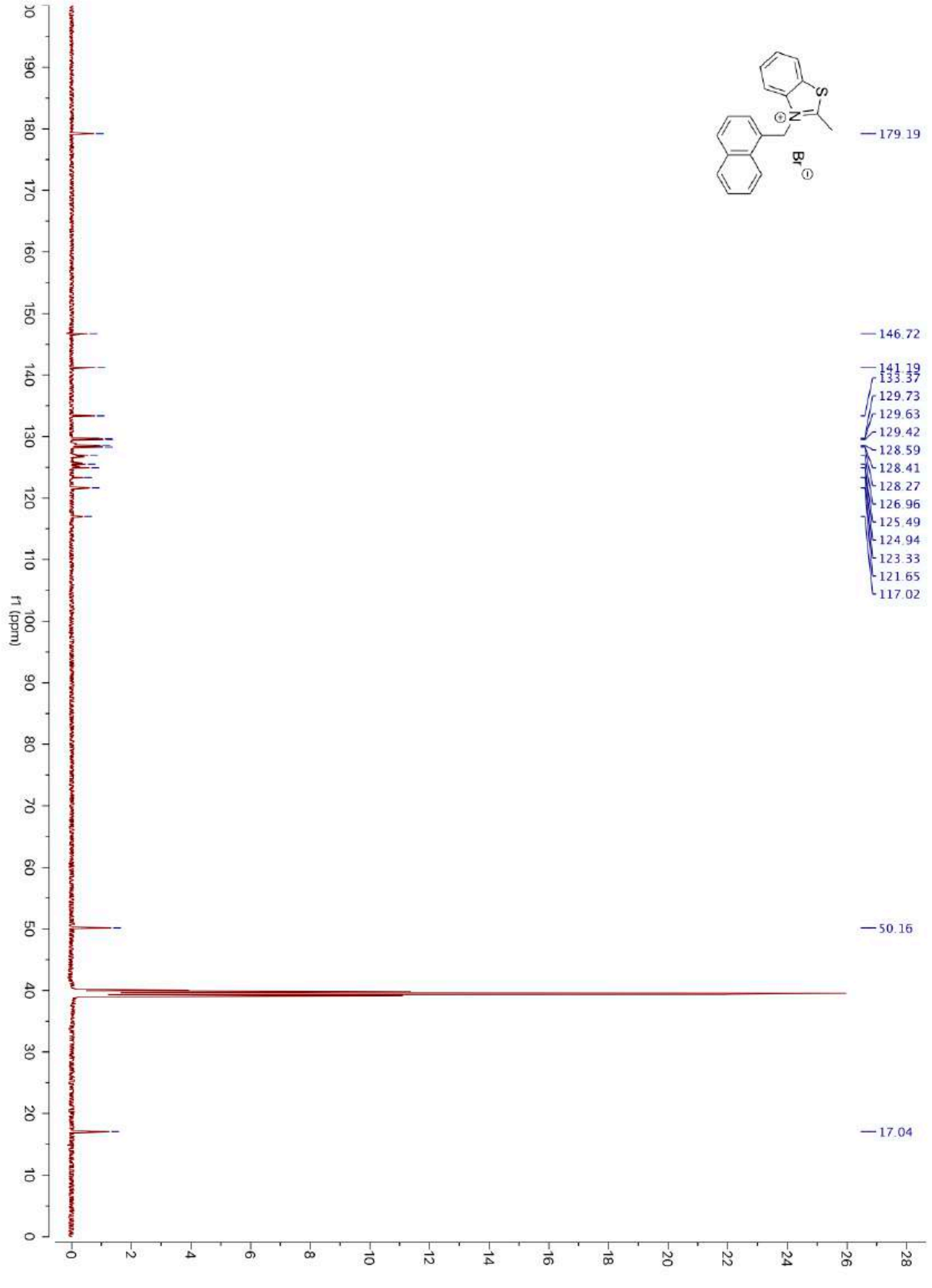
179.19
146.72
141.19
133.37
129.73
129.63
129.42
128.59
128.41
128.27
126.96
125.49
124.94
123.33
121.65
117.02
50.16
17.04
f1 (ppm)
Br

2h
^{1}H NMR

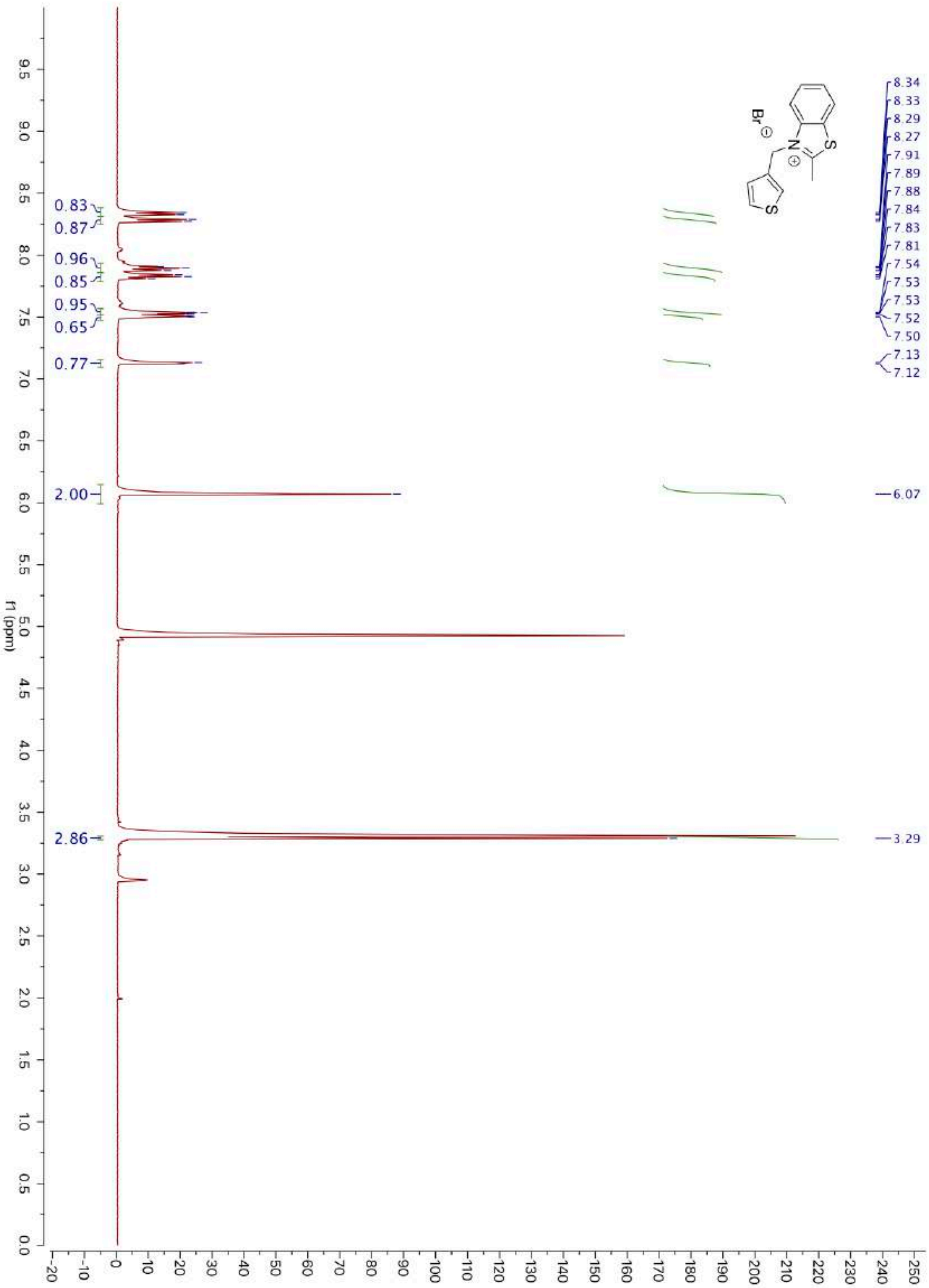

8.34
8.33
8.29
8.27
7.91
7.89
7.88
7.84
7.83
7.81
7.54
7.53
7.53
7.52
7.50
7.13
7.12
6.07
3.29
0.83
0.87
0.96
0.85
0.95
0.65
0.77
2.00
2.86
Br
N
S
S
f1 (ppm)

2g
^{13}C NMR

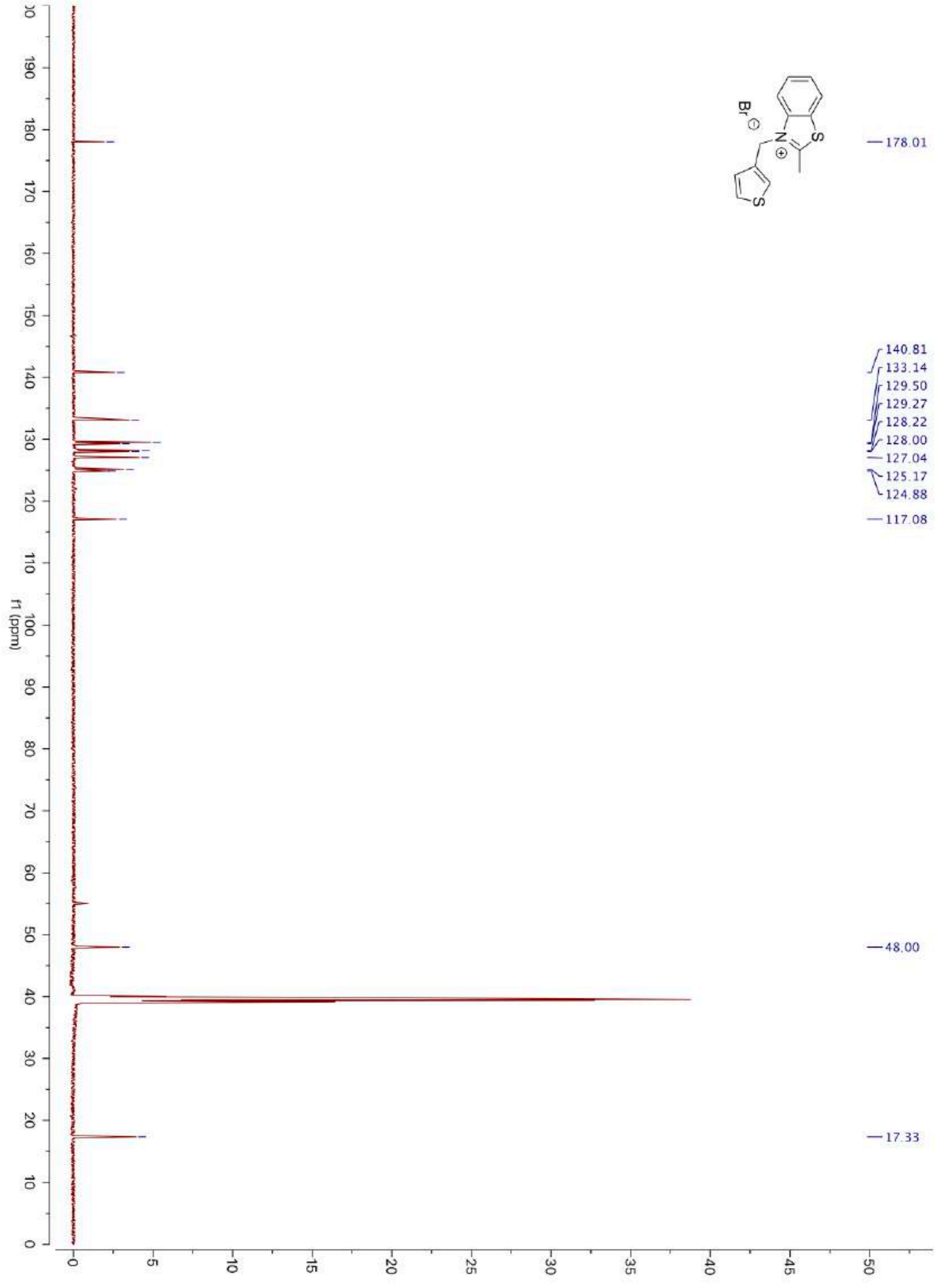

Br
178.01
140.81
133.14
129.50
129.27
128.22
128.00
127.04
125.17
124.88
117.08
48.00
17.33
f1 (ppm)

2i

^{1}H NMR

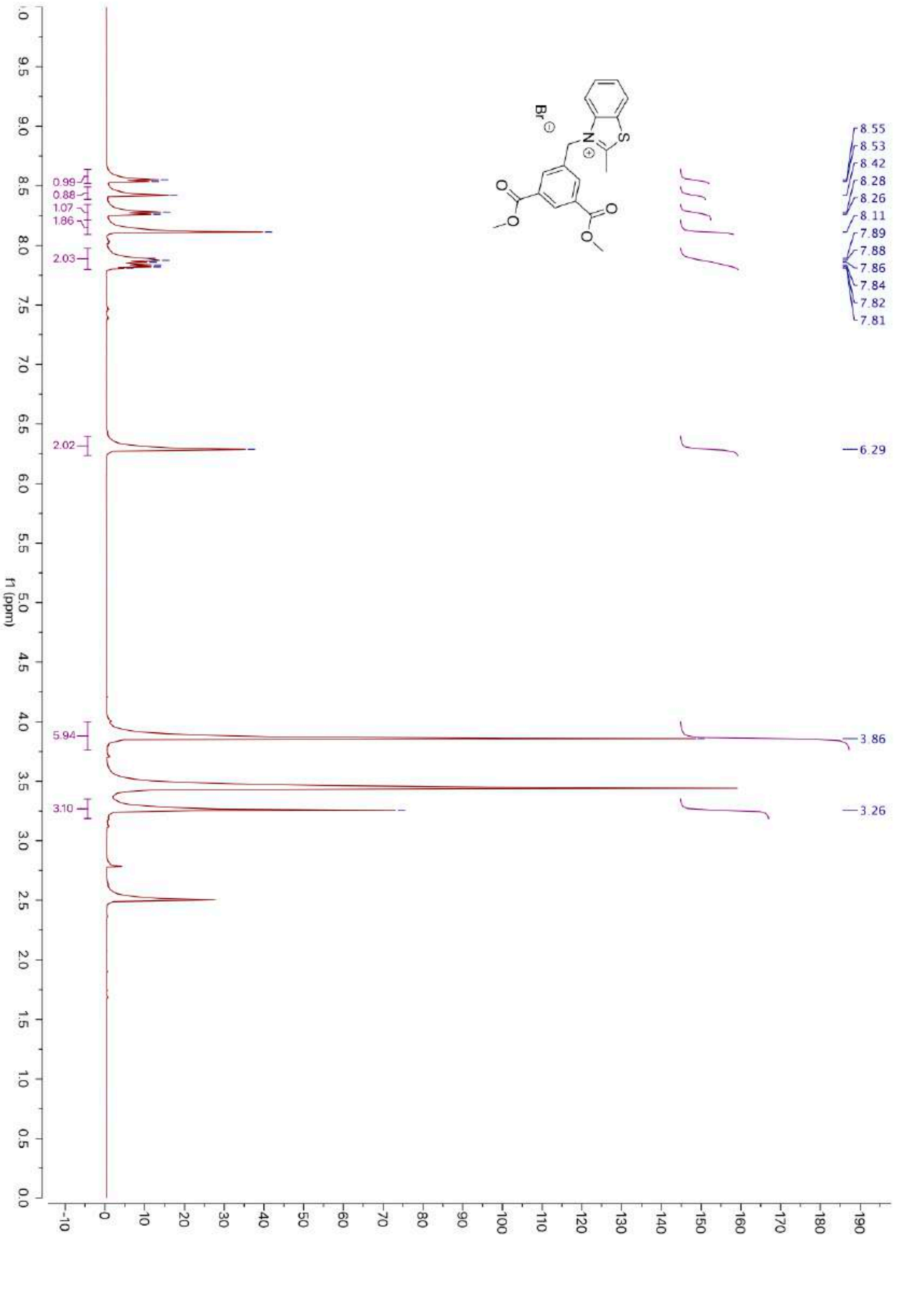

8.55
8.53
8.42
8.28
8.26
8.11
7.89
7.88
7.86
7.84
7.82
7.81
6.29
3.86
3.26
0.99
0.88
1.07
1.86
2.03
2.02
5.94
3.10
f1 (ppm)
Br
N
S

2i
^{13}C NMR

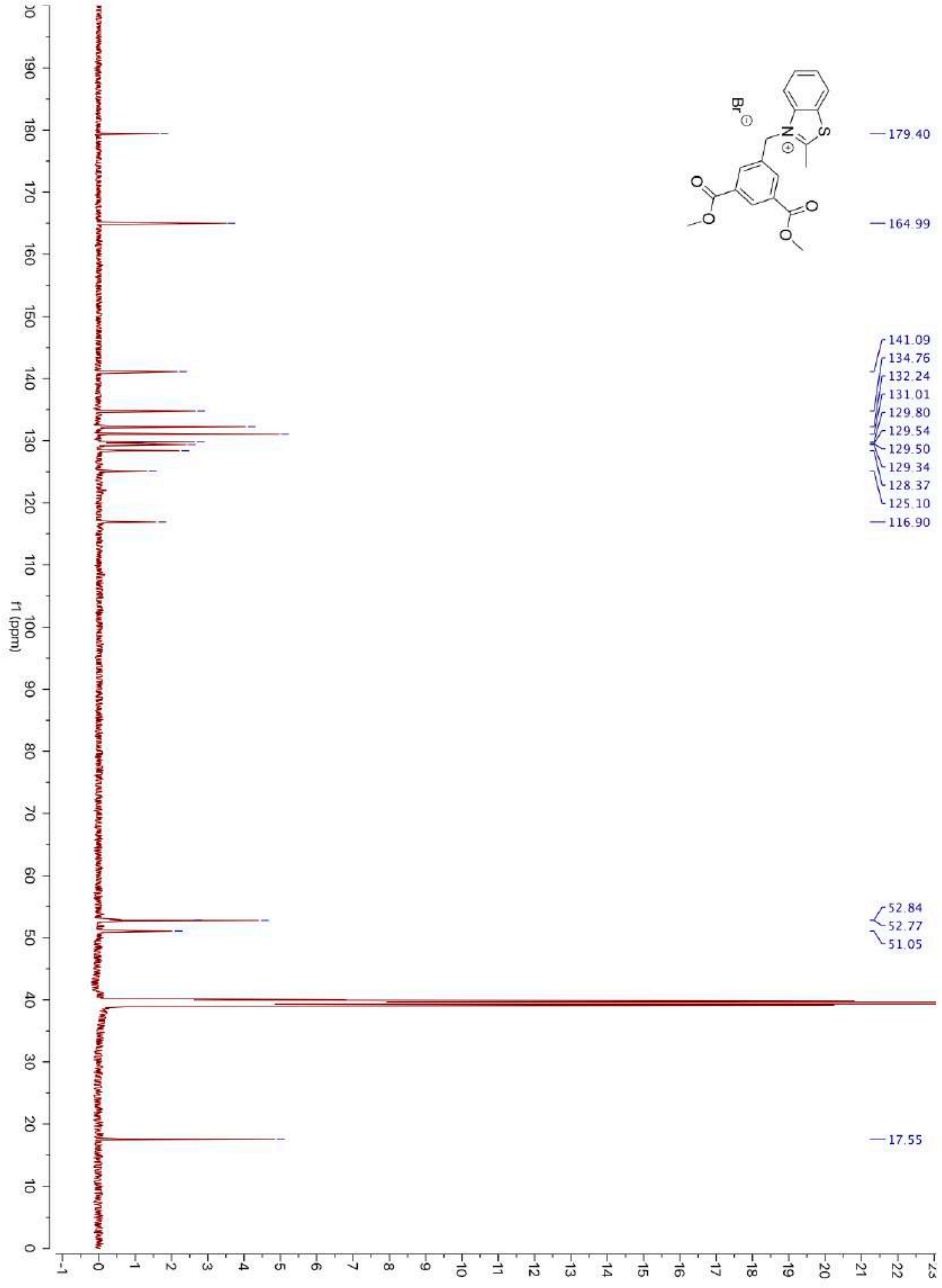

Br
N
S
179.40
164.99
141.09
134.76
132.24
131.01
129.80
129.54
129.50
129.34
128.37
125.10
116.90
52.84
52.77
51.05
17.55
f1 (ppm)

2j
^{1}H NMR

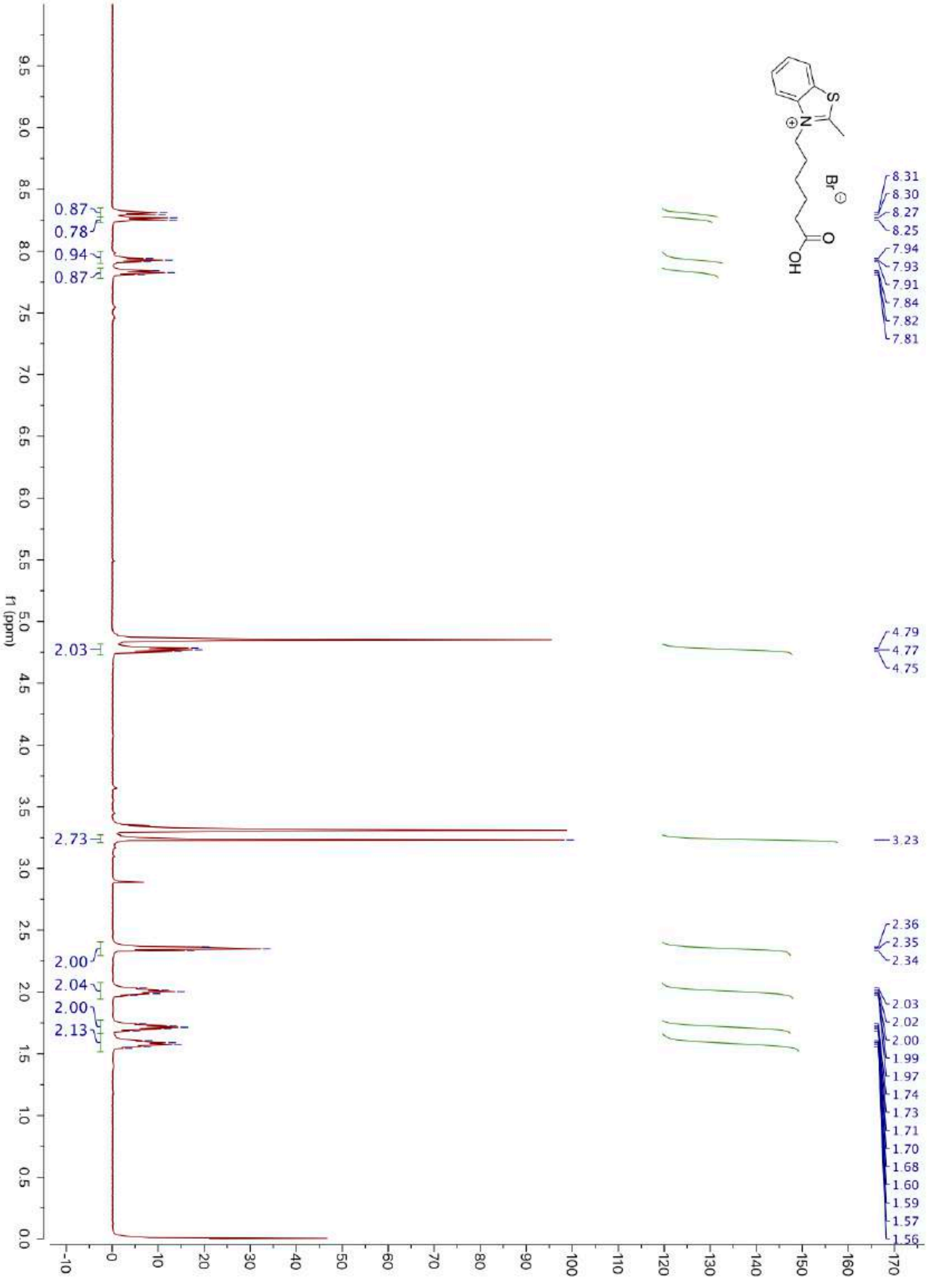
f1 (ppm)
Br
OH
O
N
S
8.31
8.30
8.27
8.25
7.94
7.93
7.91
7.84
7.82
7.81
4.79
4.77
4.75
3.23
2.36
2.35
2.34
2.03
2.02
2.00
1.99
1.97
1.74
1.73
1.71
1.70
1.68
1.60
1.59
1.57
1.56
0.87
0.78
0.94
0.87
2.03
2.73
2.00
2.04
2.00
2.13

2j
^{13}C NMR

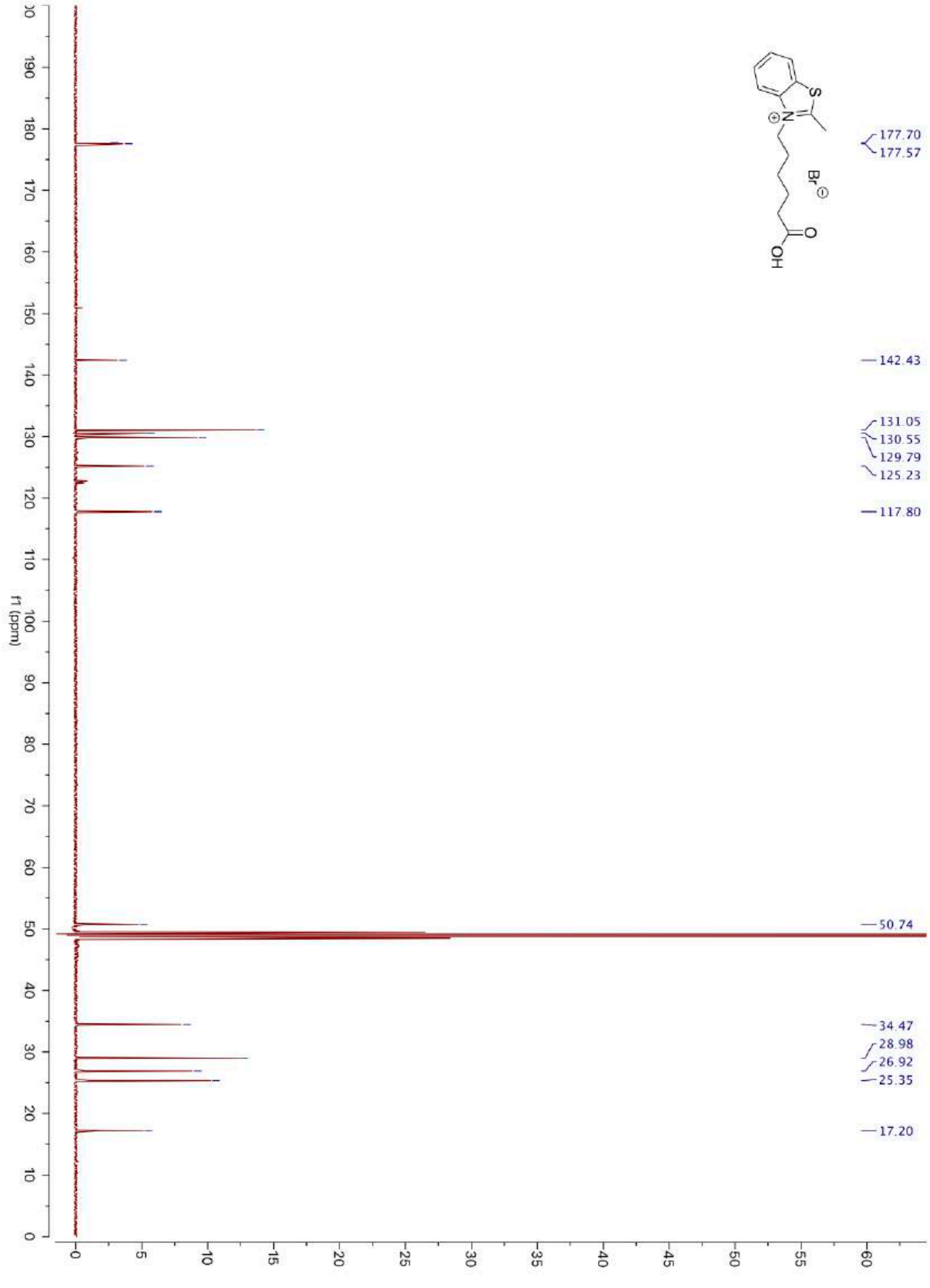
177.70
177.57
142.43
131.05
130.55
129.79
125.23
117.80
50.74
34.47
28.98
26.92
25.35
17.20
f1 (ppm)
S
N
Br
OH
O

3a

^{1}H NMR

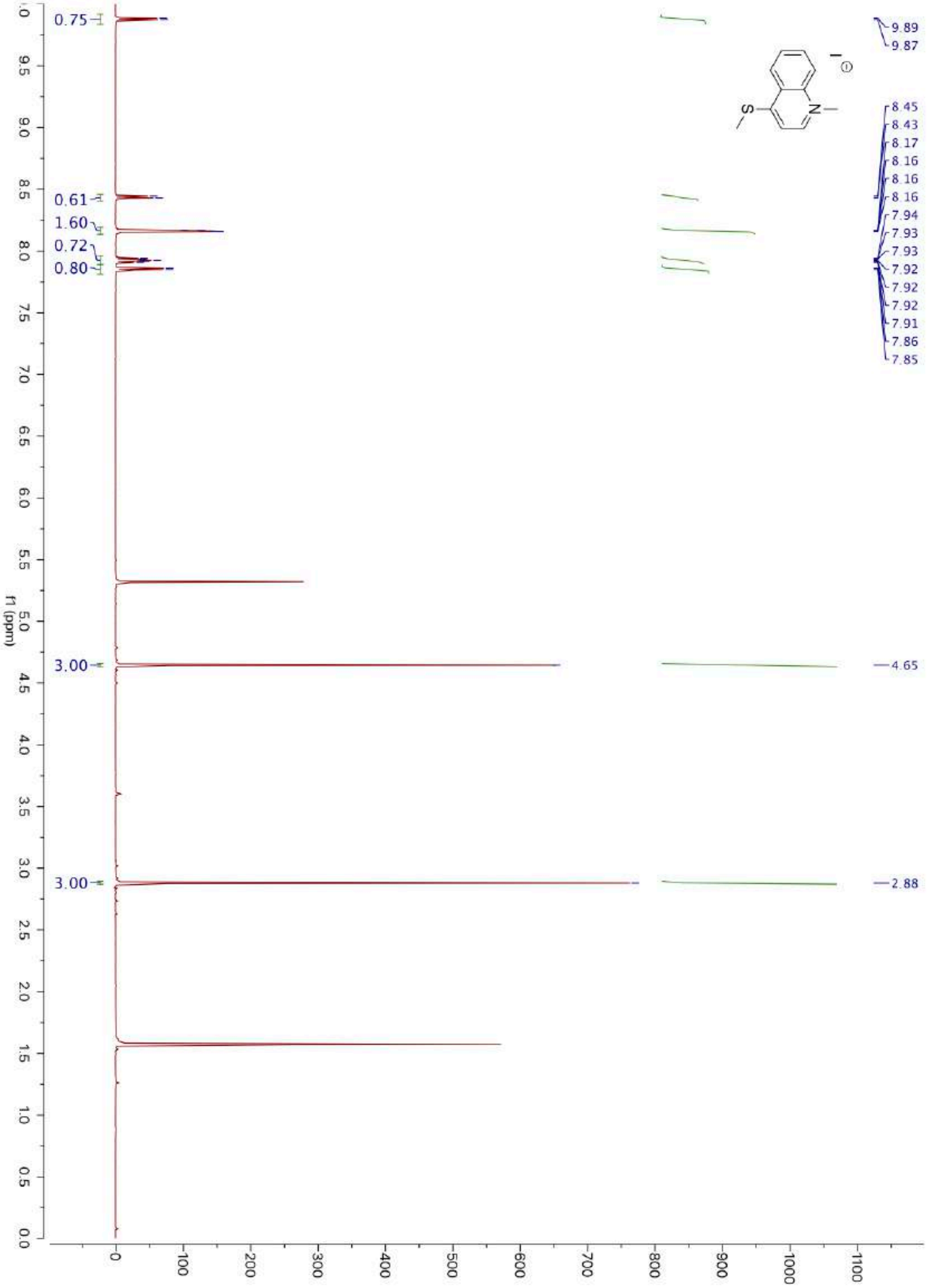
9.89
9.87
8.45
8.43
8.17
8.16
8.16
8.16
7.94
7.93
7.93
7.92
7.92
7.92
7.91
7.86
7.85
4.65
2.88
0.75
0.61
1.60
0.72
0.80
3.00
3.00
f1 (ppm)
S
N
I

3a
^{13}C NMR

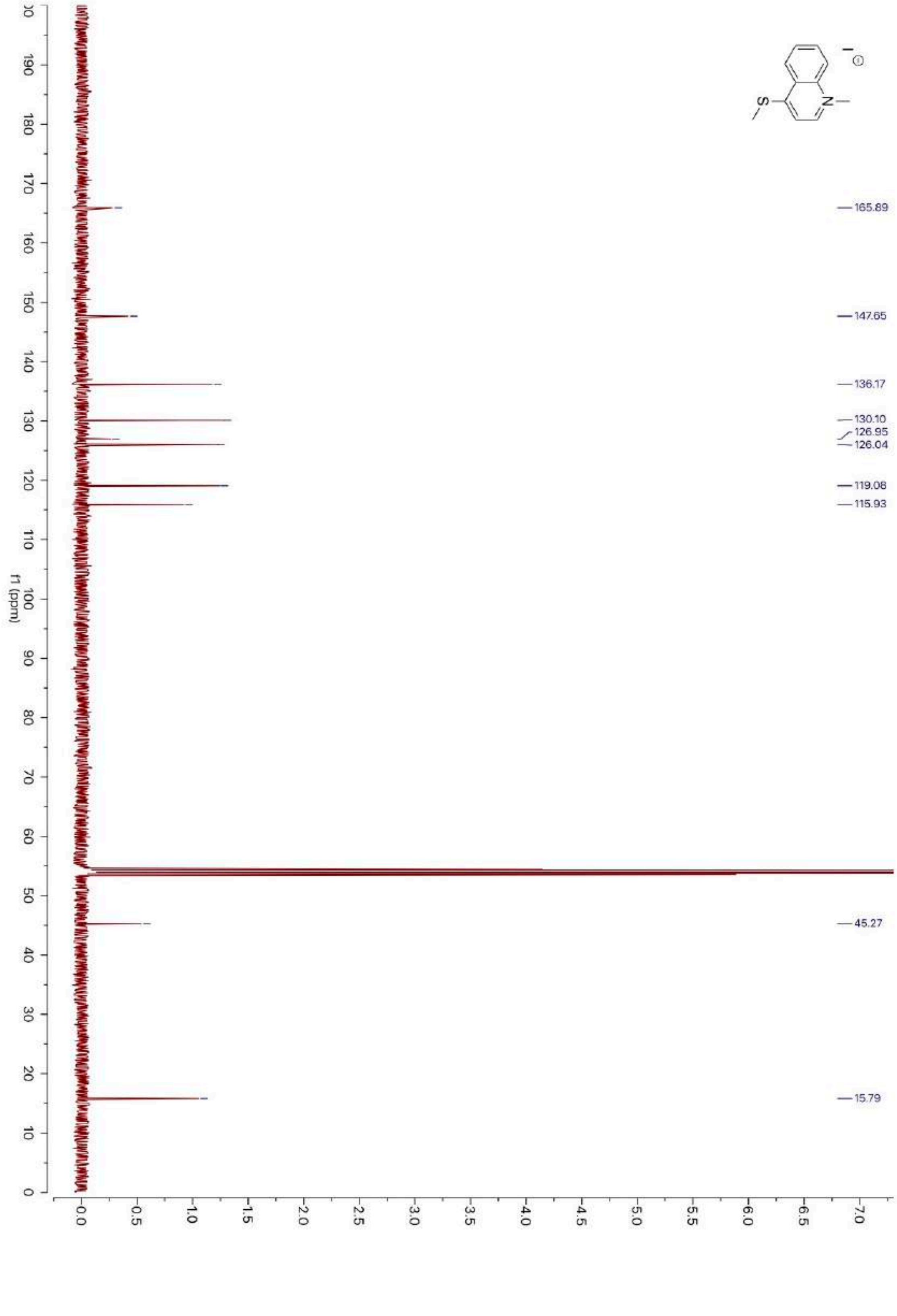

165.89
147.65
136.17
130.10
126.95
126.04
119.08
115.93
45.27
15.79
f1 (ppm)

3b

^{1}H NMR

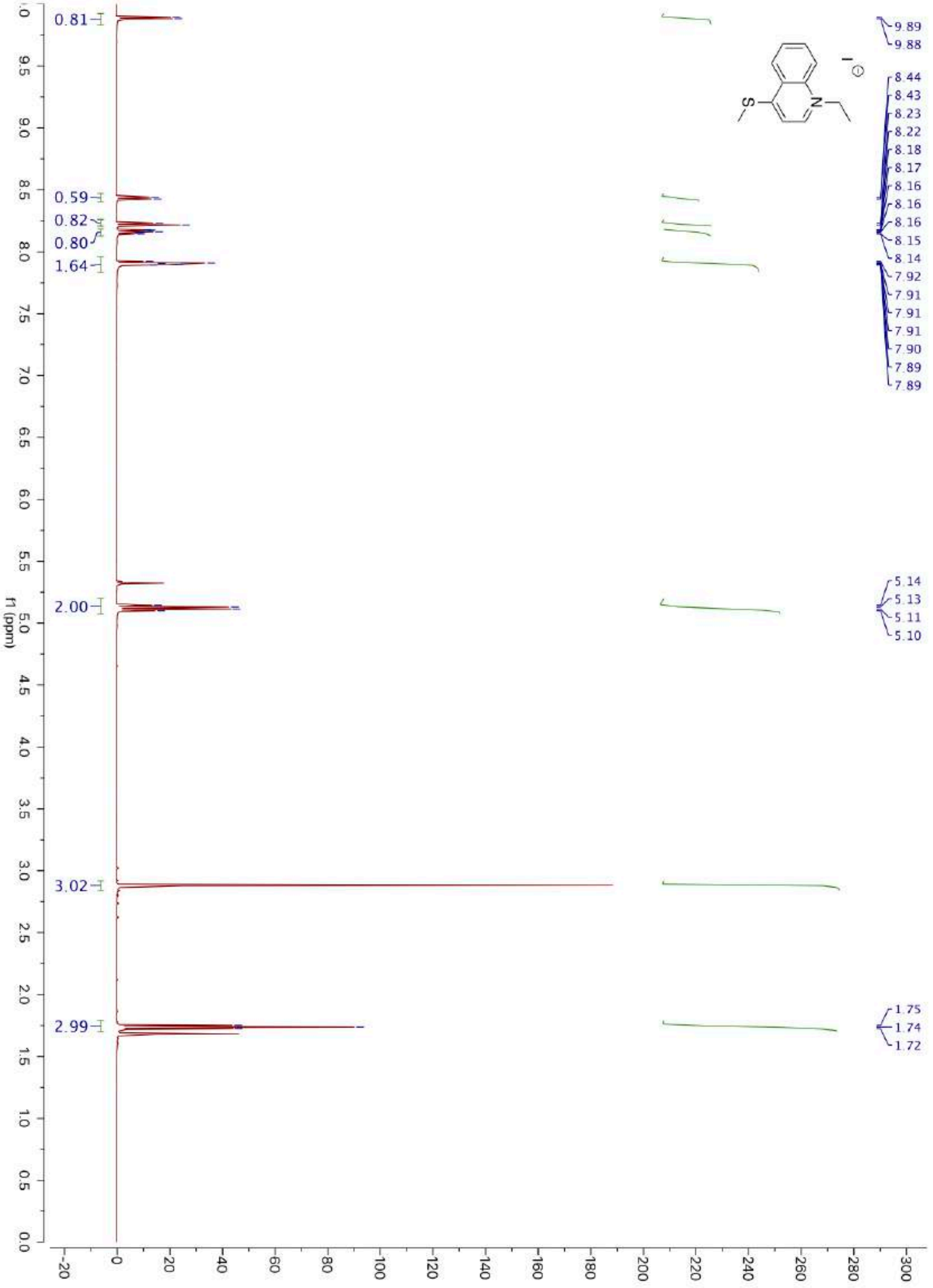

9.89
9.88
8.44
8.43
8.23
8.22
8.18
8.17
8.16
8.16
8.16
8.15
8.14
7.92
7.91
7.91
7.91
7.90
7.89
7.89
5.14
5.13
5.11
5.10
1.75
1.74
1.72
0.81
0.59
0.82
0.80
1.64
2.00
3.02
2.99
f1 (ppm)

3b
^{13}C NMR

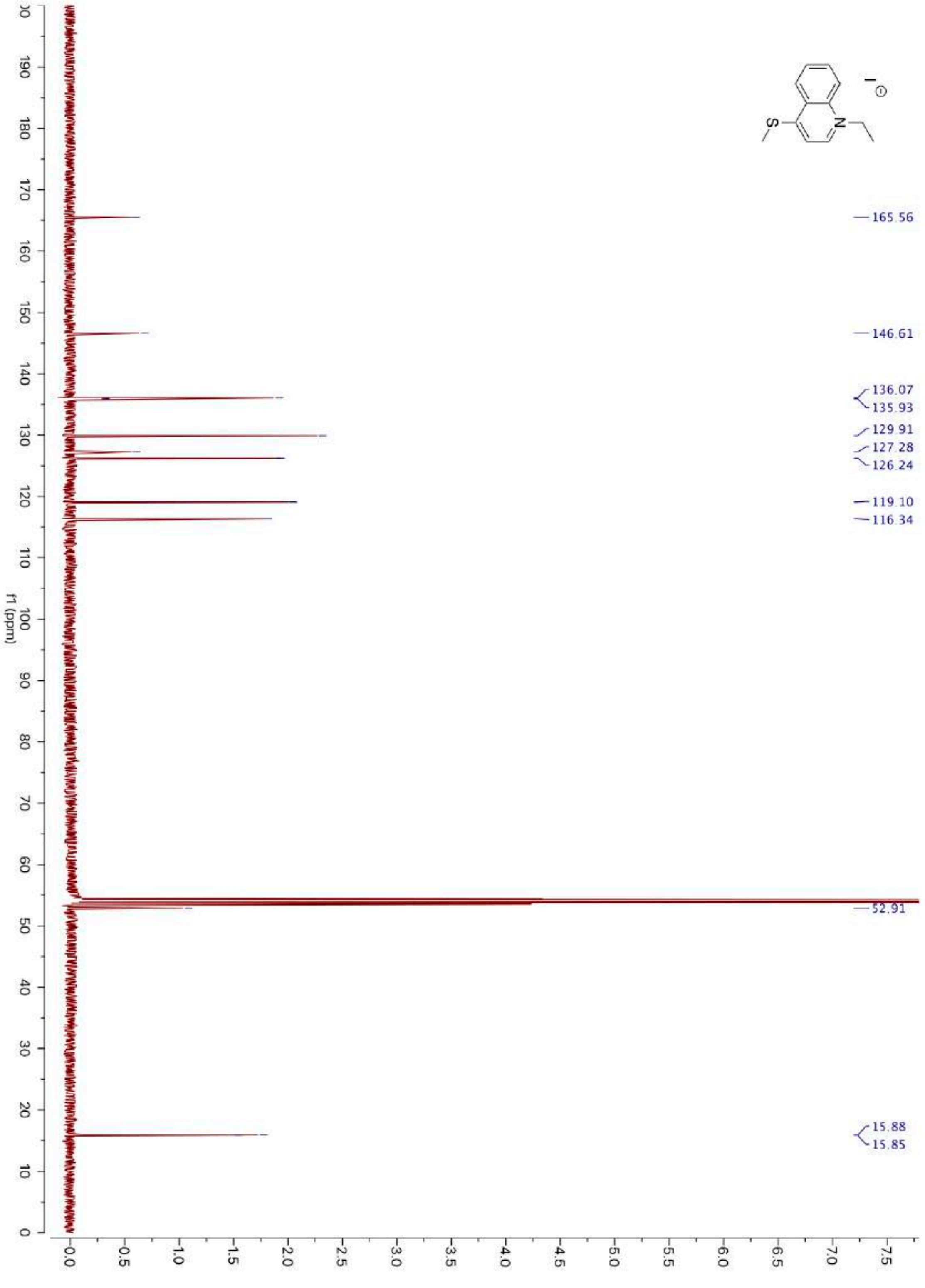

165.56
146.61
136.07
135.93
129.91
127.28
126.24
119.10
116.34
52.91
15.88
15.85
f1 (ppm)

3c
^{1}H NMR

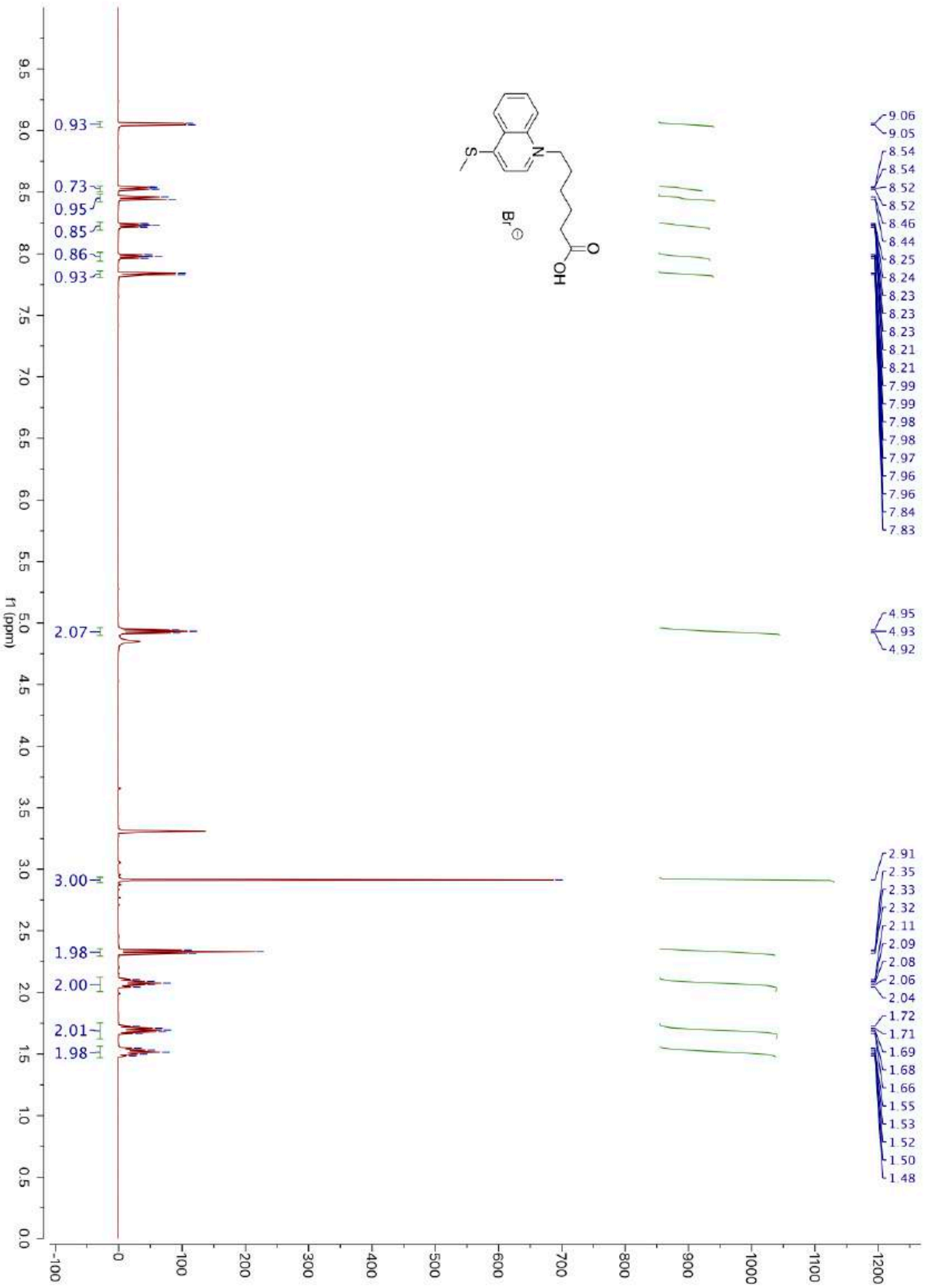

9.06
9.05
8.54
8.54
8.52
8.52
8.46
8.44
8.25
8.24
8.23
8.23
8.23
8.21
8.21
7.99
7.99
7.98
7.98
7.97
7.96
7.96
7.84
7.83
4.95
4.93
4.92
2.91
2.35
2.33
2.32
2.11
2.09
2.08
2.06
2.04
1.72
1.71
1.69
1.68
1.66
1.55
1.53
1.52
1.50
1.48
0.93
0.73
0.95
0.85
0.86
0.93
2.07
3.00
1.98
2.00
2.01
1.98
Br
OH
O
N
S
f1 (ppm)

3c
^{13}C NMR

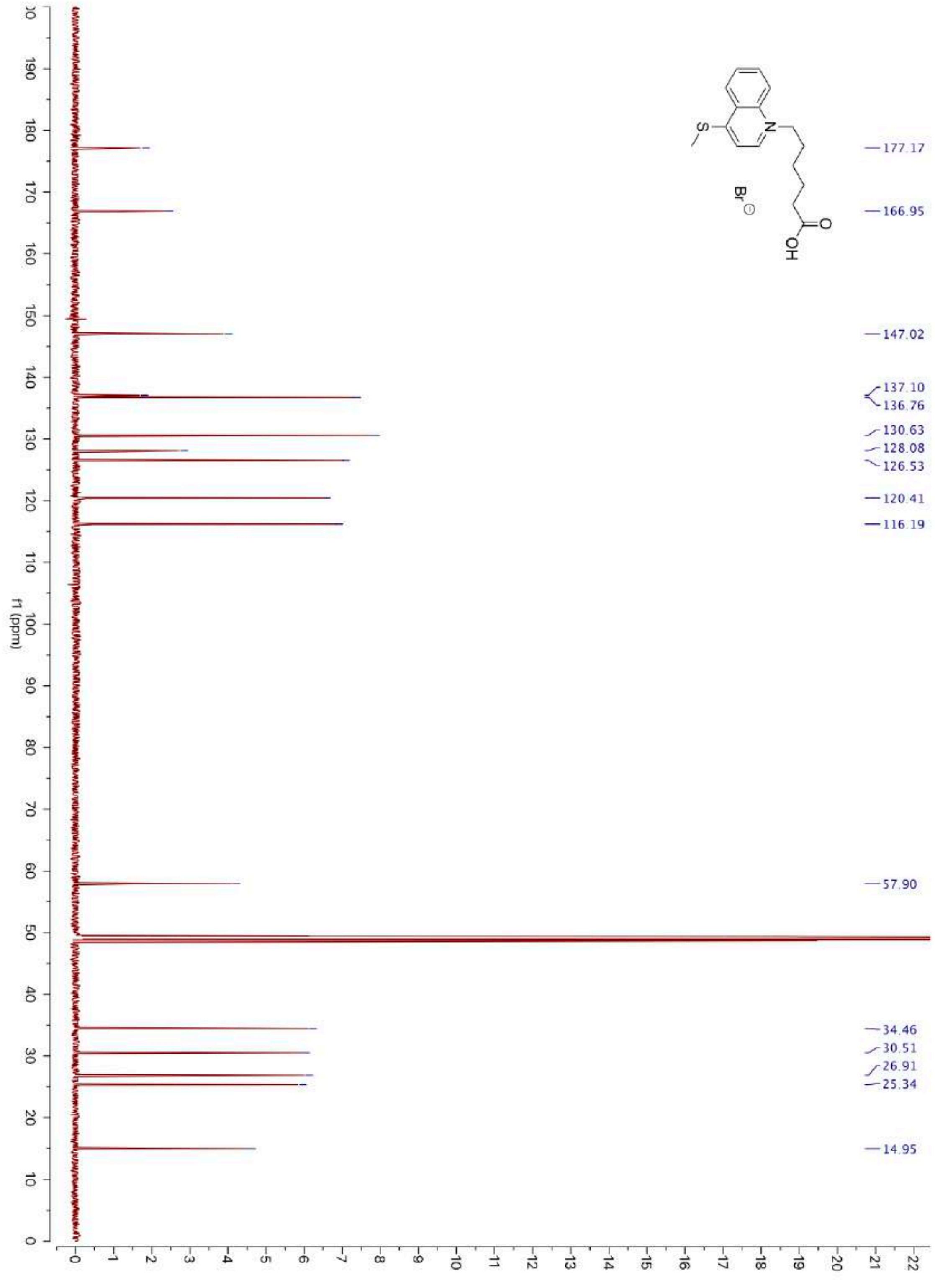

177.17
166.95
147.02
137.10
136.76
130.63
128.08
126.53
120.41
116.19
57.90
34.46
30.51
26.91
25.34
14.95
f1 (ppm)

4a
^{1}H NMR

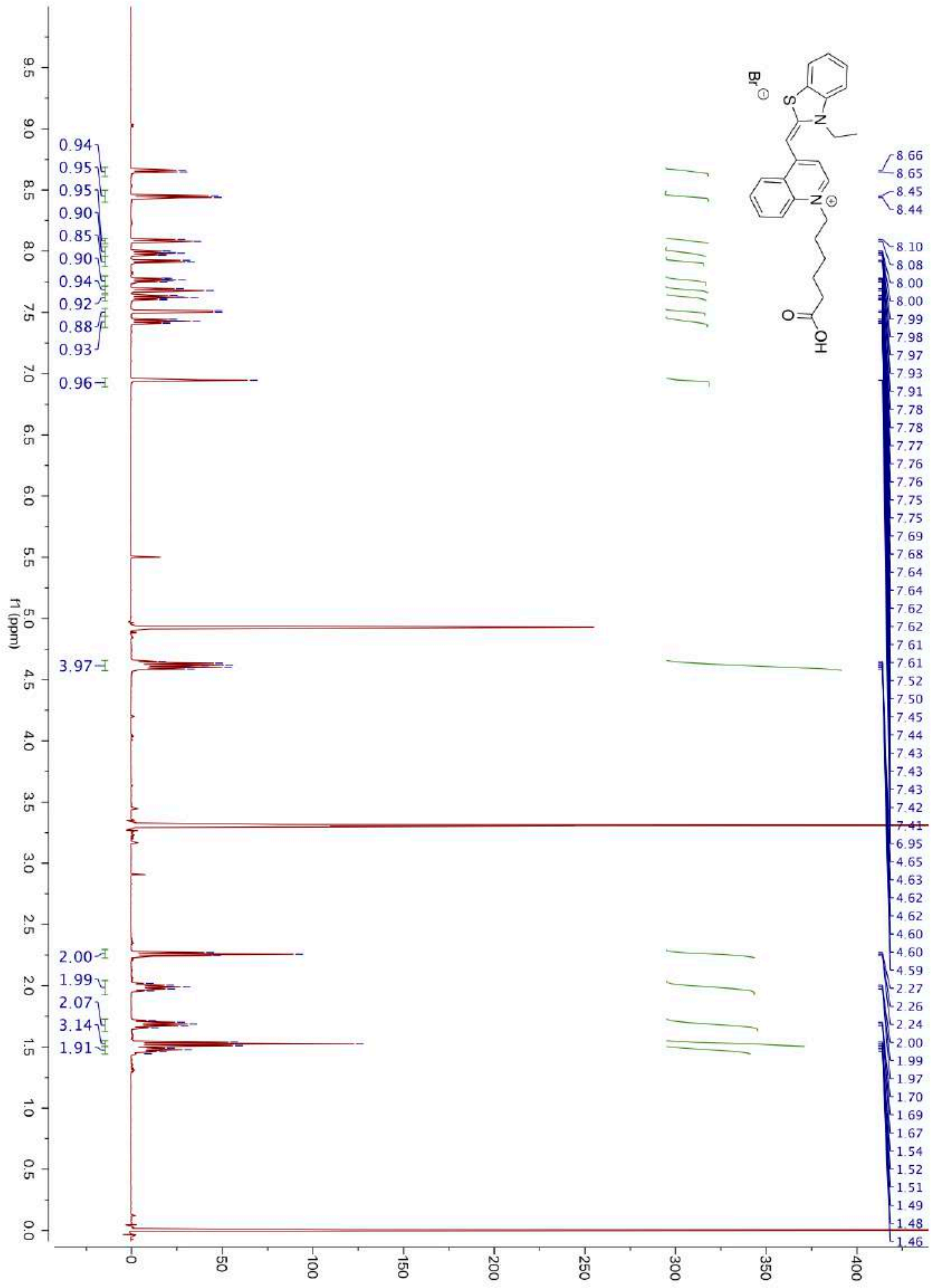

4a
^{13}C NMR

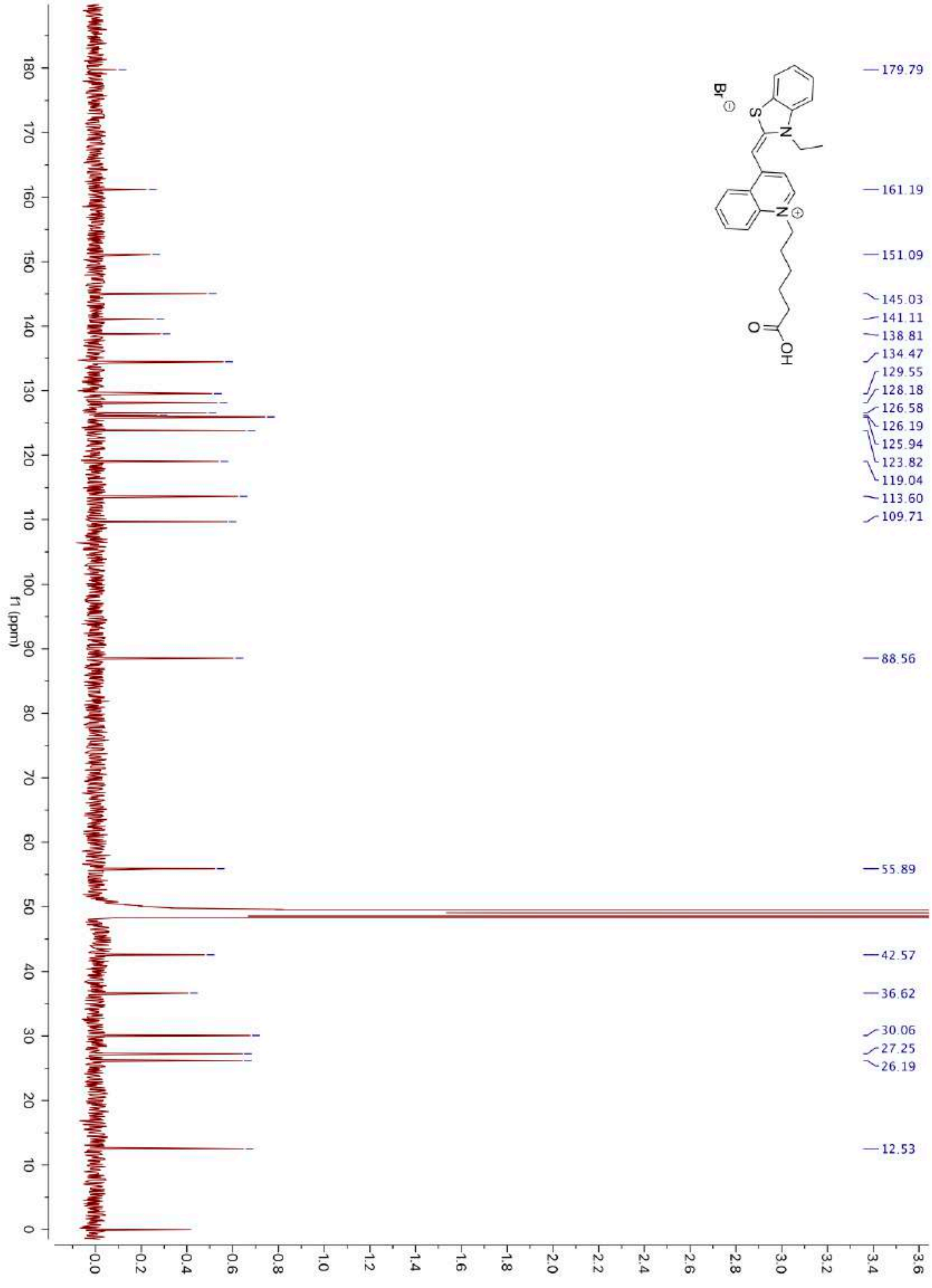
179.79
161.19
151.09
145.03
141.11
138.81
134.47
129.55
128.18
126.58
126.19
125.94
123.82
119.04
113.60
109.71
88.56
55.89
42.57
36.62
30.06
27.25
26.19
12.53
f1 (ppm)

4b

^{1}H NMR

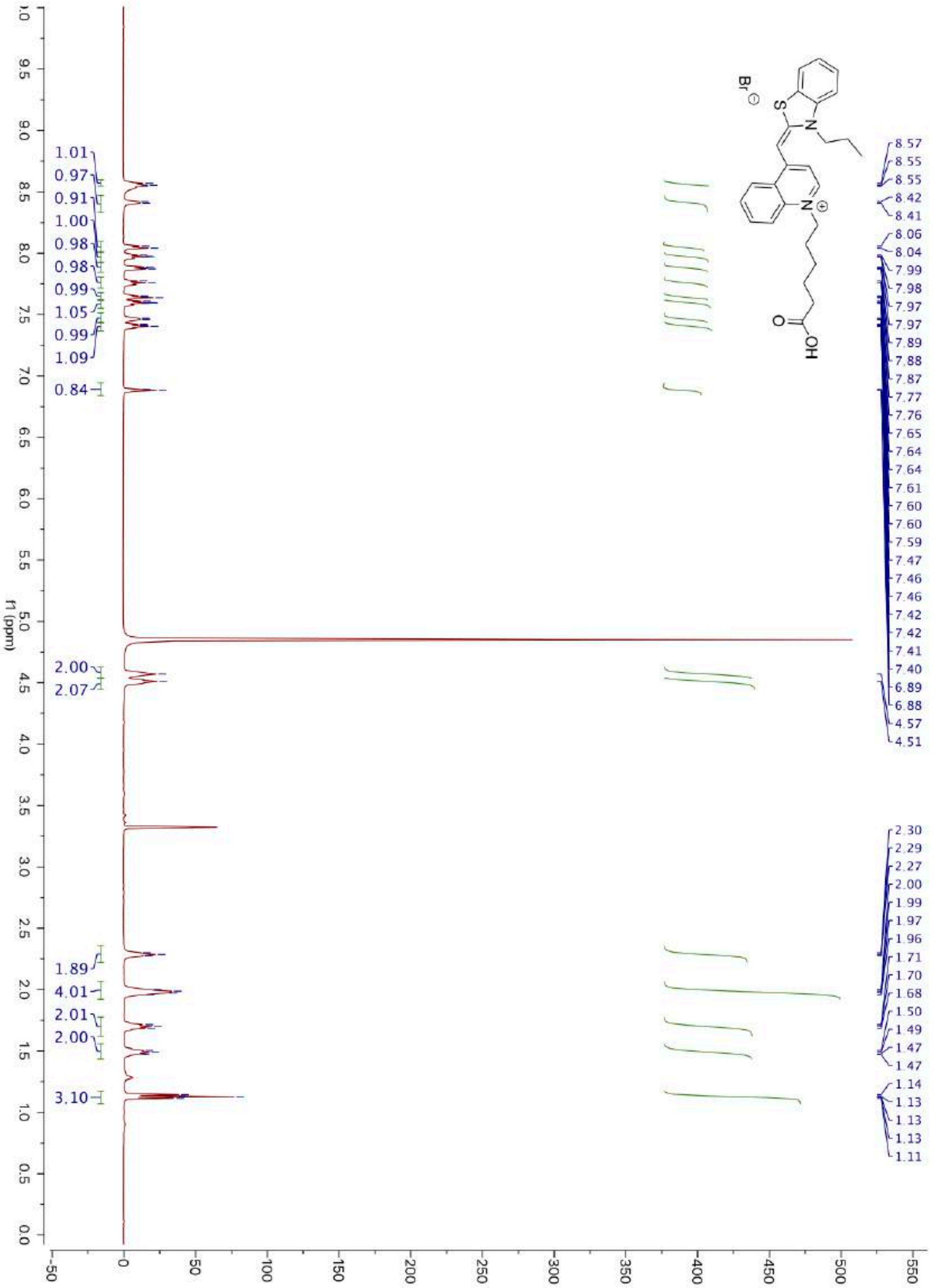

4b
^{13}C NMR

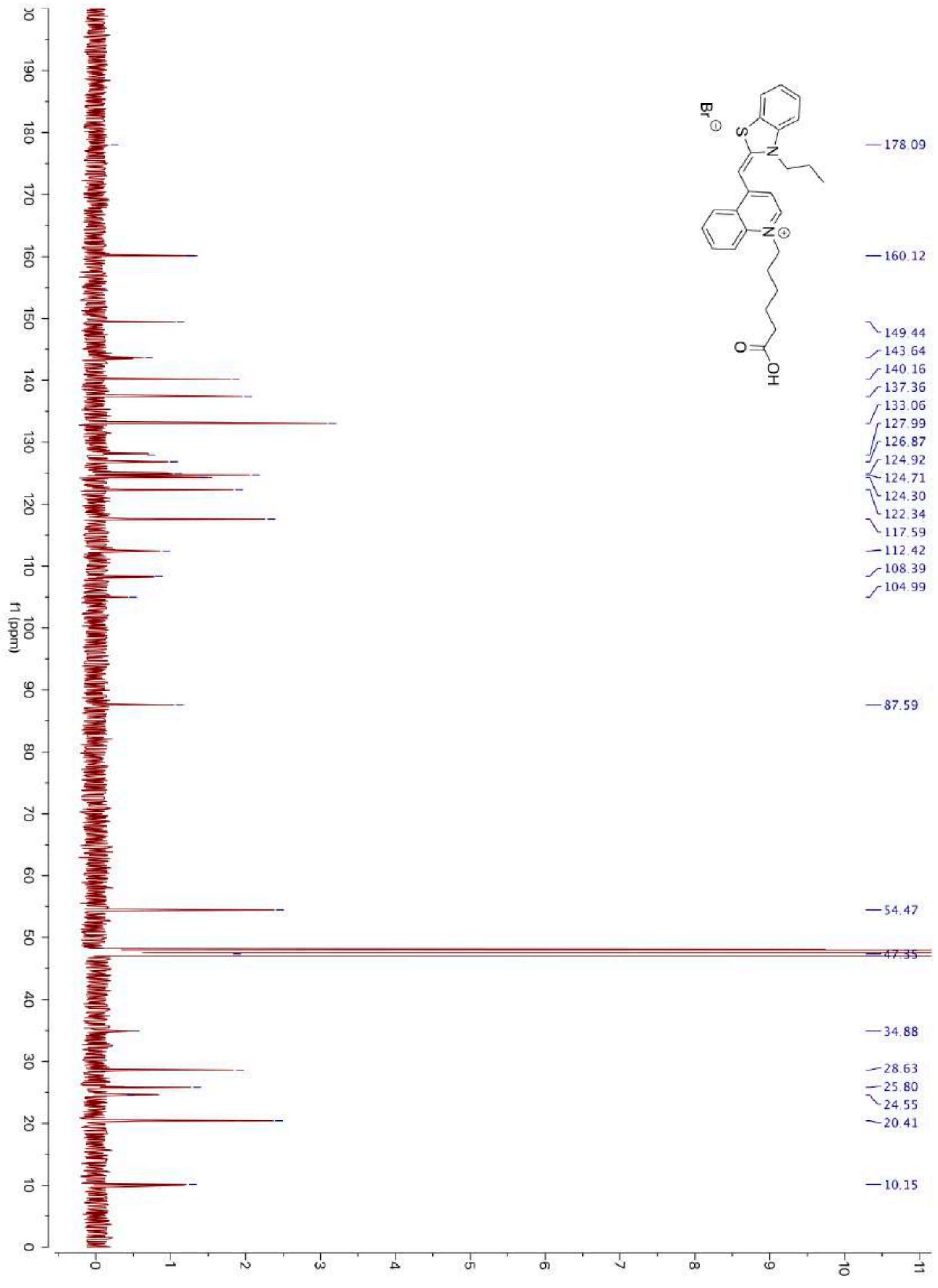
178.09
160.12
149.44
143.64
140.16
137.36
133.06
127.99
126.87
124.92
124.71
124.30
122.34
117.59
112.42
108.39
104.99
87.59
54.47
47.35
34.88
28.63
25.80
24.55
20.41
10.15
f1 (ppm)
Br
S
N
N
O
OH

4c

^{1}H NMR

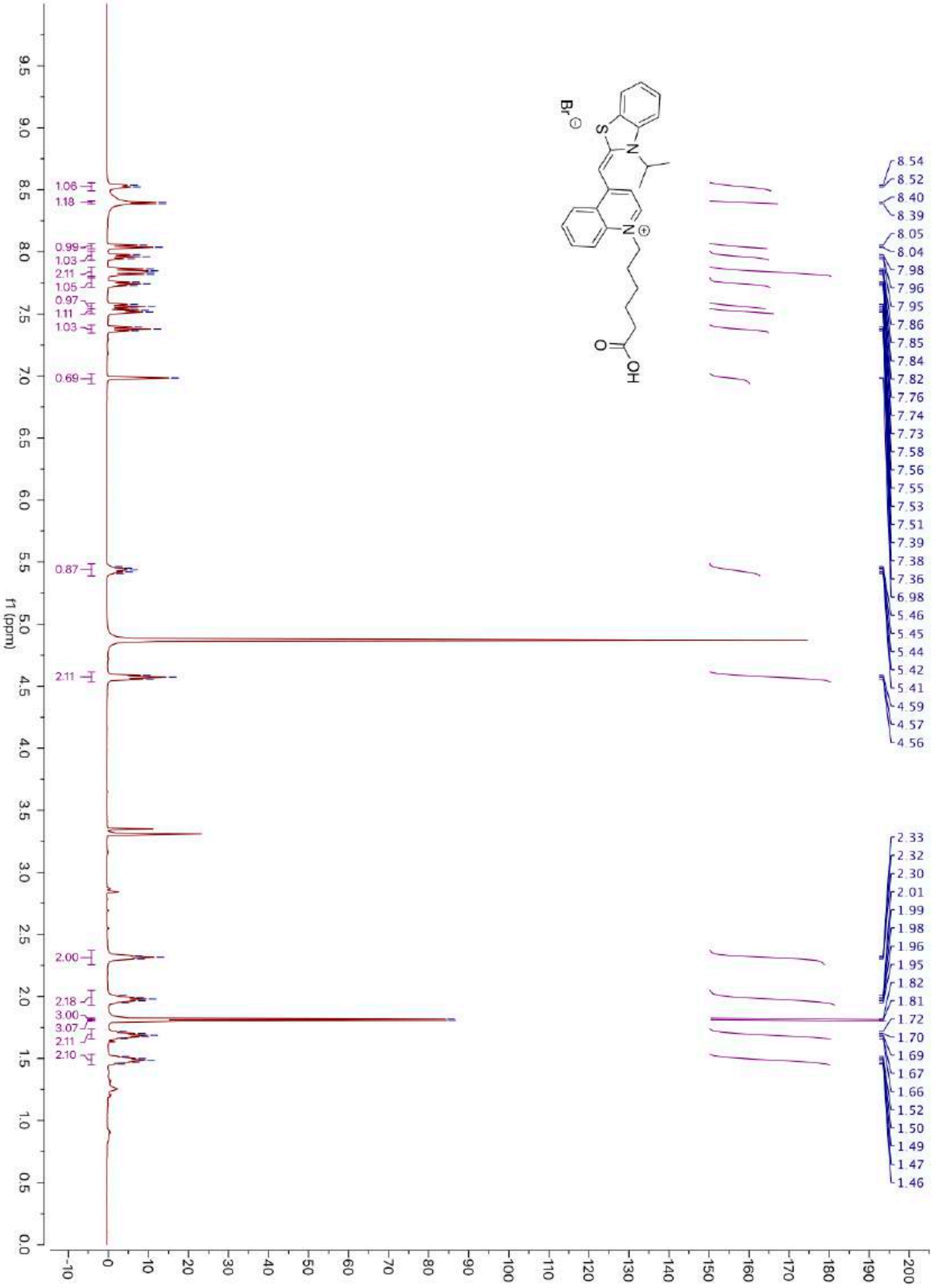

4c

^{13}C NMR

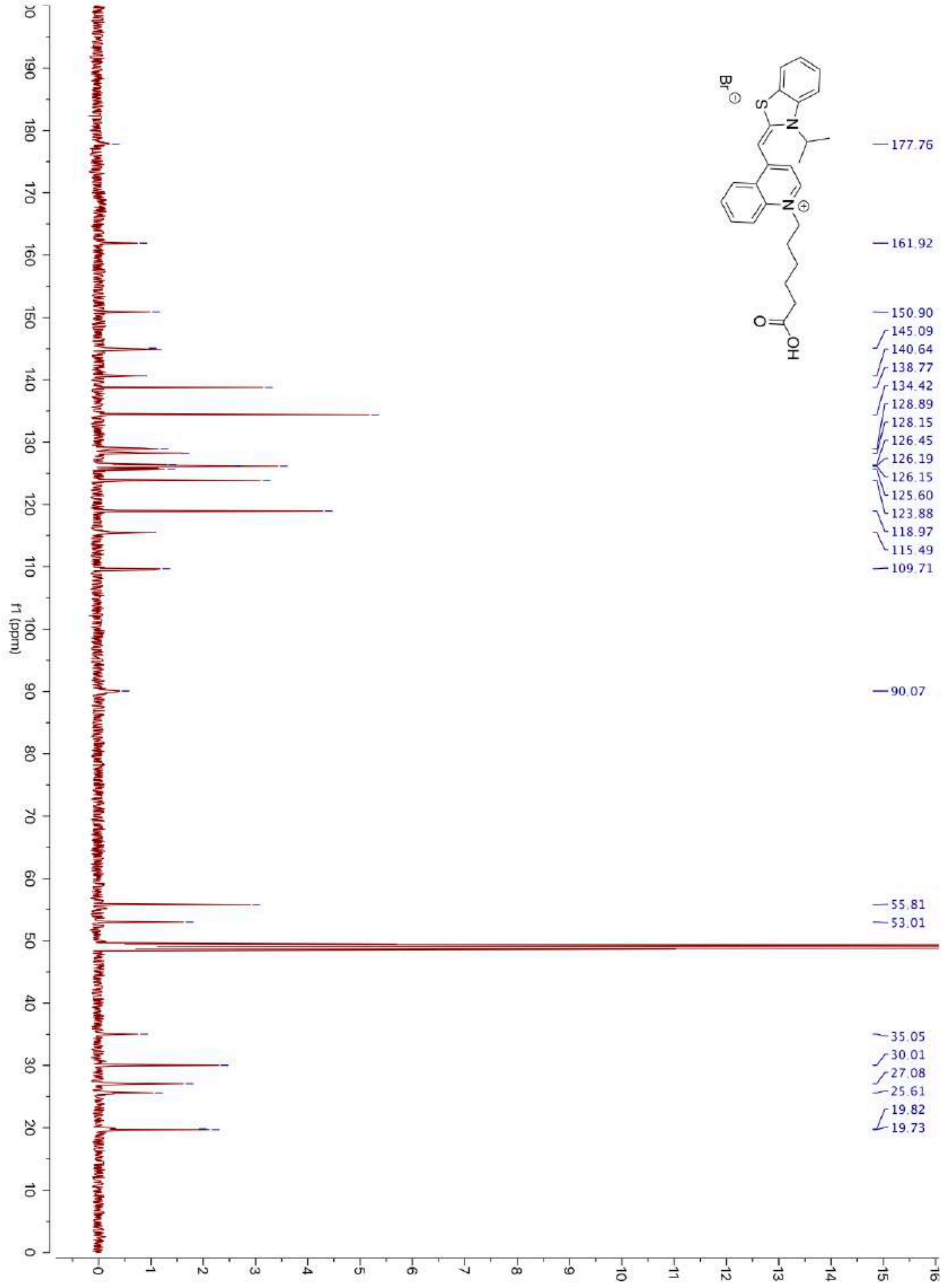

Br⁻
S
N
N⁺
O
OH
177.76
161.92
150.90
145.09
140.64
138.77
134.42
128.89
128.15
126.45
126.19
126.15
125.60
123.88
118.97
115.49
109.71
90.07
55.81
53.01
35.05
30.01
27.08
25.61
19.82
19.73
f1 (ppm)

4d

^{1}H NMR

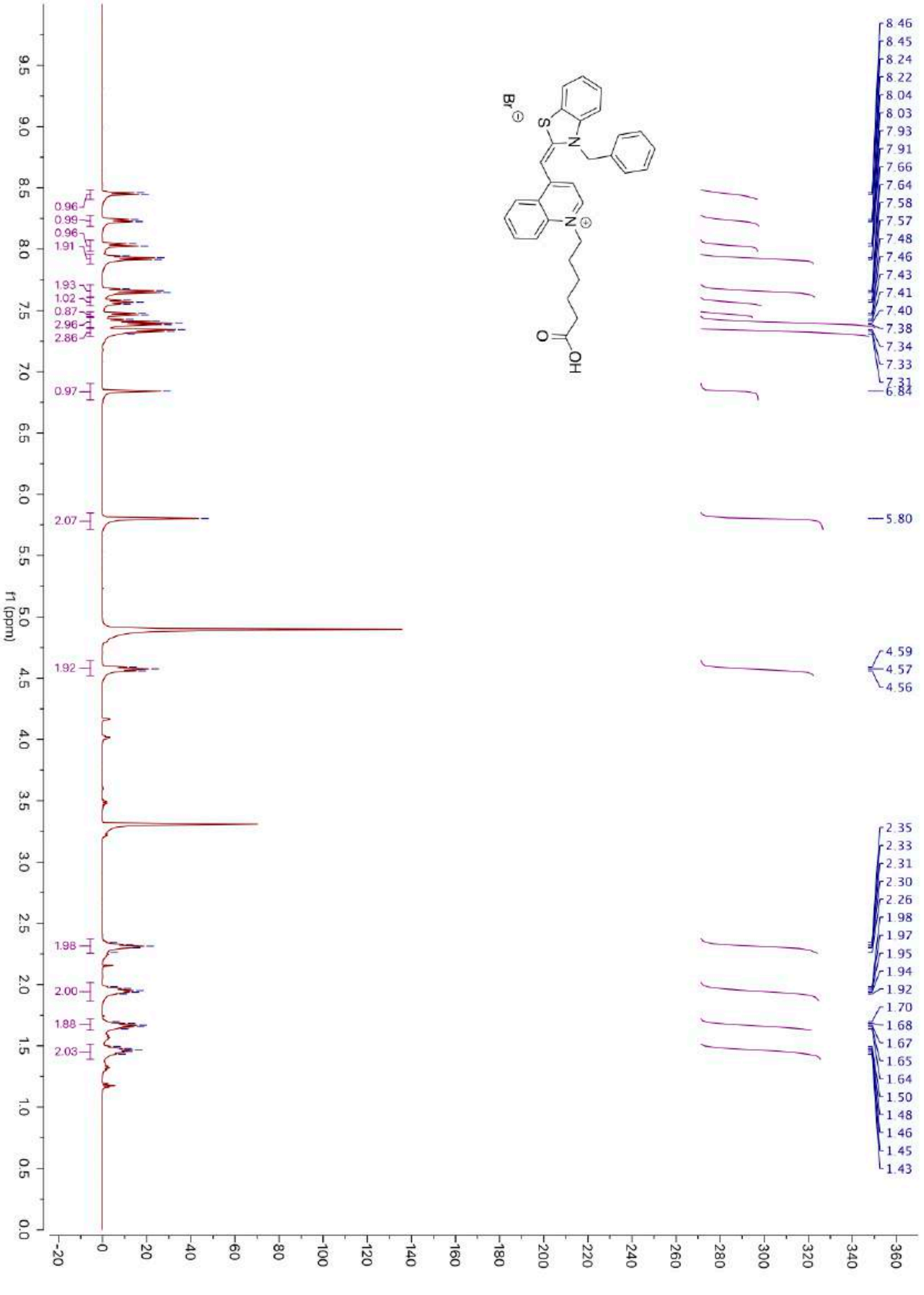
8.46
8.45
8.24
8.22
8.04
8.03
7.93
7.91
7.66
7.64
7.58
7.57
7.48
7.46
7.43
7.41
7.40
7.38
7.34
7.33
7.31
6.84
5.80
4.59
4.57
4.56
2.35
2.33
2.31
2.30
2.26
1.98
1.97
1.95
1.94
1.92
1.70
1.68
1.67
1.65
1.64
1.50
1.48
1.46
1.45
1.43
0.96
0.99
0.96
1.91
1.93
1.02
0.87
2.96
2.86
0.97
2.07
1.92
1.98
2.00
1.88
2.03
f1 (ppm)
Br
S
N
N
O
OH

4d

^{13}C NMR

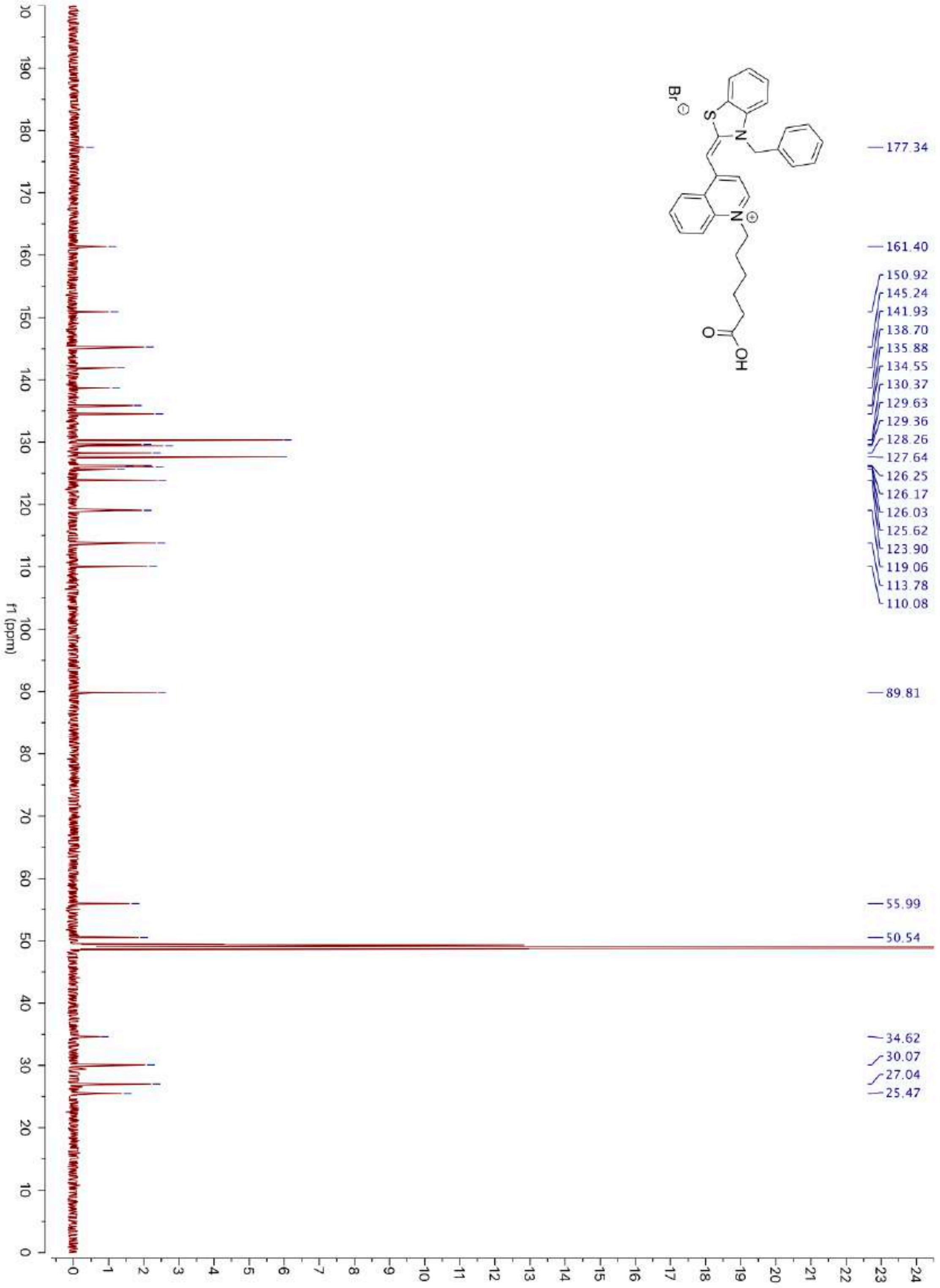
Br⁻
S
N
N⁺
O
OH
177.34
161.40
150.92
145.24
141.93
138.70
135.88
134.55
130.37
129.63
129.36
128.26
127.64
126.25
126.17
126.03
125.62
123.90
119.06
113.78
110.08
89.81
55.99
50.54
34.62
30.07
27.04
25.47
f1 (ppm)

4e
^{1}HNMR

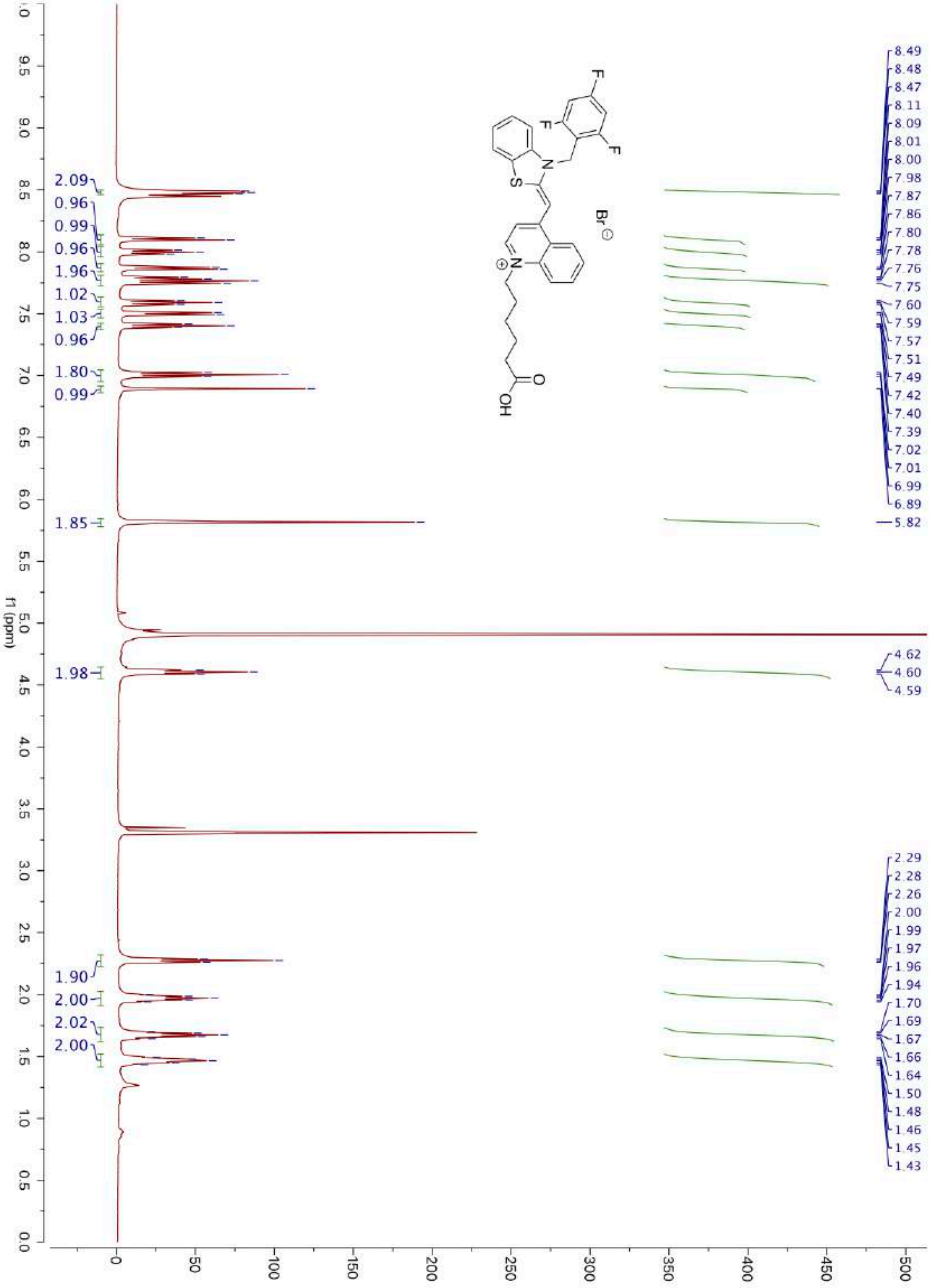

8.49
8.48
8.47
8.11
8.09
8.01
8.00
7.98
7.87
7.86
7.80
7.78
7.76
7.75
7.60
7.59
7.57
7.51
7.49
7.42
7.40
7.39
7.02
7.01
6.99
6.89
5.82
4.62
4.60
4.59
2.29
2.28
2.26
2.00
1.99
1.97
1.96
1.94
1.70
1.69
1.67
1.66
1.64
1.50
1.48
1.46
1.45
1.43
2.09
0.96
0.99
0.96
1.96
1.02
1.03
0.96
1.80
0.99
1.85
1.98
1.90
2.00
2.02
2.00
F
F
F
N
S
Br
N
O
OH
f1 (ppm)

4e
^{13}C NMR

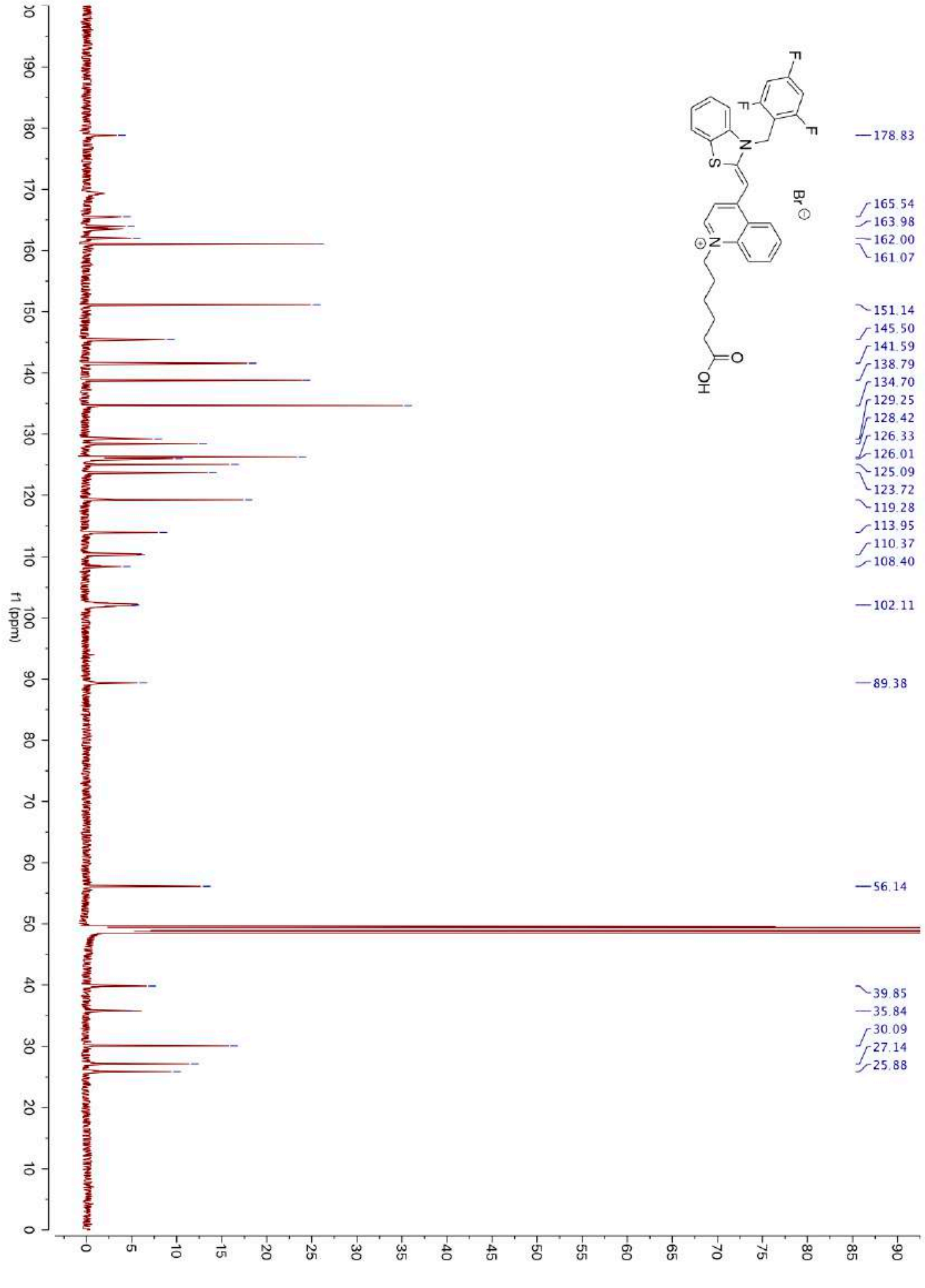

178.83
165.54
163.98
162.00
161.07
151.14
145.50
141.59
138.79
134.70
129.25
128.42
126.33
126.01
125.09
123.72
119.28
113.95
110.37
108.40
102.11
89.38
56.14
39.85
35.84
30.09
27.14
25.88
f1 (ppm)

4f

^{1}H NMR

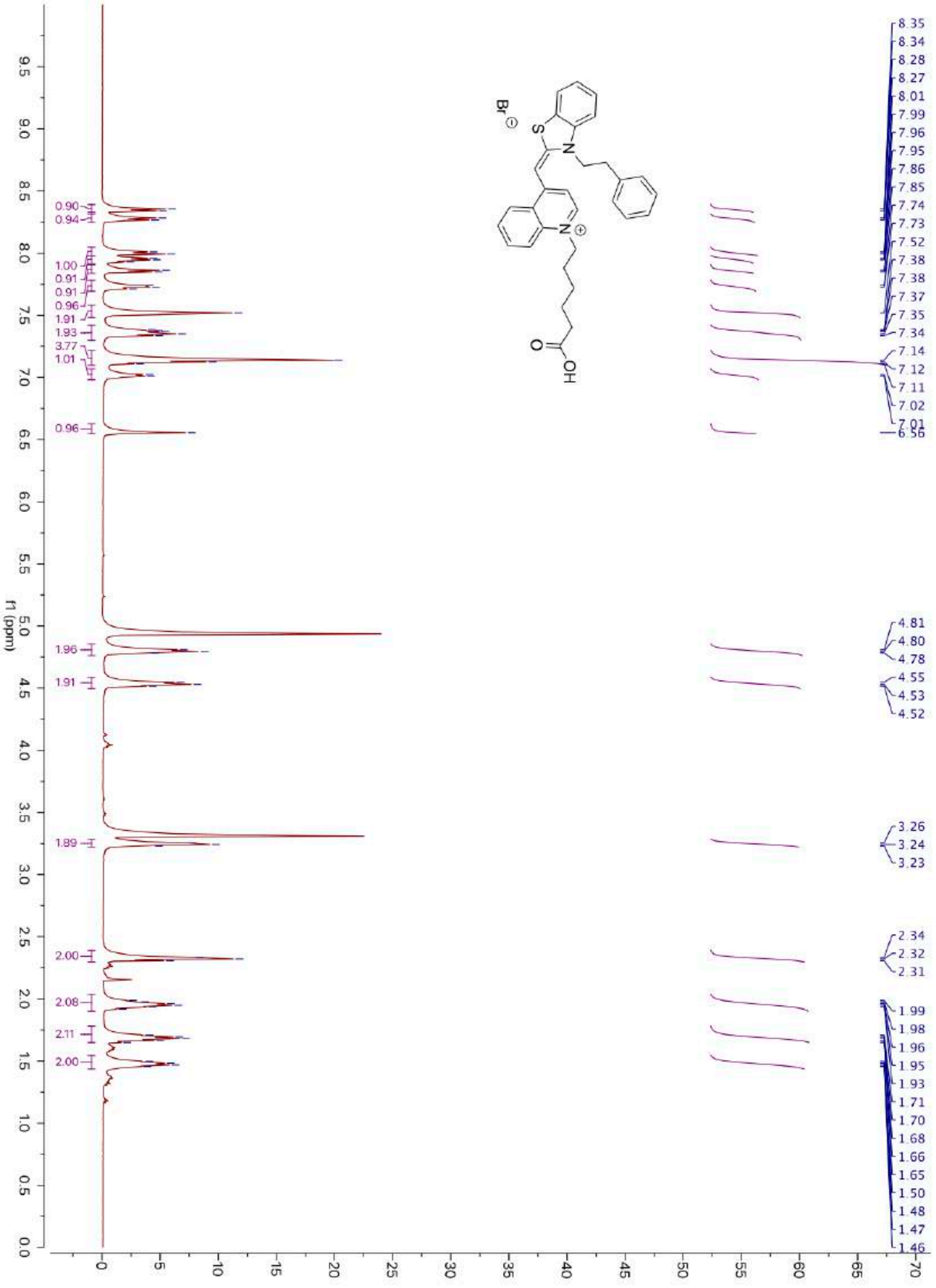

4f
^{13}C NMR

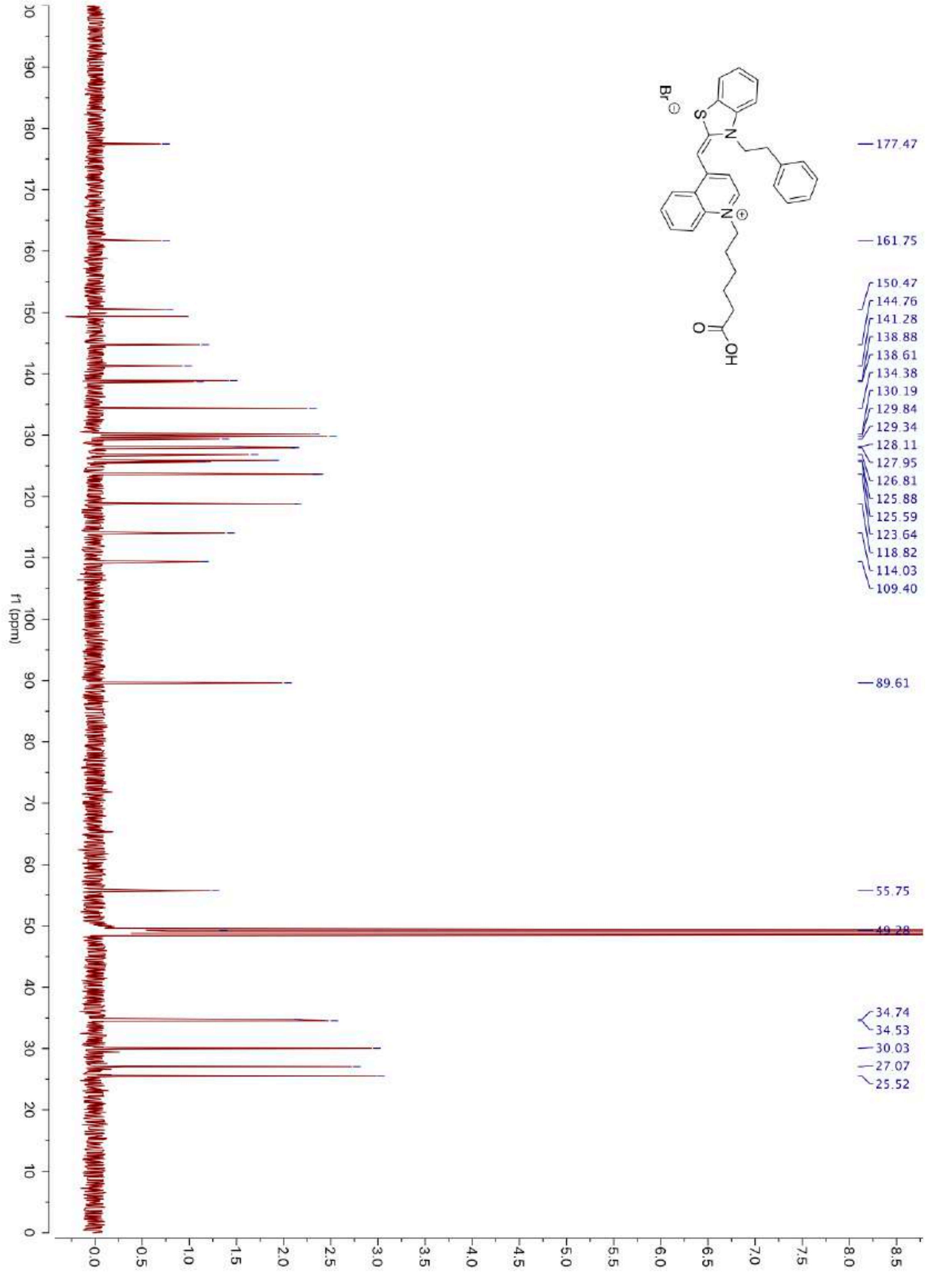
177.47
161.75
150.47
144.76
141.28
138.88
138.61
134.38
130.19
129.84
129.34
128.11
127.95
126.81
125.88
125.59
123.64
118.82
114.03
109.40
89.61
55.75
49.28
34.74
34.53
30.03
27.07
25.52
f1 (ppm)
Br
S
N
N
O
OH

4g

^{1}H NMR

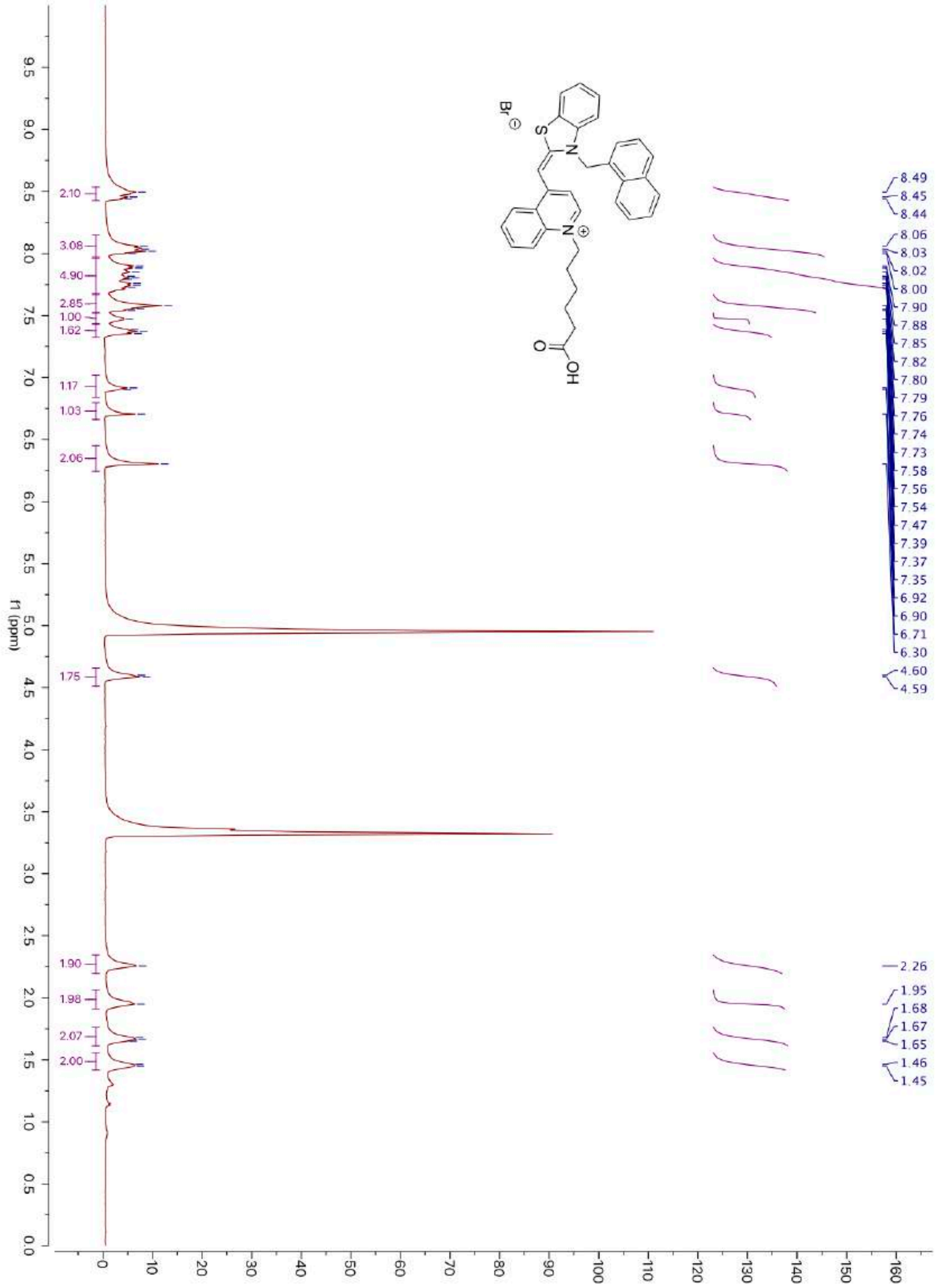

8.49
8.45
8.44
8.06
8.03
8.02
8.00
7.90
7.88
7.85
7.82
7.80
7.79
7.76
7.74
7.73
7.58
7.56
7.54
7.47
7.39
7.37
7.35
6.92
6.90
6.71
6.30
4.60
4.59
2.26
1.95
1.68
1.67
1.65
1.46
1.45
2.10
3.08
4.90
2.85
1.00
1.62
1.17
1.03
2.06
1.75
1.90
1.98
2.07
2.00
f1 (ppm)
Br
S
N
N
O
OH

4g
^{13}C NMR

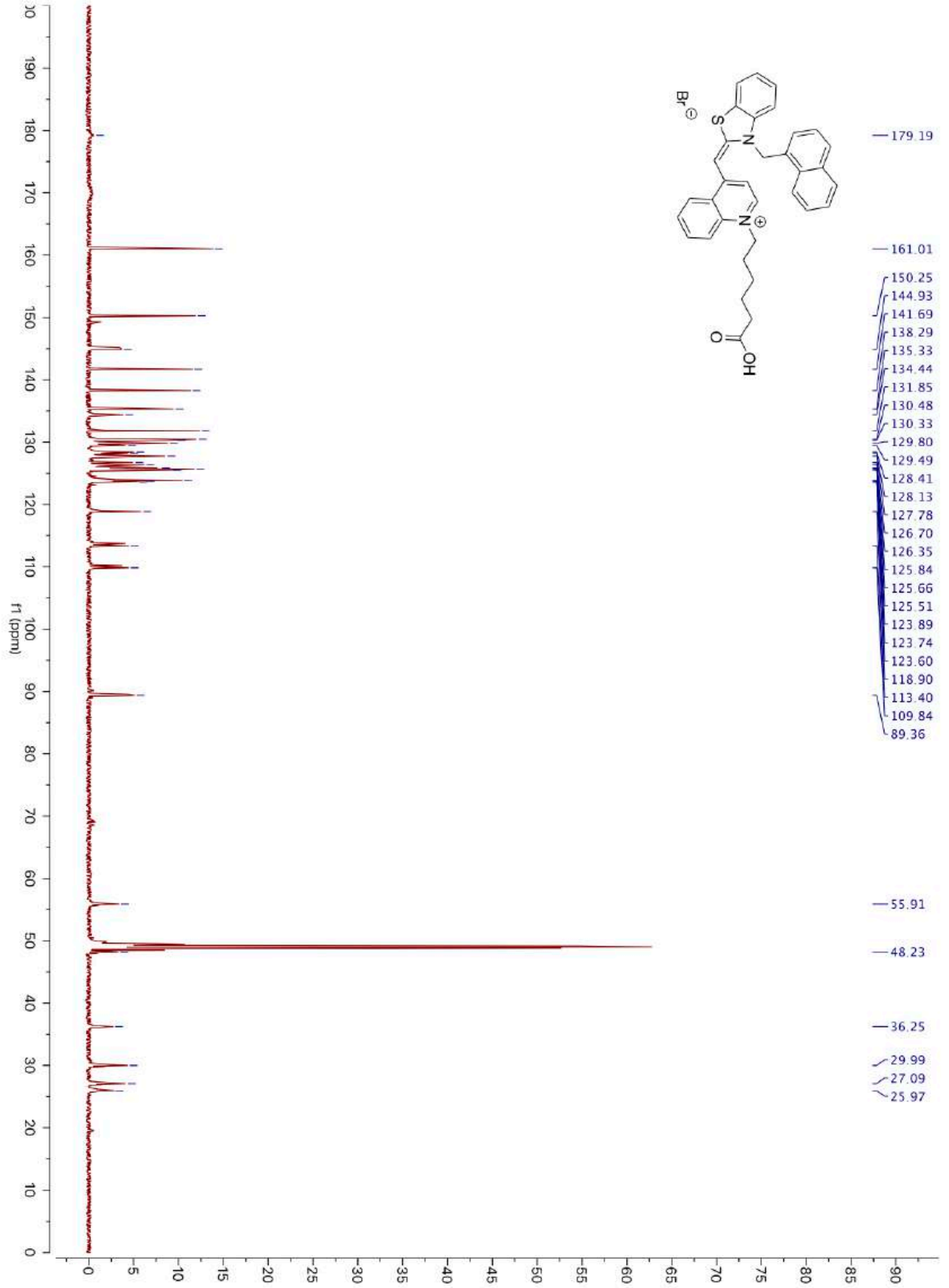
Br⊖
S
N
N⊕
O
OH
179.19
161.01
150.25
144.93
141.69
138.29
135.33
134.44
131.85
130.48
130.33
129.80
129.49
128.41
128.13
127.78
126.70
126.35
125.84
125.66
125.51
123.89
123.74
123.60
118.90
113.40
109.84
89.36
55.91
48.23
36.25
29.99
27.09
25.97
f1 (ppm)

4h
^{1}H NMR

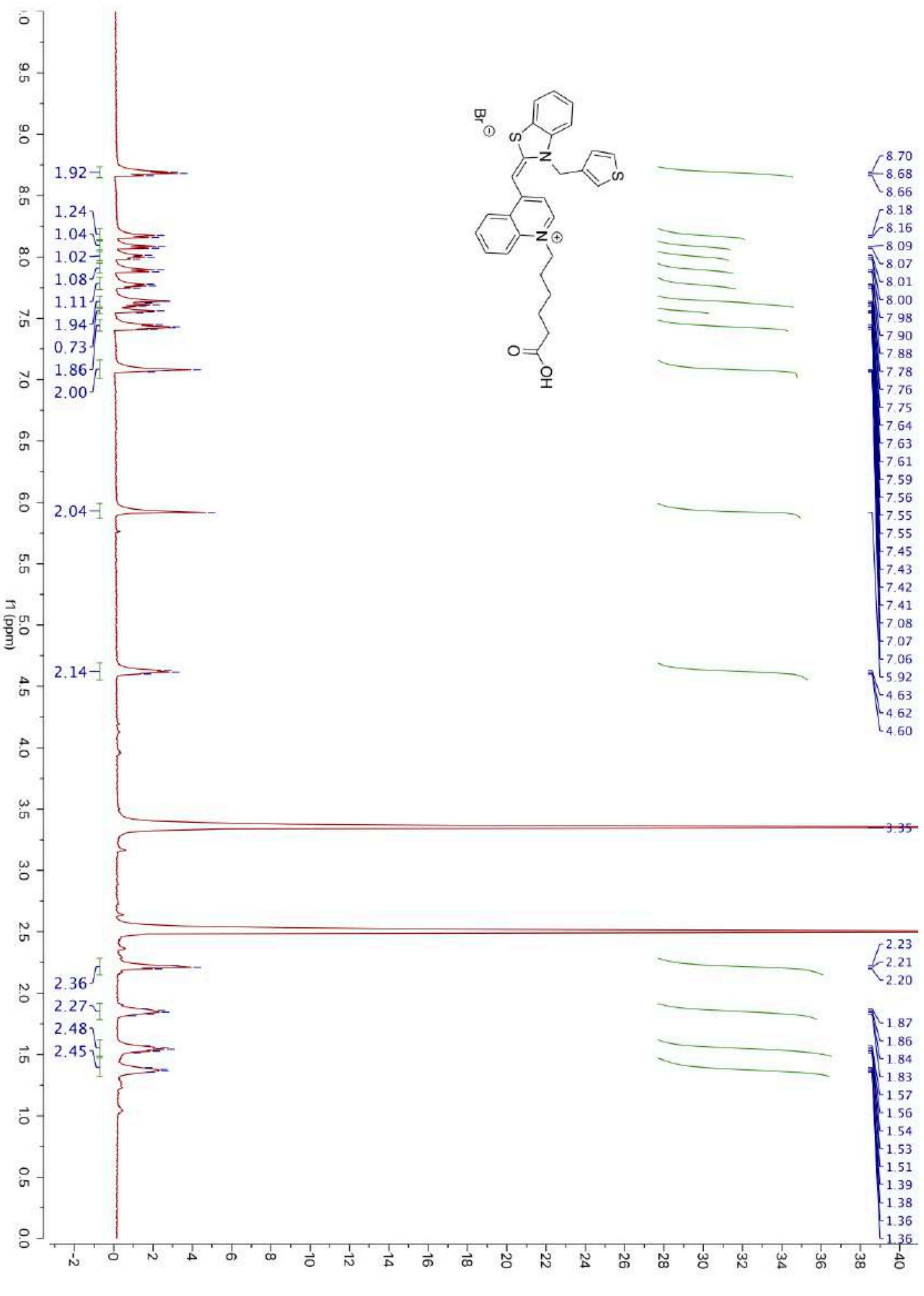

4h
^{13}C NMR

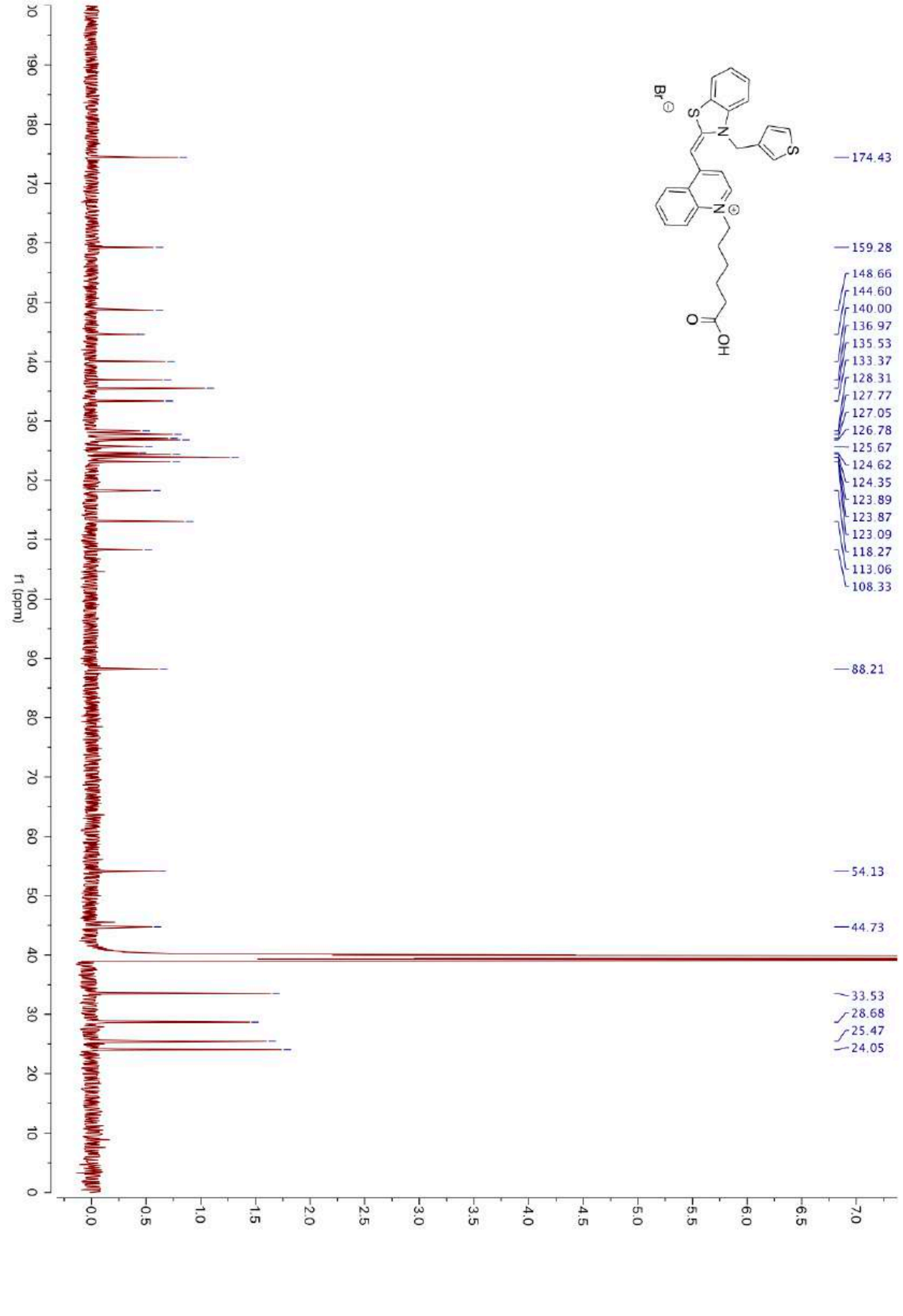

174.43
159.28
148.66
144.60
140.00
136.97
135.53
133.37
128.31
127.77
127.05
126.78
125.67
124.62
124.35
123.89
123.87
123.09
118.27
113.06
108.33
88.21
54.13
44.73
33.53
28.68
25.47
24.05
f1 (ppm)

4i

^{1}H NMR

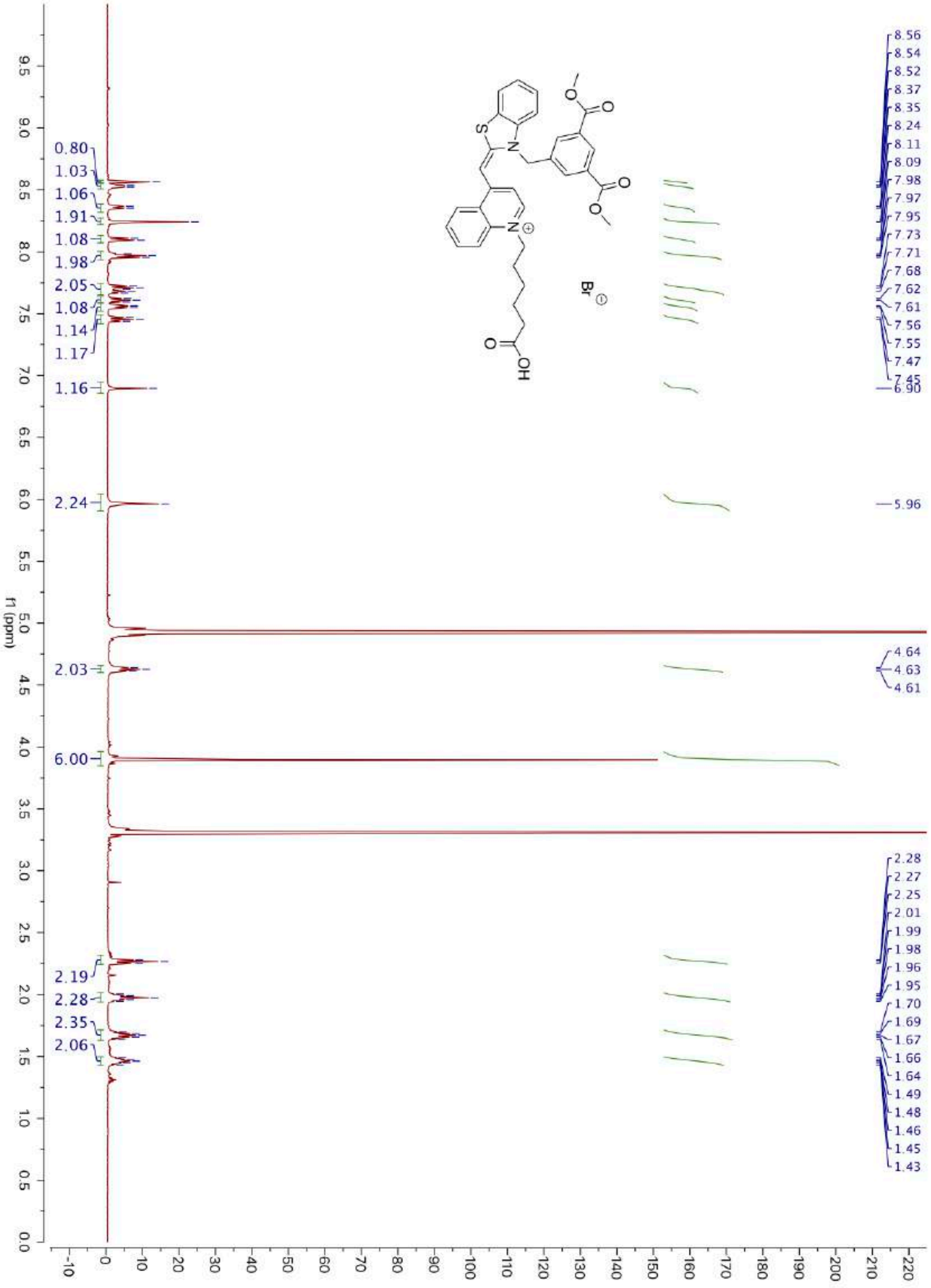

8.56
8.54
8.52
8.37
8.35
8.24
8.11
8.09
7.98
7.97
7.95
7.73
7.71
7.68
7.62
7.61
7.56
7.55
7.47
7.45
6.90
5.96
4.64
4.63
4.61
2.28
2.27
2.25
2.01
1.99
1.98
1.96
1.95
1.70
1.69
1.67
1.66
1.64
1.49
1.48
1.46
1.45
1.43
0.80
1.03
1.06
1.91
1.08
1.98
2.05
1.08
1.14
1.17
1.16
2.24
2.03
6.00
2.19
2.28
2.35
2.06
f1 (ppm)
Br

4i

^{13}C NMR

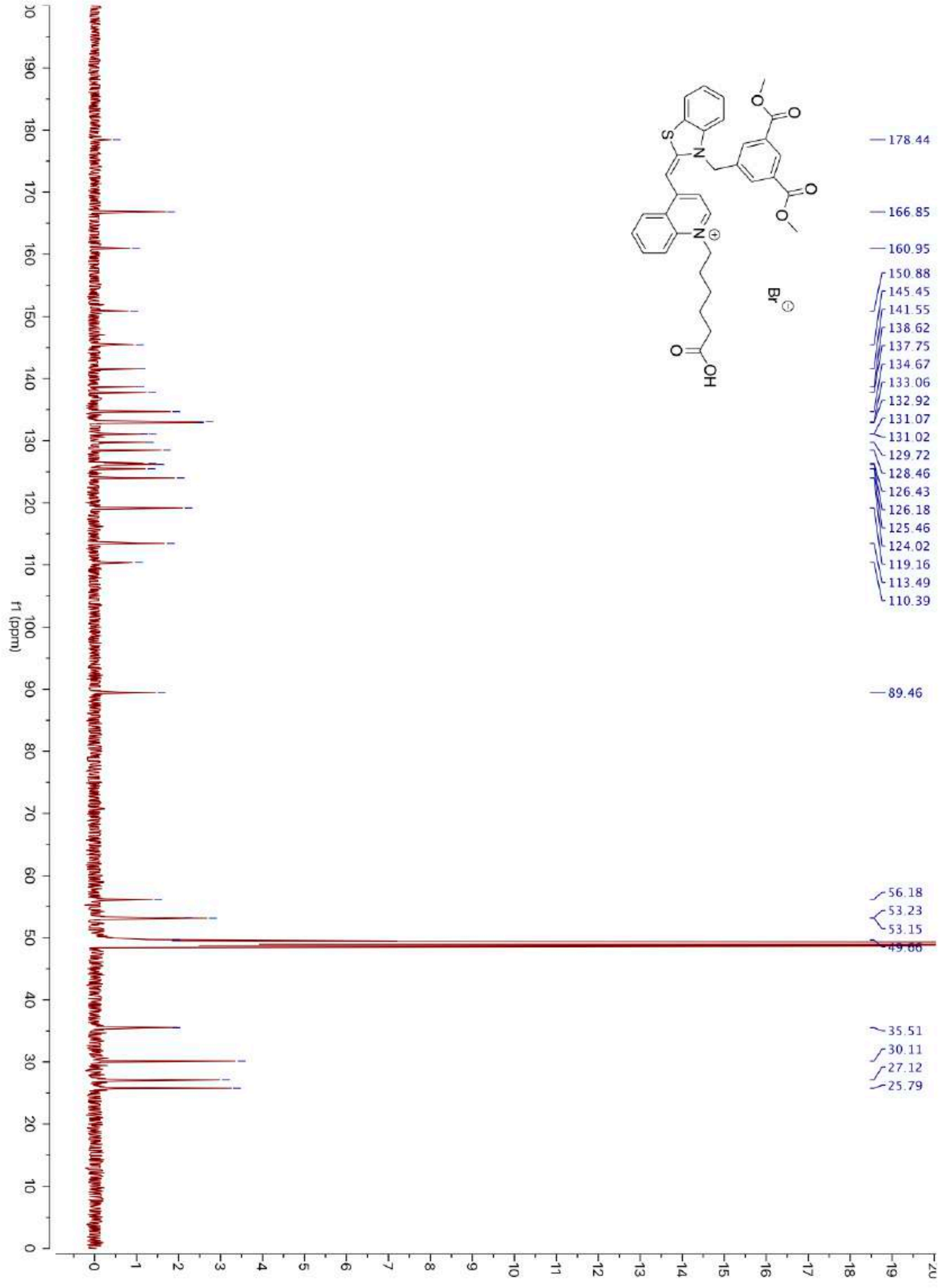

178.44
166.85
160.95
150.88
145.45
141.55
138.62
137.75
134.67
133.06
132.92
131.07
131.02
129.72
128.46
126.43
126.18
125.46
124.02
119.16
113.49
110.39
89.46
56.18
53.23
53.15
49.00
35.51
30.11
27.12
25.79
f1 (ppm)
Br

4j
^{1}H NMR

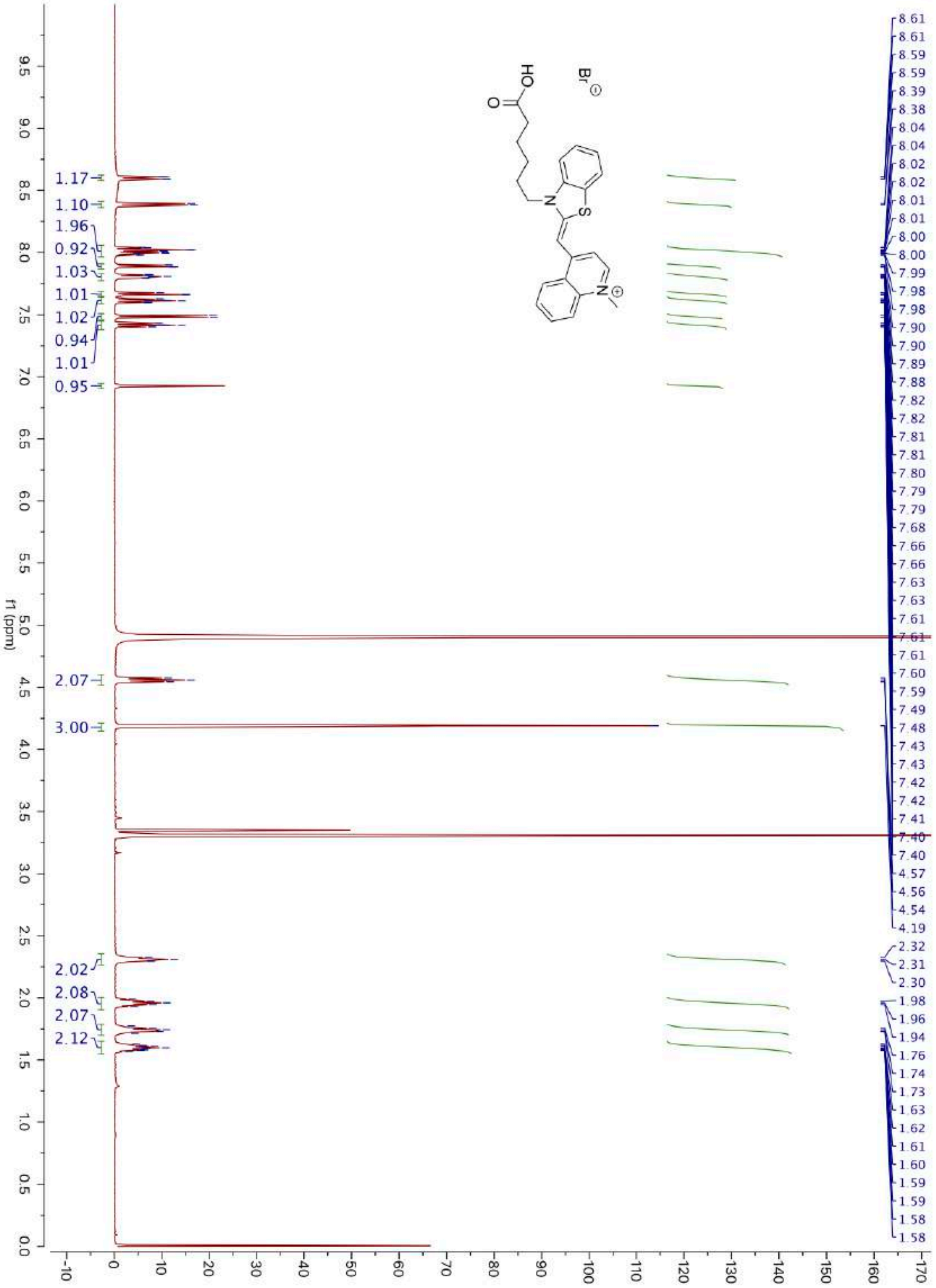
8.61
8.61
8.59
8.59
8.39
8.38
8.04
8.04
8.02
8.02
8.01
8.01
8.00
8.00
7.99
7.98
7.98
7.90
7.90
7.89
7.88
7.82
7.82
7.81
7.81
7.80
7.79
7.79
7.68
7.66
7.66
7.63
7.63
7.61
7.61
7.61
7.60
7.59
7.49
7.48
7.43
7.43
7.42
7.42
7.41
7.40
7.40
4.57
4.56
4.54
4.19
2.32
2.31
2.30
1.98
1.96
1.94
1.76
1.74
1.73
1.63
1.62
1.61
1.60
1.59
1.59
1.58
1.58
1.17
1.10
1.96
0.92
1.03
1.01
1.02
0.94
1.01
0.95
2.07
3.00
2.02
2.08
2.07
2.12
HO
O
Br
N
S
N
f1 (ppm)

4j

^{13}C NMR

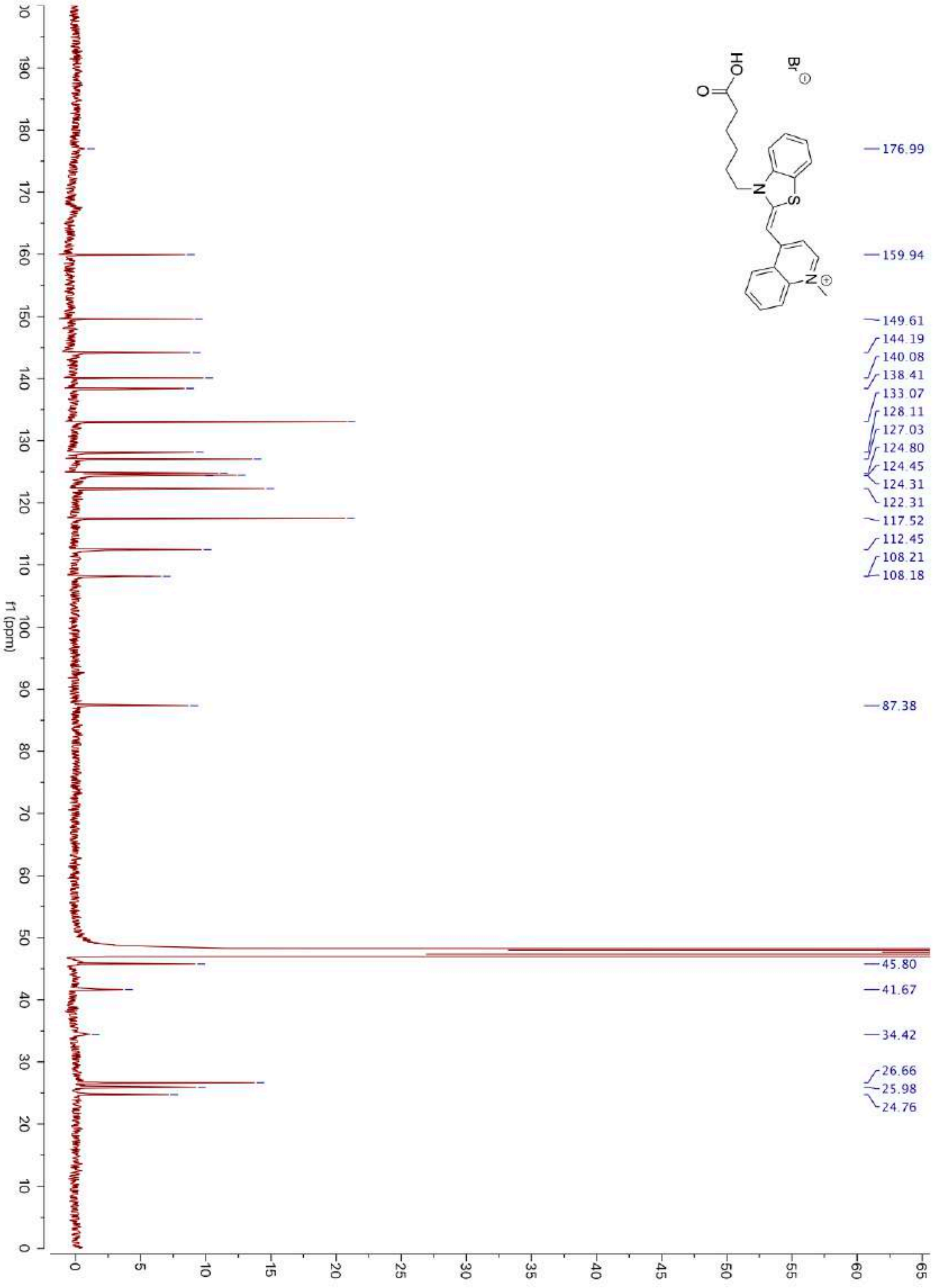

176.99
159.94
149.61
144.19
140.08
138.41
133.07
128.11
127.03
124.80
124.45
124.31
122.31
117.52
112.45
108.21
108.18
87.38
45.80
41.67
34.42
26.66
25.98
24.76
f1 (ppm)
HO
O
Br
N
S

4k

^{1}H NMR

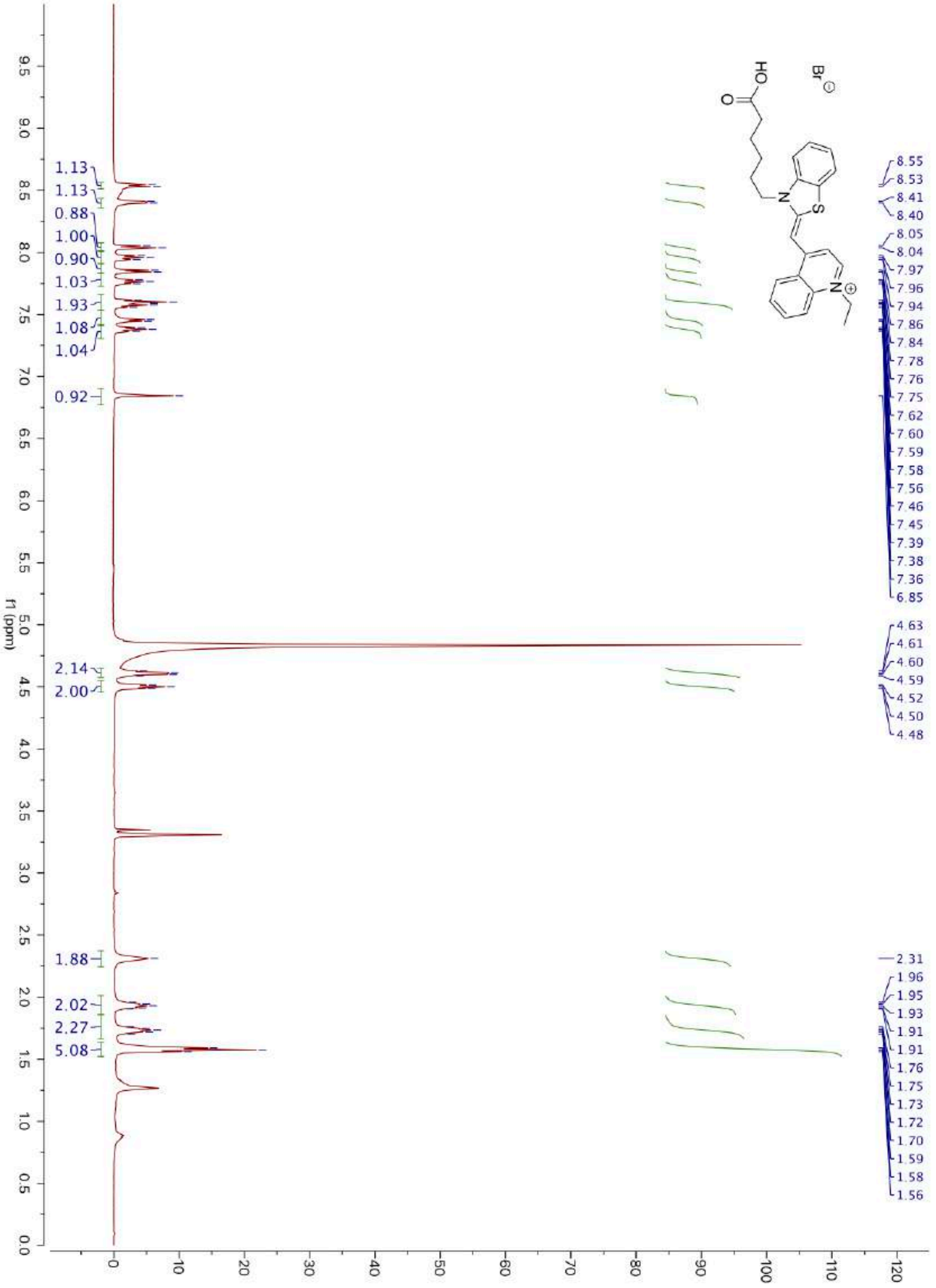

f1 (ppm)
8.55
8.53
8.41
8.40
8.05
8.04
7.97
7.96
7.94
7.86
7.84
7.78
7.76
7.75
7.62
7.60
7.59
7.58
7.56
7.46
7.45
7.39
7.38
7.36
6.85
4.63
4.61
4.60
4.59
4.52
4.50
4.48
2.31
1.96
1.95
1.93
1.91
1.91
1.76
1.75
1.73
1.72
1.70
1.59
1.58
1.56

4k

^{13}C NMR

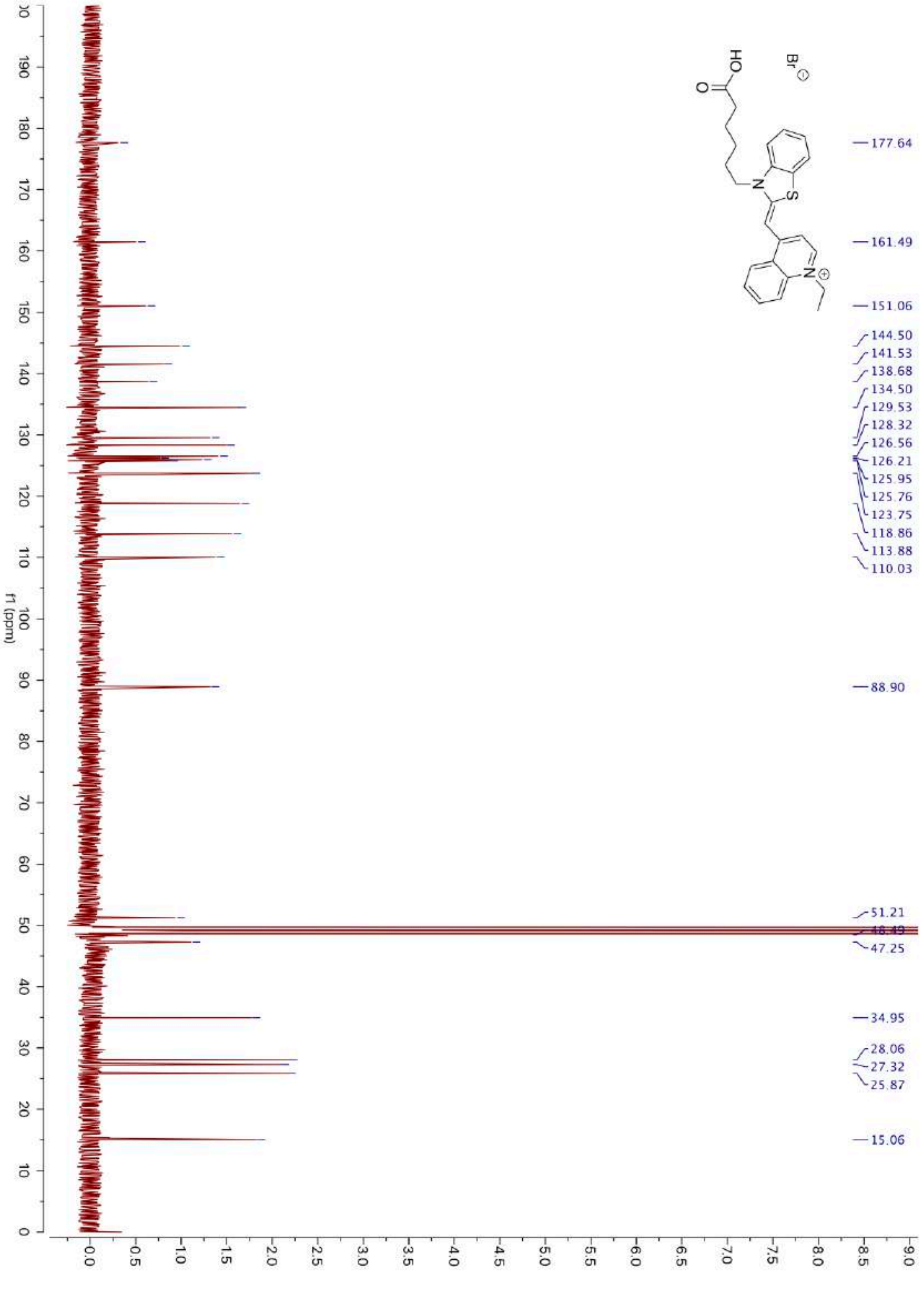

177.64
161.49
151.06
144.50
141.53
138.68
134.50
129.53
128.32
126.56
126.21
125.95
125.76
123.75
118.86
113.88
110.03
88.90
51.21
48.49
47.25
34.95
28.06
27.32
25.87
15.06
f1 (ppm)

5a

^{1}H NMR

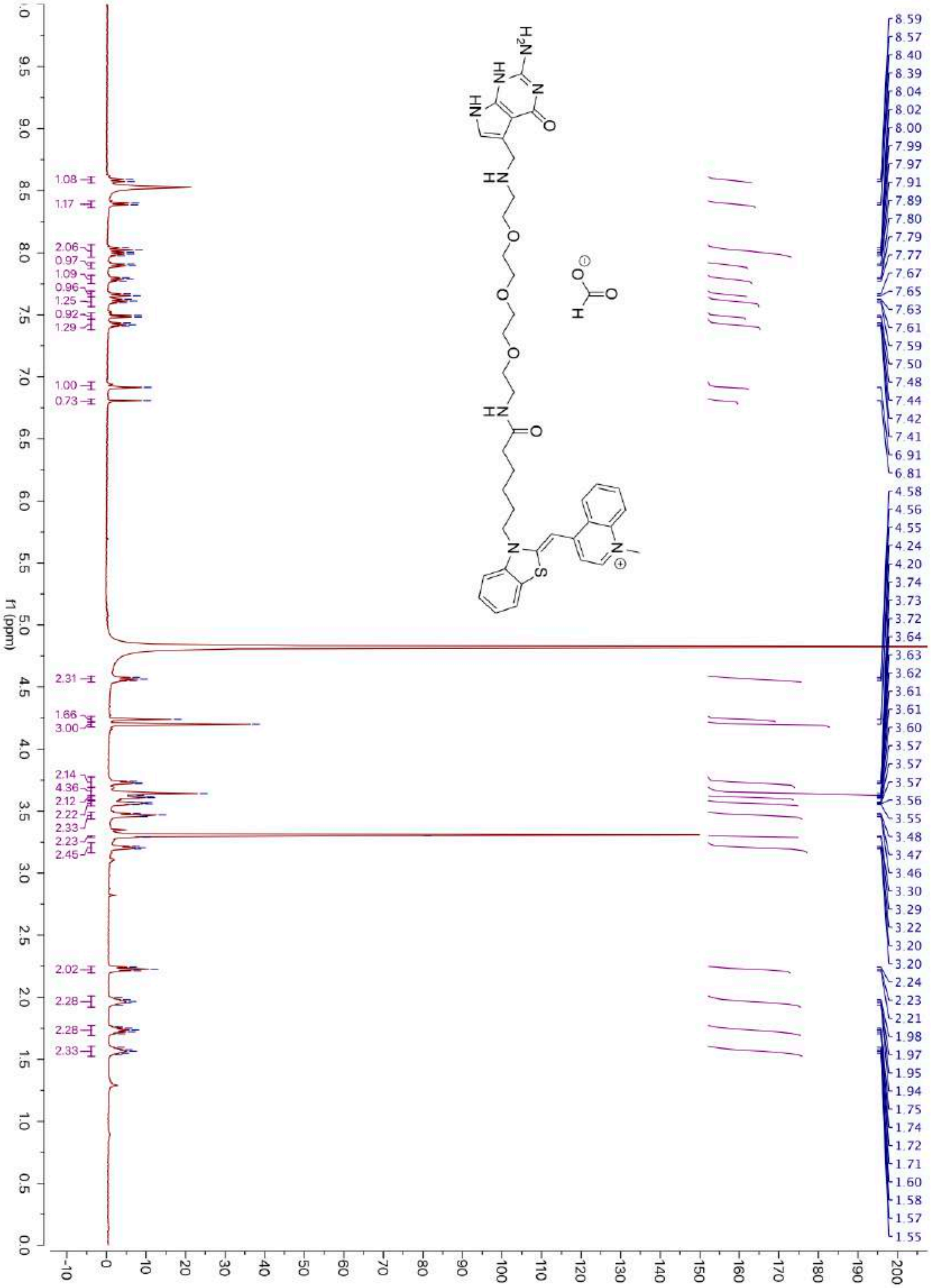

f1 (ppm)
8.59
8.57
8.40
8.39
8.04
8.02
8.00
7.99
7.97
7.91
7.89
7.80
7.79
7.77
7.67
7.65
7.63
7.61
7.59
7.50
7.48
7.44
7.42
7.41
6.91
6.81
4.58
4.56
4.55
4.24
4.20
3.74
3.73
3.72
3.64
3.63
3.62
3.61
3.61
3.60
3.57
3.57
3.57
3.56
3.55
3.48
3.47
3.46
3.30
3.29
3.22
3.20
3.20
2.24
2.23
2.21
1.98
1.97
1.95
1.94
1.75
1.74
1.72
1.71
1.60
1.58
1.57
1.55
1.08
1.17
2.06
0.97
1.09
0.96
1.25
0.92
1.29
1.00
0.73
2.31
1.66
3.00
2.14
4.36
2.12
2.22
2.33
2.23
2.45
2.02
2.28
2.28
2.33

5a

^{13}C NMR

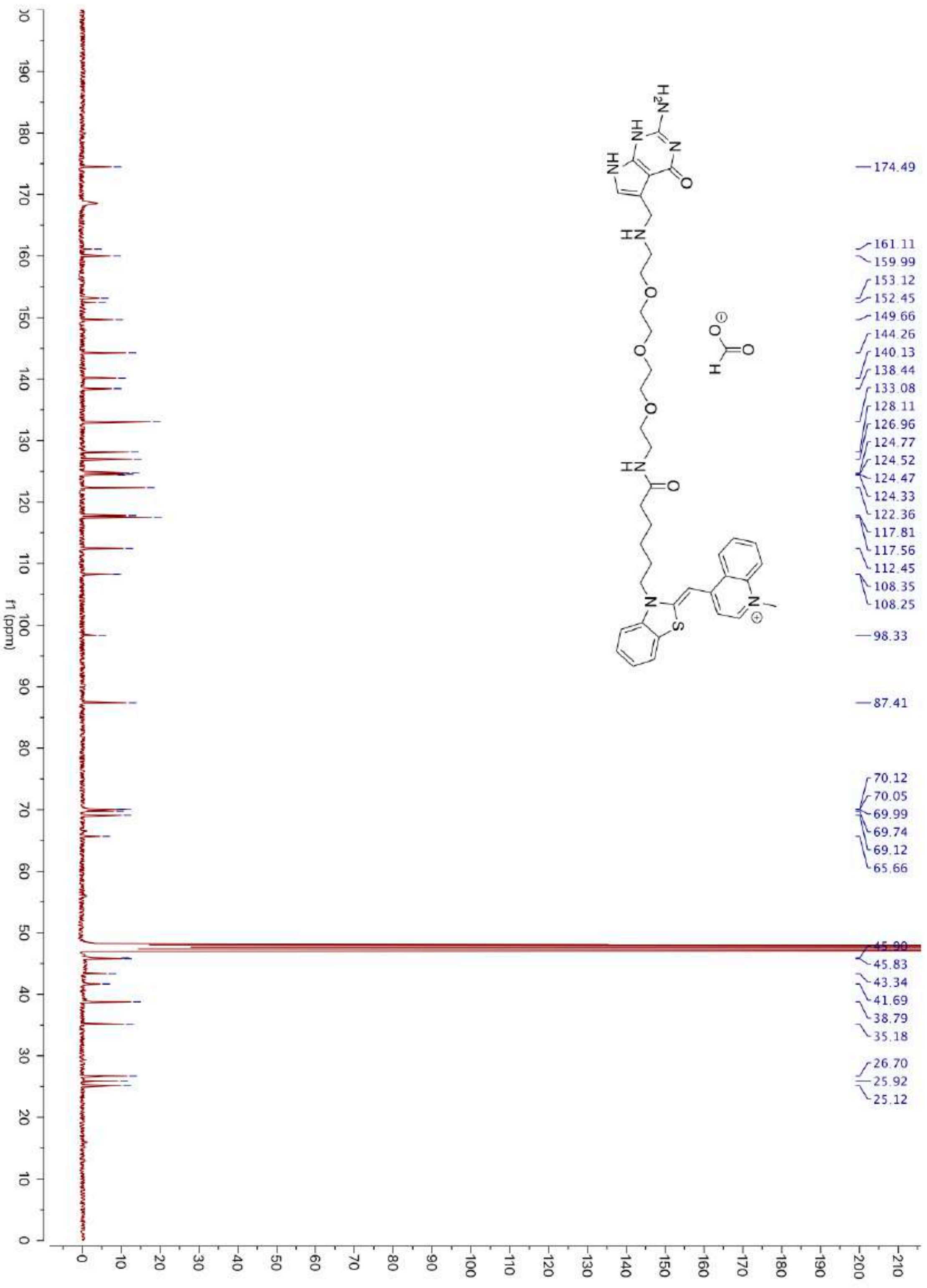
174.49
161.11
159.99
153.12
152.45
149.66
144.26
140.13
138.44
133.08
128.11
126.96
124.77
124.52
124.47
124.33
122.36
117.81
117.56
112.45
108.35
108.25
98.33
87.41
70.12
70.05
69.99
69.74
69.12
65.66
45.83
43.34
41.69
38.79
35.18
26.70
25.92
25.12
f1 (ppm)

5b

^{1}H NMR

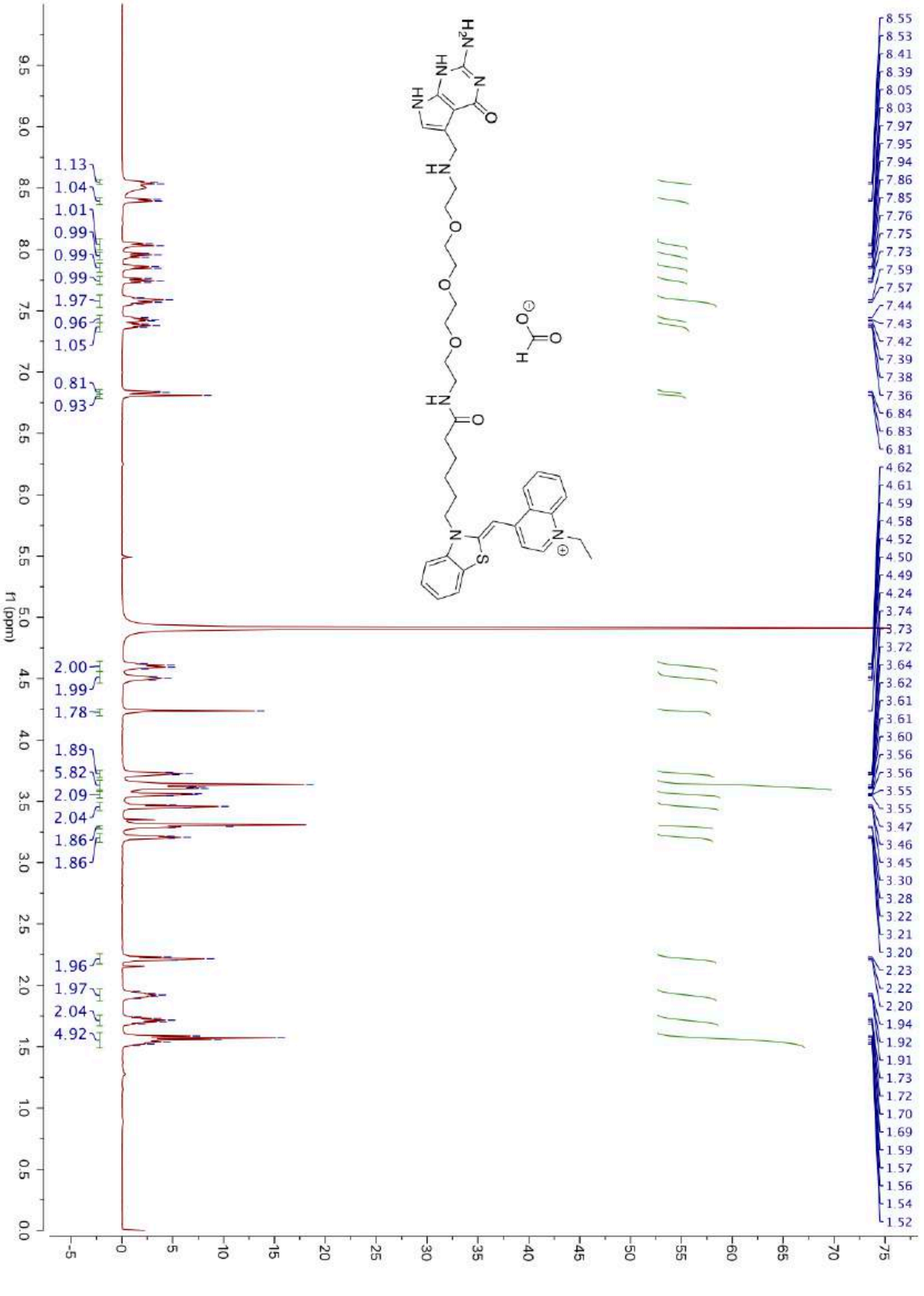
8.55
8.53
8.41
8.39
8.05
8.03
7.97
7.95
7.94
7.86
7.85
7.76
7.75
7.73
7.59
7.57
7.44
7.43
7.42
7.39
7.38
7.36
6.84
6.83
6.81
4.62
4.61
4.59
4.58
4.52
4.50
4.49
4.24
3.74
3.73
3.72
3.64
3.62
3.61
3.61
3.60
3.56
3.56
3.55
3.55
3.47
3.46
3.45
3.30
3.28
3.22
3.21
3.20
2.23
2.22
2.20
1.94
1.92
1.91
1.73
1.72
1.70
1.69
1.59
1.57
1.56
1.54
1.52
1.13
1.04
1.01
0.99
0.99
0.99
1.97
0.96
1.05
0.81
0.93
2.00
1.99
1.78
1.89
5.82
2.09
2.04
1.86
1.86
1.96
1.97
2.04
4.92
f1 (ppm)

5b

^{13}C NMR

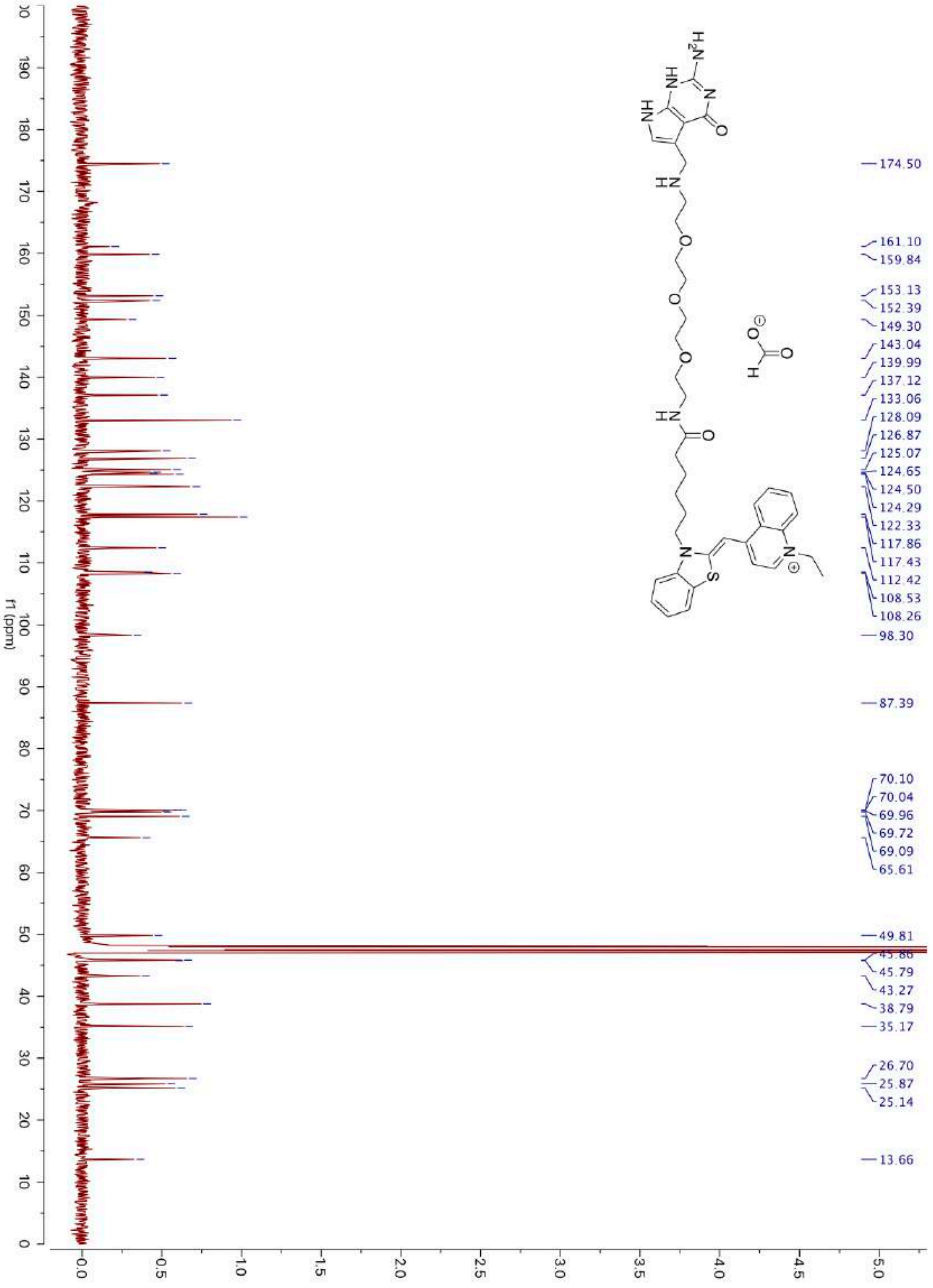
174.50
161.10
159.84
153.13
152.39
149.30
143.04
139.99
137.12
133.06
128.09
126.87
125.07
124.65
124.50
124.29
122.33
117.86
117.43
112.42
108.53
108.26
98.30
87.39
70.10
70.04
69.96
69.72
69.09
65.61
49.81
45.79
43.27
38.79
35.17
26.70
25.87
25.14
13.66
f1 (ppm)

5c

^{1}H NMR

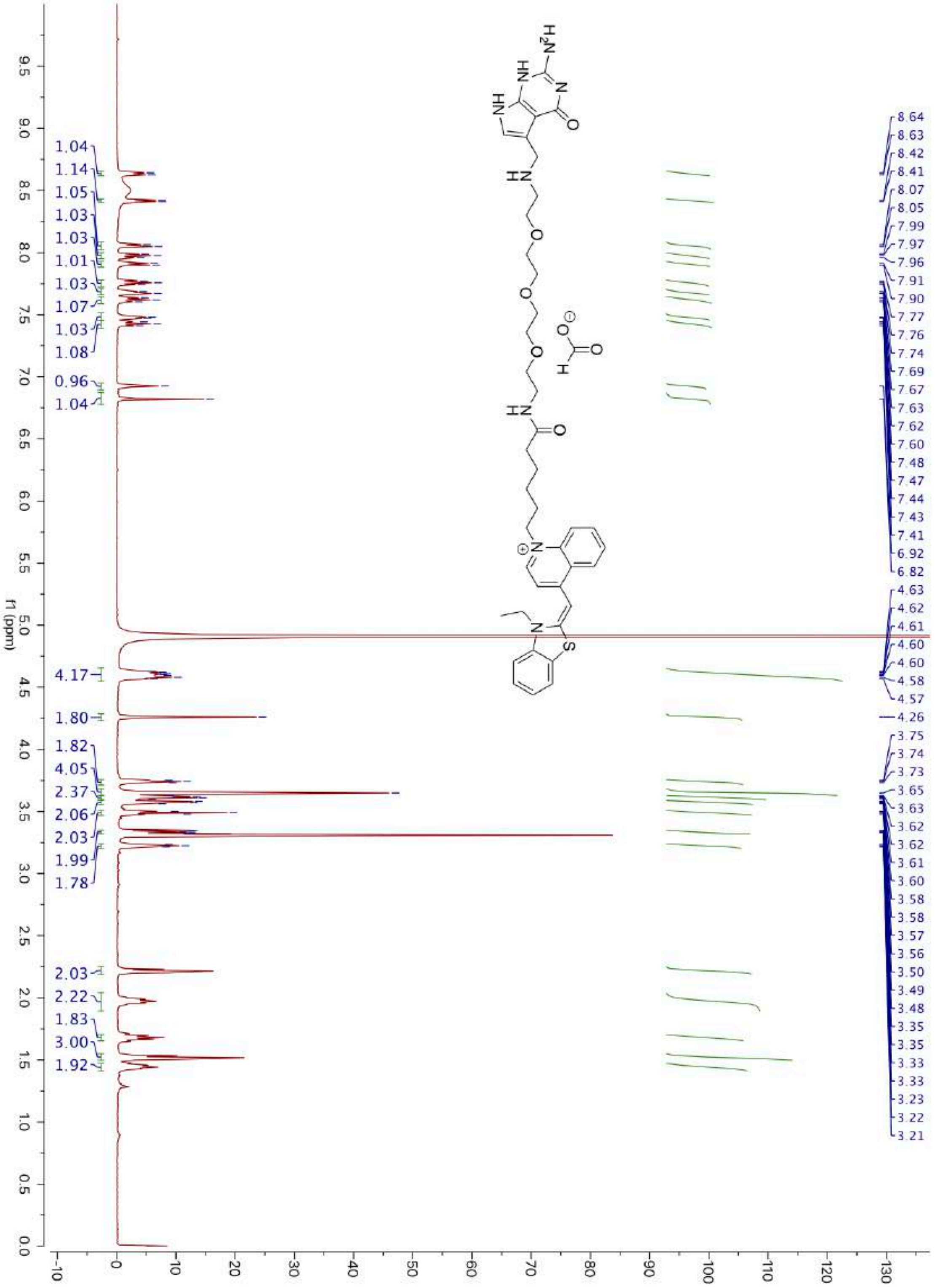

5c
^{13}C NMR

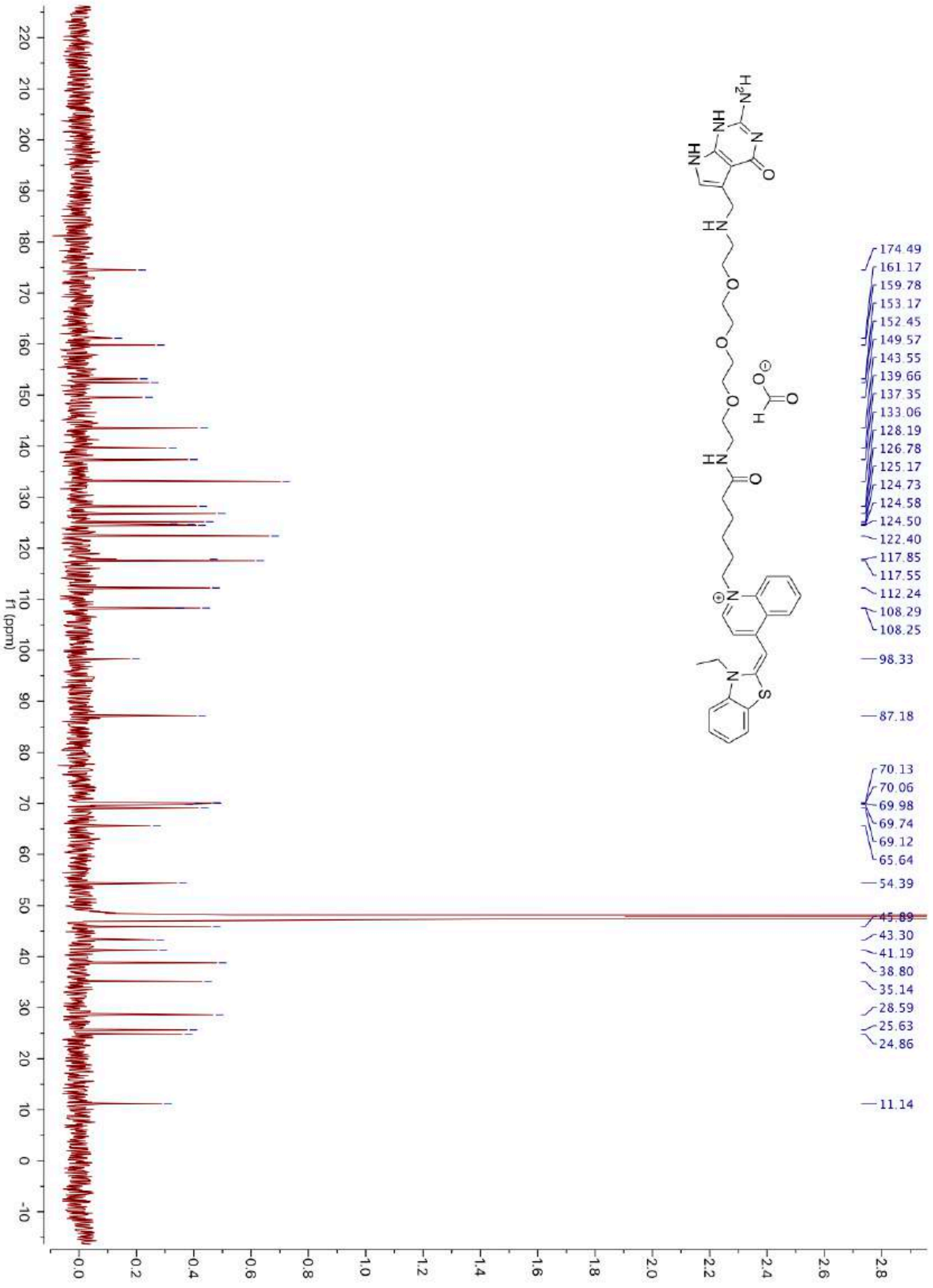
174.49
161.17
159.78
153.17
152.45
149.57
143.55
139.66
137.35
133.06
128.19
126.78
125.17
124.73
124.58
124.50
122.40
117.85
117.55
112.24
108.29
108.25
98.33
87.18
70.13
70.06
69.98
69.74
69.12
65.64
54.39
45.89
43.30
41.19
38.80
35.14
28.59
25.63
24.86
11.14
f1 (ppm)

5d

^{1}H NMR

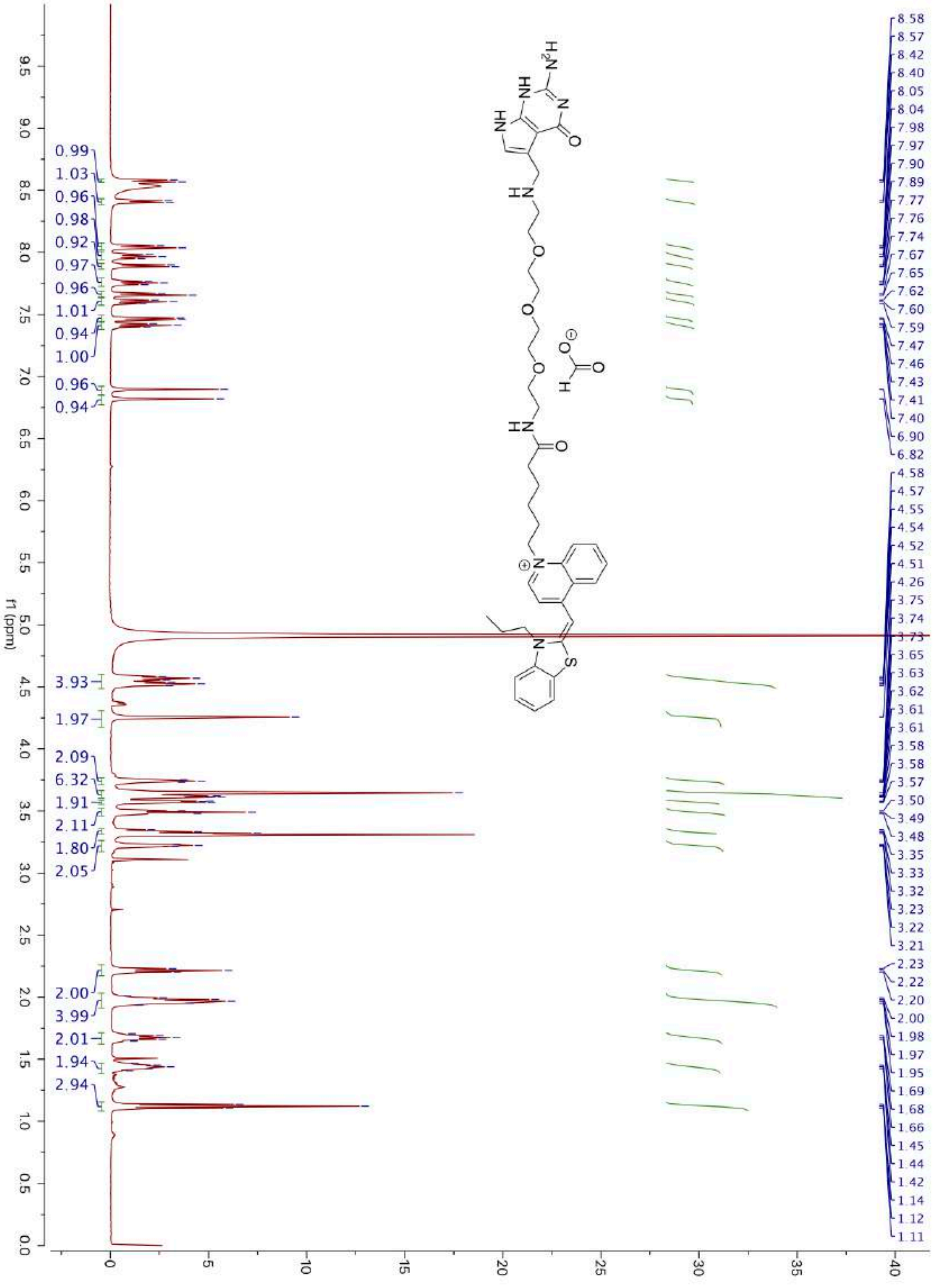

5d

^{13}C NMR

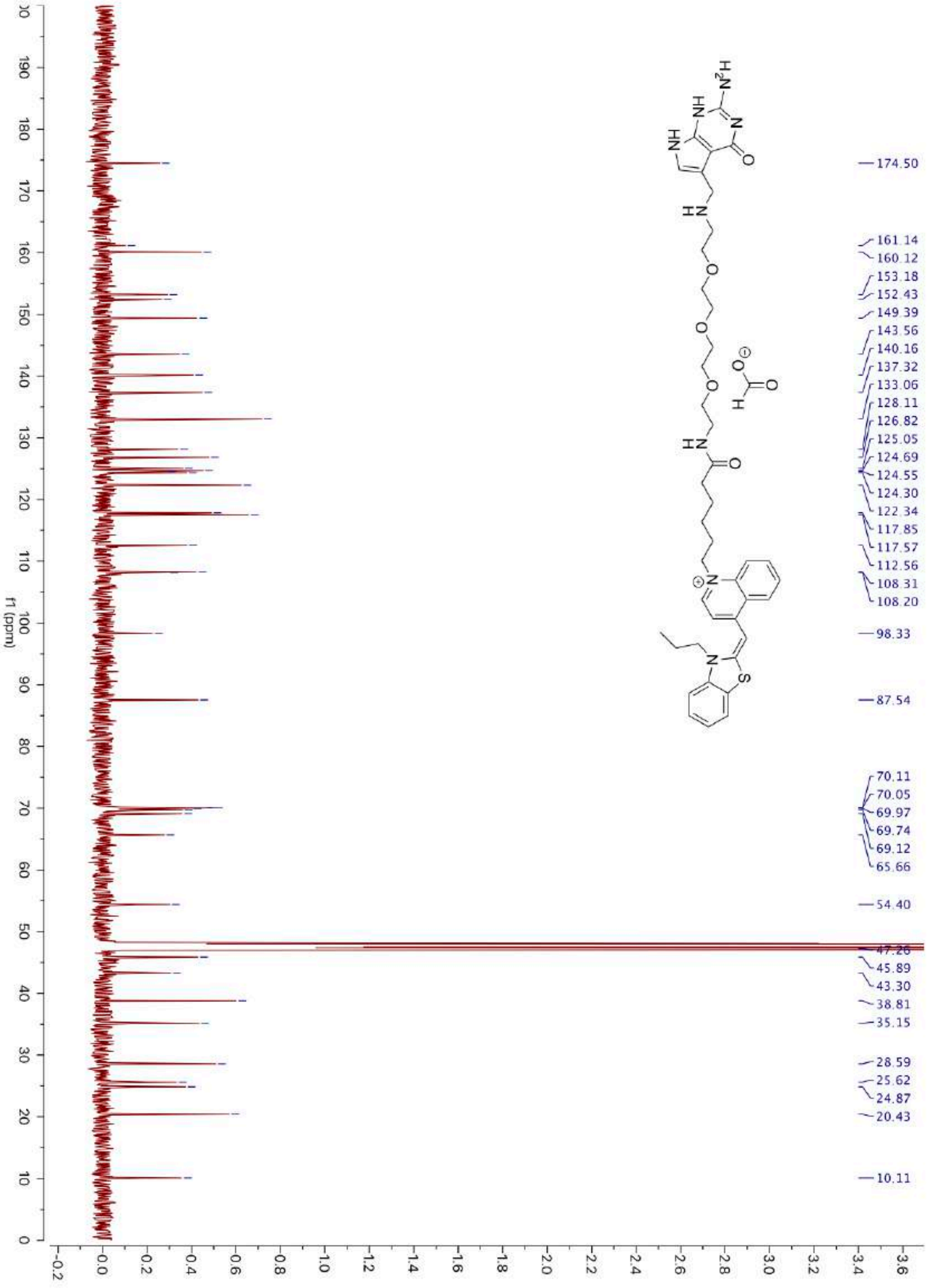
174.50
161.14
160.12
153.18
152.43
149.39
143.56
140.16
137.32
133.06
128.11
126.82
125.05
124.69
124.55
124.30
122.34
117.85
117.57
112.56
108.31
108.20
98.33
87.54
70.11
70.05
69.97
69.74
69.12
65.66
54.40
47.26
45.89
43.30
38.81
35.15
28.59
25.62
24.87
20.43
10.11
f1 (ppm)

5e

^{1}H NMR

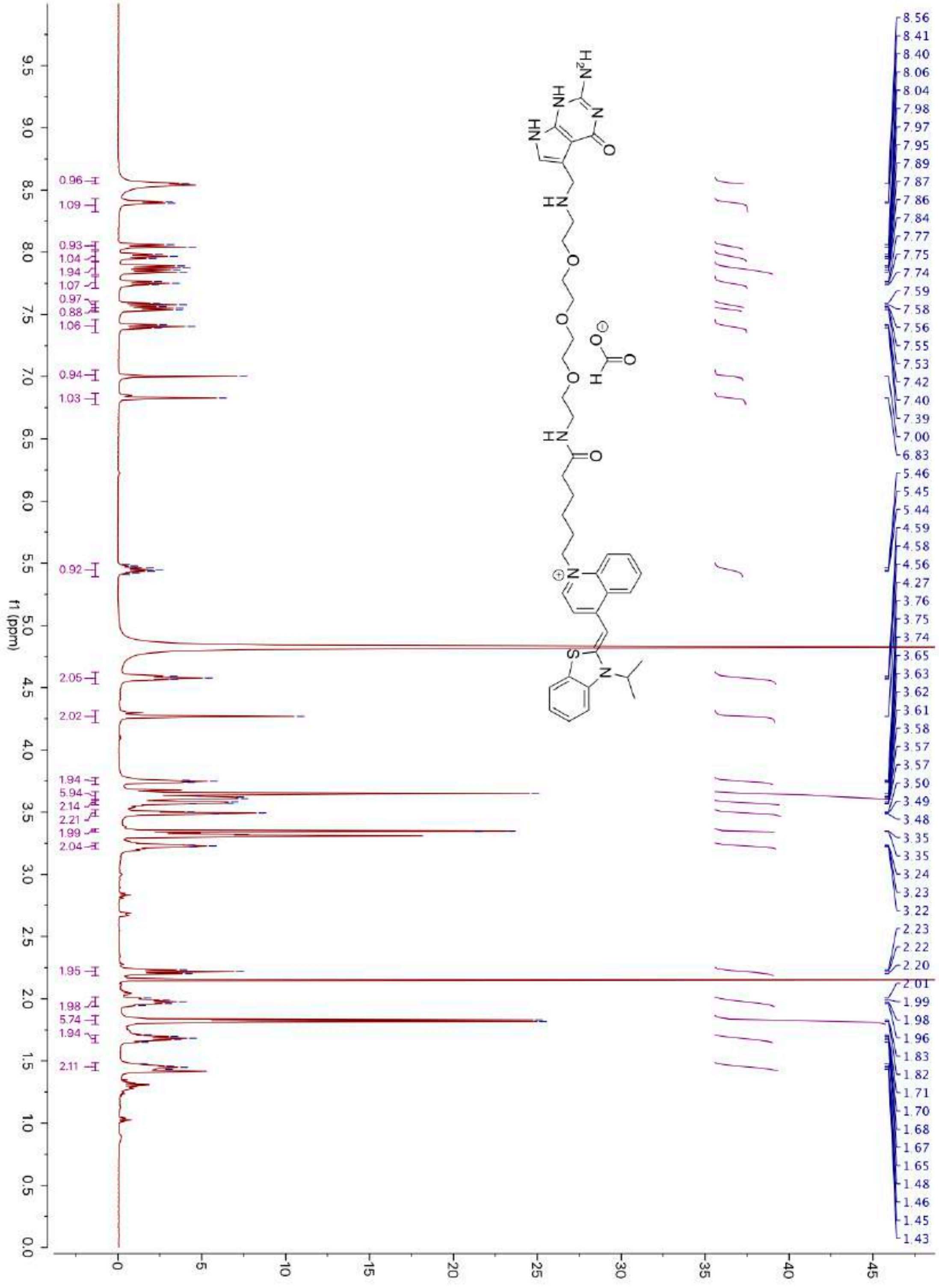

8.56 8.41 8.40 8.06 8.04 7.98 7.97 7.95 7.89 7.87 7.86 7.84 7.77 7.75 7.74 7.59 7.58 7.56 7.55 7.53 7.42 7.40 7.39 7.00 6.83 5.46 5.45 5.44 4.59 4.58 4.56 4.27 3.76 3.75 3.74 3.65 3.63 3.62 3.61 3.58 3.57 3.57 3.50 3.49 3.48 3.35 3.35 3.24 3.23 3.22 2.23 2.22 2.20 2.01 1.99 1.98 1.96 1.83 1.82 1.71 1.70 1.68 1.67 1.65 1.48 1.46 1.45 1.43
f1 (ppm)

5e

^{13}C NMR

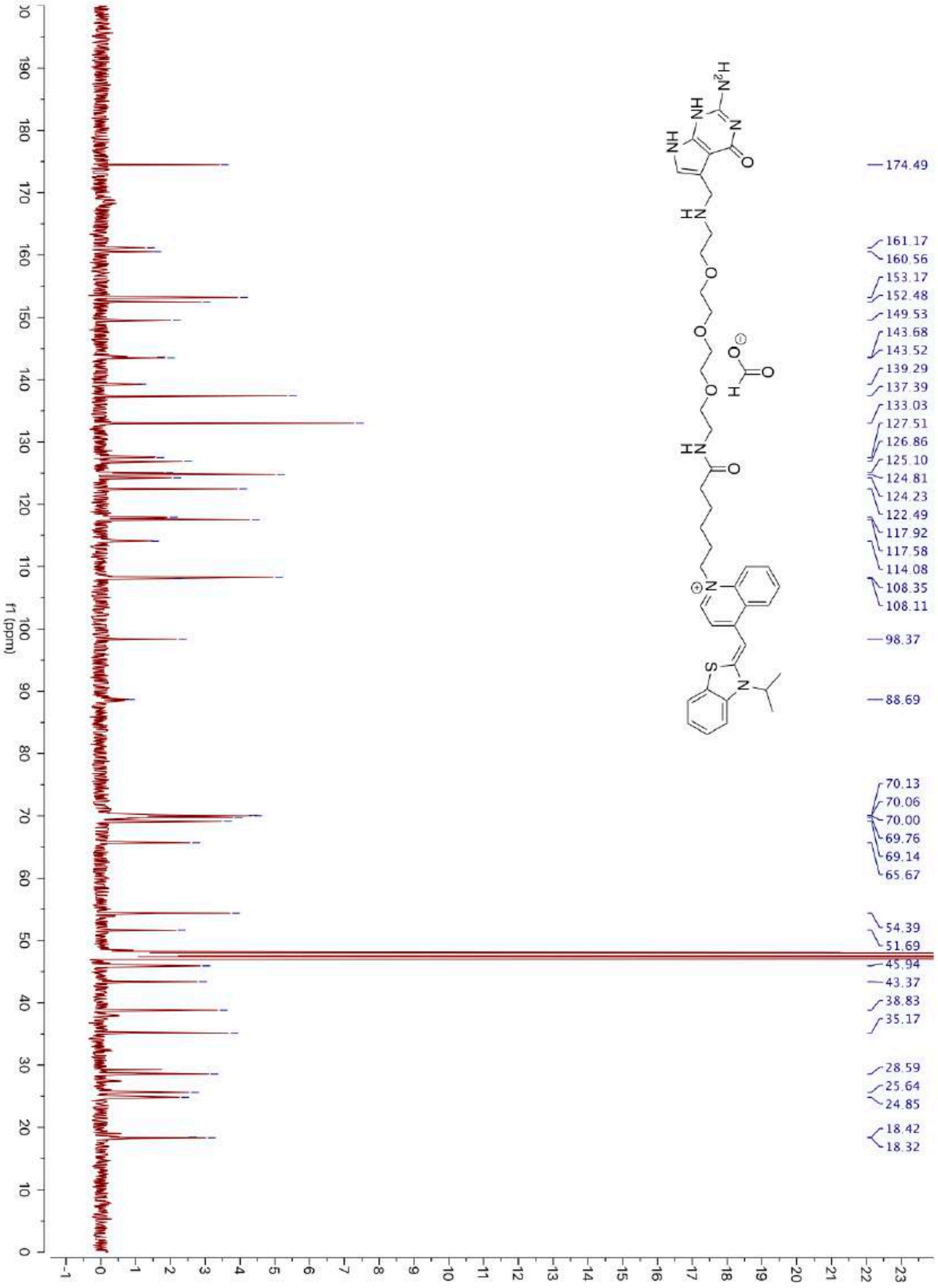
174.49
161.17
160.56
153.17
152.48
149.53
143.68
143.52
139.29
137.39
133.03
127.51
126.86
125.10
124.81
124.23
122.49
117.92
117.58
114.08
108.35
108.11
98.37
88.69
70.13
70.06
70.00
69.76
69.14
65.67
54.39
51.69
45.94
43.37
38.83
35.17
28.59
25.64
24.85
18.42
18.32
f1 (ppm)

5f

^{1}H NMR

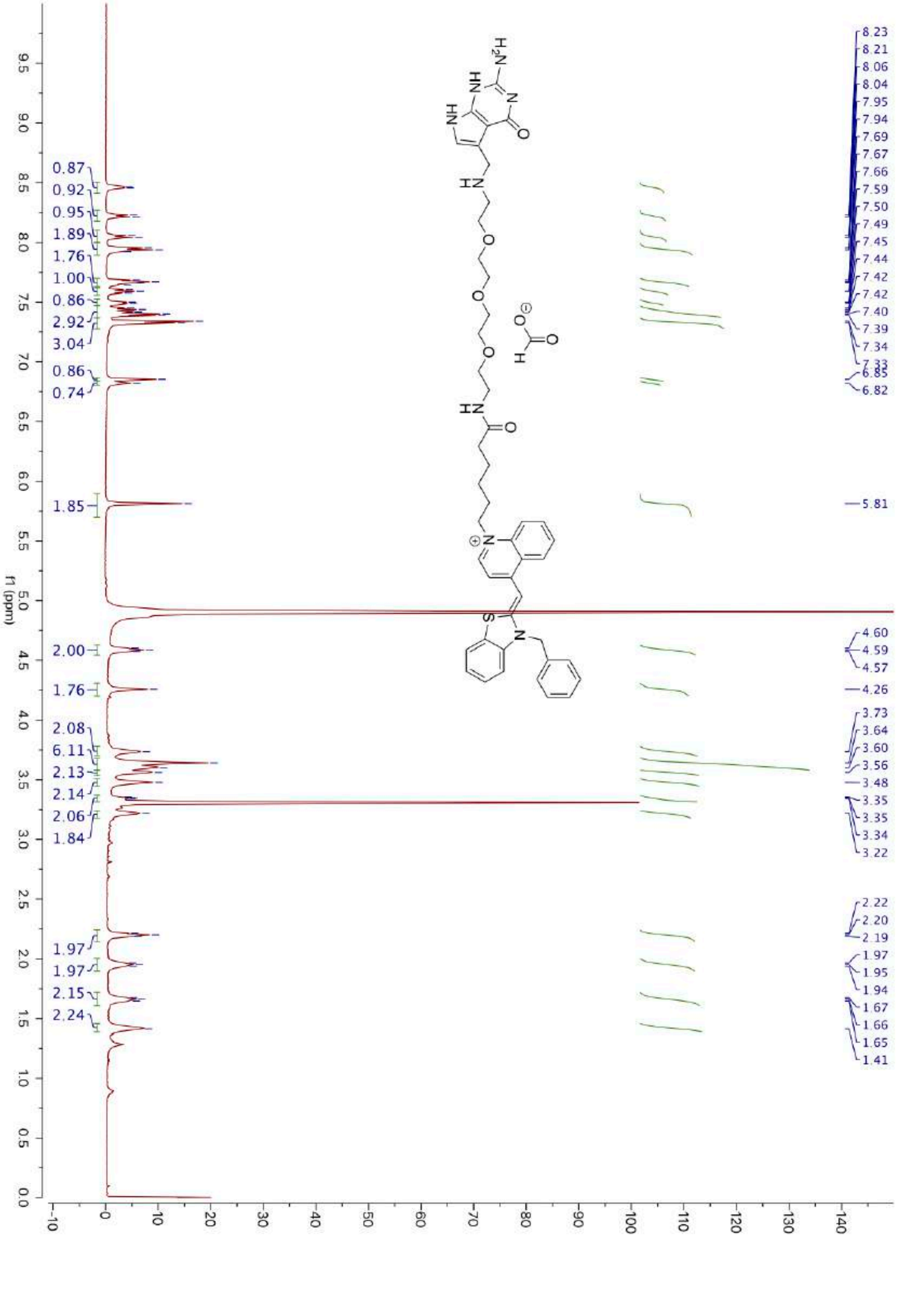

5f

^{13}C NMR

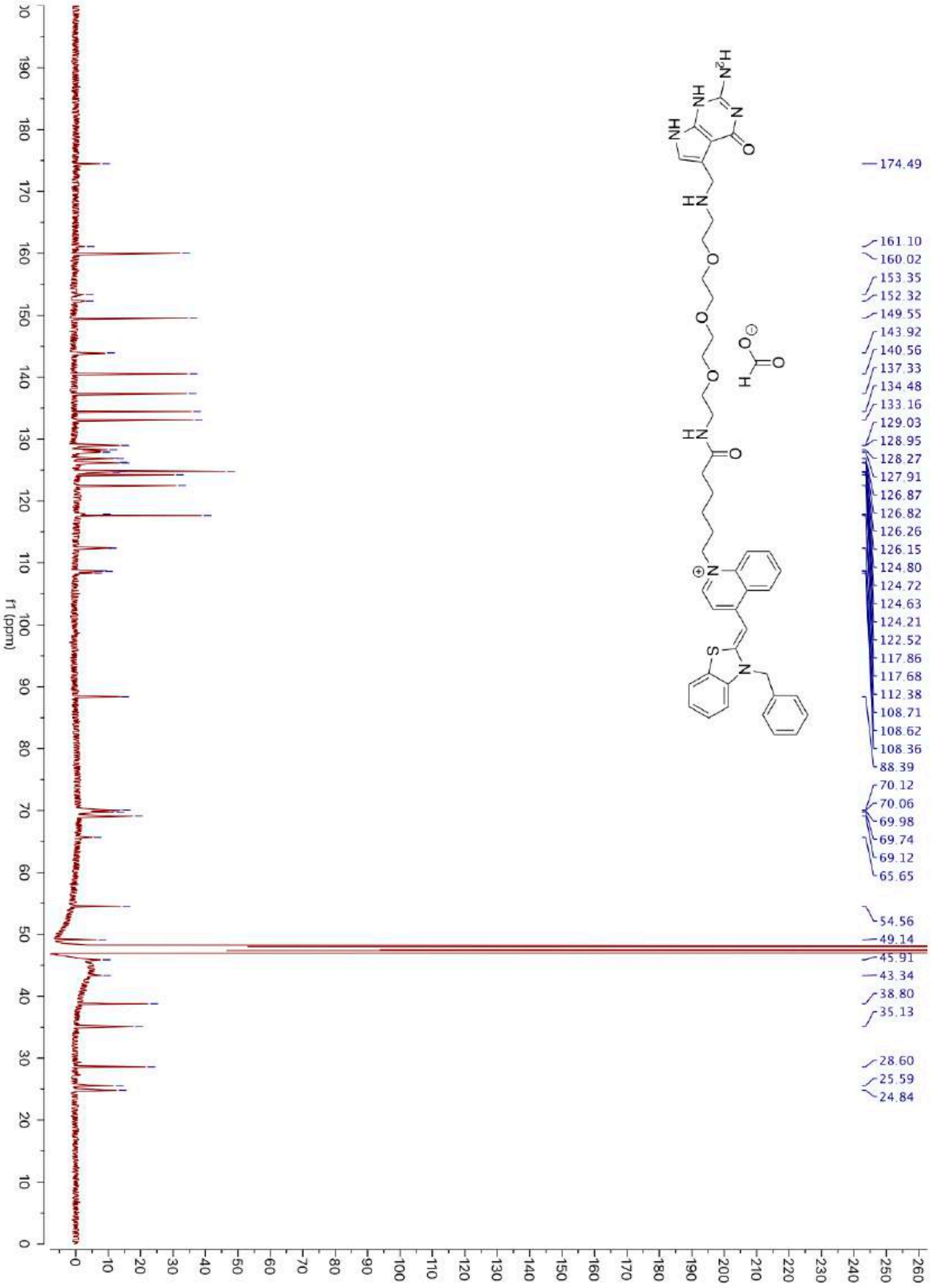

174.49
161.10
160.02
153.35
152.32
149.55
143.92
140.56
137.33
134.48
133.16
129.03
128.95
128.27
127.91
126.87
126.82
126.26
126.15
124.80
124.72
124.63
124.21
122.52
117.86
117.68
112.38
108.71
108.62
108.36
88.39
70.12
70.06
69.98
69.74
69.12
65.65
54.56
49.14
45.91
43.34
38.80
35.13
28.60
25.59
24.84
f1 (ppm)

6a
^{1}H NMR

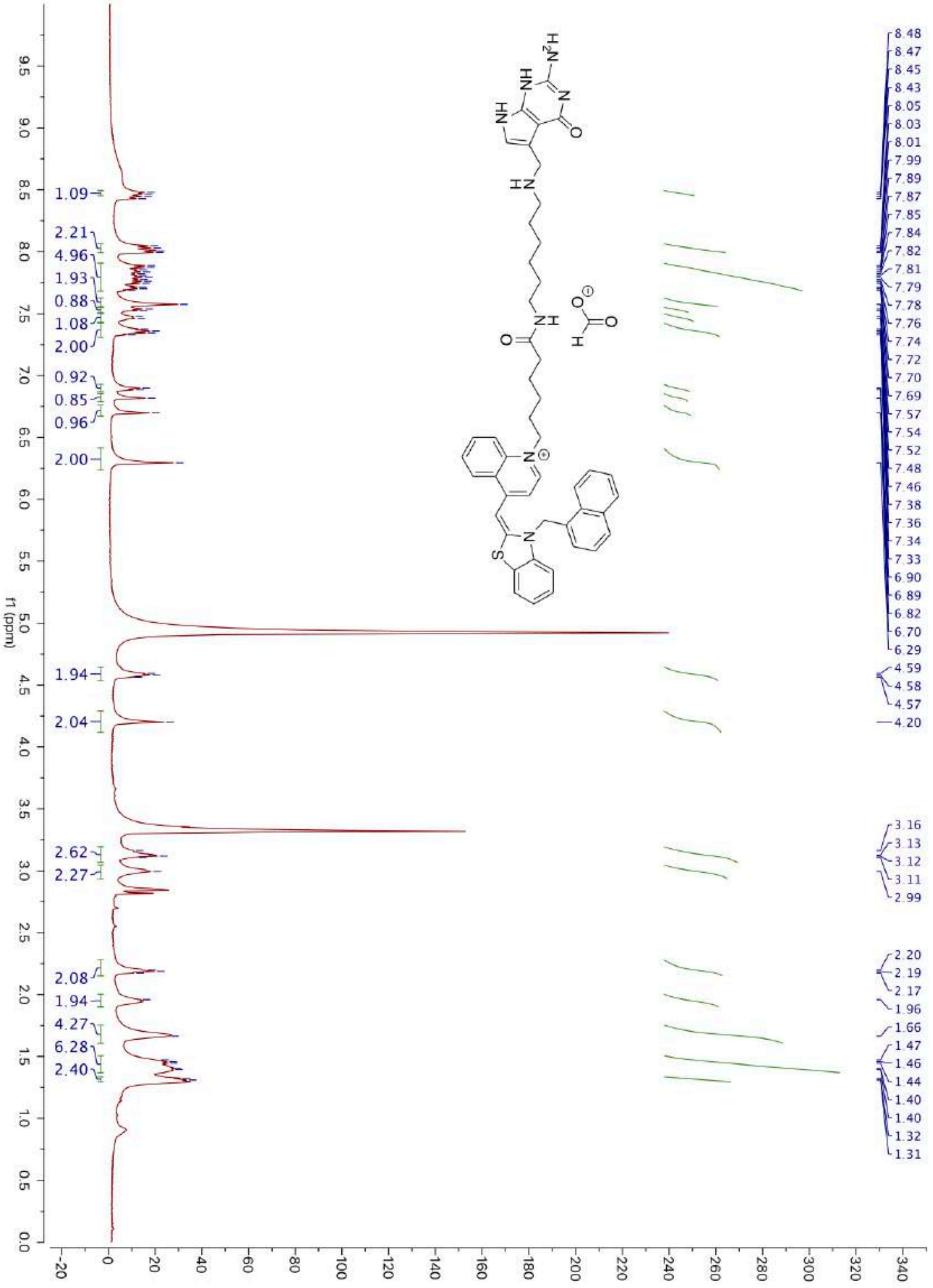
8.48
8.47
8.45
8.43
8.05
8.03
8.01
7.99
7.89
7.87
7.85
7.84
7.82
7.81
7.79
7.78
7.76
7.74
7.72
7.70
7.69
7.57
7.54
7.52
7.48
7.46
7.38
7.36
7.34
7.33
6.90
6.89
6.82
6.70
6.29
4.59
4.58
4.57
4.20
3.16
3.13
3.12
3.11
2.99
2.20
2.19
2.17
1.96
1.66
1.47
1.46
1.44
1.40
1.40
1.32
1.31
1.09
2.21
4.96
1.93
0.88
1.08
2.00
0.92
0.85
0.96
2.00
1.94
2.04
2.62
2.27
2.08
1.94
4.27
6.28
2.40
f1 (ppm)

6a

^{13}C NMR

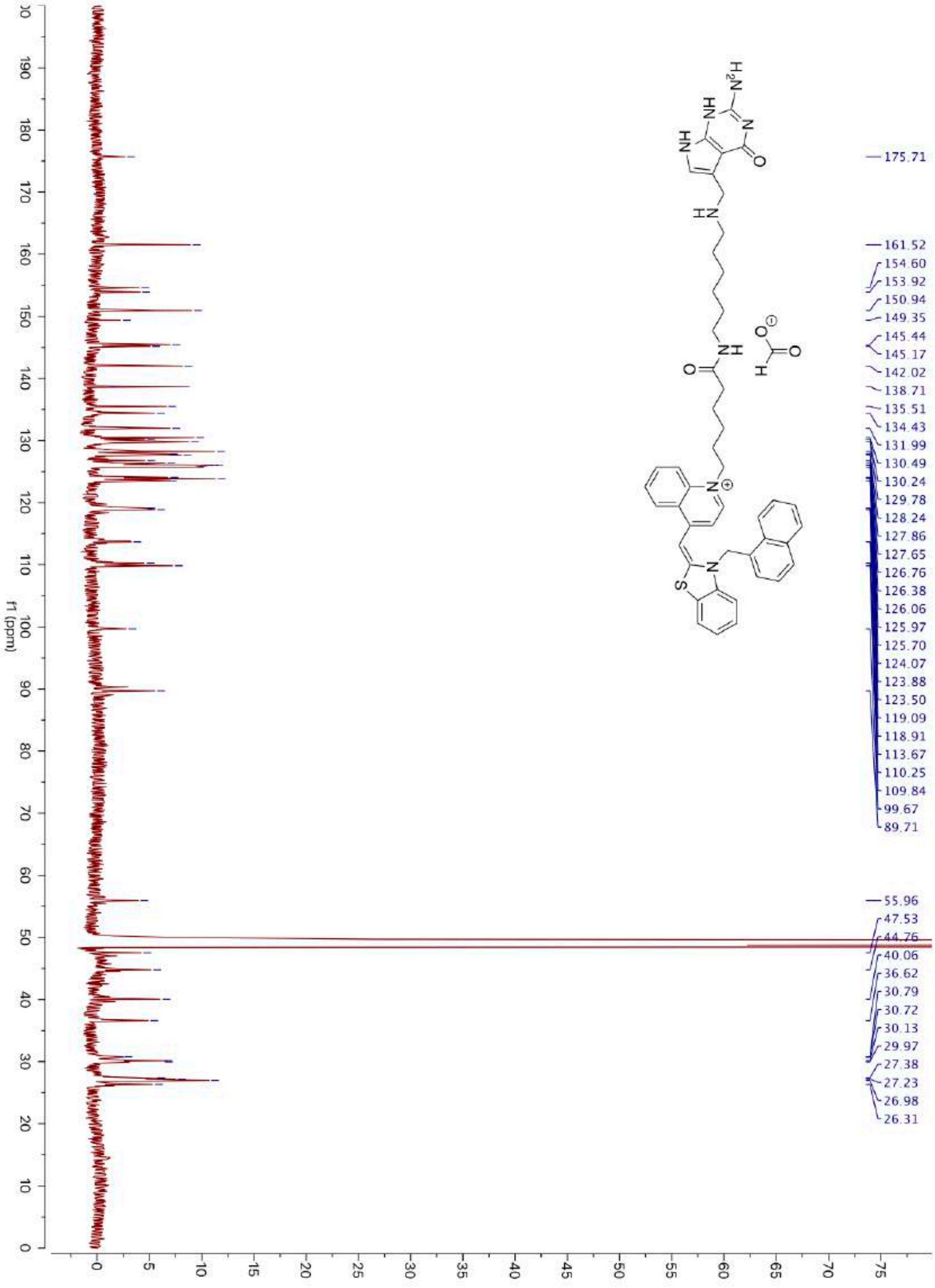
175.71
161.52
154.60
153.92
150.94
149.35
145.44
145.17
142.02
138.71
135.51
134.43
131.99
130.49
130.24
129.78
128.24
127.86
127.65
126.76
126.38
126.06
125.97
125.70
124.07
123.88
123.50
119.09
118.91
113.67
110.25
109.84
99.67
89.71
55.96
47.53
44.76
40.06
36.62
30.79
30.72
30.13
29.97
27.38
27.23
26.98
26.31
f1 (ppm)

6b

^{1}H NMR

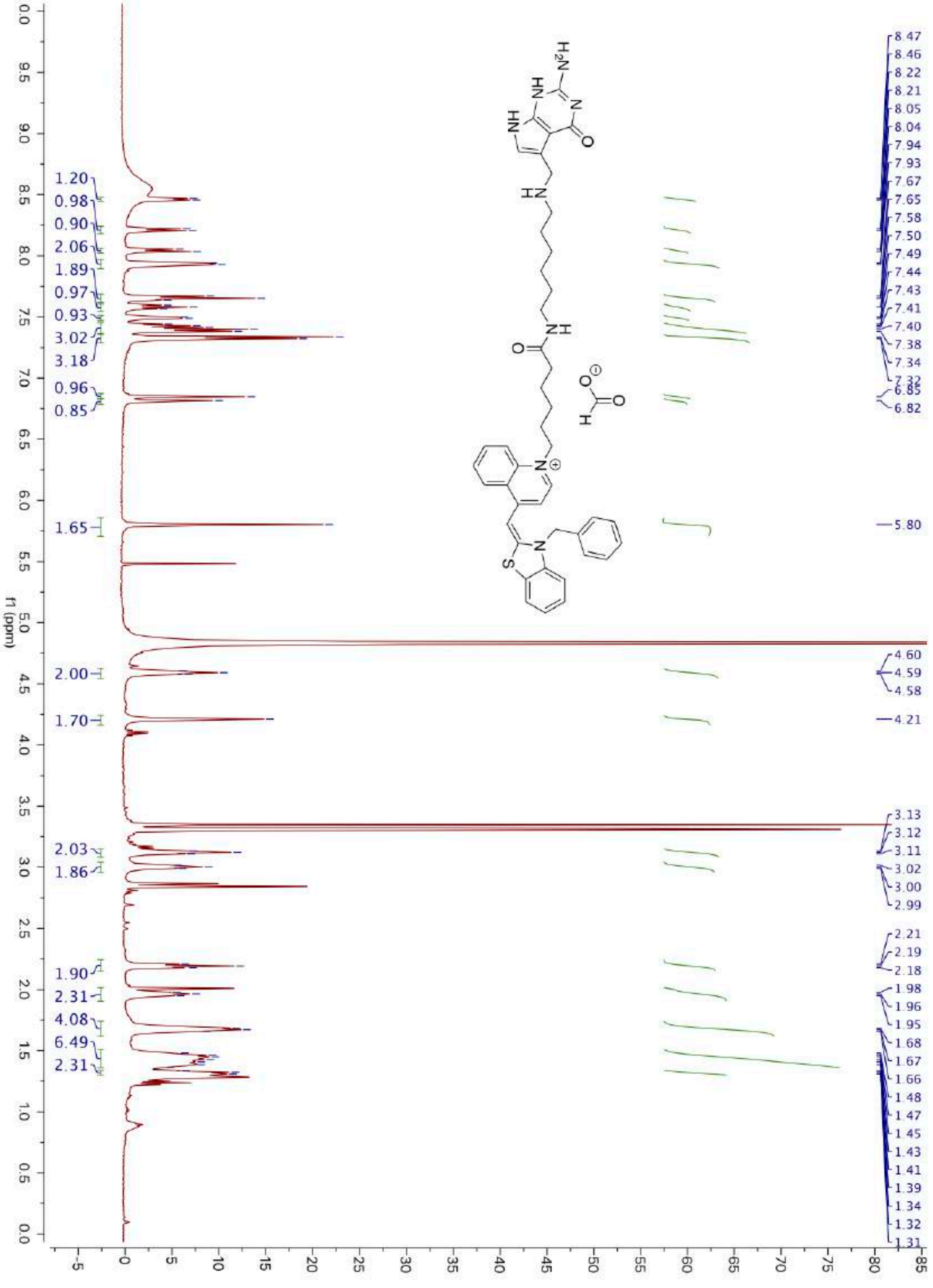

8.47
8.46
8.22
8.21
8.05
8.04
7.94
7.93
7.67
7.65
7.58
7.50
7.49
7.44
7.43
7.41
7.40
7.38
7.34
7.32
6.85
6.82
5.80
4.60
4.59
4.58
4.21
3.13
3.12
3.11
3.02
3.00
2.99
2.21
2.19
2.18
1.98
1.96
1.95
1.68
1.67
1.66
1.48
1.47
1.45
1.43
1.41
1.39
1.34
1.32
1.31
1.20
0.98
0.90
2.06
1.89
0.97
0.93
3.02
3.18
0.96
0.85
1.65
2.00
1.70
2.03
1.86
1.90
2.31
4.08
6.49
2.31
f1 (ppm)

6a
^{13}C NMR

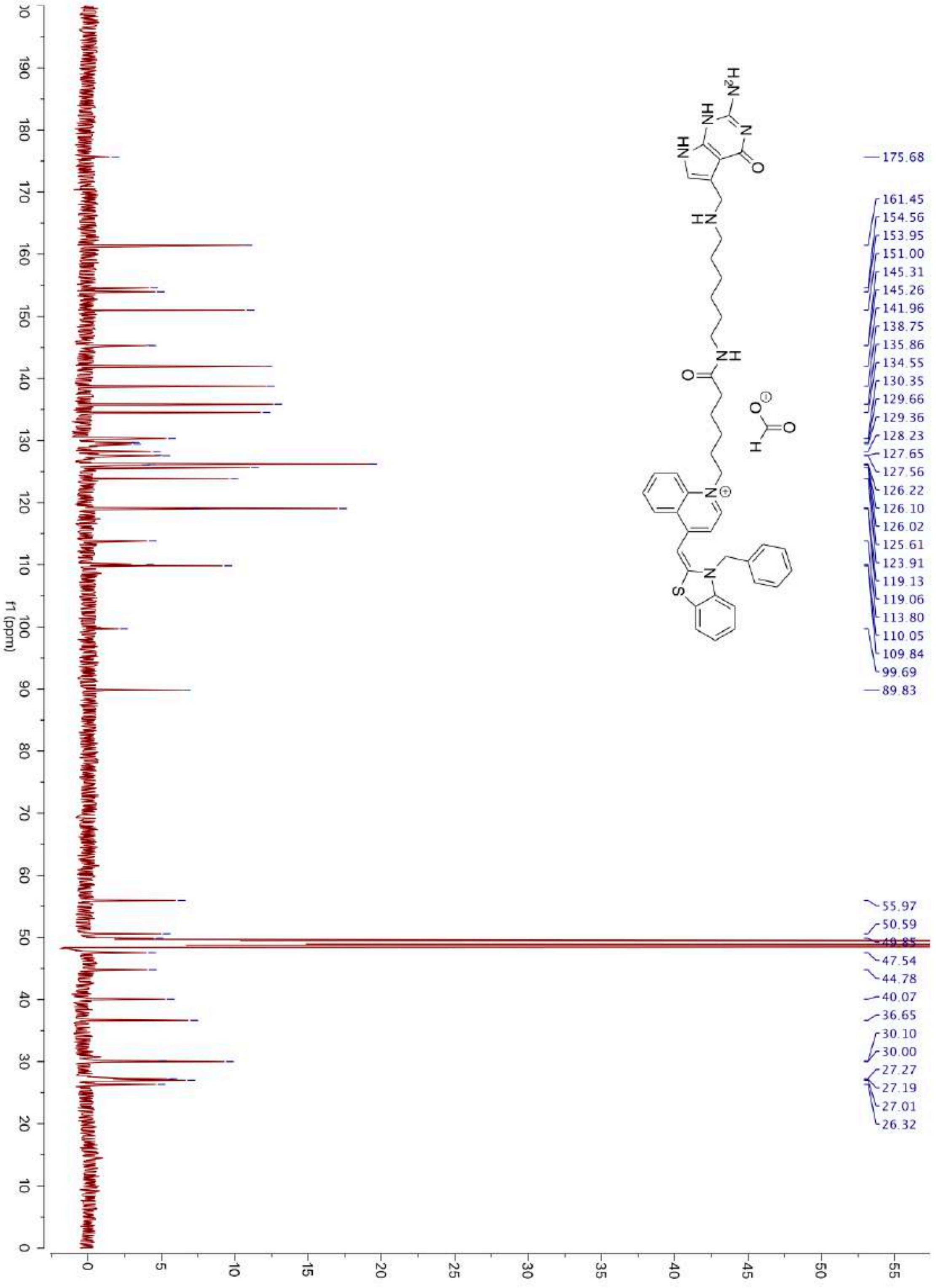

175.68
161.45
154.56
153.95
151.00
145.31
145.26
141.96
138.75
135.86
134.55
130.35
129.66
129.36
128.23
127.65
127.56
126.22
126.10
126.02
125.61
123.91
119.13
119.06
113.80
110.05
109.84
99.69
89.83
55.97
50.59
49.85
47.54
44.78
40.07
36.65
30.10
30.00
27.27
27.19
27.01
26.32
f1 (ppm)

6c

^{1}H NMR

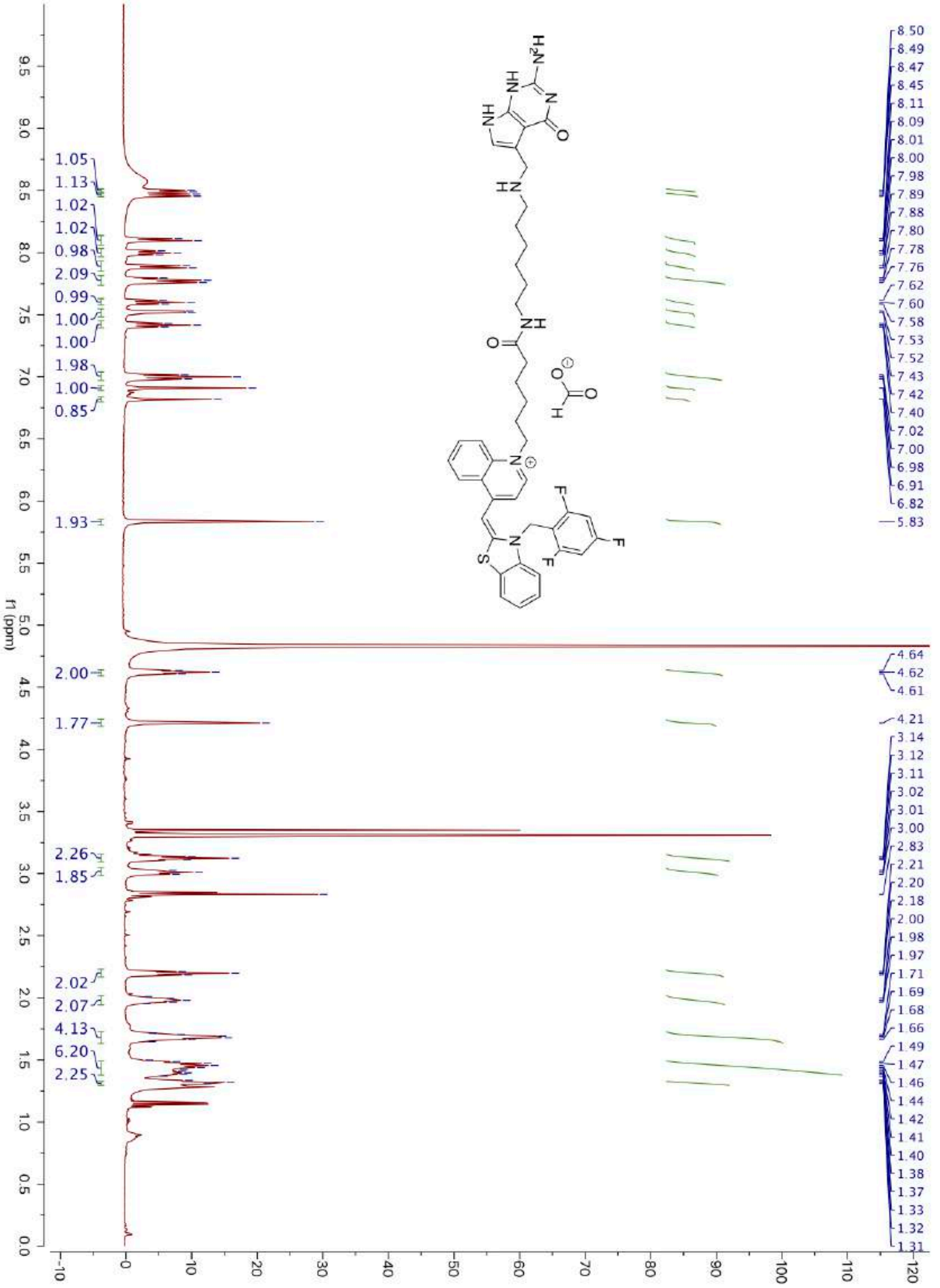

f1 (ppm)
8.50 8.49 8.47 8.45 8.11 8.09 8.01 8.00 7.98 7.89 7.88 7.80 7.78 7.76 7.62 7.60 7.58 7.53 7.52 7.43 7.42 7.40 7.02 7.00 6.98 6.91 6.82
5.83
4.64 4.62 4.61
4.21
3.14 3.12 3.11 3.02 3.01 3.00 2.83 2.21 2.20 2.18 2.00 1.98 1.97 1.71 1.69 1.68 1.66 1.49 1.47 1.46 1.44 1.42 1.41 1.40 1.38 1.37 1.33 1.32 1.31
1.05 1.13 1.02 1.02 0.98 2.09 0.99 1.00 1.00 1.98 1.00 0.85
1.93
2.00
1.77
2.26 1.85
2.02 2.07 4.13 6.20 2.25

6c
^{13}C NMR

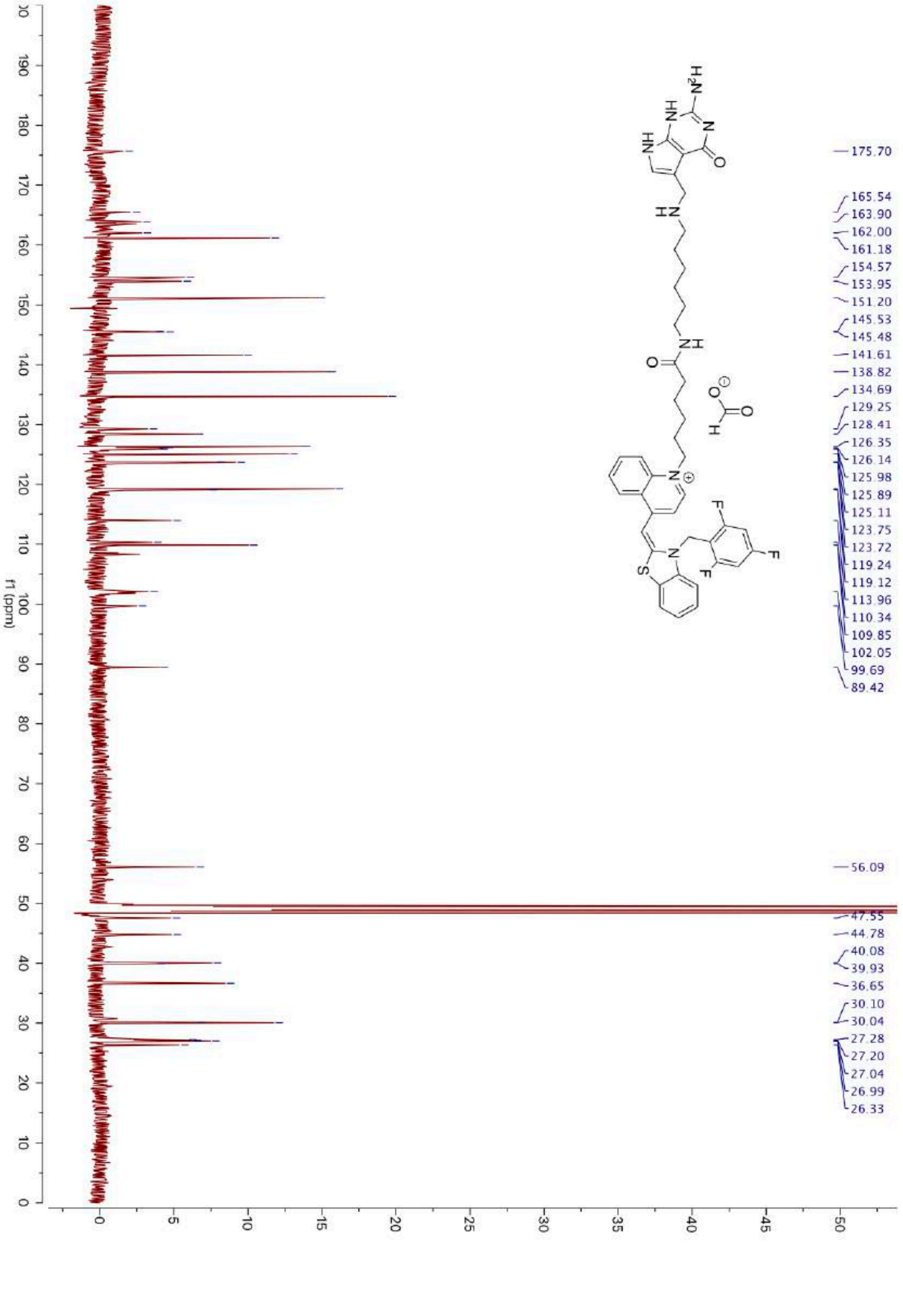
175.70
165.54
163.90
162.00
161.18
154.57
153.95
151.20
145.53
145.48
141.61
138.82
134.69
129.25
128.41
126.35
126.14
125.98
125.89
125.11
123.75
123.72
119.24
119.12
113.96
110.34
109.85
102.05
99.69
89.42
56.09
47.55
44.78
40.08
39.93
36.65
30.10
30.04
27.28
27.20
27.04
26.99
26.33
f1 (ppm)

6d

^1H NMR

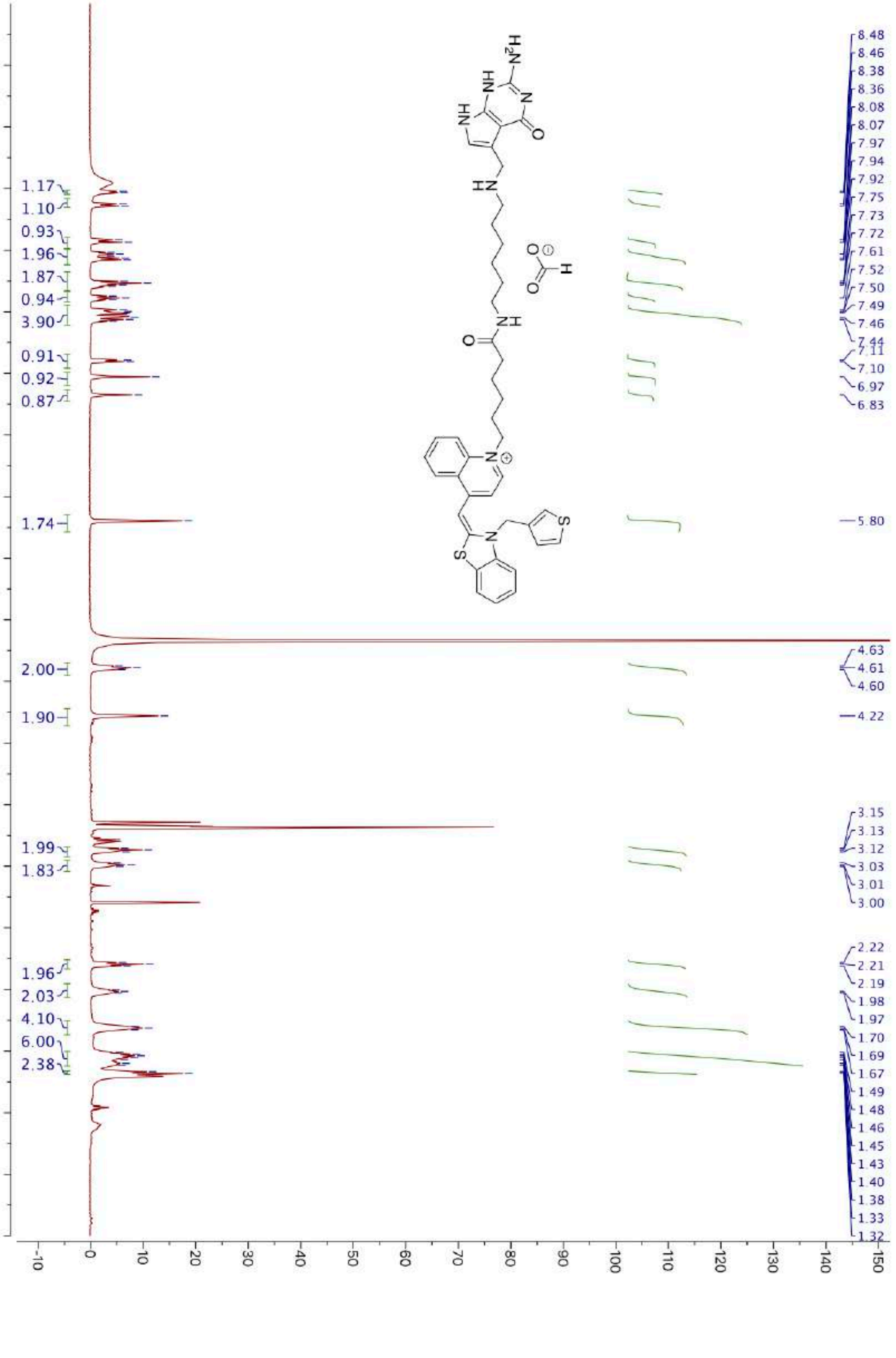
8.48
8.46
8.38
8.36
8.08
8.07
7.97
7.94
7.92
7.75
7.73
7.72
7.61
7.52
7.50
7.49
7.46
7.44
7.11
7.10
6.97
6.83
5.80
4.63
4.61
4.60
4.22
3.15
3.13
3.12
3.03
3.01
3.00
2.22
2.21
2.19
1.98
1.97
1.70
1.69
1.67
1.49
1.48
1.46
1.45
1.43
1.40
1.38
1.33
1.32
1.17
1.10
0.93
1.96
1.87
0.94
3.90
0.91
0.92
0.87
1.74
2.00
1.90
1.99
1.83
1.96
2.03
4.10
6.00
2.38
f1 (ppm)

6d

^{13}C NMR

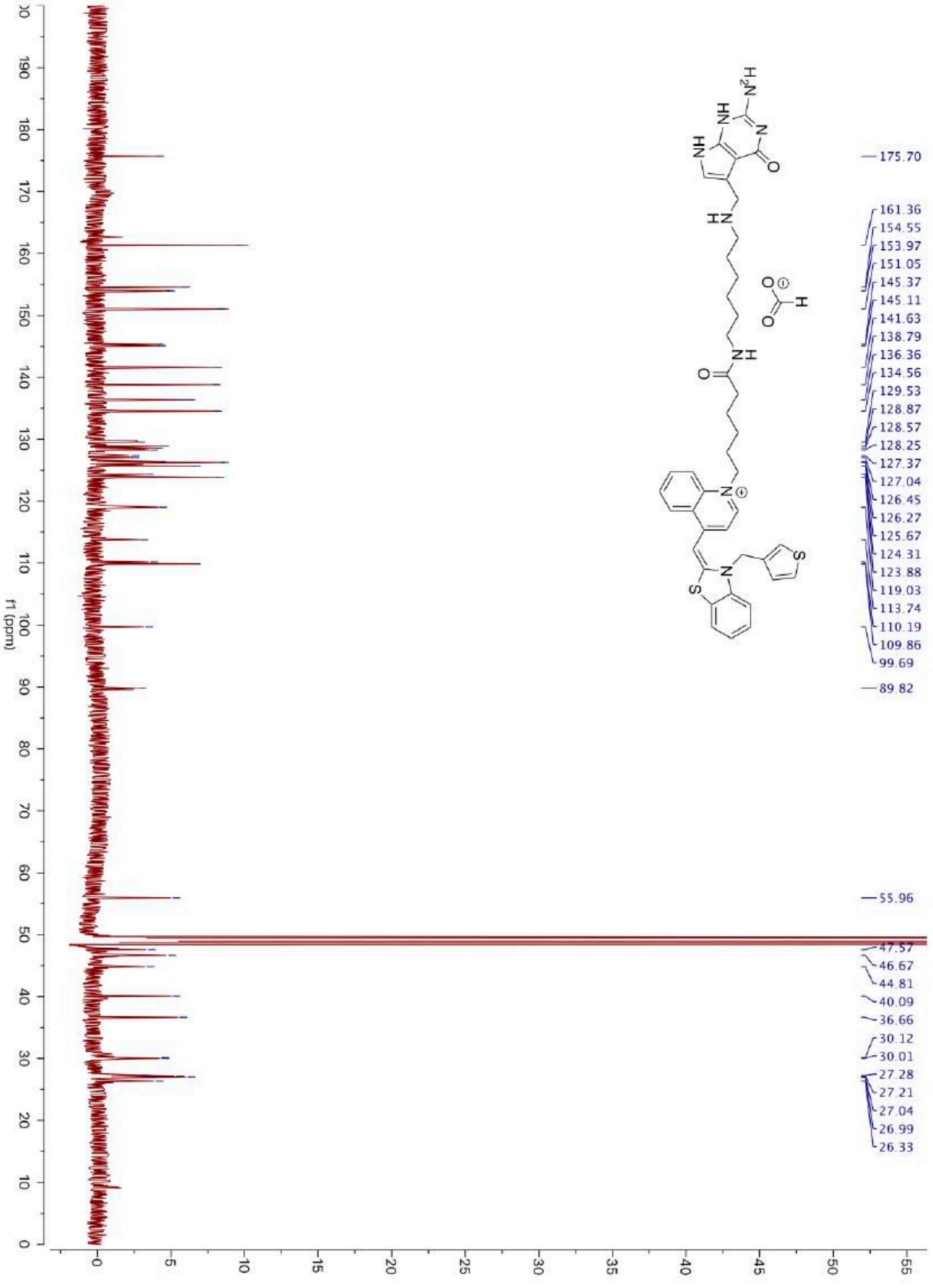
175.70
161.36
154.55
153.97
151.05
145.37
145.11
141.63
138.79
136.36
134.56
129.53
128.87
128.57
128.25
127.37
127.04
126.45
126.27
125.67
124.31
123.88
119.03
113.74
110.19
109.86
99.69
89.82
55.96
47.57
46.67
44.81
40.09
36.66
30.12
30.01
27.28
27.21
27.04
26.99
26.33
f1 (ppm)

6e

^{1}H NMR

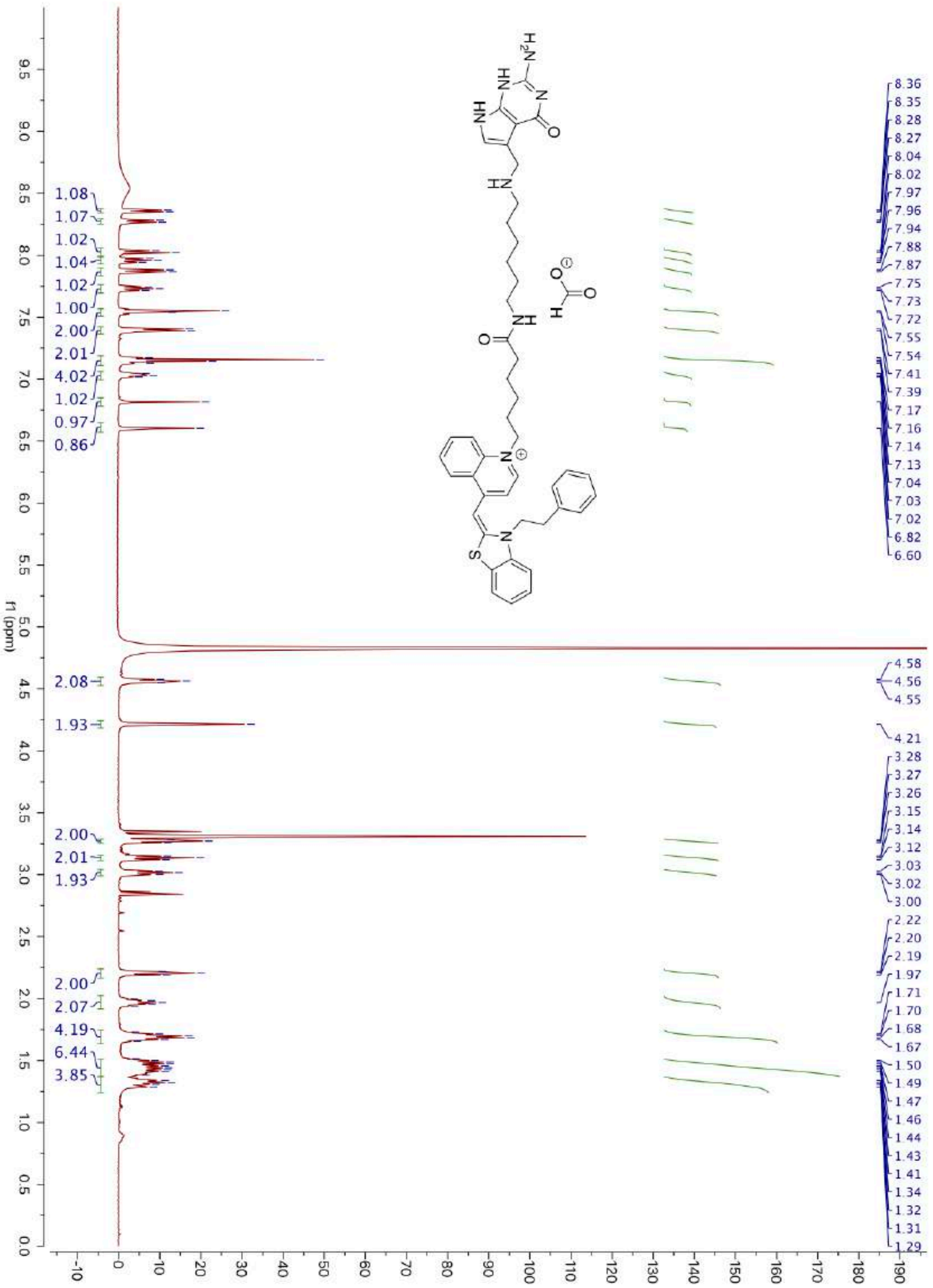

8.36
8.35
8.28
8.27
8.04
8.02
7.97
7.96
7.94
7.88
7.87
7.75
7.73
7.72
7.55
7.54
7.41
7.39
7.17
7.16
7.14
7.13
7.04
7.03
7.02
6.82
6.60
4.58
4.56
4.55
4.21
3.28
3.27
3.26
3.15
3.14
3.12
3.03
3.02
3.00
2.22
2.20
2.19
1.97
1.71
1.70
1.68
1.67
1.50
1.49
1.47
1.46
1.44
1.43
1.41
1.34
1.32
1.31
1.29
f1 (ppm)

6e
^{13}C NMR

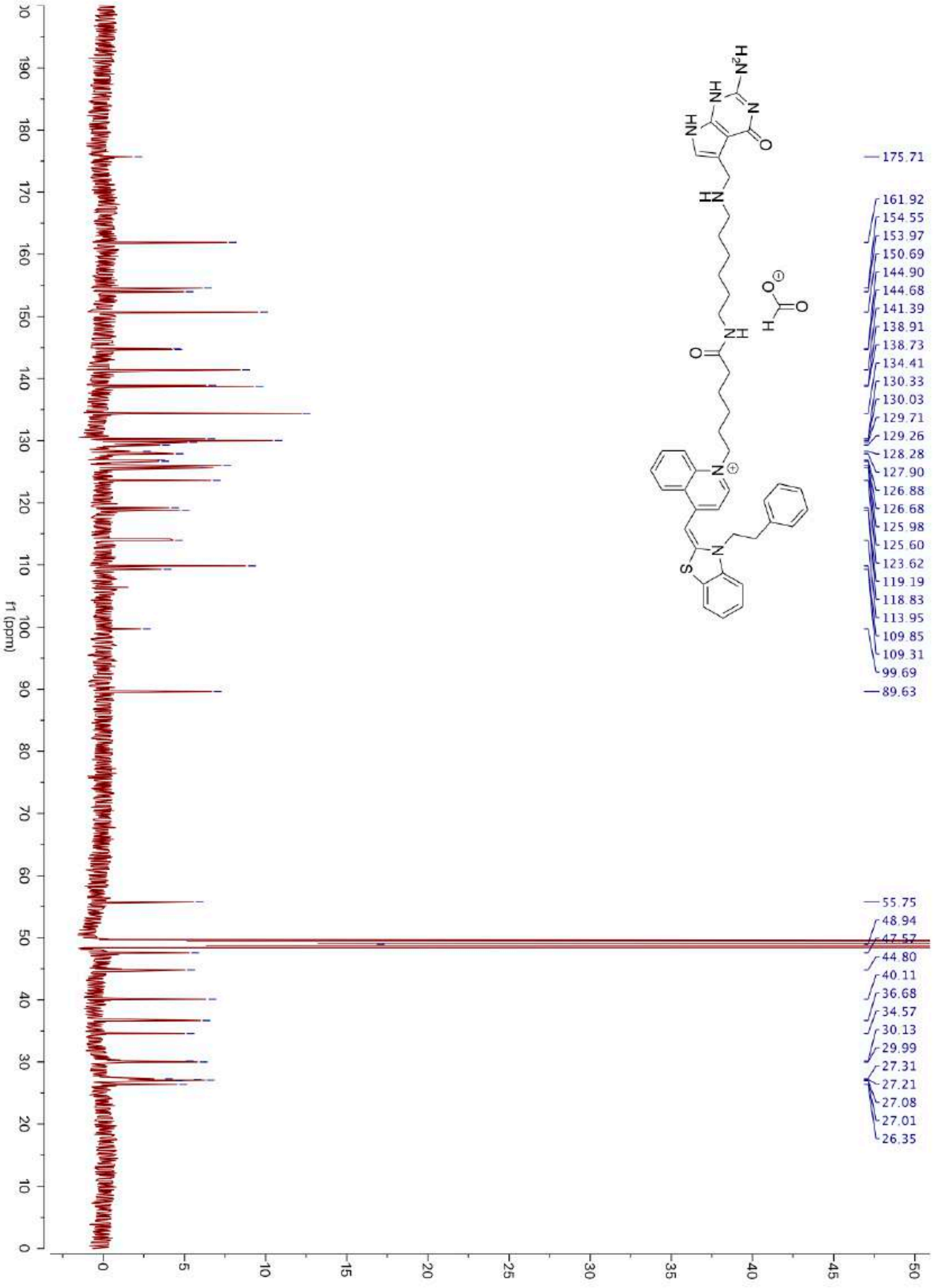
175.71
161.92
154.55
153.97
150.69
144.90
144.68
141.39
138.91
138.73
134.41
130.33
130.03
129.71
129.26
128.28
127.90
126.88
126.68
125.98
125.60
123.62
119.19
118.83
113.95
109.85
109.31
99.69
89.63
55.75
48.94
47.57
44.80
40.11
36.68
34.57
30.13
29.99
27.31
27.21
27.08
27.01
26.35
f1 (ppm)

6f

^{1}H NMR

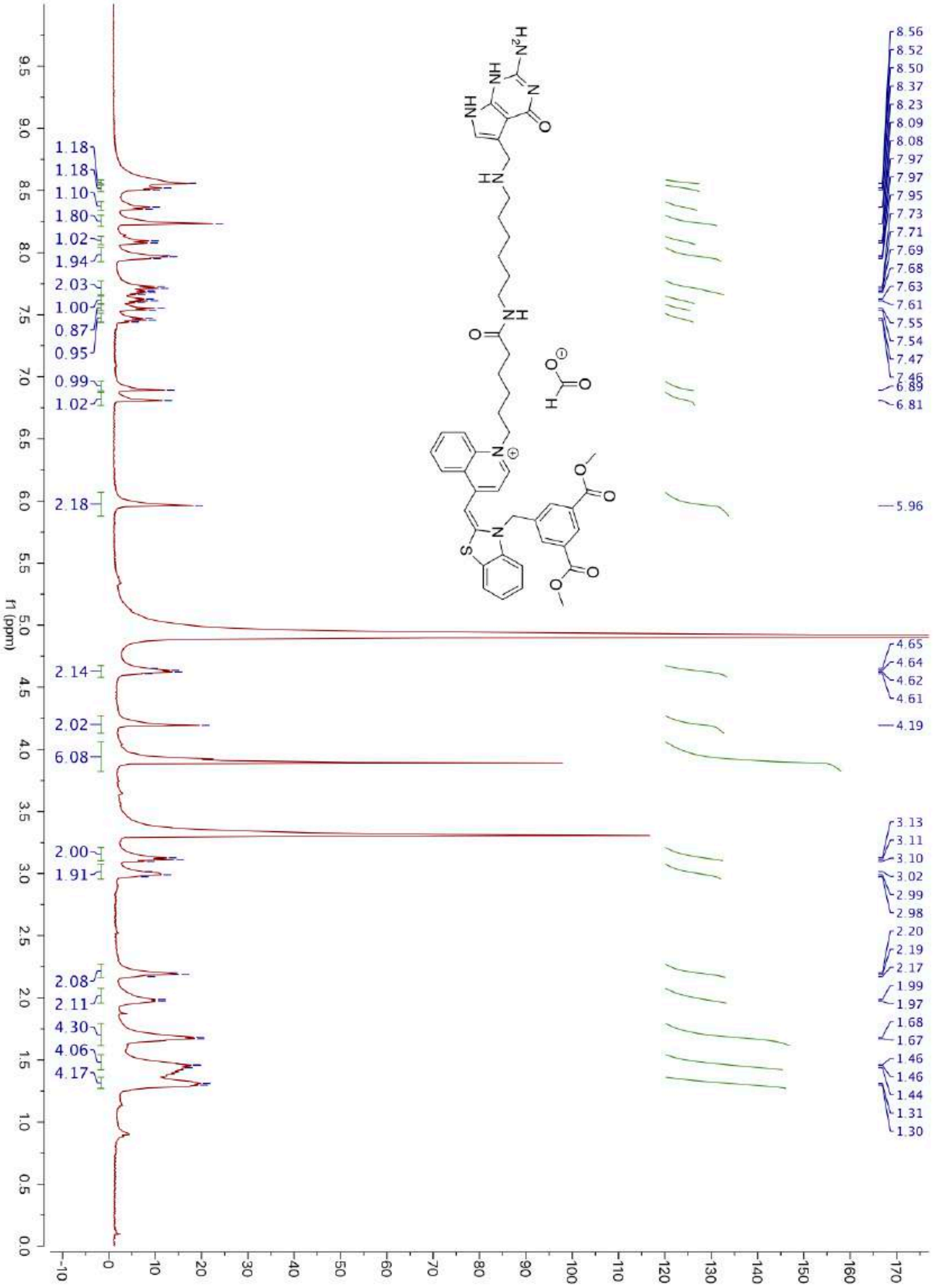

8.56
8.52
8.50
8.37
8.23
8.09
8.08
7.97
7.97
7.95
7.73
7.71
7.69
7.68
7.63
7.61
7.55
7.54
7.47
7.46
6.89
6.81
5.96
4.65
4.64
4.62
4.61
4.19
3.13
3.11
3.10
3.02
2.99
2.98
2.20
2.19
2.17
1.99
1.97
1.68
1.67
1.46
1.46
1.44
1.31
1.30
1.18
1.18
1.10
1.80
1.02
1.94
2.03
1.00
0.87
0.95
0.99
1.02
2.18
2.14
2.02
6.08
2.00
1.91
2.08
2.11
4.30
4.06
4.17
f1 (ppm)

6f

^{13}C NMR

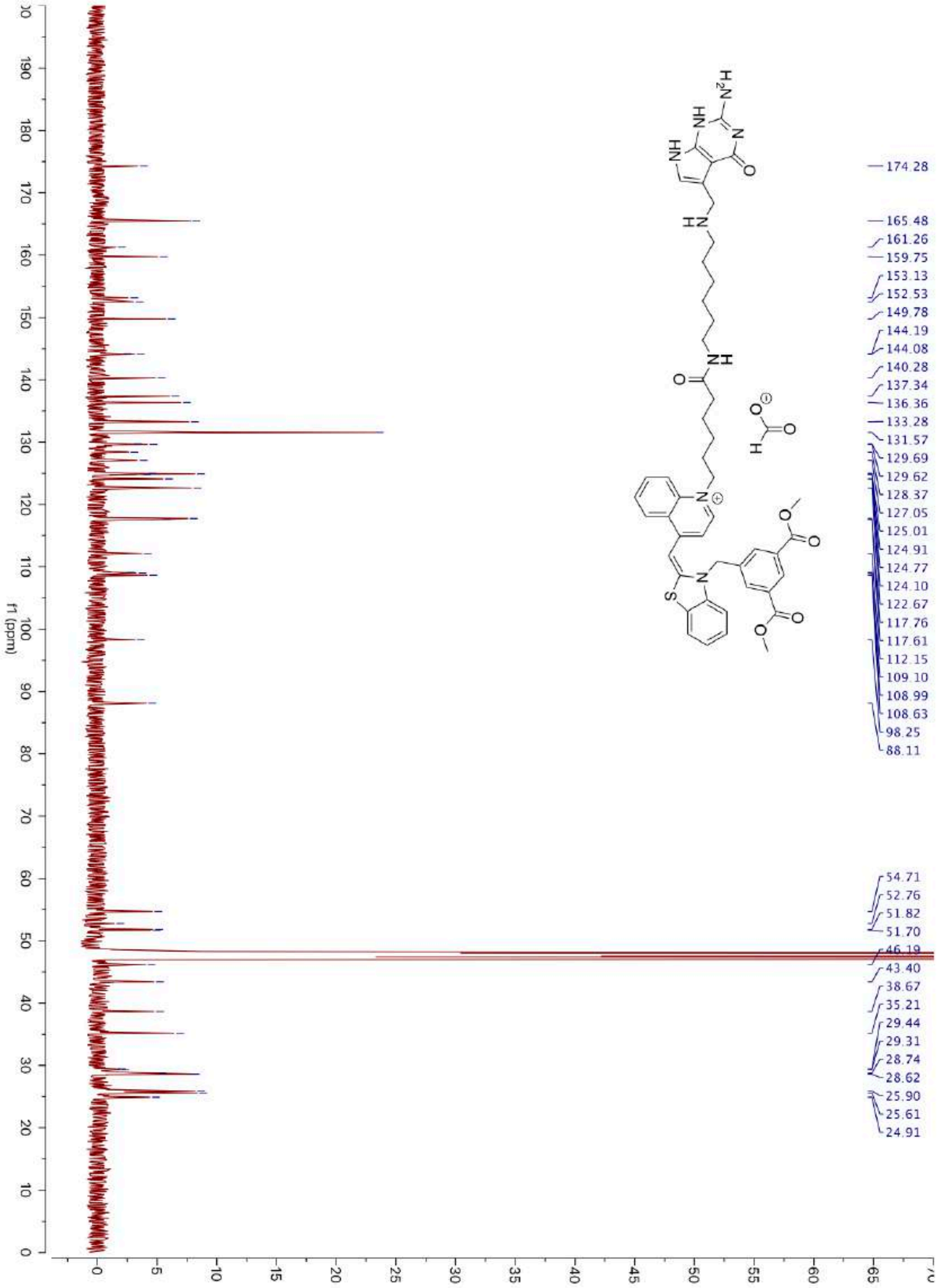
174.28
165.48
161.26
159.75
153.13
152.53
149.78
144.19
144.08
140.28
137.34
136.36
133.28
131.57
129.69
129.62
128.37
127.05
125.01
124.91
124.77
124.10
122.67
117.76
117.61
112.15
109.10
108.99
108.63
98.25
88.11
54.71
52.76
51.82
51.70
46.19
43.40
38.67
35.21
29.44
29.31
28.74
28.62
25.90
25.61
24.91
f1 (ppm)

7

^{1}H NMR

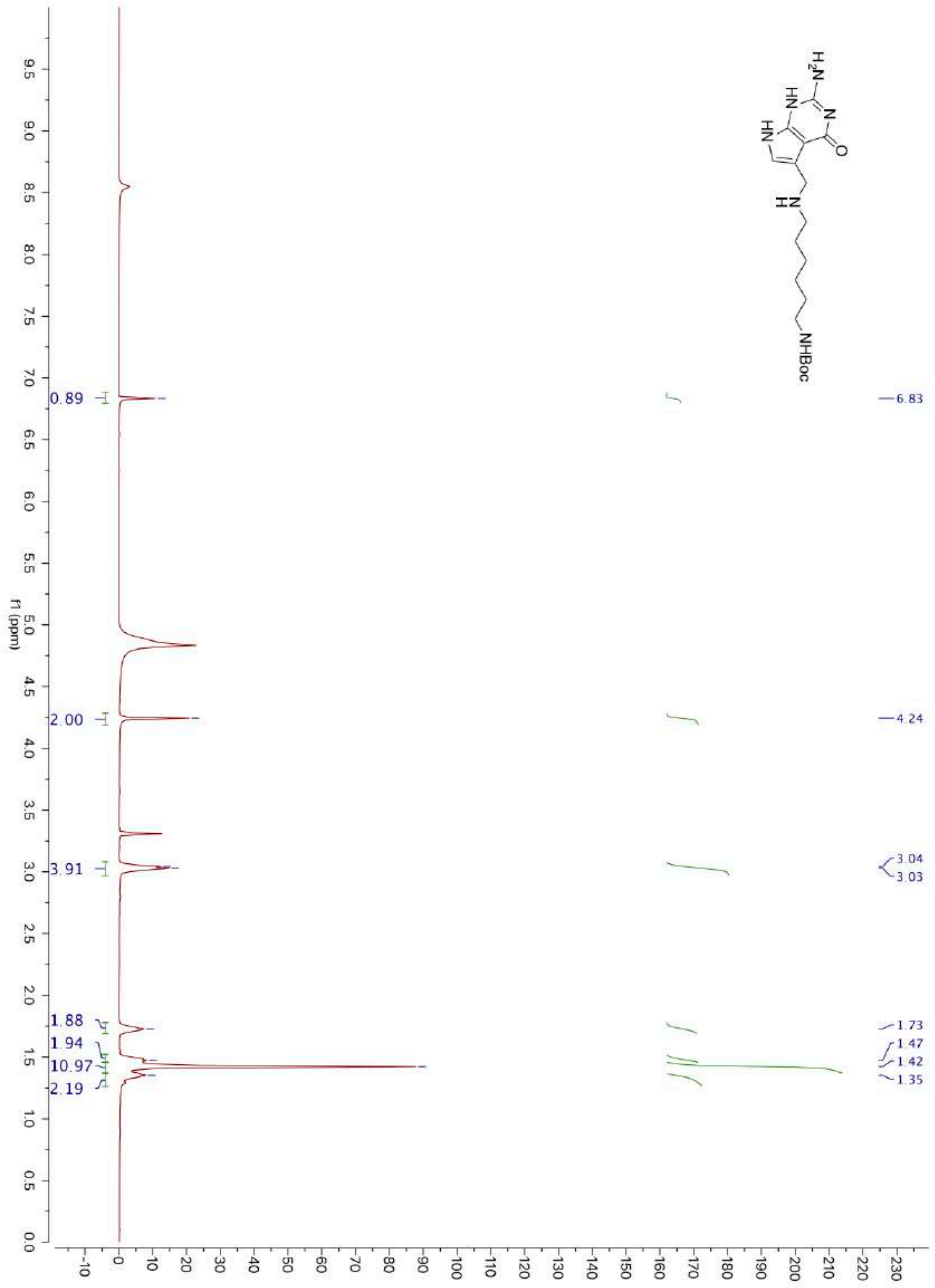

f1 (ppm)
NHBoc
6.83
4.24
3.04
3.03
1.73
1.47
1.42
1.35
0.89
2.00
3.91
1.88
1.94
10.97
2.19

7

^{13}C NMR

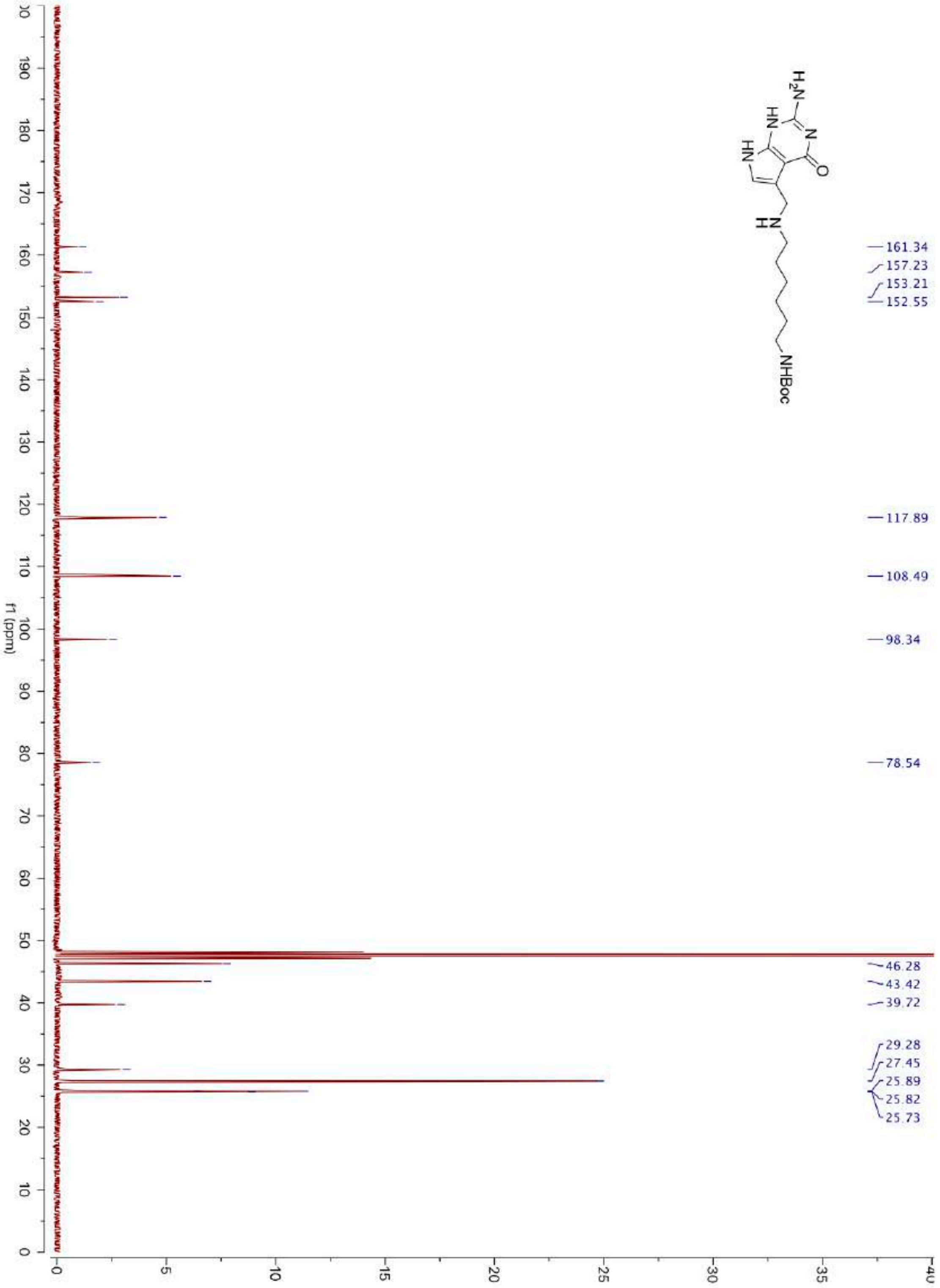

H2N
HN
N
HN
O
H
N
NHBoc
161.34
157.23
153.21
152.55
117.89
108.49
98.34
78.54
46.28
43.42
39.72
29.28
27.45
25.89
25.82
25.73
f1 (ppm)
